# Future and potential spending on health 2015–40: development assistance for health, and government, prepaid private, and out-of-pocket health spending in 184 countries

**DOI:** 10.1016/S0140-6736(17)30873-5

**Published:** 2017-05-20

**Authors:** Global Burden of Disease Health Financing Collaborator Network, Joseph L Dieleman, Madeline Campbell, Abigail Chapin, Erika Eldrenkamp, Victoria Y Fan, Annie Haakenstad, Jennifer Kates, Zhiyin Li, Taylor Matyasz, Angela Micah, Alex Reynolds, Nafis Sadat, Matthew T Schneider, Reed Sorensen, Kaja M Abbas, Semaw Ferede Abera, Aliasghar Ahmad Kiadaliri, Muktar Beshir Ahmed, Khurshid Alam, Reza Alizadeh-Navaei, Ala'a Alkerwi, Erfan Amini, Walid Ammar, Carl Abelardo T Antonio, Tesfay Mehari Atey, Leticia Avila-Burgos, Ashish Awasthi, Aleksandra Barac, Tezera Moshago Berheto, Addisu Shunu Beyene, Tariku Jibat Beyene, Charles Birungi, Habtamu Mellie Bizuayehu, Nicholas J K Breitborde, Lucero Cahuana-Hurtado, Ruben Estanislao Castro, Ferran Catalia-Lopez, Koustuv Dalal, Lalit Dandona, Rakhi Dandona, Samath D Dharmaratne, Manisha Dubey, Andé Faro, Andrea B Feigl, Florian Fischer, Joseph R Anderson Fitchett, Nataliya Foigt, Ababi Zergaw Giref, Rahul Gupta, Samer Hamidi, Hilda L Harb, Simon I Hay, Delia Hendrie, Masako Horino, Mikk Jürisson, Mihajlo B Jakovljevic, Mehdi Javanbakht, Denny John, Jost B Jonas, Seyed M Karimi, Young-Ho Khang, Jagdish Khubchandani, Yun Jin Kim, Jonas M Kinge, Kristopher J Krohn, G Anil Kumar, Ricky Leung, Hassan Magdy Abd El Razek, Mohammed Magdy Abd El Razek, Azeem Majeed, Reza Malekzadeh, Deborah Carvalho Malta, Atte Meretoja, Ted R Miller, Erkin M Mirrakhimov, Shafiu Mohammed, Gedefaw Molla, Vinay Nangia, Stefano Olgiati, Mayowa O Owolabi, Tejas Patel, Angel J Paternina Caicedo, David M Pereira, Julian Perelman, Suzanne Polinder, Anwar Rafay, Vafa Rahimi-Movaghar, Rajesh Kumar Rai, Usha Ram, Chhabi Lal Ranabhat, Hirbo Shore Roba, Miloje Savic, Sadaf G Sepanlou, Braden J Te Ao, Azeb Gebresilassie Tesema, Alan J Thomson, Ruoyan Tobe-Gai, Roman Topor-Madry, Eduardo A Undurraga, Veronica Vargas, Tommi Vasankari, Francesco S Violante, Tissa Wijeratne, Gelin Xu, Naohiro Yonemoto, Mustafa Z Younis, Chuanhua Yu, Zoubida Zaidi, Maysaa El Sayed Zaki, Christopher J L Murray

## Abstract

**Background:**

The amount of resources, particularly prepaid resources, available for health can affect access to health care and health outcomes. Although health spending tends to increase with economic development, tremendous variation exists among health financing systems. Estimates of future spending can be beneficial for policy makers and planners, and can identify financing gaps. In this study, we estimate future gross domestic product (GDP), all-sector government spending, and health spending disaggregated by source, and we compare expected future spending to potential future spending.

**Methods:**

We extracted GDP, government spending in 184 countries from 1980–2015, and health spend data from 1995–2014. We used a series of ensemble models to estimate future GDP, all-sector government spending, development assistance for health, and government, out-of-pocket, and prepaid private health spending through 2040. We used frontier analyses to identify patterns exhibited by the countries that dedicate the most funding to health, and used these frontiers to estimate potential health spending for each low-income or middle-income country. All estimates are inflation and purchasing power adjusted.

**Findings:**

We estimated that global spending on health will increase from US$9·21 trillion in 2014 to $24·24 trillion (uncertainty interval [UI] 20·47–29·72) in 2040. We expect per capita health spending to increase fastest in upper-middle-income countries, at 5·3% (UI 4·1–6·8) per year. This growth is driven by continued growth in GDP, government spending, and government health spending. Lower-middle income countries are expected to grow at 4·2% (3·8–4·9). High-income countries are expected to grow at 2·1% (UI 1·8–2·4) and low-income countries are expected to grow at 1·8% (1·0–2·8). Despite this growth, health spending per capita in low-income countries is expected to remain low, at $154 (UI 133–181) per capita in 2030 and $195 (157–258) per capita in 2040. Increases in national health spending to reach the level of the countries who spend the most on health, relative to their level of economic development, would mean $321 (157–258) per capita was available for health in 2040 in low-income countries.

**Interpretation:**

Health spending is associated with economic development but past trends and relationships suggest that spending will remain variable, and low in some low-resource settings. Policy change could lead to increased health spending, although for the poorest countries external support might remain essential.

**Funding:**

Bill & Melinda Gates Foundation.

## Introduction

Anticipation of future health spending and the source of that funding is vital for effective health policy. With reliable spending forecasts, decision makers can adjust long-term planning and processes. Investments can be made strategically to counter shortfalls or enhance growth in coming years. Because dependence on out-of-pocket health payments has been shown to reduce access to health services and increase medical impoverishment in some settings, understanding how funds will be collected, and if they will be prepaid and pooled across groups, is also of crucial importance.^1^, ^2^, ^3^, ^4^, ^5^, ^6^, ^7^, ^8^ The source of health funding often dictates the types of services and supplies procured and how efficiently those resources are deployed.^9^, ^10^, ^11^, ^12^, ^13^ Without careful planning, limited resources for health can translate into insufficient access to health services and an over-reliance on out-of-pocket payments.^14^

The health financing transition describes how health financing changes, on average, as countries develop economically: per capita health spending increases and out-of-pocket expenses comprise a smaller share of total health expenditure than previously.^15^ However, tremendous variation in health financing systems and the associated levels of financing underpins these trends. In 2014, spending per capita in low-income countries varied from US$33 to $347, and per capita spending in high-income countries varied from $853 to $9237. The health financing transition is not guaranteed to continue as new countries progress through various stages of development. Prospective health spending estimates show what past trends and relationships suggest regarding future spending and sources of those funds.

**Research in context**

**Evidence before this study**

Forecasts of total health spending, and health spending disaggregated by source into government spending, out of pocket, prepaid private, and development assistance for health are crucial inputs into health-system planning. Understanding of the opportunity to alter these probable trajectories through plausible increases in the share of gross domestic product (GDP) spent by government or the share of government expenditure spent on health to expand fiscal space for health has also become an important dimension of health policy in the era of Sustainable Development Goals.

Country-specific forecasts have been developed for a few countries. The Organisation for Economic Co-operation and Development periodically produces forecasts to 2060 for its member states and the Brazil, Russia, India, China, and South Africa. The only comprehensive set of health expenditure forecasts covering a comprehensive set of countries has been produced by Dieleman and colleagues in 2016.

**Added value of this study**

This study advances our previous assessment of future health spending in three ways. First, a key driver of future health spending in total and by source is economic development, often measured by GDP per capita. Given that there is no regularly updated set of GDP forecasts that extends to 2040 and covers all countries with similar methods, we developed GDP forecasts. To improve on previous methods used to forecast GDP and follow good forecast practice applied in other fields, we switched to forecasting GDP using an ensemble of models. We developed 1664 models and selected the 136 that met predetermined inclusion criteria and had the best out-of-sample performance. These revised GDP forecasts are more optimistic than previous estimates for Luxembourg, Qatar, and especially China. Second, we modelled the share of GDP spent by government to derive all-sector government spending estimates. These forecasts allowed us to estimate government health spending as a share of all-sector government spending. These techniques better reflect the reality that health spending by government is constrained by the size of government. This two-stage modelling of government spending captures the direct competition for scarce government resources between sectors. Third, we studied the potential of low-income and middle-income countries to increase the amount spent on health by increasing the share of GDP spent by the government, increasing the share of government budgets spent on health, or both. This exploration of the fiscal space to increase health spending was not previously completed, and was done empirically by fitting a frontier to the observed spending patterns at each level of development. This study is the first, to our knowledge, to provide a prospective empirical assessment of the potential to increase health spending in all low-income and middle-income countries.

**Implications of all the available evidence**

Because of more optimistic forecasts of GDP from our ensemble modelling approach for low-income and middle-income countries and the ability to constrain estimates of government health spending to a plausible share of all-sector government spending, we have increased our forecasts of health expenditure in low-income countries from $34–357 per capita to $42–384. Despite these shifts, spending as a share of GDP will remain low and it is likely that small growth in development assistance for health will not fill the gap. Our assessment of fiscal space shows that although the optimal policy options vary by country, there is substantial potential to increase health expenditure if countries can achieve the levels of GDP spent by the government and the share of government budgets spent on health of some countries at the same level of development.

Development assistance for health is no longer an expanding resource for developing country health budgets. Tepid growth in this area since 2010 suggests that external funding will not grow at the rate seen earlier in the millennium. This prediction intensifies the need to increase domestic spending on health in some of the poorest countries. Fiscal space analyses have been done in a number of these countries to help prepare for the slowing down of development assistance for health growth.^16^, ^17^ However, few studies have comprehensively and empirically assessed what forecasts of future income mean for government health spending and other sources of health financing.^18^, ^19^, ^20^, ^21^ Among the forecasting studies that do exist,^22^, ^23^, ^24^ few assess mechanisms that alter financing trajectories, such as future macroeconomic scenarios, changes in country prioritisation, technological advancements, and other developments.

The objective of this study is to empirically assess how existing health financing trends and relationships could be shifted and, more generally, how the need for health resources can be met in an ever-evolving global economy. Using novel methods, we estimated future gross domestic product (GDP), all-sector (also known as general) government spending, and health spending through to year 2040. We then assessed alternative scenarios in health financing, highlighting how fiscal policy changes (in government spending levels and the allocation of those resources) could affect future health spending. Together, economic forecast indicators and health spending estimates show expected and potential health expenditure, which are essential inputs to decision making as the global context becomes increasingly uncertain.

## Methods

### Overview

**Figure 1:**
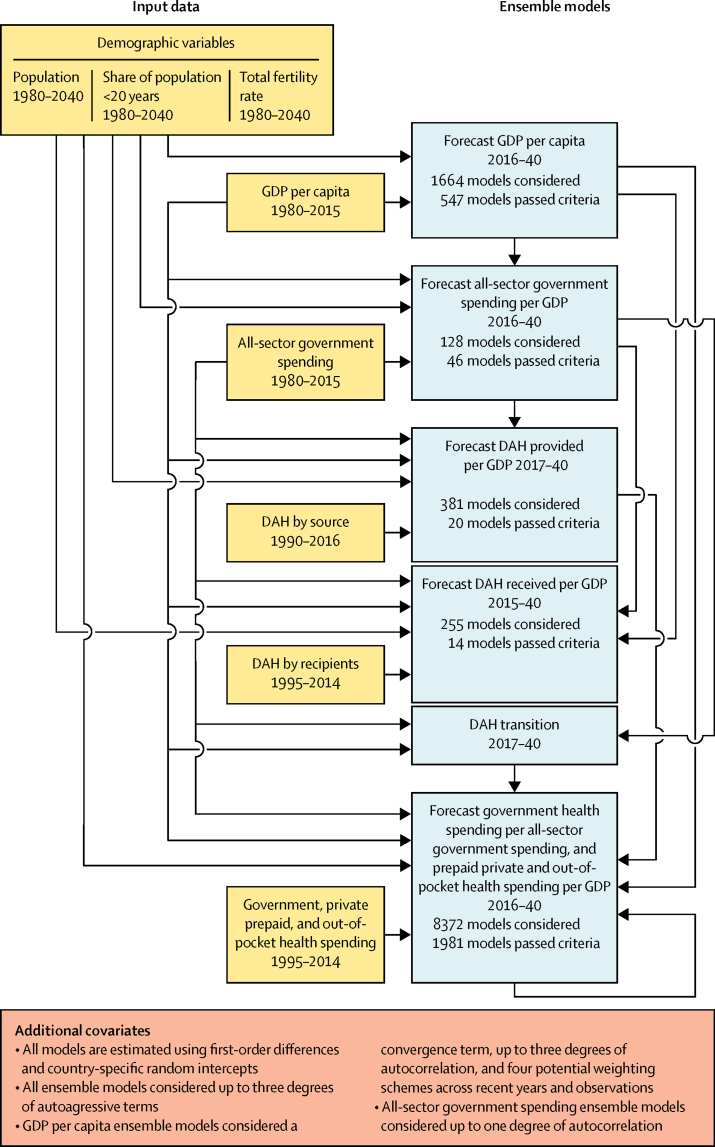
Process diagram for estimating future GDP, all-sector government spending, and health spending by source The process diagram indicates the data used by each ensemble for estimating future GDP, all-sector government spending, government health spending, prepaid private health spending, out-of-pocket health spending, or DAH. The number of models considered is the universe of specific model specifications considered for that ensemble model. Each individual model was tested against three exclusion criteria. The number of models that passed each criterion is also indicated. DAH=development assistance for health. GDP=gross domestic product.

We estimated national GDP, all-sector government spending, and health spending for each year through to 2040 for 184 countries based on past trends of the relationships between demographic and financial data over time. Future health spending was estimated by source: government, out-of-pocket, and prepaid private health spending as well as developmental assistance for health received. These four source-specific spending estimates were aggregated to form total national health spending. All projections were similar and consistent, and were based on ensemble models. Ensemble models are a standard in some areas of forecasting and rely on the estimation of many individual models and pooling the results to form a single estimate with uncertainty intervals [UIs]. These types of models have been shown to be more accurate than traditional single specification models in some circumstances.^25^, ^26^, ^27^ Additionally, our models incorporated codependencies, such that macroeconomic variables and each of the health spending variables affected each other. These methods build on previously published research with substantive improvements and are described more thoroughly in the appendix.^28^ Figure 1 outlines the processes used to estimate future GDP, all-sector government spending, and health spending by source.

### Data

We extracted health spending data for 184 countries spanning 1995 to 2014 from the Institute for Health Metrics and Evaluation's Financing Global Health 2016 database.^14^, ^29^ These data track government health spending from domestic sources, including general budget support and social health insurance; prepaid private health spending, which includes private insurance and non-governmental organisation spending; out-of-pocket health spending, which includes all spending at point-of-service and copayments; and developmental assistance for health. The data were collated and missing values (1·7% of the government spending, 14·8% of prepaid private spending, and 1·7% of out-of-pocket health spending) were imputed with multiple imputation methods from Amelia II: a program for missing data in R.^30^ The final series of data were mutually exclusive and exhaustive estimates of total health spending in each country.

GDP and all-sector government spending data spanning 1980 to 2015 were based on data collected from the International Monetary Fund, the UN, the Maddison Project, and Penn World Tables database.^31^, ^32^, ^33^, ^34^, ^35^ These data were combined with use of regression methods and previously developed for producing a complete GDP time series.^36^ All health spending, GDP, and all-sector government spending estimates from this database were reported in inflation-adjusted 2015 purchasing power parity adjusted US$.

### Estimating future GDP

We used an ensemble model that capitalised on past trends and relationships to predict GDP for 184 countries from 2016 through 2040.^28^ These models are based on data from 1980 to 2015. Altogether, 1664 models were considered to estimate the future growth rate of GDP, measured as the difference in natural log-transformed GDP. The independent variables considered were total population, share of the population younger than 20 years of age, total fertility rate, and a convergence term, which is the 1 year lag of the non-differenced dependent variable. The 1664 models included all combinations of independent variables. More specific information about the universe of models, precise model specifications, and estimated coefficients are included in the appendix.

All models were assessed against three exclusion criteria. First, we excluded models with any independent variable that was not statistically significant (α=0·1). Second, we excluded models that estimated a coefficient greater than zero for the convergence term. Third, we excluded models that produced predictions that fell outside the bounds of growth observed in the underlying data (1980–2015; appendix). After implementation of these exclusion criteria, 547 models remained.

Of these 547 models, country forecasts were based on the best performing 25% of models (136 models). The best performing models were identified by the country-specific out-of-sample validation based on root-mean-squared error. To compute this, 10 years of observed data (2006–15) were withheld, the 547 models were rerun, and predicted values for 2006–15 were compared against actual values. The 136 models selected for each country-specific and year-specific models were rerun on the entire observed data set (1980–2015) to maximise use of observed data.

Uncertainty was propagated in three ways. First, we used the ensemble modelling framework to incorporate model uncertainty. Second, we took 74 random draws from the estimated variance-covariance matrix of each model to create more than 10 000 draws to incorporate parameter uncertainty. (74 random draws was the smallest number of draws that could be used for each of the 136 models to ensure at least 10 000 total draws.) Lastly, we added correlated periods of growth or recession across countries to model global recessions, and also added country-specific and year-specific periods of growth and recession to model otherwise unexpected country-specific growth or recession. We report a point-estimate, and lower and upper confidence interval based on the mean, 2·5th and 97·5th percentile of the 10 064 draws.

### Estimating future all-sector government spending and health spending by source

All-sector government spending, out-of-pocket health spending, and prepaid private health spending were each modelled as a share of GDP with the same method used to estimate future GDP. Government health spending was modelled as a share of all-sector government spending with the same methods. For each of these models, GDP per capita (natural log-transformed) was included as a potential independent variable in the ensemble. For each of the three health spending ensembles, a 1 year lag of the other health spending variables and all-sector government spending per capita (natural log-transformed) was also included in the ensemble to ensure codependence across the health spending estimates.

We used a three-step process to estimate the amount of future development assistance for health disbursed to each low-income or middle-income country. These methods were based on previously published research.^37^ First, we extracted development assistance for health provided by 24 major sources of development assistance and modelled development assistance for health provided as a share of the source's GDP to make estimates of total development assistance for health provided through 2040. These sources of development assistance for health are generally national treasuries, for example, those of the USA or UK, or major donors such as the Bill & Melinda Gates Foundation. Second, we modelled development assistance for health received, measured as a share of the total amount of development assistance for health provided to each low-income or middle-income country through 2040. Finally, we estimated the transition of countries from middle-income to high-income status on the basis of GDP per capita estimates. This transition, estimated to be when GDP per capita surpasses $18 108 per capita, marks the point at which, according to our definition of development assistance for health, a country is no longer eligible to receive development assistance for health. To estimate expected total health spending, we summed development assistance for health received and government, prepaid private, and out-of-pocket health spending.

### Potential health and government health spending

To estimate potential health spending in low-income and middle-income countries, we used stochastic frontier analysis. In our analysis, this frontier represents the amount of spending generated by the countries with the most health spending given their level of economic development. In this case, the frontier represents potential spending, based on a country's GDP per capita and peers' health spending. For our frontier analyses, we assumed a half-normal distribution of residual, although the appendix shows robustness analyses exploring the effect of alternative assumptions. This analysis was completed with only low-income and middle-income countries because very few high-income countries are concerned with increasing spending on health.

We report potential total health spending per capita for low-income and middle-income countries by estimating the spending on health that would result if countries increased spending to the frontier level. Potential spending is greater than actual spending for most, but not all, countries, because the frontier level is above most country-specific expected spending levels. The distance between a country's expected (forecasted) spending level and the frontier represents potential increases in health spending.

Finally, we used an additional set of frontiers to analyse three policy scenarios that could be used to increase government health spending in low-income and middle-income countries. In particular, we assessed how an increase in government spending and a reprioritisation of government spending towards the health sector could separately and cumulatively increase government health spending. The first scenario supposed that governments are able to raise all-sector government spending, measured as a share of GDP, to reflect their highest spending peer. The second scenario supposed that governments are able to prioritise the health sector like their highest spending peers. And the third scenario supposed that governments are able to generate all-sector government spending in addition to prioritising the health sector like their highest spending peers. In each of these scenarios, the highest spending peers are identified using the frontier analysis. Precise specifications of these models are included in the appendix. Because it is more plausible that these gains could be made as a result of long-term policy changes, this analysis focused on the effect of health spending in 2040. All estimation and analysis was completed with Stata (version 13.1) and R (version 3.3.2).

We report expected and potential spending estimates for each country, and for World Bank income groups and Global Burden of Disease super regions. Per capita and per GDP estimates reflect the entire group, meaning is the sum of spending divided by the sum of denominators World Bank income groups are four mutually exclusive categories assigned by the World Bank and based primarily on gross national income. Global burden of disease super regions are seven mutually exclusive categories based on geography and cause of death patterns.

### Role of the funding source

The funder of the study had no role in study design, data collection, data analysis, data interpretation, or writing of the report. All authors had full access to the data in the study and JLD and CJLM had final responsibility for the decision to submit the manuscript.

## Results

Table 1 presents data for health spending in 2014 and expected health spending in 2030 and 2040. These are shown in per capita terms and as a proportion of GDP. In 2014, $9·21 trillion was spent on health worldwide. Past trends and relationships suggest that, in 2030, $16·04 trillion (UI 14·50–17·78) will be spent on health and, in 2040, $24·24 trillion (20·47–29·72) will be spent on health. In per capita terms, this growth is from $1279 in 2014 to $2872 (UI 2426–3522) in 2040, with an annualised rate of growth of 3·0% (2·4–3·8).

**Table 1:** Expected health spending in 2030 and 2040

		**2014**		**2030**		**2040**		**2014–40**
		Health spending per capita ($)	Health spending per GDP (%)	Health spending per capita ($)	Health spending per GDP (%)	Health spending per capita ($)	Health spending per GDP (%)	Annualised rate of change, health spending per capita (%)
Global		1279	8·3%	1983 (1793 to 2199)	8·2% (7·4 to 9·1)	2872 (2426 to 3522)	8·2% (7·0 to 10·1)	3·0% (2·4 to 3·8)
Income group								
	High income	5221	11·7%	7334 (6786 to 7815)	12·5% (11·5 to 13·3)	9215 (8475 to 9967)	13·1% (12·0 to 14·2)	2·1% (1·8 to 2·4)
	Upper-middle income	914	5·9%	2072 (1698 to 2583)	6·4% (5·2 to 7·9)	3903 (2770 to 5741)	6·9% (4·9 to 10·1)	5·3% (4·1 to 6·8)
	Lower-middle income	267	4·3%	525 (485 to 582)	4·7% (4·3 to 5·1)	844 (739 to 1004)	5·0% (4·4 to 6·0)	4·2% (3·8 to 4·9)
	Low income	120	7·3%	154 (133 to 181)	6·6% (5·8 to 7·8)	195 (157 to 258)	6·7% (5·4 to 8·9)	1·8% (1·0 to 2·8)
GBD super region								
	Central Europe, eastern Europe, and central Asia	1364	6·7%	1877 (1766 to 2018)	6·9% (6·5 to 7·4)	2417 (2252 to 2637)	7·1% (6·6 to 7·7)	2·1% (1·9 to 2·4)
	GBD high income	5460	12·3%	7643 (7076 to 8146)	13·1% (12·1 to 14·0)	9556 (8791 to 10337)	13·8% (12·7 to 14·9)	2·1% (1·8 to 2·4)
	Latin America and Caribbean	1082	7·3%	1534 (1350 to 1745)	8·2% (7·2 to 9·3)	2047 (1720 to 2494)	8·9% (7·5 to 10·8)	2·3% (1·7 to 3·1)
	North Africa and Middle East	870	5·2%	1246 (1137 to 1416)	5·8% (5·3 to 6·6)	1630 (1431 to 1975)	6·3% (5·5 to 7·6)	2·3% (1·8 to 3·0)
	South Asia	223	4·2%	529 (467 to 619)	4·8% (4·2 to 5·6)	935 (773 to 1203)	5·3% (4·4 to 6·8)	5·3% (4·6 to 6·2)
	Southeast Asia, east Asia, and Oceania	588	4·8%	1867 (1436 to 2471)	5·6% (4·3 to 7·4)	4035 (2640 to 6314)	6·3% (4·1 to 9·9)	7·0% (5·6 to 8·8)
	Sub-Saharan Africa	218	5·9%	259 (238 to 286)	5·6% (5·2 to 6·2)	307 (269 to 365)	5·7% (5·0 to 6·8)	1·3% (0·8 to 1·9)
Country								
	Afghanistan	159	9·7%	201 (161 to 268)	10·2% (8·1 to 13·6)	249 (179 to 388)	10·6% (7·6 to 16·5)	1·6% (0·4 to 3·3)
	Albania	642	5·9%	1202 (1022 to 1424)	6·6% (5·6 to 7·8)	1733 (1404 to 2144)	6·7% (5·5 to 8·3)	3·7% (2·9 to 4·5)
	Algeria	1004	7·2%	1567 (1248 to 2146)	9·1% (7·2 to 12·4)	2080 (1439 to 3337)	10·4% (7·2 to 16·6)	2·6% (1·3 to 4·4)
	Andorra	5723	8·1%	7230 (5789 to 8606)	8·6% (6·9 to 10·3)	8357 (5791 to 10773)	8·7% (6·1 to 11·3)	1·4% (0·0 to 2·3)
	Angola	228	3·0%	256 (169 to 321)	2·5% (1·7 to 3·1)	308 (154 to 414)	2·5% (1·2 to 3·3)	1·0% (−1·5 to 2·2)
	Antigua and Barbuda	1213	5·5%	2165 (1727 to 2767)	7·4% (5·9 to 9·4)	2987 (2175 to 4321)	8·5% (6·2 to 12·4)	3·3% (2·2 to 4·7)
	Argentina	1322	4·8%	2177 (1769 to 2985)	5·7% (4·6 to 7·8)	3012 (2202 to 4807)	6·2% (4·6 to 10·0)	3·0% (1·9 to 4·8)
	Armenia	395	4·5%	674 (549 to 907)	4·9% (4·0 to 6·7)	997 (727 to 1578)	5·3% (3·9 to 8·4)	3·4% (2·3 to 5·1)
	Australia	4032	9·0%	5606 (5186 to 6165)	9·7% (9·0 to 10·7)	6970 (6206 to 8111)	10·2% (9·1 to 11·9)	2·0% (1·6 to 2·6)
	Austria	5471	11·2%	7416 (6788 to 8143)	11·6% (10·6 to 12·7)	9257 (8270 to 10607)	12·0% (10·8 to 13·8)	1·9% (1·5 to 2·5)
	Azerbaijan	1030	5·9%	1734 (1524 to 1978)	6·3% (5·5 to 7·2)	2502 (2033 to 3062)	6·5% (5·3 to 7·9)	3·3% (2·5 to 4·0)
	Bahrain	2258	4·8%	3289 (2738 to 4136)	5·3% (4·4 to 6·7)	4380 (3426 to 6336)	5·8% (4·5 to 8·4)	2·4% (1·5 to 3·8)
	Bangladesh	92	2·9%	173 (149 to 198)	2·8% (2·4 to 3·2)	266 (206 to 327)	2·8% (2·2 to 3·5)	3·9% (3·0 to 4·7)
	Barbados	1116	7·5%	1641 (1412 to 1926)	8·7% (7·5 to 10·2)	2155 (1705 to 2736)	9·5% (7·5 to 12·0)	2·4% (1·6 to 3·3)
	Belarus	1093	5·6%	1825 (1432 to 2308)	7·0% (5·5 to 8·9)	2369 (1648 to 3243)	7·4% (5·1 to 10·1)	2·8% (1·5 to 4·0)
	Belgium	4751	10·6%	6437 (5759 to 7278)	11·2% (10·0 to 12·7)	8005 (6950 to 9572)	11·7% (10·2 to 14·0)	1·9% (1·4 to 2·6)
	Belize	503	5·8%	678 (593 to 776)	6·3 %(5·5 to 7·2)	844 (703 to 1017)	6·6% (5·5 to 8·0)	1·9% (1·2 to 2·6)
	Benin	105	5·1%	169 (134 to 221)	6·2% (4·9 to 8·1)	232 (161 to 357)	7·3% (5·0 to 11·2)	2·8% (1·6 to 4·5)
	Bhutan	279	3·6%	563 (397 to 774)	3·5% (2·5 to 4·8)	940 (517 to 1558)	3·6% (2·0 to 5·9)	4·4% (2·3 to 6·4)
	Bolivia	404	6·3%	673 (565 to 814)	7·3% (6·1 to 8·8)	943 (736 to 1252)	8·0% (6·3 to 10·7)	3·1% (2·2 to 4·2)
	Bosnia and Herzegovina	992	9·5%	1734 (1331 to 2104)	10·4% (8·0 to 12·6)	2613 (1921 to 3416)	11·6% (8·6 to 15·2)	3·5% (2·4 to 4·6)
	Botswana	903	5·5%	1395 (1168 to 1723)	6·3% (5·2 to 7·7)	1878 (1452 to 2524)	6·8% (5·3 to 9·2)	2·7% (1·8 to 3·8)
	Brazil	1357	8·3%	1994 (1657 to 2402)	10·0% (8·3 to 12·1)	2770 (2150 to 3708)	11·3% (8·7 to 15·1)	2·6% (1·7 to 3·7)
	Brunei	1811	2·6%	2254 (1741 to 3135)	3·5% (2·7 to 4·8)	2612 (1859 to 4315)	4·0% (2·8 to 6·5)	1·2% (0·1 to 3·2)
	Bulgaria	1490	8·4%	2659 (2116 to 3624)	9·7% (7·7 to 13·2)	3870 (2896 to 5754)	10·7% (8·0 to 15·9)	3·5% (2·5 to 5·0)
	Burkina Faso	83	5·0%	108 (93 to 127)	5·0% (4·3 to 5·9)	128 (101 to 168)	5·1% (4·0 to 6·6)	1·6% (0·7 to 2·6)
	Burundi	65	8·3%	85 (62 to 120)	9·6% (7·0 to 13·6)	104 (65 to 176)	10·1% (6·3 to 17·1)	1·6% (0·0 to 3·7)
	Cambodia	209	6·4%	397 (352 to 448)	6·0% (5·3 to 6·7)	642 (543 to 760)	6·1% (5·2 to 7·2)	4·1% (3·5 to 4·8)
	Cameroon	116	4·0%	156 (135 to 179)	4·1% (3·5 to 4·7)	190 (150 to 238)	4·3% (3·4 to 5·4)	1·8% (0·9 to 2·6)
	Canada	4576	10·3%	5926 (5389 to 6601)	10·7% (9·7 to 11·9)	7248 (6516 to 8528)	11·1% (10·0 to 13·1)	1·7% (1·3 to 2·3)
	Cape Verde	318	4·8%	529 (412 to 686)	4·8% (3·8 to 6·3)	768 (523 to 1124)	5·0% (3·4 to 7·4)	3·2% (1·8 to 4·7)
	Central African Republic	35	5·7%	46 (29 to 77)	9·4% (6·0 to 15·8)	58 (25 to 145)	13·8% (6·0 to 34·2)	1·5% (−1·2 to 5·3)
	Chad	89	3·8%	111 (74 to 150)	3·9% (2·6 to 5·3)	138 (75 to 212)	4·2% (2·3 to 6·4)	1·5% (−0·7 to 3·2)
	Chile	1780	7·8%	3217 (2622 to 3793)	8·8% (7·1 to 10·3)	4791 (3724 to 6105)	9·5% (7·4 to 12·1)	3·6% (2·7 to 4·6)
	China	697	5·1%	2493 (1851 to 3402)	6·0% (4·5 to 8·2)	5703 (3571 to 9218)	6·7% (4·2 to 10·8)	7·7% (6·1 to 9·6)
	Colombia	975	7·2%	1620 (1168 to 2206)	7·8% (5·7 to 10·7)	2398 (1616 to 3727)	8·5% (5·7 to 13·2)	3·2% (1·9 to 5·0)
	Comoros	111	7·1%	121 (101 to 148)	8·6% (7·1 to 10·5)	132 (96 to 184)	9·8% (7·1 to 13·6)	0·6% (−0·5 to 1·9)
	Congo (Brazzaville)	312	5·2%	424 (336 to 543)	6·1% (4·8 to 7·8)	544 (394 to 736)	7·1% (5·1 to 9·6)	2·0% (0·9 to 3·2)
	Costa Rica	1418	9·3%	2142 (1628 to 2636)	9·0% (6·8 to 11·1)	3050 (2207 to 4077)	9·3% (6·8 to 12·5)	2·8% (1·6 to 3·9)
	Côte d'Ivoire	179	5·3%	242 (214 to 275)	5·4% (4·8 to 6·1)	292 (246 to 352)	5·6% (4·7 to 6·7)	1·8% (1·2 to 2·5)
	Croatia	1734	7·8%	2263 (2064 to 2445)	7·8% (7·1 to 8·5)	2795 (2482 to 3032)	8·2% (7·3 to 8·9)	1·8% (1·3 to 2·1)
	Cuba	1706	11·1%	2326 (1635 to 3134)	11·3% (7·9 to 15·2)	3097 (2091 to 4454)	12·3% (8·3 to 17·7)	2·1% (0·8 to 3·6)
	Cyprus	2019	7·2%	2864 (2520 to 3352)	8·0% (7·0 to 9·4)	3655 (3021 to 4619)	8·7% (7·2 to 10·9)	2·2% (1·5 to 3·1)
	Czech Republic	2384	7·4%	3146 (2753 to 3657)	7·1% (6·3 to 8·3)	3856 (3240 to 4708)	7·3% (6·2 to 9·0)	1·8% (1·1 to 2·5)
	DR Congo	46	4·5%	67 (52 to 86)	5·1% (3·9 to 6·6)	83 (56 to 123)	5·5% (3·8 to 8·2)	2·1% (0·8 to 3·7)
	Denmark	5075	10·8%	6251 (5488 to 6890)	10·7% (9·4 to 11·8)	7373 (5855 to 8735)	10·8% (8·6 to 12·8)	1·4% (0·5 to 2·0)
	Djibouti	357	10·9%	613 (486 to 838)	13·9% (11·0 to 18·9)	842 (598 to 1324)	15·6% (11·1 to 24·5)	3·1% (1·9 to 4·8)
	Dominica	599	5·5%	859 (740 to 1012)	6·2% (5·3 to 7·3)	1092 (874 to 1406)	6·6% (5·3 to 8·5)	2·2% (1·4 to 3·2)
	Dominican Republic	601	4·4%	1211 (930 to 1567)	4·9% (3·7 to 6·3)	1833 (1316 to 2498)	5·1% (3·7 to 6·9)	4·1% (2·9 to 5·3)
	Ecuador	1071	9·2%	1491 (1261 to 1758)	10·2% (8·6 to 12·0)	1935 (1534 to 2410)	11·0% (8·7 to 13·7)	2·2% (1·3 to 3·0)
	Egypt	581	5·4%	903 (820 to 1016)	5·5% (4·9 to 6·1)	1212 (1070 to 1453)	5·5% (4·9 to 6·6)	2·7% (2·3 to 3·4)
	El Salvador	567	6·8%	1018 (826 to 1354)	7·7% (6·3 to 10·3)	1520 (1089 to 2337)	8·6% (6·1 to 13·2)	3·6 %(2·4 to 5·2)
	Equatorial Guinea	1411	3·7%	1435 (1163 to 1792)	3·6% (2·9 to 4·5)	1746 (1302 to 2291)	3·8% (2·8 to 4·9)	0·8% (−0·3 to 1·8)
	Eritrea	59	5·1%	68 (53 to 88)	4·8% (3·7 to 6·2)	84 (56 to 129)	5·1% (3·4 to 7·9)	1·2% (−0·2 to 2·9)
	Estonia	1830	6·4%	3274 (2683 to 4230)	7·9% (6·5 to 10·2)	4554 (3386 to 6301)	8·7% (6·5 to 12·1)	3·3% (2·3 to 4·6)
	Ethiopia	85	5·5%	149 (115 to 197)	4·9% (3·8 to 6·5)	212 (153 to 311)	4·6% (3·3 to 6·7)	3·3% (2·2 to 4·8)
	Federated States of Micronesia	490	16·1%	608 (359 to 972)	17·2% (10·1 to 27·5)	767 (302 to 1703)	19·4% (7·7 to 43·1)	1·3% (−1·8 to 4·6)
	Fiji	399	4·5%	558 (503 to 614)	4·6% (4·1 to 5·0)	705 (630 to 804)	4·7% (4·2 to 5·4)	2·1% (1·7 to 2·6)
	Finland	3935	9·3%	5061 (4654 to 5562)	9·5% (8·8 to 10·5)	6209 (5648 to 6920)	9·9% (9·0 to 11·1)	1·7% (1·3 to 2·1)
	France	4589	11·3%	5963 (5487 to 6689)	11·6% (10·6 to 13·0)	7402 (6768 to 8671)	12·0% (11·0 to 14·1)	1·8% (1·4 to 2·4)
	Gabon	612	3·4%	985 (799 to 1248)	4·7% (3·9 to 6·0)	1336 (966 to 1900)	5·8% (4·2 to 8·2)	2·8% (1·7 to 4·2)
	Georgia	700	7·3%	1236 (1026 to 1427)	8·9% (7·4 to 10·3)	1608 (1268 to 1972)	9·2% (7·3 to 11·3)	3·1% (2·2 to 3·8)
	Germany	5356	11·2%	7612 (6630 to 8575)	12·0% (10·5 to 13·5)	9659 (8134 to 11311)	12·7% (10·7 to 14·8)	2·2% (1·5 to 2·8)
	Ghana	146	3·5%	218 (177 to 264)	3·7% (3·0 to 4·4)	288 (214 to 381)	3·8% (2·8 to 5·0)	2·5% (1·4 to 3·5)
	Greece	2170	8·1%	2833 (2484 to 3383)	8·3% (7·3 to 9·9)	3462 (2923 to 4570)	8·6% (7·3 to 11·4)	1·7% (1·1 to 2·8)
	Grenada	737	6·1%	1096 (967 to 1259)	6·3% (5·6 to 7·2)	1412 (1157 to 1755)	6·6% (5·4 to 8·2)	2·4% (1·7 to 3·2)
	Guatemala	466	6·2%	594 (540 to 648)	6·2% (5·6 to 6·7)	715 (622 to 808)	6·3% (5·4 to 7·1)	1·6% (1·1 to 2·0)
	Guinea	101	7·4%	127 (100 to 163)	7·9% (6·2 to 10·1)	165 (114 to 243)	8·9% (6·1 to 13·0)	1·8% (0·4 to 3·2)
	Guinea-Bissau	77	5·3%	98 (75 to 131)	5·7% (4·4 to 7·6)	115 (74 to 194)	6·0% (3·9 to 10·2)	1·4% (−0·1 to 3·4)
	Guyana	438	5·4%	685 (589 to 812)	5·8% (5·0 to 6·9)	903 (733 to 1142)	6·1% (5·0 to 7·7)	2·7% (1·9 to 3·5)
	Haiti	154	8·9%	205 (164 to 262)	9·4% (7·5 to 12·0)	250 (178 to 385)	9·6% (6·8 to 14·7)	1·7% (0·5 to 3·4)
	Honduras	420	8·8%	568 (513 to 654)	8·8% (8·0 to 10·1)	716 (625 to 887)	9·0% (7·9 to 11·2)	2·0% (1·5 to 2·8)
	Hungary	1855	7·2%	2706 (2522 to 3028)	7·3% (6·8 to 8·2)	3441 (3140 to 4128)	7·5% (6·9 to 9·0)	2·3% (1·9 to 3·0)
	Iceland	3959	8·7%	5491 (4824 to 6314)	9·2% (8·1 to 10·6)	6869 (5809 to 8455)	9·6% (8·1 to 11·8)	2·0% (1·4 to 2·8)
	India	253	4·5%	629 (550 to 747)	5·1% (4·4 to 6·0)	1138 (927 to 1488)	5·6% (4·6 to 7·3)	5·5% (4·8 to 6·6)
	Indonesia	265	2·5%	509 (443 to 588)	2·6% (2·3 to 3·0)	793 (640 to 986)	2·7% (2·2 to 3·4)	4·0% (3·3 to 4·9)
	Iran	1073	6·5%	1558 (1263 to 1874)	7·3% (5·9 to 8·8)	2051 (1489 to 2709)	7·8% (5·7 to 10·4)	2·4% (1·2 to 3·4)
	Iraq	828	5·7%	1018 (787 to 1401)	5·9% (4·6 to 8·2)	1230 (860 to 1897)	6·4% (4·5 to 9·9)	1·4% (0·1 to 3·1)
	Ireland	4006	7·6%	5989 (4758 to 7222)	7·8% (6·2 to 9·4)	7363 (5145 to 9737)	8·1% (5·7 to 10·7)	2·2% (0·9 to 3·3)
	Israel	2722	7·7%	3747 (3312 to 4249)	8·4% (7·4 to 9·5)	4534 (3695 to 5491)	8·7% (7·1 to 10·5)	1·9% (1·1 to 2·6)
	Italy	3311	9·0%	4154 (3805 to 4502)	8·8% (8·1 to 9·6)	5135 (4580 to 5713)	9·2% (8·2 to 10·2)	1·6% (1·2 to 2·0)
	Jamaica	477	5·4%	773 (650 to 955)	7·0% (5·9 to 8·6)	1000 (748 to 1399)	7·7% (5·8 to 10·8)	2·7% (1·7 to 4·0)
	Japan	3816	10·2%	5729 (4452 to 6820)	11·7% (9·1 to 13·9)	7695 (6122 to 9315)	13·0% (10·3 to 15·7)	2·6% (1·8 to 3·3)
	Jordan	839	7·4%	1097 (982 to 1226)	7·4% (6·6 to 8·3)	1335 (1144 to 1565)	7·6% (6·5 to 8·9)	1·7% (1·1 to 2·3)
	Kazakhstan	1143	4·3%	1545 (1343 to 1817)	4·2% (3·6 to 4·9)	2047 (1787 to 2500)	4·3% (3·8 to 5·3)	2·1% (1·7 to 2·9)
	Kenya	197	6·4%	237 (194 to 302)	5·9% (4·9 to 7·6)	286 (209 to 423)	6·1% (4·5 to 9·0)	1·3% (0·2 to 2·8)
	Kiribati	168	9·6%	184 (81 to 281)	9·9% (4·4 to 15·2)	214 (58 to 386)	10·8% (2·9 to 19·6)	0·5% (−3·9 to 3·1)
	Kuwait	2075	3·0%	3208 (2309 to 4950)	4·2% (3·0 to 6·5)	4368 (2792 to 8124)	4·9% (3·1 to 9·1)	2·6% (1·1 to 5·1)
	Kyrgyzstan	236	6·9%	315 (272 to 369)	7·4% (6·4 to 8·6)	384 (302 to 492)	7·7% (6·1 to 9·9)	1·8% (0·9 to 2·7)
	Laos	113	2·0%	186 (144 to 234)	1·5% (1·2 to 1·9)	285 (178 to 419)	1·4% (0·9 to 2·1)	3·3% (1·7 to 4·8)
	Latvia	1427	5·9%	2036 (1833 to 2247)	5·8% (5·2 to 6·4)	2564 (2246 to 2898)	5·8% (5·1 to 6·6)	2·2% (1·7 to 2·6)
	Lebanon	1060	6·4%	1484 (1222 to 1825)	6·3% (5·2 to 7·8)	1895 (1458 to 2499)	6·5% (5·0 to 8·5)	2·1% (1·2 to 3·2)
	Lesotho	319	11·6%	521 (371 to 667)	12·3% (8·8 to 15·8)	726 (464 to 1010)	13·0% (8·3 to 18·0)	3·0% (1·4 to 4·3)
	Liberia	345	39·3%	287 (257 to 333)	27·1% (24·3 to 31·4)	276 (224 to 373)	22·2% (18·0 to 29·9)	–0·9% (−1·6 to 0·3)
	Libya	751	5·0%	781 (534 to 1147)	6·8% (4·7 to 10·0)	979 (590 to 1637)	8·8% (5·3 to 14·7)	0·8% (−0·9 to 2·9)
	Lithuania	1830	6·5%	2904 (2579 to 3381)	6·6% (5·9 to 7·7)	3871 (3242 to 4809)	6·7% (5·6 to 8·3)	2·8% (2·1 to 3·6)
	Luxembourg	7105	6·9%	10 593 (9569 to 12306)	7·4% (6·7 to 8·6)	13 924 (11726 to 17 455)	7·9% (6·6 to 9·9)	2·5% (1·9 to 3·3)
	Macedonia	887	6·5%	1368 (1240 to 1504)	6·8% (6·2 to 7·5)	1742 (1549 to 1931)	6·9% (6·1 to 7·7)	2·5% (2·1 to 2·9)
	Madagascar	52	3·7%	65 (54 to 80)	4·2% (3·5 to 5·2)	73 (56 to 106)	4·4% (3·4 to 6·4)	1·3% (0·3 to 2·7)
	Malawi	148	12·9%	184 (148 to 233)	13·4% (10·8 to 17·0)	219 (160 to 320)	13·9% (10·1 to 20·2)	1·4% (0·3 to 2·9)
	Malaysia	1047	4·1%	1783 (1576 to 2102)	4·1% (3·6 to 4·8)	2528 (2099 to 3249)	4·1% (3·4 to 5·3)	3·2% (2·6 to 4·2)
	Maldives	1980	13·5%	3623 (2656 to 5154)	13·1% (9·6 to 18·6)	6070 (3725 to 9978)	13·9% (8·6 to 22·9)	4·0% (2·3 to 6·0)
	Mali	162	7·4%	229 (193 to 275)	7·3% (6·2 to 8·8)	300 (231 to 402)	7·9% (6·1 to 10·6)	2·2% (1·3 to 3·4)
	Malta	3058	9·7%	5997 (5097 to 7328)	12·1% (10·3 to 14·8)	8840 (6975 to 11 329)	13·5% (10·7 to 17·4)	3·9% (3·1 to 4·9)
	Marshall Islands	599	17·2%	679 (495 to 851)	15·7% (11·5 to 19·7)	785 (448 to 1130)	15·8% (9·0 to 22·7)	0·9% (−1·1 to 2·3)
	Mauritania	153	3·7%	204 (171 to 251)	4·0% (3·3 to 4·9)	258 (193 to 366)	4·4% (3·3 to 6·2)	1·9% (0·9 to 3·2)
	Mauritius	880	4·6%	1942 (1454 to 2542)	5·5% (4·1 to 7·2)	3459 (2435 to 5042)	6·4% (4·5 to 9·4)	5·0% (3·8 to 6·5)
	Mexico	1088	6·3%	1413 (1217 to 1611)	6·7% (5·8 to 7·7)	1726 (1403 to 2084)	7·1% (5·8 to 8·6)	1·7% (0·9 to 2·4)
	Moldova	527	10·3%	711 (620 to 822)	10·5% (9·1 to 12·1)	910 (755 to 1122)	10·7% (8·8 to 13·1)	2·0%(1·3 to 2·8)
	Mongolia	575	4·7%	1078 (837 to 1406)	4·7% (3·7 to 6·2)	1685 (1177 to 2462)	4·8% (3·4 to 7·0)	3·9% (2·7 to 5·4)
	Montenegro	1015	6·6%	1613 (1373 to 2074)	7·5% (6·4 to 9·6)	2189 (1734 to 3138)	8·2% (6·5 to 11·8)	2·8% (2·0 to 4·2)
	Morocco	505	5·9%	765 (700 to 833)	5·6% (5·2 to 6·1)	1056 (945 to 1160)	5·7% (5·1 to 6·2)	2·7% (2·3 to 3·1)
	Mozambique	92	7·8%	96 (62 to 142)	5·3% (3·4 to 7·8)	117 (59 to 222)	4·9% (2·5 to 9·3)	0·7% (−1·6 to 3·3)
	Myanmar	121	2·5%	394 (273 to 613)	3·3% (2·3 to 5·1)	979 (476 to 2210)	4·5% (2·2 to 10·1)	7·4% (5·1 to 10·8)
	Namibia	936	9·3%	1437 (1277 to 1692)	9·8% (8·7 to 11·5)	1929 (1590 to 2499)	10·2% (8·4 to 13·2)	2·7% (2·0 to 3·6)
	Nepal	138	5·8%	226 (197 to 259)	5·6% (4·9 to 6·5)	321 (263 to 388)	5·6% (4·6 to 6·7)	3·1% (2·4 to 3·8)
	Netherlands	5234	10·7%	7799 (6370 to 9036)	12·2% (10·0 to 14·2)	10 186 (8436 to 12 098)	13·4% (11·1 to 16·0)	2·5% (1·8 to 3·1)
	New Zealand	4050	11·0%	5496 (4595 to 6193)	11·4% (9·5 to 12·9)	6868 (5624 to 8063)	11·9% (9·8 to 14·0)	1·9% (1·2 to 2·5)
	Nicaragua	450	9·1%	652 (518 to 753)	9·3% (7·4 to 10·7)	830 (618 to 1005)	9·5% (7·1 to 11·5)	2·2% (1·2 to 3·0)
	Niger	66	6·7%	81 (66 to 101)	6·8% (5·6 to 8·5)	98 (73 to 139)	7·3% (5·4 to 10·4)	1·4% (0·4 to 2·8)
	Nigeria	225	3·7%	287 (245 to 343)	3·8% (3·2 to 4·5)	343 (268 to 449)	3·9% (3·0 to 5·1)	1·5% (0·6 to 2·6)
	Norway	6537	10·0%	9758 (8486 to 11 459)	11·6% (10·1 to 13·6)	12 734 (10 505 to 16 034)	12·7% (10·5 to 16·0)	2·4% (1·8 to 3·3)
	Oman	1467	3·5%	2507 (1908 to 4034)	4·5% (3·4 to 7·2)	3631 (2369 to 7390)	5·2% (3·4 to 10·5)	3·1% (1·8 to 6·0)
	Pakistan	132	2·7%	212 (184 to 250)	2·9% (2·6 to 3·5)	296 (237 to 383)	3·2% (2·6 to 4·2)	3·0% (2·2 to 4·0)
	Panama	1743	8·0%	3094 (2659 to 3563)	8·0% (6·9 to 9·2)	4569 (3750 to 5565)	8·1% (6·7 to 9·9)	3·6% (2·8 to 4·3)
	Papua New Guinea	108	4·4%	168 (139 to 206)	4·7% (3·9 to 5·7)	224 (167 to 304)	5·0% (3·8 to 6·8)	2·7% (1·6 to 3·8)
	Paraguay	863	9·8%	1374 (1146 to 1760)	10·8% (9·0 to 13·8)	1916 (1460 to 2827)	11·6% (8·9 to 17·1)	2·9% (1·9 to 4·4)
	Peru	626	5·2%	942 (807 to 1158)	5·3% (4·6 to 6·5)	1276 (1032 to 1692)	5·5% (4·5 to 7·3)	2·6% (1·9 to 3·7)
	Philippines	330	4·7%	559 (494 to 624)	5·2% (4·6 to 5·8)	787 (661 to 920)	5·5% (4·6 to 6·4)	3·2% (2·6 to 3·8)
	Poland	1629	6·3%	2836 (2528 to 3134)	5·9% (5·3 to 6·5)	4264 (3679 to 4873)	5·9% (5·1 to 6·7)	3·6% (3·0 to 4·1)
	Portugal	2697	9·3%	3774 (3110 to 4600)	9·8% (8·1 to 12·0)	4784 (3934 to 6355)	10·5% (8·7 to 14·0)	2·1% (1·4 to 3·2)
	Qatar	2663	2·2%	3785 (2922 to 5426)	2·7% (2·1 to 3·9)	5006 (3392 to 8591)	3·1% (2·1 to 5·3)	2·2% (0·9 to 4·3)
	Romania	1077	5·5%	2258 (1703 to 3063)	6·8% (5·1 to 9·2)	3500 (2608 to 4864)	7·7% (5·7 to 10·7)	4·3% (3·3 to 5·6)
	Russia	1877	7·1%	2287 (2100 to 2623)	7·5% (6·9 to 8·6)	2665 (2416 to 3206)	7·7% (7·0 to 9·3)	1·3% (0·9 to 2·0)
	Rwanda	158	9·4%	217 (165 to 289)	8·5% (6·4 to 11·3)	278 (188 to 448)	8·4% (5·6 to 13·4)	2·0% (0·6 to 3·9)
	Saint Lucia	755	6·7%	1023 (897 to 1212)	6·8% (6·0 to 8·1)	1340 (1086 to 1782)	7·4% (6·0 to 9·8)	2·1% (1·3 to 3·2)
	Saint Vincent and the Grenadines	917	8·8%	1203 (968 to 1545)	8·7% (7·0 to 11·2)	1506 (1106 to 2137)	9·2% (6·8 to 13·1)	1·8% (0·7 to 3·1)
	Samoa	365	7·2%	433 (338 to 643)	6·7% (5·2 to 9·9)	555 (403 to 856)	7·3% (5·3 to 11·2)	1·5% (0·4 to 3·2)
	São Tomé and Príncipe	251	7·9%	317 (241 to 416)	8·1% (6·2 to 10·6)	397 (262 to 608)	8·9% (5·9 to 13·7)	1·6% (0·2 to 3·3)
	Saudi Arabia	2320	4·4%	3355 (2554 to 5027)	5·3% (4·0 to 8·0)	4590 (3089 to 8043)	6·3% (4·2 to 11·1)	2·4% (1·1 to 4·6)
	Senegal	121	5·2%	153 (130 to 184)	5·3% (4·5 to 6·4)	182 (140 to 245)	5·7% (4·4 to 7·7)	1·5% (0·5 to 2·6)
	Serbia	1392	10·3%	1864 (1714 to 2037)	10·4% (9·6 to 11·4)	2319 (2113 to 2616)	10·7% (9·8 to 12·1)	1·9% (1·5 to 2·3)
	Seychelles	853	3·3%	1599 (1118 to 2226)	4·0% (2·8 to 5·5)	2498 (1355 to 3834)	4·5% (2·5 to 7·0)	3·8% (1·7 to 5·6)
	Sierra Leone	255	13·5%	250 (214 to 311)	15·7% (13·4 to 19·5)	290 (227 to 423)	15·9% (12·5 to 23·1)	0·4% (−0·4 to 1·9)
	Singapore	3981	4·8%	6990 (5335 to 9135)	6·0% (4·6 to 7·9)	10 035 (7204 to 14 611)	7·0% (5·0 to 10·2)	3·4% (2·2 to 4·8)
	Slovakia	2203	7·7%	3798 (3306 to 4375)	8·0% (7·0 to 9·2)	5354 (4571 to 6557)	8·2% (7·0 to 10·1)	3·3% (2·7 to 4·0)
	Slovenia	2845	9·1%	3970 (3482 to 4776)	9·4% (8·2 to 11·3)	4961 (4010 to 6494)	9·8% (7·9 to 12·8)	2·0% (1·3 to 3·1)
	Solomon Islands	107	5·8%	111 (75 to 157)	4·9% (3·3 to 7·0)	141 (82 to 230)	5·4% (3·2 to 8·8)	0·9% (−1·0 to 2·8)
	Somalia	33	6·9%	36 (27 to 50)	6·9% (5·2 to 9·5)	42 (27 to 72)	7·3% (4·7 to 12·4)	0·8% (−0·7 to 2·9)
	South Africa	1172	8·9%	1499 (1346 to 1684)	9·7% (8·7 to 10·9)	1815 (1555 to 2165)	10·3% (8·9 to 12·3)	1·6% (1·0 to 2·3)
	South Korea	2507	7·1%	4838 (4088 to 5783)	9·0% (7·6 to 10·8)	6859 (5323 to 8897)	10·1% (7·9 to 13·2)	3·7% (2·8 to 4·7)
	South Sudan	94	3·6%	120 (84 to 182)	5·1% (3·6 to 7·7)	145 (78 to 283)	6·4% (3·4 to 12·5)	1·4% (−0·7 to 4·1)
	Spain	3096	9·0%	4245 (3808 to 4645)	9·0% (8·0 to 9·8)	5194 (4510 to 5846)	9·1% (7·9 to 10·2)	1·9% (1·4 to 2·4)
	Sri Lanka	402	3·5%	911 (716 to 1180)	3·8% (3·0 to 5·0)	1645 (1207 to 2289)	4·3% (3·1 to 5·9)	5·2% (4·1 to 6·4)
	Sudan	334	8·3%	457 (380 to 543)	8·0% (6·6 to 9·5)	594 (478 to 730)	8·1% (6·5 to 9·9)	2·1% (1·3 to 2·9)
	Suriname	731	4·3%	940 (765 to 1171)	4·2% (3·4 to 5·2)	1195 (856 to 1630)	4·3% (3·1 to 5·9)	1·8% (0·6 to 3·0)
	Swaziland	745	9·5%	1132 (923 to 1430)	11·5% (9·4 to 14·5)	1467 (1062 to 2094)	12·8% (9·2 to 18·2)	2·4% (1·3 to 3·8)
	Sweden	5446	11·8%	8048 (6984 to 9231)	13·1% (11·4 to 15·0)	10 194 (8079 to 12 326)	13·9% (11·1 to 16·9)	2·3% (1·5 to 3·0)
	Switzerland	7831	12·8%	9702 (8612 to 10 687)	13·4% (11·9 to 14·7)	11 365 (9797 to 12 870)	14·0% (12·1 to 15·9)	1·4% (0·8 to 1·8)
	Syria	562	3·4%	736 (618 to 908)	3·7% (3·1 to 4·5)	926 (703 to 1274)	4·0% (3·0 to 5·5)	1·8% (0·8 to 3·0)
	Tajikistan	200	7·3%	309 (266 to 362)	8·9% (7·7 to 10·5)	398 (324 to 509)	9·8% (8·0 to 12·6)	2·5% (1·8 to 3·4)
	Tanzania	166	6·4%	239 (194 to 303)	6·2% (5·0 to 7·8)	308 (225 to 445)	6·4% (4·6 to 9·2)	2·2% (1·1 to 3·6)
	Thailand	633	4·1%	1113 (861 to 1390)	4·3% (3·4 to 5·4)	1689 (1315 to 2326)	4·7% (3·7 to 6·5)	3·6% (2·7 to 4·8)
	The Bahamas	1996	7·7%	2658 (2387 to 3054)	8·6% (7·7 to 9·8)	3306 (2792 to 4163)	9·1% (7·7 to 11·5)	1·8% (1·2 to 2·7)
	The Gambia	151	9·2%	174 (138 to 228)	10·2% (8·1 to 13·4)	199 (134 to 326)	11·4% (7·7 to 18·6)	0·9% (−0·4 to 2·8)
	Timor-Leste	105	1·9%	216 (139 to 329)	3·0% (2·0 to 4·6)	302 (155 to 532)	3·5% (1·8 to 6·1)	3·7% (1·5 to 6·0)
	Togo	81	5·5%	114 (99 to 134)	6·1% (5·2 to 7·1)	142 (113 to 187)	6·4% (5·1 to 8·4)	2·1% (1·2 to 3·1)
	Tonga	253	5·3%	399 (279 to 594)	6·4% (4·5 to 9·5)	553 (352 to 954)	7·6% (4·8 to 13·1)	2·8% (1·2 to 4·9)
	Trinidad and Tobago	1823	5·8%	2518 (2216 to 2919)	6·3% (5·5 to 7·3)	3177 (2671 to 4034)	6·5% (5·5 to 8·3)	2·0% (1·4 to 2·9)
	Tunisia	791	6·9%	1099 (992 to 1232)	7·2% (6·5 to 8·1)	1390 (1195 to 1653)	7·5% (6·4 to 8·9)	2·1% (1·5 to 2·7)
	Turkey	1040	5·3%	1748 (1556 to 2032)	5·7% (5·1 to 6·6)	2441 (2096 to 3065)	6·0% (5·1 to 7·5)	3·1% (2·6 to 4·0)
	Turkmenistan	396	2·3%	925 (763 to 1132)	2·7% (2·2 to 3·3)	1638 (1237 to 2191)	3·0% (2·3 to 4·1)	5·2% (4·2 to 6·3)
	Uganda	347	18·1%	313 (262 to 370)	11·6% (9·7 to 13·7)	384 (307 to 489)	11·6% (9·3 to 14·8)	0·3% (−0·5 to 1·3)
	Ukraine	659	7·0%	673 (584 to 781)	7·5% (6·5 to 8·7)	715 (557 to 899)	7·7% (6·0 to 9·7)	0·3% (−0·6 to 1·1)
	United Arab Emirates	2561	3·6%	3290 (2724 to 4287)	4·2% (3·4 to 5·4)	4182 (3227 to 6245)	4·6% (3·5 to 6·8)	1·8% (0·9 to 3·3)
	UK	3749	9·1%	5002 (4276 to 5803)	9·3% (7·9 to 10·8)	6169 (5056 to 7605)	9·6% (7·9 to 11·8)	1·8% (1·1 to 2·6)
	USA	9237	16·6%	12 448 (11 293 to 13 528)	17·7% (16·0 to 19·2)	15 026 (13 412 to 16 776)	18·5% (16·5 to 20·7)	1·8% (1·4 to 2·2)
	Uruguay	1837	8·6%	2766 (2289 to 3130)	8·9% (7·4 to 10·1)	3716 (2963 to 4400)	9·3% (7·4 to 11·1)	2·6% (1·8 to 3·2)
	Uzbekistan	397	5·9%	802 (648 to 1024)	7·2% (5·8 to 9·2)	1299 (931 to 1894)	8·3% (6·0 to 12·1)	4·3% (3·2 to 5·8)
	Vanuatu	149	5·4%	214 (145 to 331)	7·3% (5·0 to 11·3)	283 (162 to 524)	8·9% (5·1 to 16·5)	2·2% (0·3 to 4·7)
	Venezuela	1010	5·3%	1125 (988 to 1277)	5·7% (5·0 to 6·5)	1285 (1082 to 1528)	6·0% (5·1 to 7·2)	0·9% (0·3 to 1·5)
	Vietnam	398	7·0%	919 (740 to 1123)	7·6% (6·1 to 9·2)	1545 (1121 to 2038)	7·9% (5·8 to 10·5)	5·0% (3·8 to 6·0)
	Yemen	233	5·8%	229 (179 to 299)	7·0% (5·5 to 9·1)	276 (197 to 400)	7·4% (5·3 to 10·7)	0·6% (−0·6 to 2·0)
	Zambia	216	5·4%	287 (232 to 363)	5·6% (4·5 to 7·1)	345 (251 to 497)	5·7% (4·2 to 8·2)	1·7% (0·6 to 3·1)

Data in parentheses are uncertainty intervals. Data are 2015 purchasing power parity US$. GDP=gross domestic product. GBD=global burden of disease.

**Figure 2:**
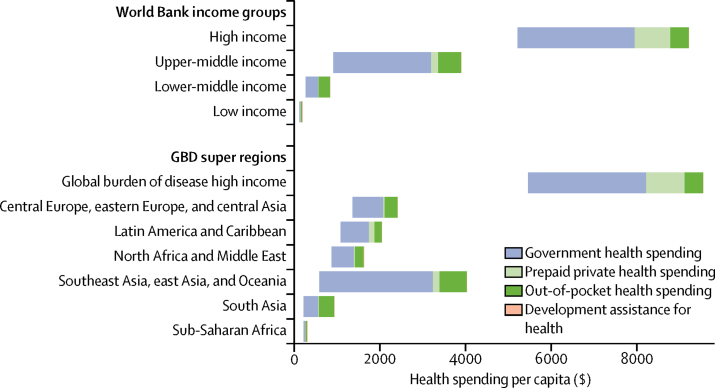
Increases in health spending by source, 2016 World Bank income group, and GBD super region in 2014–40 Per capita spending is measured in 2015 purchasing power parity US$. The left side of each bar marks the 2014 health spending for each group. The right side of the bar represents the expected 2040 health spending. The bar shows the expected increase in health spending between 2014 and 2040, and highlights the source of the spending growth. GBD=global burden of disease.

Figure 2 shows how per capita health spending is expected to increase between 2014 and 2040 in World Bank income groups and global burden of disease super regions. This growth is inflation and purchasing power adjusted. Health spending growth is highest in the groups that already spend the most on health. For example, high-income countries, which spent $5221 per capita in 2014, are expected to increase spending by $3994 (UI 3254–4746) between 2014 and 2040 and upper-middle-income countries, which spent $914 in 2014, are expected to increase per capita spending by $2989 (1856–4827). Meanwhile, lower-middle-income countries, which spent $267 per capita in 2014, are expected to increase spending by $577 (UI 472–737), and low-income countries, which spent $120 in 2014 are expected to increase spending by $75 (39–137). Sub-Saharan Africa is expected to increase spending from $218 per capita in 2014 by $89 (UI 51–147).

In terms of growth rates, the middle-income countries are expected to grow much faster than low-income and high-income country groups. Upper-middle-income countries are expected to grow the fastest of the income groups at 5·3% (UI 4·1–6·8), whereas lower-middle income countries are expected to grow only a little slower at 4·2% (3·8–4·9). A slower growth rate is expected in low-income countries 1·8% (UI 1·0–2·8) and in high-income countries at 2·1% (1·8–2·4**)**.

The growth in per capita health spending shown in figure 2 will largely be driven by increases in government health spending. Globally, government health spending per capita will increase by $1126 (UI 697–1763) between 2014 and 2040. Gains will be largest in high-income countries. The next largest increase in government spending is estimated to be in southeast Asia, eastern Asia, and Oceania; additionally major increases in per capita government spending are expected in China and Maldives. Out-of-pocket and prepaid private health financing are also expected to grow, although less than growth in government spending. In low-income and middle-income countries, development assistance for health per capita is expected to increase by only $3·2 (UI −4·0 to 19·5) globally between 2014 and 2040.

Underpinning these trends, tremendous variation in the levels of health spending exists. In 2014, health spending per capita ranged from $33 in Somalia to $9237 in the USA. In 2040, national spending is expected to span an even larger range: from $42 (UI $23–72) in Somalia to $15026 ($13 412–16 776) in the USA. We estimated that spending in countries that were considered low-income in 2016 would grow from $120 per capita in 2014 to $154 (UI 133–181) per capita in 2030, and $195 (157–258) per capita in 2040. For lower-middle-income countries, we expect 2030 per capita spending will grow from $267 to $525 (UI 485–582) and to $844 (738–1004) in 2040. Upper-middle-income countries are expected to increase per capita health spending from $914 to $2072 (UI 1698–2583) in 2030 and to $3903 (2770–5741) in 2040. Finally, we expect high-income countries to increase per capita spending from $5221 in 2014 to $7334 (UI 6786–7815) in 2030 and $9215 (8475–9967) in 2040.

Table 2 shows that the share of health spending financed by governments is expected to increase as well. This increase is true at the global level and for all World Bank 2016 income groups and all global burden of disease super regions. Government spending as a share of the total is expected to increase the most in upper-middle-income countries, whereas the share of government spending is expected to increase by only a little, from 59·2% in 2014 to 65·3% (UI 58·7–72·3) in 2040, although total health spending is expected to increase substantially. Globally, the share of health spending that is financed through out-of-pocket payments is expected to decrease from 22·8% in 2014 to 21·4% (UI 16·5–26·2) in 2040. This proportion is expected to drop in 164 of 184 countries included in this study.

**Table 2:** Expected health spending by source in 2040

	**2014**				**2040**			
	Government spending as share of total (%)	Prepaid private spending as share of total (%)	Out-of-pocket spending as share of total (%)	Development assistance for health as share of total (%)	Government spending as share of total (%)	Prepaid private spending as share of total (%)	Out-of-pocket spending as share of total (%)	Development assistance for health as share of total (%)
**Global**								
Total	59·2%	17·4%	22·8%	0·6%	65·3% (58·7–72·3)	12·9% (10·1–16·0)	21·4% (16·5–26·2)	0·4% (0·1–0·9)
**Income level**								
High income	63·4%	22·7%	13·9%	0%	65·5% (62·0–68·5)	22·0% (19·7–25·2)	12·5% (11·2–13·9)	0·0% (0·0–0·0)
Upper-middle income	57·2%	8·7%	33·8%	0·3%	71·2% (59·3–82·6)	6·4% (3·9–9·6)	22·3% (12·8–32·9)	0·0% (0·0–0·1)
Lower-middle income	35·9%	3·1%	58%	3%	45·6% (38·5–54·5)	2·7% (2·2–3·2)	50·5% (42·1–57·2)	1·2% (0·4–2·7)
Low income	18%	17·2%	29·1%	35·7%	29·4% (20·8–38·3)	14·4% (10·4–18·1)	29·9% (21·9–37·0)	26·3% (12·1–44·9)
**GBD super region**								
Central Europe, eastern Europe, and central Asia	58·5%	2·8%	38·5%	0·3%	62·5% (57·7–65·8)	3·2% (2·7–4·2)	34·1% (31·0–39·1)	0·2% (0·0–0·5)
Global Burden of Disease high income	62·8%	23·4%	13·8%	0%	64·8% (61·2–67·9)	22·8% (20·4–26·0)	12·5% (11·2–13·9)	0·0% (0·0–0·0)
Latin America and Caribbean	51·6%	16·1%	31·7%	0·7%	59·6% (52·1–67·4)	14·7% (11·3–19·1)	25·5% (20·3–30·9)	0·2% (0·1–0·5)
North Africa and Middle East	60·1%	4·3%	34·9%	0·7%	63·9% (58·6–70·5)	3·9% (3·1–4·8)	31·6% (25·7–36·6)	0·6% (0·2–1·4)
South Asia	31%	2·6%	64·7%	1·7%	43·5% (33·0–56·6)	2·1% (1·5–2·5)	54·0% (41·5–64·1)	0·4% (0·1–1·1)
Southeast Asia, East Asia, and Oceania	58·6%	5·2%	35·7%	0·5%	73·2% (58·8–85·7)	4·9% (2·7–8·1)	21·8% (11·0–35·1)	0·1% (0·0–0·2)
Sub-Saharan Africa	33·5%	20·8%	29·2%	16·6%	39·0% (32·0–45·4)	15·5% (12·7–18·0)	31·1% (25·6–36·3)	14·4% (5·9–27·3)
**Country**								
Afghanistan	15%	0%	54·1%	30·9%	19·1% (9·0–43·0)	0·5% (0·3–0·9)	50·2% (30·7–65·9)	30·1% (15·1–53·4)
Albania	48·3%	0%	49·8%	1·9%	58·1% (49·4–68·0)	0·8% (0·6–1·0)	41·0% (31·2–49·6)	0·1% (0·0–1·2)
Algeria	72·7%	0·7%	26·5%	0%	80·7% (72·4–89·0)	0·6% (0·3–1·0)	18·7% (10·6–26·7)	0·0% (0·0–0·1)
Andorra	78%	6%	15·9%	0%	78·5% (69·5–84·4)	6·5% (4·5–9·9)	15·0% (10·5–21·5)	0·0% (0·0–0·0)
Angola	70%	0%	26·6%	3·4%	61·9% (32·3–76·0)	2·3% (1·5–4·4)	32·7% (19·8–59·5)	3·1% (0·7–8·5)
Antigua and Barbuda	68·3%	8%	23·7%	0%	77·6% (68·9–85·4)	6·4% (4·1–9·4)	16·0% (10·1–23·2)	0·0% (0·0–0·0)
Argentina	55·8%	13·2%	30·9%	0%	65·0% (53·6–79·7)	11·3% (6·5–16·3)	23·7% (13·3–33·3)	0·0% (0·0–0·0)
Armenia	40·6%	3%	52·8%	3·6%	52·8% (40·1–71·7)	3·3% (1·9–5·3)	42·3% (25·0–54·7)	1·5% (0·0–6·0)
Australia	70·4%	9·9%	19·7%	0%	72·0% (66·7–76·7)	9·8% (7·8–12·7)	18·2% (14·8–23·3)	0·0% (0·0–0·0)
Austria	78%	5·8%	16·2%	0%	79·3% (76·0–82·6)	5·7% (4·6–8·1)	15·0% (12·4–17·6)	0·0% (0·0–0·0)
Azerbaijan	20·9%	4·3%	74·2%	0·6%	26·5% (18·1–39·1)	4·1% (3·0–5·8)	69·4% (57·2–77·9)	0·0% (0·0–0·0)
Bahrain	65·3%	10·6%	24·1%	0%	71·8% (64·1–81·2)	9·9% (6·3–14·3)	18·3% (12·1–24·1)	0·0% (0·0–0·0)
Bangladesh	22·7%	0%	65·6%	11·7%	30·2% (21·3–42·6)	1·8% (1·4–2·4)	63·6% (51·0–73·1)	4·5% (0·8–11·6)
Barbados	63·5%	6·6%	29·9%	0%	69·2% (60·0–77·1)	6·1% (4·3–8·8)	24·7% (17·7–33·3)	0·0% (0·0–0·0)
Belarus	66·9%	0·1%	32·6%	0·4%	68·2% (55·0–79·0)	0·8% (0·5–1·3)	31·0% (20·3–44·0)	0·0% (0·0–0·0)
Belgium	77·9%	4·3%	17·8%	0%	79·9% (76·6–83·6)	4·1% (3·2–5·2)	16·1% (12·9–18·8)	0·0% (0·0–0·0)
Belize	64·7%	9·5%	23%	2·9%	68·2% (61·2–74·8)	9·7% (7·3–13·1)	19·8% (15·2–24·7)	2·3% (0·5–5·7)
Benin	35%	0%	35·5%	29·6%	56·1% (39·3–73·7)	1·2% (0·6–2·0)	25·7% (15·6–36·3)	16·9% (6·0–34·4)
Bhutan	70·7%	0%	25·1%	4·2%	76·0% (58·4–88·1)	1·6% (0·8–2·7)	22·2% (10·7–39·0)	0·2% (0·0–1·9)
Bolivia	70·2%	3·4%	23·1%	3·3%	77·5% (70·1–84·2)	2·9% (1·8–4·6)	18·2% (12·4–24·6)	1·5% (0·4–3·6)
Bosnia and Herzegovina	70%	0%	28%	2%	78·8% (70·6–86·4)	0·5% (0·3–0·6)	20·4% (12·9–28·2)	0·4% (0·0–2·3)
Botswana	49·9%	35%	5·1%	10%	60·7% (49·2–72·1)	34·5% (24·2–45·2)	4·2% (2·9–5·7)	0·6% (0·0–7·0)
Brazil	45·9%	28·5%	25·5%	0·1%	56·1% (44·4–68·3)	24·8% (17·5–33·0)	19·1% (13·0–26·1)	0·0% (0·0–0·1)
Brunei	93·9%	0·1%	6%	0%	94·0% (89·4–97·0)	1·4% (0·8–2·1)	4·6% (2·1–8·9)	0·0% (0·0–0·0)
Bulgaria	54·7%	0·9%	44·3%	0·2%	61·1% (49·5–75·1)	0·7% (0·4–1·3)	38·3% (24·4–49·7)	0·0% (0·0–0·0)
Burkina Faso	35·8%	0%	38·6%	25·6%	40·5% (28·9–50·5)	1·1% (0·7–1·7)	38·1% (27·5–48·9)	20·3% (7·9–38·7)
Burundi	23·7%	0%	19·1%	57·2%	36·2% (17·2–55·4)	0·8% (0·4–1·4)	16·9% (8·9–26·8)	46·1% (23·0–71·0)
Cambodia	14·2%	0%	65·4%	20·4%	25·0% (15·1–34·8)	1·0% (0·7–1·5)	67·3% (56·9–77·7)	6·7% (1·6–16·5)
Cameroon	17%	3·5%	68·5%	10·9%	24·9% (16·1–37·7)	3·4% (2·5–4·8)	63·4% (51·0–73·8)	8·3% (2·9–17·8)
Canada	72·1%	14·1%	13·8%	0%	74·8% (71·5–79·1)	12·9% (10·7–14·8)	12·3% (9·6–14·7)	0·0% (0·0–0·0)
Cape Verde	58·4%	0·1%	22·2%	19·2%	68·4% (52·9–80·4)	1·2% (0·7–2·0)	21·9% (14·1–31·4)	8·6% (1·2–21·4)
Central African Republic	9%	0%	34·2%	56·7%	10·1% (2·9–20·1)	0·5% (0·1–1·0)	18·4% (5·8–36·2)	71·0% (44·8–90·7)
Chad	48·5%	1·3%	37·2%	12·9%	51·0% (20·7–71·8)	1·6% (0·8–2·8)	36·0% (20·2–60·4)	11·5% (3·3–26·6)
Chile	49·5%	19%	31·5%	0%	57·1% (45·8–67·0)	16·0% (12·1–20·7)	26·8% (20·1–34·5)	0·0% (0·0–0·0)
China	60·3%	5%	34·6%	0%	74·7% (59·1–87·5)	4·9% (2·6–8·3)	20·4% (9·3–34·8)	0·0% (0·0–0·0)
Colombia	71·9%	9·5%	15·3%	3·2%	77·8% (67·8–86·7)	10·4% (6·0–15·9)	11·7% (6·5–17·6)	0·1% (0·0–1·5)
Comoros	22·1%	20·1%	42·8%	14·9%	26·0% (14·2–43·2)	16·4% (11·4–22·1)	38·6% (25·3–52·3)	19·0% (6·6–39·8)
Congo (Brazzaville)	80·7%	0·3%	17·4%	1·7%	84·5% (77·2–89·6)	0·7% (0·5–1·1)	13·5% (8·9–20·5)	1·2% (0·4–3·1)
Costa Rica	73·1%	1·8%	25%	0%	73·5% (63·8–81·4)	1·8% (1·3–2·6)	24·6% (17·1–33·9)	0·0% (0·0–0·0)
Côte d'Ivoire	22·1%	8·2%	54·6%	15·1%	30·5% (22·4–40·6)	8·3% (6·2–10·9)	50·3% (41·2–58·7)	10·9% (3·7–22·9)
Croatia	81·9%	6·9%	11·2%	0%	81·9% (77·8–84·9)	7·5% (6·1–10·2)	10·7% (8·0–14·4)	0·0% (0·0–0·0)
Cuba	95·5%	0%	4·4%	0·2%	95·4% (93·1–97·1)	0·4% (0·3–0·6)	4·1% (2·6–6·3)	0·0% (0·0–0·2)
Cyprus	46%	4·4%	49·6%	0%	54·2% (45·6–64·5)	4·3% (3·0–6·2)	41·4% (32·0–49·4)	0·0% (0·0–0·0)
Czech Republic	84·8%	0·8%	14·4%	0%	85·1% (81·7–88·3)	0·9% (0·7–1·5)	14·0% (10·9–17·3)	0·0% (0·0–0·0)
DR Congo	21·3%	0%	37·4%	41·3%	33·6% (18·8–53·0)	1·0% (0·6–1·7)	35·5% (21·0–50·8)	29·9% (11·9–53·6)
Denmark	84·8%	1·9%	13·4%	0%	84·3% (80·1–87·5)	2·2% (1·7–3·3)	13·4% (10·5–17·1)	0·0% (0·0–0·0)
Djibouti	58·3%	0%	34·6%	7·1%	67·6% (54·9–81·4)	0·3% (0·2–0·5)	27·3% (15·7–38·4)	4·8% (1·4–11·4)
Dominica	68·7%	3%	28·3%	0%	73·0% (64·4–80·7)	2·7% (1·8–4·1)	24·3% (17·1–32·7)	0·0% (0·0–0·1)
Dominican Republic	63·4%	11·4%	21%	4·2%	74·0% (63·3–82·7)	11·1% (7·3–16·5)	14·9% (8·6–22·4)	0·0% (0·0–0·0)
Ecuador	48·8%	2·2%	48·5%	0·5%	53·1% (42·1–63·7)	2·0% (1·4–3·0)	44·6% (34·3–55·4)	0·3% (0·0–0·8)
Egypt	39·9%	1·5%	58·3%	0·2%	39·5% (33·5–49·5)	1·7% (1·2–2·5)	58·8% (49·0–64·7)	0·0% (0·0–0·2)
El Salvador	64·7%	4·9%	28·8%	1·6%	73·8% (63·5–84·5)	4·8% (2·7–7·6)	20·7% (11·8–29·8)	0·6% (0·0–2·1)
Equatorial Guinea	79·2%	0%	20·7%	0·1%	77·1% (65·6–84·4)	1·4% (1·0–1·9)	21·5% (14·5–33·1)	0·0% (0·0–0·0)
Eritrea	23·4%	0%	35·2%	41·4%	26·4% (12·4–42·3)	1·1% (0·7–1·7)	35·5% (21·5–50·9)	37·1% (16·6–61·2)
Estonia	79%	0·3%	20·8%	0%	82·1% (74·5–88·6)	0·6% (0·4–0·8)	17·3% (10·9–24·9)	0·0% (0·0–0·0)
Ethiopia	26·9%	0%	28·4%	44·7%	38·8% (24·2–53·2)	1·3% (0·7–2·0)	31·9% (19·6–43·9)	28·0% (9·6–52·3)
Federated States of Micronesia	0%	0%	7·7%	92·3%	8·3% (2·4–19·4)	0·3% (0·1–0·7)	7·9% (2·9–16·9)	83·5% (64·6–94·3)
Fiji	63·8%	7·5%	23%	5·7%	64·2% (57·0–69·8)	8·2% (6·4–10·7)	22·6% (18·3–29·5)	4·9% (1·2–12·0)
Finland	78%	3·1%	18·9%	0%	79·2% (76·5–82·0)	3·1% (2·6–4·0)	17·7% (15·0–20·2)	0·0% (0·0–0·0)
France	79·9%	13·6%	6·5%	0%	80·0% (76·2–83·5)	14·2% (11·4–17·8)	5·9% (4·5–7·1)	0·0% (0·0–0·0)
Gabon	67·4%	8·8%	22%	1·8%	81·0% (72·9–87·8)	5·3% (3·3–8·3)	13·3% (8·1–20·0)	0·4% (0·0–2·2)
Georgia	19·4%	18·9%	59·1%	2·6%	23·4% (15·5–36·1)	31·8% (20·8–40·9)	43·4% (33·4–54·3)	1·4% (0·0–4·9)
Germany	77·3%	9·4%	13·3%	0%	79·8% (75·8–83·2)	8·3% (6·8–10·0)	12·0% (9·7–14·6)	0·0% (0·0–0·0)
Ghana	52·8%	3·1%	27·1%	17%	61·1% (47·5–72·6)	3·1% (2·1–4·9)	25·6% (18·0–35·2)	10·2% (3·4–21·5)
Greece	61·7%	3·4%	34·9%	0%	63·4% (56·8–72·4)	3·9% (2·8–5·7)	32·7% (24·6–38·8)	0·0% (0·0–0·0)
Grenada	46·6%	2%	51·2%	0·2%	51·2% (42·8–62·1)	2·4% (1·8–3·0)	46·4% (35·8–54·6)	0·1% (0·0–0·3)
Guatemala	36·9%	8·2%	52·1%	2·8%	38·3% (31·3–45·5)	8·6% (7·2–10·8)	50·6% (43·8–57·1)	2·4% (0·7–5·7)
Guinea	20·4%	0%	34·5%	45·1%	40·2% (22·9–57·8)	1·0% (0·5–1·5)	31·2% (19·7–44·4)	27·6% (11·1–50·7)
Guinea-Bissau	6%	0%	52·1%	41·9%	3·2% (1·4–6·8)	0·9% (0·5–1·3)	48·1% (26·7–69·6)	47·8% (24·9–71·1)
Guyana	53·5%	2·9%	36·5%	7·1%	57·6% (47·4–67·7)	3·0% (2·1–4·3)	34·8% (26·2–44·2)	4·7% (1·2–11·5)
Haiti	0%	29·6%	29·6%	40·8%	1·1% (0·3–2·9)	34·4% (21·3–46·8)	27·6% (16·6–38·4)	37·0% (15·9–60·3)
Honduras	47·2%	5%	43·3%	4·6%	50·9% (44·3–60·8)	5·3% (3·7–7·5)	40·6% (32·2–46·9)	3·2% (0·6–7·8)
Hungary	68·1%	4·4%	27·5%	0%	68·6% (63·0–74·2)	4·1% (3·3–4·8)	27·3% (22·3–33·1)	0·0% (0·0–0·0)
Iceland	82·3%	0%	17·7%	0%	83·2% (79·7–86·8)	0·5% (0·4–0·6)	16·3% (12·7–19·8)	0·0% (0·0–0·0)
India	31·3%	2·4%	65·6%	0·7%	43·7% (32·5–57·5)	1·9% (1·4–2·4)	54·3% (41·0–65·2)	0·1% (0·0–0·4)
Indonesia	42·7%	2·7%	53·5%	1·1%	47·7% (37·3–58·8)	3·0% (2·0–4·4)	49·3% (38·5–59·4)	0·1% (0·0–0·6)
Iran	43·8%	5·3%	50·8%	0%	47·0% (30·6–61·0)	5·9% (3·9–9·1)	47·1% (34·1–62·8)	0·0% (0·0–0·0)
Iraq	58·2%	3%	38·4%	0·5%	62·1% (47·9–76·9)	3·3% (1·9–5·0)	34·4% (21·0–47·4)	0·2% (0·0–0·7)
Ireland	67·6%	14·3%	18·1%	0%	66·8% (53·3–76·2)	16·1% (11·2–23·6)	17·1% (11·8–24·5)	0·0% (0·0–0·0)
Israel	61·5%	11·2%	27·3%	0%	62·0% (53·7–69·4)	11·9% (9·1–15·7)	26·1% (20·7–32·4)	0·0% (0·0–0·0)
Italy	77·4%	0·9%	21·7%	0%	78·0% (72·0–83·1)	0·9% (0·8–1·1)	21·0% (16·0–27·2)	0·0% (0·0–0·0)
Jamaica	50·5%	19·4%	27·8%	2·3%	62·5% (50·8–74·1)	15·7% (10·4–21·9)	20·3% (13·9–27·1)	1·5% (0·2–4·3)
Japan	83·6%	2·4%	13·9%	0%	86·6% (82·9–89·5)	2·3% (1·7–3·6)	11·1% (8·5–14·2)	0·0% (0·0–0·0)
Jordan	66·8%	8%	21·1%	4·1%	67·4% (60·5–74·0)	9·2% (7·1–12·0)	20·2% (14·9–26·3)	3·2% (0·0–7·8)
Kazakhstan	54·4%	0%	45·3%	0·3%	55·3% (49·0–63·8)	1·2% (0·9–1·4)	43·5% (35·3–49·8)	0·0% (0·0–0·0)
Kenya	37·8%	3·8%	23·4%	35%	39·5% (25·5–58·4)	4·9% (3·2–6·6)	25·6% (16·1–35·2)	30·1% (12·4–51·7)
Kiribati	79·3%	0%	2·8%	17·9%	68·3% (22·4–90·8)	0·6% (0·3–1·8)	3·1% (1·3–8·9)	27·9% (6·7–71·0)
Kuwait	85·9%	1·3%	12·7%	0%	89·9% (83·0–95·2)	1·1% (0·6–1·7)	9·0% (4·1–15·6)	0·0% (0·0–0·0)
Kyrgyzstan	47·7%	1·3%	37·3%	13·7%	52·4% (40·5–64·3)	1·3% (0·9–2·0)	34·6% (25·2–44·6)	11·6% (3·9–24·7)
Laos	28·3%	0·4%	36·6%	34·7%	44·4% (25·5–65·7)	3·6% (2·0–6·3)	41·9% (23·9–61·4)	10·1% (0·0–32·5)
Latvia	63·2%	1·7%	35·1%	0%	63·4% (56·5–70·4)	2·0% (1·5–3·5)	34·6% (27·4–41·5)	0·0% (0·0–0·0)
Lebanon	47·6%	14·9%	36·4%	1·1%	50·5% (38·3–63·9)	16·2% (11·7–21·5)	33·1% (22·1–43·4)	0·2% (0·0–1·4)
Lesotho	63·4%	0·3%	15%	21·3%	70·7% (52·5–83·0)	0·5% (0·3–0·8)	12·6% (7·8–20·0)	16·3% (5·3–34·8)
Liberia	0%	0%	7·8%	92·2%	1·8% (0·2–6·5)	0·3% (0·2–0·4)	13·8% (9·8–17·8)	84·2% (78·5–89·1)
Libya	73·5%	0%	26·5%	0%	82·0% (65·1–91·6)	0·6% (0·3–1·0)	17·3% (8·0–34·2)	0·1% (0·0–0·4)
Lithuania	67·9%	0·8%	31·3%	0%	66·9% (60·2–73·8)	0·9% (0·6–1·3)	32·3% (25·5–38·8)	0·0% (0·0–0·0)
Luxembourg	83·9%	5·5%	10·6%	0%	85·3% (81·6–89·0)	5·5% (4·0–8·1)	9·2% (6·6–11·6)	0·0% (0·0–0·0)
Macedonia	63·1%	0%	36·6%	0·3%	62·1% (56·6–68·2)	0·7% (0·6–0·9)	37·2% (31·0–42·7)	0·0% (0·0–0·3)
Madagascar	29·5%	0%	34·3%	36·2%	39·0% (26·0–50·4)	1·3% (0·8–2·1)	30·5% (19·9–41·2)	29·2% (12·3–52·0)
Malawi	33·5%	14%	9·3%	43·1%	46·4% (30·2–60·7)	12·7% (8·0–17·8)	8·8% (5·6–12·7)	32·1% (14·2–54·8)
Malaysia	56%	8·1%	35·8%	0%	56·3% (48·5–67·3)	9·8% (6·9–13·5)	34·0% (24·4–41·4)	0·0% (0·0–0·0)
Maldives	79·4%	2%	18·5%	0%	78·1% (66·0–87·7)	2·3% (1·2–3·9)	19·6% (11·0–30·5)	0·0% (0·0–0·0)
Mali	22%	10·9%	43·6%	23·5%	36·8% (24·3–52·0)	9·6% (5·9–14·7)	38·3% (27·1–49·3)	15·2% (5·3–31·3)
Malta	69·2%	2%	28·9%	0%	78·0% (71·0–84·6)	1·7% (1·2–2·5)	20·4% (14·0–27·0)	0·0% (0·0–0·0)
Marshall Islands	62·9%	2·1%	11·8%	23·2%	63·1% (38·6–79·2)	2·6% (1·5–4·6)	13·0% (8·1–21·8)	21·3% (6·7–45·4)
Mauritania	44·5%	1·4%	44·7%	9·3%	53·3% (40·2–68·7)	1·5% (0·9–2·1)	38·6% (25·6–50·8)	6·7% (2·2–14·9)
Mauritius	50·8%	0·7%	48%	0·4%	65·7% (53·1–79·1)	0·9% (0·5–1·7)	33·3% (20·2–45·7)	0·0% (0·0–0·0)
Mexico	51·7%	4·2%	44%	0·1%	55·5% (45·3–63·6)	4·6% (3·4–6·5)	39·8% (32·2–49·9)	0·0% (0·0–0·1)
Moldova	47·2%	8·2%	38·3%	6·3%	47·2% (37·8–56·9)	9·4% (6·9–13·5)	38·9% (29·9–48·5)	4·5% (0·3–15·3)
Mongolia	51·4%	0·9%	41·9%	5·8%	57·4% (42·2–73·6)	1·2% (0·8–2·0)	41·2% (25·4–56·3)	0·2% (0·0–2·6)
Montenegro	55·3%	2·7%	41·4%	0·6%	62·2% (52·7–74·3)	2·4% (1·4–4·0)	35·4% (24·1–44·5)	0·0% (0·0–0·3)
Morocco	33·1%	7·6%	58·4%	0·9%	30·5% (24·9–35·3)	8·8% (7·2–11·3)	60·3% (55·4–65·7)	0·4% (0·0–1·2)
Mozambique	10·6%	0·6%	8·5%	80·2%	16·7% (4·9–37·3)	1·1% (0·5–2·1)	17·3% (8·1–31·1)	64·9% (38·9–84·6)
Myanmar	36·2%	0%	45·6%	18·2%	73·5% (51·8–90·7)	1·5% (0·5–2·9)	23·7% (8·3–44·0)	1·3% (0·0–8·3)
Namibia	53·5%	31·2%	6·9%	8·4%	61·8% (53·2–71·7)	29·2% (21·8–36·0)	6·2% (4·4–8·3)	2·8% (0·0–9·9)
Nepal	28·6%	5·9%	47·7%	17·8%	37·1% (29·3–46·4)	6·6% (4·7–9·7)	47·5% (37·6–55·3)	8·8% (1·5–21·3)
Netherlands	88·4%	6·3%	5·3%	0%	90·0% (87·1–92·2)	5·6% (4·3–7·8)	4·3% (3·1–6·4)	0·0% (0·0–0·0)
New Zealand	82·3%	6·6%	11%	0%	83·1% (78·9–86·4)	7·1% (5·4–9·5)	9·8% (7·5–12·4)	0·0% (0·0–0·0)
Nicaragua	50·9%	3·8%	37·3%	8%	54·9% (40·6–64·3)	4·1% (2·9–6·2)	35·9% (27·4–48·1)	5·1% (0·9–12·5)
Niger	26·3%	0%	49·5%	24·2%	39·8% (25·4–58·1)	0·8% (0·5–1·3)	45·4% (30·5–58·9)	14·0% (4·6–32·1)
Nigeria	22·1%	0·8%	70·1%	7%	24·7% (12·3–41·3)	1·5% (1·0–2·2)	67·1% (51·5–79·7)	6·8% (2·2–14·8)
Norway	83·1%	3·7%	13·2%	0%	86·8% (83·6–90·0)	3·1% (2·2–4·5)	10·1% (7·5–12·8)	0·0% (0·0–0·0)
Oman	91·8%	2·3%	5·9%	0%	93·6% (89·8–97·2)	1·9% (0·8–3·1)	4·5% (1·8–7·8)	0·0% (0·0–0·0)
Pakistan	32·1%	6·1%	55·4%	6·4%	47·6% (37·1–60·6)	5·4% (3·8–7·8)	42·9% (31·8–52·6)	4·1% (1·3–9·3)
Panama	72·5%	4·5%	22·3%	0·8%	75·0% (68·3–81·7)	5·4% (4·0–7·4)	19·6% (13·5–25·6)	0·0% (0·0–0·0)
Papua New Guinea	60·1%	3·9%	10·1%	25·9%	74·0% (59·3–84·6)	1·9% (1·3–2·6)	9·7% (6·2–14·5)	14·4% (4·9–29·8)
Paraguay	45·6%	4·6%	49·3%	0·5%	54·4% (42·3–70·2)	4·2% (2·6–6·1)	41·1% (26·7–52·7)	0·2% (0·0–0·7)
Peru	63·3%	6·3%	30%	0·4%	66·0% (57·3–75·5)	6·2% (4·4–9·1)	27·7% (19·4–35·8)	0·1% (0·0–0·5)
Philippines	33·6%	10·2%	54·3%	1·9%	43·1% (33·4–51·6)	10·3% (8·0–13·6)	45·7% (38·0–54·2)	0·9% (0·2–2·3)
Poland	71·4%	5%	23·6%	0%	72·4% (66·4–78·9)	6·0% (4·5–9·1)	21·6% (15·4–27·1)	0·0% (0·0–0·0)
Portugal	66·6%	5·9%	27·6%	0%	67·5% (59·1–77·1)	6·8% (4·5–9·6)	25·7% (17·6–34·1)	0·0% (0·0–0·0)
Qatar	85·7%	7·4%	6·9%	0%	89·1% (82·9–94·4)	6·1% (3·1–10·0)	4·8% (2·1–9·0)	0·0% (0·0–0·0)
Romania	79·1%	0·4%	18·9%	1·6%	86·6% (81·0–91·5)	0·7% (0·5–1·1)	12·7% (7·9–18·1)	0·0% (0·0–0·0)
Russia	51·8%	2·8%	45·5%	0%	53·2% (43·9–58·7)	3·0% (2·2–4·5)	43·9% (38·4–53·6)	0·0% (0·0–0·0)
Rwanda	0%	22·4%	22·6%	55%	1·7% (0·4–5·0)	28·1% (16·1–40·7)	27·3% (15·6–39·5)	42·9% (20·5–66·7)
Saint Lucia	49·2%	0·8%	45·6%	4·4%	58·9% (49·6–70·1)	0·9% (0·6–1·4)	37·6% (27·3–46·0)	2·6% (0·0–8·6)
Saint Vincent and the Grenadines	46·1%	2%	48·2%	3·6%	51·1% (37·0–67·1)	1·7% (1·0–3·3)	44·6% (29·6–58·1)	2·5% (0·0–7·6)
Samoa	87·2%	0%	5·9%	6·9%	84·9% (73·9–92·5)	0·7% (0·4–1·0)	5·8% (3·3–8·4)	8·5% (2·6–19·3)
São Tomé and Príncipe	31·1%	8%	11·9%	49%	47·3% (27·7–65·4)	5·9% (3·2–9·9)	10·6% (5·8–17·1)	36·2% (15·9–60·2)
Saudi Arabia	78·7%	6·2%	15·1%	0%	82·7% (74·4–91·2)	5·5% (2·7–8·7)	11·7% (5·8–18·7)	0·0% (0·0–0·0)
Senegal	39·4%	0%	33·8%	26·9%	46·8% (33·6–60·2)	1·1% (0·7–1·7)	31·9% (22·3–41·9)	20·3% (7·8–38·5)
Serbia	62·5%	0·3%	37%	0·1%	61·9% (55·4–66·5)	0·5% (0·4–0·8)	37·6% (33·0–44·1)	0·0% (0·0–0·3)
Seychelles	93·6%	4%	2·4%	0·1%	96·0% (92·4–97·9)	2·5% (1·3–5·3)	1·5% (0·6–3·1)	0·0% (0·0–0·0)
Sierra Leone	5·1%	9·2%	50·1%	35·6%	7·4% (4·0–12·0)	9·0% (5·6–12·3)	49·0% (32·7–63·0)	34·7% (22·0–55·1)
Singapore	42·4%	1·9%	55·7%	0%	56·2% (41·2–71·0)	1·7% (1·0–2·8)	42·1% (27·9–56·7)	0·0% (0·0–0·0)
Slovakia	76·3%	0%	23·7%	0%	77·5% (71·1–84·3)	0·6% (0·5–0·8)	21·9% (15·1–28·3)	0·0% (0·0–0·0)
Slovenia	73·2%	14·5%	12·3%	0%	73·1% (66·4–80·1)	15·6% (11·2–21·0)	11·3% (8·1–14·2)	0·0% (0·0–0·0)
Solomon Islands	67%	0%	4%	29·1%	55·9% (30·8–78·4)	1·0% (0·6–1·7)	4·5% (2·5–7·6)	38·5% (15·8–64·9)
Somalia	25%	1·2%	28·5%	45·2%	24·5% (11·6–38·0)	1·2% (0·6–2·0)	28·5% (15·5–42·2)	45·8% (22·4–70·7)
South Africa	47%	44·2%	6·4%	2·4%	53·8% (46·3–61·7)	38·7% (31·8–45·3)	5·2% (3·8–6·6)	2·4% (0·0–6·2)
South Korea	56%	6·6%	37·4%	0%	66·7% (57·0–75·7)	5·9% (4·0–8·9)	27·4% (19·6–35·8)	0·0% (0·0–0·0)
South Sudan	21%	0%	40·7%	38·3%	27·5% (9·1–58·3)	0·9% (0·4–1·5)	23·9% (11·0–40·1)	47·7% (20·0–75·4)
Spain	71·1%	4·8%	24·1%	0%	71·2% (64·4–76·8)	4·7% (3·9–6·2)	24·1% (18·4–31·0)	0·0% (0·0–0·0)
Sri Lanka	54·5%	1%	42·3%	2·1%	62·8% (50·6–74·2)	1·7% (1·0–2·7)	35·6% (24·6–47·1)	0·0% (0·0–0·0)
Sudan	20·4%	0·9%	76·6%	2·2%	22·9% (14·1–32·0)	1·0% (0·7–1·6)	74·5% (65·3–83·5)	1·6% (0·5–3·7)
Suriname	67·6%	15·5%	15·2%	1·7%	68·6% (56·5–78·3)	16·4% (11·1–23·5)	14·9% (9·3–22·2)	0·1% (0·0–1·7)
Swaziland	66·6%	8·4%	10%	15%	70·3% (55·0–82·3)	6·5% (4·2–9·6)	8·1% (5·1–12·2)	15·1% (5·3–31·1)
Sweden	85·1%	0·6%	14·2%	0%	86·8% (82·5–89·7)	0·6% (0·4–1·0)	12·6% (9·8–16·8)	0·0% (0·0–0·0)
Switzerland	60·3%	15·2%	24·5%	0%	66·2% (60·4–70·7)	10·3% (8·8–12·1)	23·5% (20·0–28·7)	0·0% (0·0–0·0)
Syria	44·5%	3·3%	51·6%	0·6%	52·3% (40·4–67·0)	3·3% (2·1–4·9)	43·8% (29·8–55·7)	0·6% (0·1–1·4)
Tajikistan	22·9%	8·7%	57·9%	10·6%	39·6% (29·1–53·3)	5·0% (2·9–9·9)	47·9% (36·7–57·7)	7·5% (2·4–16·6)
Tanzania	20·3%	17·1%	20·2%	42·4%	34·0% (20·7–53·1)	23·3% (14·9–32·0)	20·1% (12·6–28·5)	22·7% (8·5–43·0)
Thailand	78·7%	8·6%	12·1%	0·7%	82·1% (75·8–88·2)	8·8% (5·7–13·0)	9·1% (5·1–13·2)	0·0% (0·0–0·0)
The Bahamas	45·9%	24·9%	29·2%	0%	49·7% (41·1–60·6)	23·4% (17·8–29·5)	26·8% (19·9–35·3)	0·0% (0·0–0·0)
The Gambia	47·4%	0%	13·6%	39%	46·2% (26·2–64·0)	0·5% (0·3–0·9)	12·0% (6·7–18·3)	41·4% (19·7–66·3)
Timor-Leste	51·6%	0%	7·4%	41%	58·5% (27·0–82·7)	1·6% (0·8–2·9)	5·4% (2·2–11·5)	34·5% (11·7–66·4)
Togo	29·7%	7·8%	44·3%	18·3%	41·5% (29·9–55·9)	7·0% (5·1–9·2)	39·0% (28·5–48·5)	12·5% (4·4–26·2)
Tonga	69·5%	0·4%	11·7%	18·5%	75·7% (57·5–89·0)	0·8% (0·4–1·4)	8·2% (4·3–12·5)	15·3% (4·5–33·8)
Trinidad and Tobago	54·5%	6·8%	38·7%	0%	56·3% (46·9–66·8)	7·1% (5·0–9·7)	36·7% (27·1–46·5)	0·0% (0·0–0·0)
Tunisia	57·2%	4·5%	38·1%	0·2%	60·7% (53·6–67·4)	4·4% (3·5–6·1)	34·8% (28·6–41·6)	0·1% (0·0–0·3)
Turkey	78·4%	3·5%	18%	0·1%	79·5% (75·3–84·4)	3·0% (2·2–3·6)	17·5% (13·2–21·5)	0·0% (0·0–0·0)
Turkmenistan	59·2%	8·7%	31·6%	0·6%	67·6% (57·4–76·8)	6·1% (4·3–8·3)	26·2% (18·5–35·2)	0·0% (0·0–0·0)
Uganda	0·9%	64·8%	16·4%	18%	3·7% (1·3–8·0)	52·5% (40·1–62·3)	25·1% (18·7–31·4)	18·7% (7·2–35·8)
Ukraine	51·3%	0·9%	46·8%	0·9%	48·1% (34·8–56·8)	1·0% (0·7–1·7)	49·0% (40·4–62·3)	2·0% (0·2–7·0)
United Arab Emirates	72·3%	9·9%	17·8%	0%	75·7% (66·9–84·9)	9·0% (5·4–13·5)	15·3% (9·1–22·7)	0·0% (0·0–0·0)
UK	83·1%	7·1%	9·7%	0%	83·3% (79·3–86·9)	7·1% (5·6–8·9)	9·5% (7·3–12·6)	0·0% (0·0–0·0)
USA	49·8%	38·8%	11·4%	0%	51·9% (46·2–57·4)	37·8% (33·2–43·2)	10·2% (8·7–11·9)	0·0% (0·0–0·0)
Uruguay	71·2%	13·2%	15·6%	0%	74·2% (67·0–79·6)	12·7% (9·8–17·2)	13·1% (9·3–17·7)	0·0% (0·0–0·0)
Uzbekistan	51·9%	2·6%	43·7%	1·7%	69·3% (57·6–80·6)	1·6% (1·0–2·2)	28·5% (17·7–39·6)	0·6% (0·0–1·7)
Vanuatu	56·7%	0%	5·4%	37·9%	69·0% (43·2–88·4)	0·7% (0·3–1·3)	3·6% (1·7–6·0)	26·7% (8·2–53·2)
Venezuela	29·3%	6·3%	64·3%	0%	35·7% (26·0–46·0)	6·4% (4·8–8·8)	58·0% (48·4–67·0)	0·0% (0·0–0·0)
Vietnam	53%	6·9%	37·4%	2·7%	66·9% (54·5–77·9)	5·7% (4·1–7·8)	27·1% (17·0–38·5)	0·4% (0·0–1·8)
Yemen	14·3%	1·7%	74·7%	9·3%	13·5% (3·9–27·7)	1·4% (0·9–2·2)	67·8% (48·0–83·9)	17·3% (5·2–39·0)
Zambia	32·6%	0%	27·7%	39·7%	44·7% (29·2–59·8)	1·1% (0·7–1·7)	27·2% (17·8–37·6)	26·9% (10·6–49·2)

Data in parentheses are uncertainty intervals. Data are percentages. GBD=global burden of disease.

**Figure 3:**
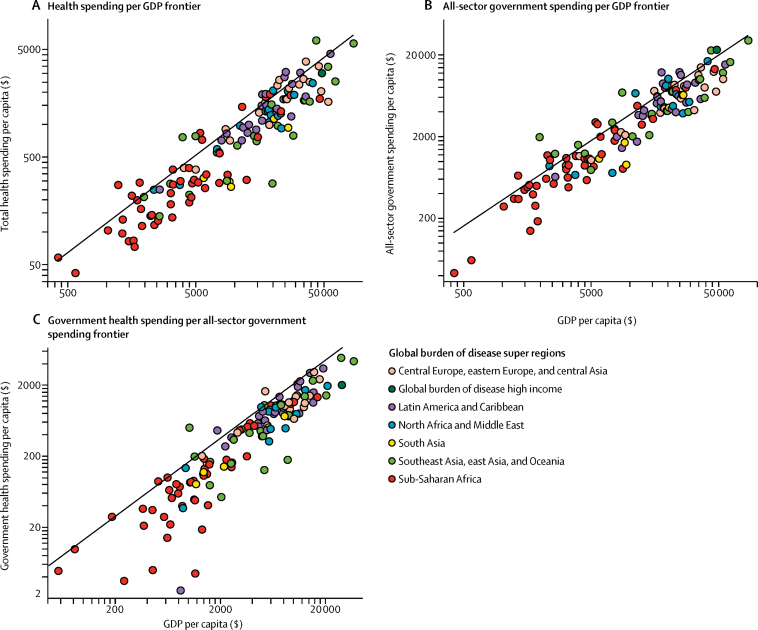
Expected health spending frontiers in 2040 Per capita spending is measured in 2015 PPP US$. The fitted lines are the estimated spending frontier. GDP=gross domestic product. PPP$=purchasing power parity US$.

Figure 3 shows the frontiers associated with potential total health spending, all-sector government spending, and government health spending. All three panels show an upward sloping frontier, meaning that more potential spending is associated with larger GDP per capita or all-sector government spending. The gap between the frontier and individual countries suggests that many countries might be able to divert more resources to health.

Table 3 (columns 2 and 3) provides country-specific estimates of the additional resources available if each low-income and middle-income country increased health spending to its predicted potential, as determined by GDP per capita and the frontier. The frontier analysis suggests that low-income countries as a whole could spend 64·3% (UI 13·0–115·1) more on health, across all sources, if all countries spent as much as their highest spending peers. Overall, lower-middle-income countries would spend 80·7% (UI 26·2–139·0) more and upper-middle-income countries would spend 19·9% (0·0–94·0) more, if all countries spent at the level marked by the frontier. Across regions, countries in sub-Saharan Africa would expand health spending the most, with an 82·8% (UI 35·7–121·1) increase, followed by south Asia (71·7%, 0·1–155·3) and north Africa and the Middle East (35·7%, 7·2–65·8).

**Table 3:** Potential total and government health spending for low-income and middle-income countries in 2040

	**Total health spending per capita in 2040 ($)**		**Government health spending per capita in 2040 ($)**			
	Expected	Potential	Expected	Potential government spending based on increasing all-sector government revenue	Potential government spending based on health sector prioritisation	Potential government spending based on increasing all-sector government revenue and health sector prioritisation
**Income level**						
Upper-middle income	3903 (2770–5741)	4638 (3130–6180)	2812 (1755–4635)	3643 (2055–7195)	5955 (4483–7746)	7406 (5043–10 275)
Lower-middle income	844 (739–1004)	1525 (1108–1942)	387 (289–545)	826 (440–1328)	913 (779–1061)	1837 (1131–2539)
Low income	195 (157–258)	321 (255–394)	57 (45–76)	121 (69–198)	161 (132–194)	285 (183–385)
**Region**						
Central Europe, eastern Europe, and central Asia	2417 (2252–2637)	2511 (2121–2999)	1511 (1377–1671)	1798 (1301–2389)	2370 (1874–2866)	3399 (2464–4373)
Latin America and Caribbean	2047 (1720–2494)	2297 (1964–2699)	1226 (915–1661)	1661 (1220–2224)	2298 (1760–2894)	2933 (2170–3695)
North Africa and Middle East	1630 (1431–1975)	1837 (1470–2153)	1045 (856–1387)	1261 (839–1821)	1753 (1495–2075)	2512 (1864–3192)
South Asia	935 (773–1203)	1599 (973–2244)	413 (260–673)	812 (317–1693)	1021 (829–1248)	1916 (1048–2985)
Southeast Asia, east Asia, and Oceania	4035 (2640–6314)	5080 (3064–7000)	2997 (1711–5223)	3980 (2023–8524)	6382 (4542–8659)	8255 (5310–11 921)
Sub-Saharan Africa	307 (269–365)	557 (435–656)	119 (102–142)	272 (151–444)	271 (222–335)	556 (356–737)
**Country**						
Afghanistan	249 (179–388)	272 (249–405)	49 (25–142)	58 (49–184)	213 (170–262)	237 (175–351)
Albania	1733 (1404–2144)	2292 (1733–3129)	1013 (732–1406)	1684 (1013–3152)	1809 (1280–2434)	2913 (1624–4449)
Algeria	2080 (1439–3337)	2114 (2080–3316)	1696 (1061–2954)	1752 (1696–3065)	2951 (1696–4187)	3005 (1858–4189)
Andorra	308 (154–414)	1190 (595–1893)	198 (56–291)	506 (198–1670)	745 (198–1822)	1379 (558–2439)
Argentina	3012 (2202–4807)	4020 (3012–6243)	1999 (1274–3799)	2144 (1999–4787)	6906 (4356–9293)	7202 (4808–10088)
Armenia	997 (727–1578)	1718 (1128–2367)	539 (337–1106)	981 (539–2283)	1203 (661–1926)	2057 (1177–3137)
Azerbaijan	2502 (2033–3062)	3308 (2502–4978)	671 (424–1141)	1433 (671–3408)	2263 (1357–3466)	4505 (2338–7650)
Bangladesh	266 (206–327)	919 (531–1330)	81 (53–128)	365 (122–780)	224 (160–308)	965 (429–1569)
Belarus	2369 (1648–3243)	2802 (2369–3845)	1634 (952–2496)	5473 (2214–10 802)	1649 (1634–2507)	5496 (2390–10 802)
Belize	844 (703–1017)	1212 (844–1645)	578 (443–747)	834 (578–1391)	965 (711–1264)	1361 (862–2049)
Benin	232 (161–357)	352 (232–477)	133 (75–250)	167 (133–349)	276 (163–448)	326 (195–476)
Bhutan	940 (517–1558)	2379 (1364–3313)	728 (320–1349)	1420 (728–3316)	1826 (810–3360)	3078 (1541–4702)
Bolivia	943 (736–1252)	1126 (943–1596)	734 (533–1038)	962 (734–1836)	983 (734–1360)	1266 (773–1961)
Bosnia and Herzegovina	2613 (1921–3416)	2613 (2613–3376)	2069 (1407–2847)	2362 (2069–4442)	2467 (2069–3005)	2782 (2069–4525)
Botswana	1878 (1452–2524)	2420 (1878–3756)	1152 (777–1780)	1677 (1152–3860)	2253 (1513–3184)	3145 (1923–5459)
Brazil	2770 (2150–3708)	2771 (2770–3774)	1572 (994–2509)	1644 (1572–2850)	3551 (2066–5144)	3660 (2306–5211)
Bulgaria	3870 (2896–5754)	3895 (3870–5805)	2412 (1516–4264)	2789 (2412–5934)	4102 (2762–5681)	4577 (3144–6558)
Burkina Faso	128 (101–168)	281 (178–393)	52 (37–62)	100 (52–189)	125 (70–202)	232 (121–367)
Burundi	104 (65–176)	127 (104–188)	36 (19–56)	53 (36–109)	64 (36–101)	90 (51–147)
Cambodia	642 (543–760)	1026 (642–1458)	162 (86–250)	367 (162–773)	522 (297–806)	1100 (551–1695)
Cameroon	190 (150–238)	469 (328–609)	48 (30–82)	98 (48–193)	218 (140–309)	428 (237–609)
Cape Verde	768 (523–1124)	1421 (902–1991)	532 (294–874)	1046 (532–2177)	895 (532–1461)	1642 (879–2554)
Central African Republic	58 (25–145)	64 (58–151)	5 (2–6)	21 (6–56)	9 (5–17)	34 (16–82)
Chad	138 (75–212)	358 (218–548)	74 (16–142)	183 (74–535)	155 (74–329)	321 (150–618)
China	5703 (3571–9218)	6658 (5703–9661)	4326 (2357–7776)	5429 (4326–12550)	9191 (6338–12763)	11233 (6662–16 657)
Colombia	2398 (1616–3727)	2555 (2398–3708)	1888 (1107–3215)	2573 (1888–5485)	2512 (1888–3379)	3316 (2108–5490)
Comoros	132 (96–184)	160 (132–214)	35 (19–65)	53 (35–121)	79 (45–126)	118 (67–176)
Congo (Brazzaville)	544 (394–736)	768 (544–1097)	461 (316–651)	658 (461–1397)	655 (461–1114)	871 (461–1433)
Costa Rica	3050 (2207–4077)	3087 (3050–4049)	2261 (1437–3273)	3393 (2261–6331)	2651 (2261–3468)	3885 (2338–6331)
Côte d'Ivoire	292 (246–352)	542 (367–736)	90 (63–132)	227 (100–449)	210 (130–316)	509 (274–773)
Cuba	3097 (2091–4454)	3113 (3097–4468)	2961 (1955–4323)	3063 (2961–4822)	3371 (2961–4284)	3470 (2961–4822)
DR Congo	83 (56–123)	176 (90–276)	28 (16–53)	42 (28–108)	106 (43–202)	141 (70–235)
Djibouti	842 (598–1324)	846 (842–1324)	579 (358–1057)	586 (579–1062)	811 (579–1119)	817 (579–1119)
Dominica	1092 (874–1406)	1533 (1092–2056)	802 (605–1106)	928 (802–1604)	1733 (1122–2473)	1945 (1283–2729)
Dominican Republic	1833 (1316–2498)	3071 (1877–4419)	1367 (861–2019)	3766 (1367–8015)	1651 (1367–2471)	4354 (2049–8015)
Ecuador	1935 (1534–2410)	1937 (1935–2403)	1035 (697–1466)	1416 (1035–2645)	1488 (1035–2078)	1966 (1231–2987)
Egypt	1212 (1070–1453)	1966 (1270–2607)	481 (384–708)	930 (481–1697)	1291 (821–1866)	2408 (1353–3493)
El Salvador	1520 (1089–2337)	1673 (1520–2292)	1136 (743–1953)	1136 (1136–1868)	3541 (2905–4166)	3541 (2905–4166)
Equatorial Guinea	1746 (1302–2291)	3905 (2057–7064)	1350 (927–1872)	2232 (1350–5465)	3964 (1545–7506)	5826 (2727–10 675)
Eritrea	84 (56–129)	190 (126–264)	22 (11–36)	30 (22–68)	121 (60–195)	152 (88–230)
Ethiopia	212 (153–311)	476 (241–843)	81 (59–113)	281 (85–691)	141 (81–262)	446 (164–913)
Federated States of Micronesia	767 (302–1703)	772 (767–1643)	53 (27–86)	58 (53–114)	404 (291–537)	431 (299–593)
Fiji	705 (630–804)	1399 (947–1901)	452 (401–525)	729 (452–1189)	1015 (712–1416)	1604 (965–2369)
Gabon	1336 (966–1900)	2075 (1344–2884)	1088 (723–1643)	1466 (1088–2956)	2099 (1220–3269)	2689 (1640–4001)
Georgia	1608 (1268–1972)	1694 (1608–2263)	380 (243–657)	718 (380–1709)	1069 (645–1673)	1893 (1042–2993)
Ghana	288 (214–381)	765 (451–1107)	178 (110–264)	330 (178–746)	465 (217–794)	783 (401–1279)
Grenada	1412 (1157–1755)	1928 (1412–2806)	729 (550–1051)	928 (729–1805)	1970 (1273–2837)	2447 (1548–3823)
Guatemala	715 (622–808)	1089 (734–1424)	275 (205–354)	747 (338–1306)	444 (323–607)	1182 (648–1702)
Guinea	165 (114–243)	214 (165–283)	66 (33–115)	101 (66–210)	119 (66–203)	170 (97–259)
Guinea-Bissau	115 (74–194)	221 (140–318)	3 (2–8)	18 (5–54)	42 (14–95)	173 (84–284)
Guyana	903 (733–1142)	1381 (931–1828)	523 (380–741)	877 (523–1625)	983 (598–1449)	1583 (925–2314)
Haiti	250 (178–385)	294 (250–410)	3 (1–7)	4 (3–14)	156 (120–199)	241 (141–362)
Honduras	716 (625–887)	800 (716–1074)	366 (302–529)	587 (366–1122)	516 (370–692)	805 (475–1243)
India	1138 (927–1488)	1822 (1138–2666)	505 (307–848)	959 (505–2112)	1234 (994–1518)	2226 (1135–3615)
Indonesia	793 (640–986)	2524 (1483–3592)	382 (252–565)	1216 (433–2529)	1078 (739–1491)	3259 (1561–5182)
Iran	2051 (1489–2709)	2341 (2051–3318)	980 (498–1554)	3011 (980–6876)	1125 (980–1669)	3319 (1516–6876)
Iraq	1230 (860–1897)	1740 (1230–2654)	783 (424–1444)	862 (783–1751)	2413 (1047–4132)	2598 (1409–4143)
Jamaica	1000 (748–1399)	1217 (1000–1759)	634 (386–1034)	813 (634–1713)	1130 (701–1617)	1416 (879–2165)
Jordan	1335 (1144–1565)	1632 (1335–2450)	901 (740–1118)	1426 (901–2576)	1266 (901–1893)	1930 (1152–3286)
Kazakhstan	2047 (1787–2500)	3972 (2466–5564)	1138 (896–1587)	3326 (1508–6221)	2010 (1296–2945)	5627 (2938–8574)
Kenya	286 (209–423)	490 (329–636)	112 (83–213)	195 (112–404)	266 (184–387)	452 (258–657)
Kyrgyzstan	384 (302–492)	514 (384–738)	202 (143–291)	409 (202–887)	262 (202–422)	502 (242–900)
Laos	285 (178–419)	1793 (908–2675)	127 (65–230)	267 (127–672)	1111 (705–1617)	2159 (1022–3587)
Lebanon	1895 (1458–2499)	2559 (1895–4858)	968 (603–1549)	2481 (968–6948)	1467 (968–2684)	3426 (1476–8244)
Lesotho	726 (464–1010)	729 (726–1003)	516 (294–757)	523 (516–773)	755 (523–987)	760 (527–987)
Liberia	276 (224–373)	277 (276–377)	5 (1–18)	8 (5–32)	81 (29–150)	112 (55–185)
Libya	979 (590–1637)	1142 (979–2158)	811 (441–1470)	868 (811–1962)	1875 (1256–2453)	1976 (1260–2949)
Macedonia	1742 (1549–1931)	2234 (1742–3016)	1080 (954–1211)	1895 (1080–2951)	1630 (1161–2328)	2803 (1652–4112)
Madagascar	73 (56–106)	193 (121–285)	28 (24–33)	144 (60–291)	32 (28–49)	160 (72–295)
Malawi	219 (160–320)	223 (219–327)	100 (77–135)	136 (100–260)	119 (100–181)	157 (100–262)
Malaysia	2528 (2099–3249)	5014 (3226–6840)	1428 (1126–2117)	2281 (1428–4204)	4856 (3393–6525)	7506 (4449–11 002)
Maldives	6070 (3725–9978)	6095 (6070–9722)	4823 (2464–8764)	4996 (4823–9589)	6896 (4823–9314)	7072 (4823–9811)
Mali	300 (231–402)	409 (300–554)	112 (67–191)	154 (112–321)	287 (167–426)	376 (203–560)
Marshall Islands	785 (448–1130)	788 (785–1111)	502 (228–729)	1293 (502–2853)	504 (502–729)	1294 (502–2853)
Mauritania	258 (193–366)	602 (378–820)	140 (89–244)	175 (140–376)	516 (271–801)	621 (360–885)
Mauritius	3459 (2435–5042)	4438 (3459–6149)	2308 (1365–3890)	4450 (2308–9867)	3498 (2714–4538)	6485 (3433–10 491)
Mexico	1726 (1403–2084)	2178 (1726–2889)	962 (672–1253)	1526 (962–2585)	1752 (1467–2084)	2714 (1695–3940)
Moldova	910 (755–1122)	928 (910–1220)	429 (332–579)	655 (429–1222)	596 (429–887)	881 (515–1432)
Mongolia	1685 (1177–2462)	2982 (1685–4412)	981 (566–1731)	1565 (981–3579)	2841 (1304–4932)	4136 (2112–6479)
Montenegro	2189 (1734–3138)	2394 (2189–3258)	1376 (942–2314)	1823 (1376–3803)	2348 (1879–2842)	3043 (2118–4445)
Morocco	1056 (945–1160)	1708 (1083–2472)	322 (248–382)	547 (322–968)	1241 (862–1692)	2050 (1140–3174)
Mozambique	117 (59–222)	267 (141–384)	18 (7–43)	20 (18–51)	257 (135–392)	274 (155–401)
Myanmar	979 (476–2210)	1987 (979–3099)	752 (269–2005)	1074 (752–3391)	1995 (1266–2630)	2597 (1400–4288)
Namibia	1929 (1590–2499)	1955 (1929–2563)	1200 (913–1752)	1308 (1200–2257)	2234 (1565–2902)	2392 (1635–3279)
Nepal	321 (263–388)	592 (383–840)	119 (93–160)	268 (120–517)	267 (152–438)	568 (295–914)
Nicaragua	830 (618–1005)	883 (830–1136)	459 (265–600)	1158 (495–2085)	468 (459–611)	1170 (532–2085)
Niger	98 (73–139)	159 (104–226)	40 (23–75)	40 (40–76)	162 (117–210)	163 (120–212)
Nigeria	343 (268–449)	877 (538–1202)	86 (36–177)	477 (113–1245)	204 (86–469)	921 (453–1441)
Pakistan	296 (237–383)	897 (579–1146)	142 (95–227)	330 (142–641)	429 (241–698)	934 (516–1324)
Panama	4569 (3750–5565)	4857 (4569–6570)	3432 (2676–4386)	5369 (3432–10 023)	4471 (3432–6044)	6806 (3664–10 906)
Papua New Guinea	224 (167–304)	465 (307–707)	166 (120–235)	236 (166–492)	312 (210–421)	432 (263–713)
Paraguay	1916 (1460–2827)	1916 (1916–2741)	1067 (665–1979)	1250 (1067–2870)	1696 (1067–2358)	1948 (1197–3121)
Peru	1276 (1032–1692)	2057 (1291–2807)	848 (635–1253)	2066 (880–3965)	1079 (848–1416)	2573 (1268–4033)
Philippines	787 (661–920)	1335 (866–1930)	340 (226–454)	979 (432–1864)	540 (401–705)	1514 (799–2396)
Romania	3500 (2608–4864)	3868 (3500–5144)	3041 (2162–4400)	4656 (3041–8158)	3655 (3041–5076)	5491 (3233–8320)
Russia	2665 (2416–3206)	3013 (2665–4031)	1413 (1281–1635)	1787 (1413–2841)	3268 (2198–4350)	4064 (2704–5923)
Rwanda	278 (188–448)	364 (278–545)	4 (1–13)	7 (4–25)	219 (130–345)	320 (177–518)
Saint Lucia	1340 (1086–1782)	1666 (1340–2423)	798 (574–1237)	821 (798–1404)	2537 (1790–3379)	2586 (1809–3419)
Saint Vincent and the Grenadines	1506 (1106–2137)	1568 (1506–2142)	784 (442–1393)	1038 (784–2218)	1397 (888–2027)	1805 (1133–2683)
Samoa	555 (403–856)	766 (555–1077)	474 (333–775)	616 (474–1243)	656 (474–966)	823 (495–1279)
São Tomé and Príncipe	397 (262–608)	474 (397–648)	184 (113–281)	328 (184–720)	279 (184–516)	456 (233–732)
Senegal	182 (140–245)	345 (227–456)	85 (63–127)	134 (85–255)	194 (139–266)	298 (169–446)
Serbia	2319 (2113–2616)	2320 (2319–2618)	1434 (1287–1608)	1542 (1434–2096)	2416 (1998–2834)	2587 (2120–3411)
Sierra Leone	290 (227–423)	291 (290–419)	21 (12–34)	62 (21–167)	65 (26–134)	165 (50–322)
Solomon Islands	141 (82–230)	291 (176–419)	78 (41–131)	81 (78–139)	310 (208–426)	317 (213–427)
Somalia	42 (27–72)	74 (43–107)	10 (6–13)	37 (11–69)	13 (10–18)	47 (19–80)
South Africa	1815 (1555–2165)	1821 (1815–2174)	981 (751–1315)	1289 (981–2192)	1489 (1204–1794)	1930 (1340–2737)
South Sudan	145 (78–283)	256 (182–336)	41 (15–113)	41 (41–114)	291 (172–545)	298 (179–545)
Sri Lanka	1645 (1207–2289)	3323 (1964–4684)	1048 (617–1693)	3100 (1056–6644)	1664 (1048–2575)	4574 (2237–7322)
Sudan	594 (478–730)	741 (594–1017)	136 (81–198)	653 (192–1409)	179 (136–309)	790 (353–1409)
Suriname	1195 (856–1630)	2430 (1635–3257)	828 (500–1253)	2099 (828–5089)	1463 (828–2989)	3231 (1788–5337)
Swaziland	1467 (1062–2094)	1467 (1467–2111)	1036 (699–1614)	1119 (1036–2006)	1321 (1036–1815)	1411 (1036–2056)
Syria	926 (703–1274)	2077 (1242–2971)	492 (327–814)	747 (492–1698)	1852 (1060–2902)	2639 (1434–4162)
Tajikistan	398 (324–509)	439 (398–594)	160 (102–264)	232 (160–482)	283 (168–429)	395 (223–603)
Tanzania	308 (225–445)	507 (308–714)	106 (62–213)	200 (106–462)	271 (147–455)	474 (237–743)
Thailand	1689 (1315–2326)	3086 (2004–4388)	1392 (1023–2031)	2080 (1392–4139)	2910 (2187–3741)	4190 (2608–6445)
The Gambia	199 (134–326)	212 (199–315)	88 (68–118)	156 (88–292)	99 (88–152)	171 (91–292)
Togo	142 (113–187)	252 (162–358)	60 (38–99)	91 (60–192)	147 (72–244)	208 (115–316)
Tonga	553 (352–954)	740 (553–1027)	424 (244–814)	474 (424–973)	788 (424–1186)	854 (528–1219)
Tunisia	1390 (1195–1653)	1713 (1390–2380)	847 (672–1085)	1427 (847–2530)	1239 (960–1539)	2034 (1223–3157)
Turkey	2441 (2096–3065)	3421 (2441–4850)	1946 (1613–2570)	2103 (1946–3513)	4936 (3814–6212)	5322 (3921–7459)
Turkmenistan	1638 (1237–2191)	4423 (2345–7144)	1117 (730–1668)	2674 (1117–6216)	2927 (1491–4977)	6439 (2886–11 326)
Uganda	384 (307–489)	392 (384–506)	14 (5–31)	41 (14–116)	113 (68–169)	309 (148–496)
Ukraine	715 (557–899)	910 (715–1376)	343 (232–413)	625 (343–1221)	547 (354–814)	954 (517–1642)
Uzbekistan	1299 (931–1894)	1448 (1299–2093)	912 (564–1496)	945 (912–1712)	2017 (1191–2802)	2091 (1303–2902)
Vanuatu	283 (162–524)	348 (283–546)	197 (106–426)	287 (197–749)	236 (197–430)	335 (197–749)
Venezuela	1285 (1082–1528)	1931 (1285–2976)	463 (295–677)	1000 (463–2250)	1197 (626–1994)	2376 (1162–4193)
Vietnam	1545 (1121–2038)	1794 (1545–2466)	1040 (660–1509)	2078 (1040–4032)	1166 (1040–1599)	2279 (1113–4032)
Yemen	276 (197–400)	404 (276–548)	37 (10–85)	85 (37–228)	166 (107–239)	357 (192–538)
Zambia	345 (251–497)	622 (377–820)	153 (108–224)	319 (153–618)	309 (179–519)	604 (316–877)

Data in parentheses are uncertainty intervals. Data are 2015 purchasing power parity US$.

**Figure 4:**
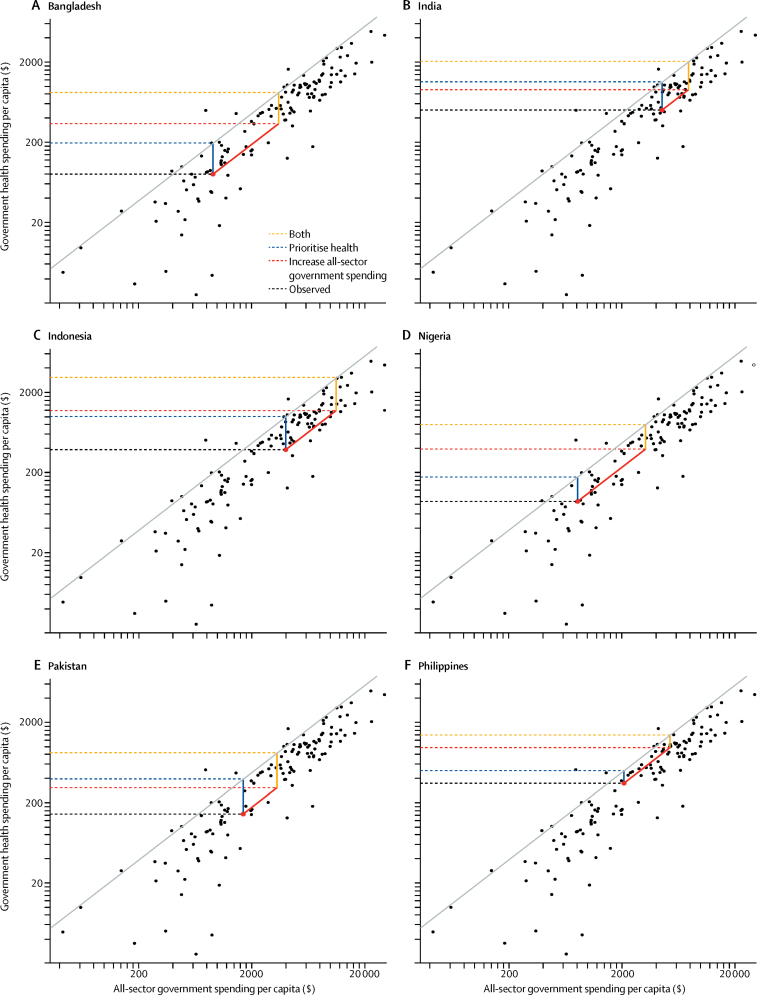
Potential government health spending of the six most populated low-income and lower-middle-income countries in 2040 Per capita spending is measured in 2015 PPP US$. The grey fitted lines are the estimated government health spending frontier. The short red lines parallel to the frontier represents the increases possible simply by raising more government spending. The vertical distance between the black and coloured lines represents potential increases in government health spending.

Figure 4 shows policy counterfactuals for the six most populous low-income and lower-middle-income countries and table 3 (columns 4 through 7) lists country-specific results. In this figure and table, the effects of three scenarios are shown: government spending increases (ie, increasing all-sector government spending to meet the frontier determined by GDP); reprioritisation of health in the government budget (ie, increasing government health spending to the frontier determined by all-sector government spending); and both government spending increases and reprioritisation of health in the government budget. In figure 4, the vertical distance between the three counterfactuals and the starting position (in black) illustrates the potential gains. The magnitude of the increases resulting from the policy changes vary depending on the country context. For instance, in Bangladesh, Nigeria, and Philippines, increases in all-sector government spending results in more government health spending rather than an increase in the share of the all-sector government budget that is devoted to health. By contrast, in India and Pakistan, increases in the share of the all-sector government budget that is devoted to health increases government health spending more than increases in all-sector government spending.

The implications of the policy changes for government health spending per capita vary across income groupings and region. For low-income countries, raising of government spending would increase government health spending per capita by $68 (UI 10–161), whereas reprioritisation of health would augment government health spending per capita by $107 (73–148). Government spending increases translate into $408 (UI 35–966) per capita in lower-middle-income countries and $569 (0–4585) per capita in upper-middle-income countries. Reprioritisation of health would, by contrast, augment government health spending per capita by $525 (UI 349–697) in lower-middle-income countries and $3145 (761–5392) in upper-middle-income countries. Overall, in low-income and middle-income countries, more government health spending per capita is increased by reprioritisation of health in the government budget, rather than the raising of more government resources, although table 3 shows enormous country level variation. By contrast, sub-Saharan Africa stands out; increases in the amount of government spending to the frontier would lead to more health resources rather than an increase in the allocation of government spending on health.

**Figure 5:**
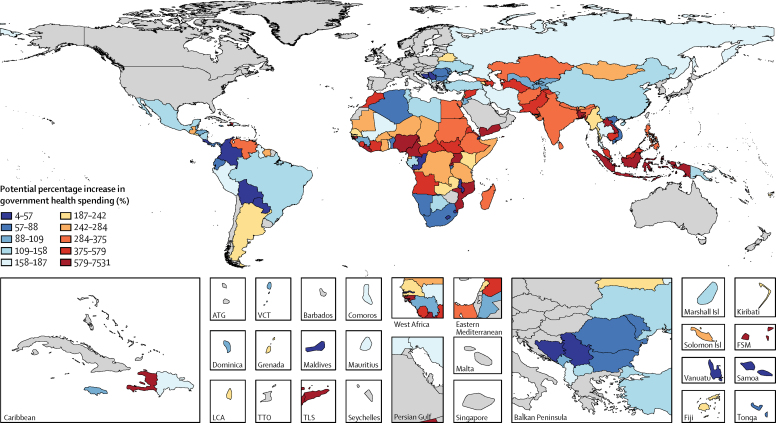
Potential increase in government health spending in 2040 The potential increase is the ratio of potential government health spending over expected government health spending, where potential spending is based on generating all-sector government spending and prioritising health sector at the level indicated by each frontier, based on each country's gross domestic product per capita in 2040. High-income countries and Zimbabwe are grey because we did not complete the potential spend counterfactual for these countries. ATG=Antigua and Barbuda. FSM=Federated States of Micronesia. LCA=Saint Lucia. TLS=Timor-Leste. TTO=Trinidad and Tobago. VCT=Saint Vincent and the Grenadines.

Figure 5 highlights the range of potential increases in government health spending as a proportion of GDP for low-income and middle-income countries. Countries with the highest potential increases are predominantly in Africa, the Middle East, and southeast Asia.

**Figure 6:**
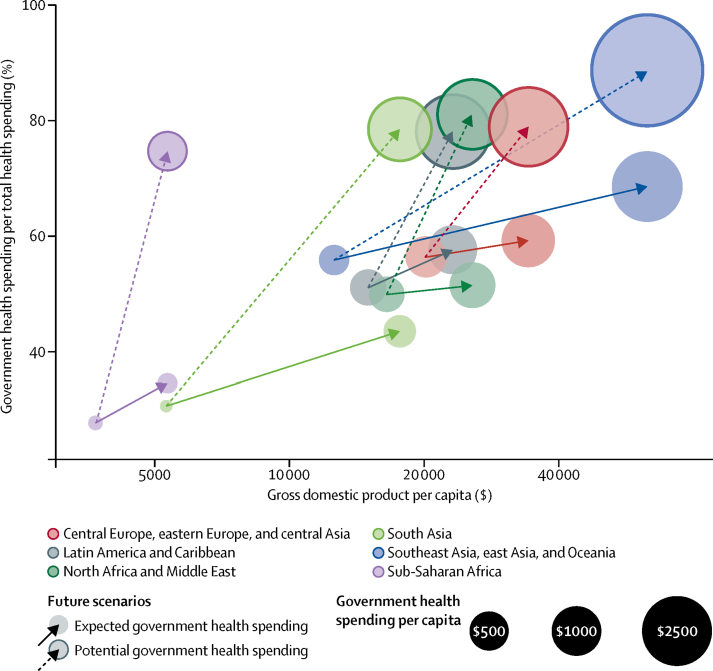
Government health financing expected and potential by global burden of disease super region in 2040 Per capita spending is measured in 2015 purchasing power parity US$. The size of the dot is scaled to reflect the amount of government health spending per capita. Each global burden of disease region has three bubbles. The bubble on the left marks the 2014 amount.

Figure 6 shows the difference between expected and potential government spending for each global burden of disease super region. The gain is shown in each region from the generation of more government spending and reprioritisation of the health sector, to the extent indicated by the frontier. In each region, these policy changes would lead to more government spending per capita on health and government health expenditure constituting a much higher share of total health expenditure. In per capita terms, southeast Asia, east Asia, and Oceania would increase government health spending per capita the most by reaching the potential levels. South Asia could potentially make the biggest increase in the share of total spending that is from the government.

## Discussion

With GDP, all-sector government spending, and health spending forecast estimates as well as frontier analysis, we assessed alternative scenarios illustrating potential increase in funds for health. We find that future, expected health spending varies substantially across countries. However, increases in total and government health spending are expected for nearly all countries.

This study highlights the multifaceted role economic development has in the trajectory of health financing. Economic growth leads to more spending across sectors as well as on health. Our analysis emphasises that in most countries, and especially China, expected economic growth is likely to catalyse more health spending.

Although economic growth is clearly a major driver of health spending, it is not deterministic. We find that a great deal of variation remains for countries at similar levels of economic development. The USA and the United Arab Emirates, for instance, which are expected to have similar GDP per capita in 2040, spend very different amounts on health. The USA is expected to spend 18·5% (UI 16·5–20·7) of GDP on health by 2040, whereas the United Arab Emirates is estimated to spend just 4·6% (3·5–6·8) by that time. This finding is true at lower levels of income as well: Namibia expected to spend 10·2% (UI 8·4–13·2) of GDP on health in 2040, and India, estimated to spend 5·6% (4·6–7·3) of GDP on health in 2040, are expected to have similar GDP per capita in 2040. Overall, our 2040 health spending per capita estimates range from $42 to $15 026, and from 1·4% of GDP to 22·2%.

Based on our potential spending estimates, most governments in low-income and middle-income countries could spend more on health in 2040 than in 2014 if they increased spending levels to reach the spending frontier, although the best strategy for increasing government spending depends on the country. In some countries, such as Bangladesh, Nigeria, and the Philippines, generation of more all-sector government spending would lead to the largest increase in government spending on health. For example, in 2014, government health spending per capita in Bangladesh was $21. In 2040, this figure is expected to be $81 (UI 53–128). This amount is nonetheless below the $86 per capita estimated in 2012 purchasing power parity dollars to be a minimum required to provide universal primary health-care services.^38^ However, we estimate that if the government could generate more all-sector government spending at the frontier, as determined by their level of development, there would be an additional $254 (UI 32–710) available per capita to spend towards meeting health sector goals. Mechanisms for increasing government spending revolve often around tax policy and the enforcement of tax policy. In other countries, such as India and Pakistan, reprioritisation of government spending towards the health sector is key. In Pakistan, for instance, government spending on health per capita was $42 in 2014. Government spending per capita is expected to increase to $142 (UI 95–227) in 2040. With reprioritisation of government spending on health, an additional $267 (UI 82–558) could be spent per capita on health. Implementation of both policy options would naturally lead to more spending on health.

However, the ability to reach spending frontiers and realise potential spending is more within reach for some countries than others. Contextual features such as national debt, corruption, or a substantive portion of the economy being informal (meaning it is not taxed or monitored by the government) might be distinct challenges for some countries, and might make reaching the spending frontier difficult. Prospective, country-specific, fiscal space analyses are needed to provide countries with tailored, country-specific capacity to spend assessments.

This study also shows that the differences in spending across countries, and across income groups in particular, are expected to persist. In some cases, in fact, we expect that without proactive policy changes, these differences will widen over time. Furthermore, our mean development assistance for health estimates suggest that development assistance for health will only marginally increase by 2040. The weak growth of health spending in the future in some countries is a result of the expected tepid growth in development assistance for health, and underlines the important part that development assistance for health continues to play in supporting health and health systems in low-income and middle-income countries.^14^ An important factor related to this difference is how health spending is distributed within each country. We report per capita health spending in this paper, which excludes within-country inequalities. As health spending in most countries is unequal, and most of the world's poor now live in middle-income countries, understanding how development assistance for health can target those most in need will be increasingly important.

The Sustainable Development Goals (SDGs) include a subgoal on health financing that aims to “substantially increase health financing…especially in least developed countries and small island developing States.”^39^ Additionally, the Addis Ababa Action Agenda of 2015 has already made the case for increasing domestic funding for health by re-envisioning such commitments as investment cases and introducing innovative financing mechanisms, including private funding and external support.^40^

In addition to planning for the SDGs, an understanding of future health spending is important in the context of the ongoing epidemiological transition occurring in many middle-income (and some low-income) countries. As countries' health burdens transition from infectious and childhood diseases to chronic, non-communicable diseases, different health system tools are needed. In some cases, these tools are expensive and require different health system infrastructure. As these health system demands are anticipated, health financing experts must develop plans rooted in medium-term and long-term forecasts, and consider a diverse set of policy options for raising the necessary resources.

An important point of caution is that this study does not assess the necessary amount of spending, and whether resources are spent efficiently and equitably. More spending does not necessarily guarantee better population health outcomes, and certainly does not secure more equitable distribution of health outcomes. Indeed, in most high-income countries, health policy spending targets revolve around the reduction of spending and strategies to increase efficiency. Health spending forecasts are valuable in high-income countries as a note of caution, because they reflect high levels of expected future spending based on past trends and relationships. However, our frontier analyses were designed to motivate governments and other development assistance partners to look at how countries compare, and to assess the strategies best suited to increase funding in low-income and middle-income countries. It is just as vital to understand how and where increased health spending is used, in addition to how it was mobilised. Improvement in the tracking of health resources is an important complement to our analysis of the future of global health financing.

Estimation of future spending is inherently uncertain. Health spending is a complicated product of national, international, and subnational policy decision making, institutional factors, the supply and demand of the health system, economic development, and even war, civil strife, natural disaster, and other environmental issues potentially related to climate change. Many of these factors are not forecasted through 2040. To make credible health spending estimates through 2040, we rely on past global and country specific spending trends and the relationships between these variables and economic development, government spending, and demographic variables. Because these methods and forecasting in general are far from exact, we quantified uncertainty by propagating UIs that increased the further we projected into the future. These intervals propagate data, model, and parameter uncertainty, and should be interpreted as plausible ranges, as established by past trends and relationships. Although the mean projection marks our point estimate, the large UIs reflect space for policy change and potential changes in spending as determined by key policy and decision makers.

In addition to these UIs, four limitations to this research should be noted. First, the underlying retrospective data include measurement error and imputation. Precise data that are comparable across time and across countries, and are complete for a long time period and all countries, are not available. The measurement of out-of-pocket spending and prepaid private spending is particularly challenging in countries without precise expenditure tracking or where informal payments (under the table or black market) make up a major share of health spending. This research relies on the best data available, although it is not without its flaws. When forecasting, measurement error is likely to be exacerbated, which is why we prefer wide UIs that mark a great deal of variation.

Second, expected health spending for 2040 is based on past trends and relationships. These projections are based on empirical relationships that are observed between 1995 and 2014, across 184 countries. We model relationships associated with country trends, economic development, fiscal shocks, and the demographic transition, but cannot anticipate policy or environmental changes that have not been observed in our retrospective data. This research does not address novel policies that skirt observed historic norms, potential environmental changes and challenges, and technological innovations in excess of the innovations observed during the last 20 years.

Third, stochastic frontier analyses are just one method to implement peer country comparisons. Other methods such as data envelope analysis or simple calculations of the mean, median, and various percentiles in spending ratios, constitute other potential approaches. We chose stochastic frontier analysis because this technique allows for data measurement error and considers a broader range of peer countries on which to base the frontier. Additionally, we used a half-normal distribution of residual for our stochastic frontier. Alternatives for this assumption exist as well. Robustness analyses presented in the appendix show that although country-specific results vary, the primary conclusions of this analysis persist across these modelling choices.

Fourth, it is crucial to understand that the frontier analysis presented in this research is based exclusively on one input. The frontiers used to identify potential national health spending and potential all-sector government spending are based on expected GDP per capita, whereas the frontier used to identify potential government health spending is based on expected all-sector government spending. These are long-term counterfactuals and do not consider the short-run realities more thoroughly explored in fiscal space analyses. Issues related to debt, government capacity, structure of the economy, demand, health system efficiency, disease burden, and population age structure are also excluded from in this analysis.

In conclusion, variation in GDP and health spending is expected to persist through 2040. Past trends and relationships suggest that health spending levels will continue to diverge globally and even within income groups. Increases in spending to reflect potential levels, as determined by GDP per capita and peer nations, would lead to more resources for health. However, the pathways to ensure these increases vary from country to country. This analysis can inform decision makers about possible methods to mobilise funds for health, given their country's level of development and financing environment. Despite expected increases in spending, this spending in some places will probably be insufficient to meet complex health needs, underlining the ongoing role of development assistance for health in some countries. Insights into spending trajectories and financing gaps are crucial as health stakeholders face the ambitious SDG agenda and the push towards universal health coverage.

Correspondence to: Dr Joseph L Dieleman, 2301 5th Avenue, Suite 600, Seattle, WA 98121, USA **dieleman@uw.edu**

**This online publication has been corrected. The corrected version first appeared at thelancet.com on May 18, 2017**
