# Department of Error

**DOI:** 10.1016/S0140-6736(17)31302-8

**Published:** 2017-05-20

*Global Burden of Disease Health Financing Collaborator Network. Future and potential spending on health 2015–40: development assistance for health, and government, prepaid private, and out-of-pocket health spending in 184 countries.* Lancet *2017; **389:** 2005–30* —The collaborators for this Article also include Lalit Dandona (Institute for Health Metrics and Evaluation, University of Washington, Seattle, WA, USA and Public Health Foundation of India, New Delhi, India), Rakhi Dandona (Public Health Foundation of India, New Delhi, India and Institute for Health Metrics and Evaluation, University of Washington, Seattle, WA, USA), and G Anil Kumar (Public Health Foundation of India, New Delhi, India). These corrections have been made to the online version as of May 18, 2017, and the printed Article is correct.

