# Supplementary material for: Future and potential spending on health 2015–40: development assistance for health, and government, prepaid private, and out-of-pocket health spending in 184 countries
Source: Lancet. 2017 May 20;389(10083):2005–30. doi: 10.1016/S0140-6736(17)30873-5 (PMC5440765; doi:10.1016/S0140-6736(17)30873-5)
Supplement: Supplementary appendix [file mmc1.pdf]

THE LANCET

## Supplementary appendix

This appendix formed part of the original submission and has been peer reviewed. We post it as supplied by the authors.

Supplement to: Global Burden of Disease Health Financing Collaborator Network. Future and potential spending on health 2015–40: development assistance for health, and government, prepaid private, and out-of-pocket health spending in 184 countries. *Lancet* 2017; published online April 19. http://dx.doi.org/10.1016/S0140-6736(17)30873-5.

SUPPLEMENTARY METHODS ANNEX

Future of Global Health Financing

Version: April 6, 2017

Global Burden of Disease Health Financing Collaborator Network – full author list in final pages of appendix

# Table of Contents

SECTION 1. INTRODUCTION ........ 3

SECTION 2. DATA ........ 4

Data sources ........ 4

Institute for Health Metrics and Evaluation's Development Assistance for Health Database ........ 4

World Health Organization's Global Health Observatory ........ 4

UN World Population Prospects ........ 5

WB, IMF, PWT, and Maddison ........ 5

Imputation of missing variables ........ 5

Descriptive statistics ........ 6

SECTION 3. STATISTICAL MODELS ........ 7

Ensemble modeling ........ 7

Universe of model specifications and ensembles ........ 7

Ensemble model selection ........ 10

Baseline model specification ........ 12

Inclusion criteria ........ 12

Estimation and inclusion statistics ........ 17

Uncertainty ........ 18

Additional tables ........ 20

FRONTIER ANALYSIS ........ 33

Approaches to frontier analysis ........ 33

Analyses ........ 35

Robustness analysis ........ 36

REFERENCES ........ 38

Country policy scenario figures ........ 43

# SECTION 1. INTRODUCTION

Information on future health spending is critical for planning and policymaking and ultimately for improving population health. Globally, tepid growth in development assistance for health, and locally, competing national priorities heighten the need and relevance of such forecast data to facilitate adequate planning to limit any losses in global health gains made in recent times.

The objective of this study is to provide data on future health spending patterns that can guide decision-makers. In order to achieve this objective, we conducted two types of analyses. First, we employed novel methods to generate expected health spending and potential health spending estimates. Second, we empirically assessed two avenues in government fiscal policy – increases in taxation and reprioritizing of the health sector in the government budget – through which potential government health spending could be adjusted to provide more resources to the health sector.

These analyses produced a comprehensive and comparable set of gross domestic product and all-sector government spending estimates which were used to examine prospective counterfactuals to illustrate expected and potential health spending in the years to come. Besides the baseline guidance that expected health spending estimates provide, the estimates on potential health spending provide alternative scenarios for decision-makers on what might be possible with policy adjustments.

The purpose of this appendix is to describe in detail the methodology used in our analyses. Subsequent sections contain information on all data sources, ensemble forecasting strategies, inclusion criteria, and uncertainty estimation used to generate our estimates as well as our guidelines on how our counterfactual analysis was conducted. Section 2 describes how the datasets used for the analyses were created. Section 3 details how the data were used in our analyses.

# SECTION 2. DATA

We used data from seen sources for the analyses: (i) WHO's Global Health Observatory (WHO), ii) Institute for Health Metrics and Evaluation's Development Assistance for Health Database (IHME), iii) International Monetary Fund (IMF) database, iv) Penn World Tables (PWT), v) World Bank World Development Indicators database (WB), vi) Maddison Project, and vii) United Nations World Population Prospects (WPP). Specifically, we collected health expenditure information on all available sources defined in Table 1 that is comparable across countries and complete for most countries from WHO and IHME, and demographic data from the WPP, while the underlying data for producing gross domestic product (GDP) and general government expenditure (GGE) were extracted from the IMF, WB, and PWT. Table 1 below presents the definitions for the various health expenditure sources.

## Data sources

Table 1. Definitions of health expenditure sources

| Health expenditure type | Definition |
|---|---|
| **Development assistance for health** | Financial and in-kind contributions from global health channels that aim to improve or maintain health in low- or middle-income countries. |
| **Government health expenditure as source** | Government health expenditure as source only includes domestically financed government expenditure on health. |
| **Out-of-pocket expenditure** | Paid by individuals for health services; considered catastrophic if exceeding 40% of a household's annual income. |
| **Prepaid private health expenditure** | Private risk pooling against catastrophic health expenditure; includes private insurance and non-governmental organizations. |

### Institute for Health Metrics and Evaluation's Development Assistance for Health Database

Development assistance for health estimates were obtained from the Institute for Health Metrics and Evaluation's Development Assistance for Health Database.[1] To generate these estimates, IHME collected audited budgets, annual reports, and project records from the primary development agencies providing assistance for the health sector. These records are augmented by information acquired via correspondence, and are standardized and compiled to provide a comprehensive perspective on international financial flows for health. These estimates are tracked backward to the source of the funds and forward to the country recipient, and are available through 2016 and 2014, respectively.

### World Health Organization's Global Health Observatory

WHO estimates health spending by source for 184 countries from 1995 to 2014. This database is updated annually and draws on publicly available documents from countries and international organizations such as National Health Accounts (NHAs), government ministry reports, and estimates from the World Bank and International Monetary Fund. NHAs are considered the gold standard and adhere to the guidelines set forth in *A System of Health Accounts*, 2011.

We adjusted these data by converting them from current local currency to fractions of GDP (both extracted from WHO), completed the series using multiple imputation, and converted them back into 2015 purchasing-power-parity (PPP$) terms. Multiple imputation is a common method used to impute missing data.[2]

## UN World Population Prospects

The UN World Population Prospects (WPP) provides population forecasts by age, sex, country, and year from 1950 until 2100. Using a cohort-component approach, WPP utilizes life tables to generate forecast age-specific mortality rates. Their modeling strategy involves a hierarchical Bayesian model of female life expectancy that prioritizes country data if available, but otherwise draws on regional data. A separate step models the male-female difference in life expectancy. From this data source, we generate multiple indicators of demographic context, such as the proportion of the population under the age of 20, and the dependency ratio (the ratio of working-age population [20 to 65 year olds] to the non-working-age population). We use the dependency ratio to measure the effect of the demographic dividend, a term used to signify a period where country fertility rates decrease due to declines in child mortality rates.

## WB, IMF, PWT, and Maddison

The World Bank World Development Indicators Database provides data on a wide range of development-related variables, including data on GDP and GDP per capita. Data series in this database begin in 1960. The IMF World Economic Outlook Database provides data on various macroeconomic indicators. Macroeconomic series data are available from 1980 to present. The Penn World Tables is a database that provides real national accounts data for 167 countries and territories. The data series starts in 1950. The Maddison Project database provides historical GDP, GDP per capita, and population data dating as far back as Roman times. We utilized GDP per capita as a primary covariate to produce forecasts. GDP per capita from 1950 through 2015 was constructed using the method described in James *et al.*[3] The method utilized extracted data from a number of sources (IMF, WB, PWT and Maddison), and used multiple random effects models to estimate a mean GDP per capita series to be used in our analysis. Similarly, we used the same methodology to produce a mean GGE per GDP series, from 1950 through 2015.

## Imputation of missing variables

We used the Amelia 1.7 package to impute missing values.[4] This procedure improves on mean imputation and single imputation and is specifically designed for cross-sectional longitudinal data such as our own.[4] Amelia has the capability to identify a panel data structure, and therefore executes the imputation in country- and year-specific dimensions. We converted the fractions to be imputed in logit space in order to ensure that the reverse transformation is between 0 and 1, and included them in three degrees of lags and leads each. Additionally, we included the log of GDP per capita as one of the supporting variables in the imputation process.

A complete panel of data for this study, 184 countries for 1995–2014, makes up 3,680 country-years of data. The missingness ranges from 1.8% in government health expenditure to 14.8% in prepaid private expenditure. Taking the dataset, D, which has observed and missing country-years, Amelia assumes that the variables are jointly multivariate normally distributed and the missingness in the data is random.

That is:

$$P(D \mid \mu, \Sigma) \sim MVN\ (D \mid \mu, \Sigma)$$

with the following likelihood function:

$$L(\mu,\Sigma \mid D) \sim \sum_{i=1}^{N} MVN(d(i) \mid \mu,\Sigma)$$

where *d(i)* is observation *i* in the dataset, μ is the mean, and Σ is the variance.

Amelia uses an expectation-maximization algorithmic approach in order to estimate the unknown parameters, μ and Σ. The predicted value for missing country-years is the mean of the imputed values across 100 datasets. The uncertainty is represented by the variation across the multiple imputations for each missing value. We use the full set of WHO data, IHME's DAH, GDP per capita, and GGE per capita data, linear country-specific time trends, and a ridge prior to inform our imputations.

## Descriptive statistics

Table 2 presents descriptive statistics for all the variables included in the analyses, while Table 3 has the Pearson's correlation values among the variables.

**Table 2. Summary statistics of variables included in the analyses**

| Variable | Mean | Median | Standard deviation | Minimum | Maximum |
|---|---|---|---|---|---|
| **GHES / GGE** | 0.129 | 0.121 | 0.063 | 0.001 | 0.405 |
| **OOP / GDP** | 0.020 | 0.018 | 0.012 | 0.001 | 0.094 |
| **PPP / GDP** | 0.004 | 0.002 | 0.008 | 0.001 | 0.117 |
| **DAH / GDP** | 0.007 | 0.001 | 0.015 | 0.001 | 0.362 |
| **GDP per capita** | 18,805.210 | 10,834.700 | 21,272.080 | 294.305 | 156,479.100 |
| **GGE / GDP** | 0.268 | 0.258 | 0.100 | 0.042 | 0.969 |
| **Pop** | 3.95 * 10^7 | 8.47 * 10^6 | 1.43 * 10^8 | 48,614.000 | 1.51 * 10^9 |
| **% Pop < 20** | 0.268 | 0.257 | 0.111 | 0.074 | 0.505 |
| **TFR** | 2.797 | 2.241 | 1.406 | 1.155 | 7.746 |

**Table 3. Correlation matrix of variables included in the analyses**

| | GHES / GGE | OOP / GDP | PPP / GDP | DAH / GDP | GDP per capita | GGE / GDP | Pop | % Pop < 20 | TFR |
|---|---|---|---|---|---|---|---|---|---|
| GHES / GGE | 1 | | | | | | | | |
| OOP / GDP | -0.1509 | 1 | | | | | | | |
| PPP / GDP | 0.1932 | 0.0342 | 1 | | | | | | |
| DAH / GDP | -0.2127 | 0.0589 | 0.0381 | 1 | | | | | |
| GDP per capita | 0.3931 | -0.2924 | 0.0736 | -0.2468 | 1 | | | | |
| GGE / GDP | 0.1817 | -0.2595 | -0.0776 | -0.0092 | 0.1911 | 1 | | | |
| Pop | -0.0531 | 0.0422 | 0.041 | -0.0626 | -0.0478 | -0.1182 | 1 | | |
| % Pop < 20 | -0.1088 | 0.0603 | 0.0166 | -0.0488 | -0.09 | -0.1564 | 0.9655 | 1 | |
| TFR | -0.4569 | 0.1296 | -0.031 | 0.3332 | -0.488 | -0.309 | -0.0832 | -0.0179 | 1 |

# SECTION 3. STATISTICAL MODEL

## Ensemble modeling

We used an ensemble modeling technique to create our forecasts, similar to our previous publication.[5] The strength of ensemble modeling is that our forecasts draw on multiple predictions derived from different specifications in order to create a stronger overall prediction, eliminating the need for a researcher to select one preferred model.[6–9]

This study capitalizes on past trends and relationships in health financing to forecast health expenditure by source for 184 countries, from 2014 through 2040, utilizing an advanced ensemble modeling approach.[5] We assessed 10,800 model variants, out of which a total of 2,833 models passed our inclusion criteria to be included in the ensembles. To begin with, we forecast the gross domestic product (GDP) of 188 countries and the general government expenditure (GGE) of 187 countries from 2016 to 2040. After that, we forecast each of the components of total health expenditure (GHE, PPP, OOP, DAH) and then aggregated each country's forecasts to generate total health expenditure from 2015 to 2040 for 184 countries. Four countries (Taiwan, Palestine, North Korea, and Zimbabwe) had to be excluded from the analyses due to inadequate data.

## Universe of model specifications and ensembles

After assembling the data, we developed a diverse set of plausible forecasting models. We assessed 10,800 model variants. These models included autoregressive terms, population, total fertility rate, other health financing variables, share of the population below 20, convergence terms, auto-correlated residuals and country-specific random intercepts. We converted all our data to use first differences in order to account for non-stationarity.

### *Dependent variables*

We forecast a sequence of dependent variables in this paper in the following order: GDP per capita, GGE per GDP, DAH per GDP, GHES per GGE, OOP per GDP, and PPP per GDP. The last four components in the list were aggregated to produce total health expenditures.

### *Autoregressive terms*

An autoregressive term is a lag of the dependent variable; for example, the $i^{th}$ lag of an outcome $Y_t$ is $Y_{t-i}$, and we explored models with zero to three lags.

### *Unique country trajectories*

We allowed for unique country trajectories by including country-specific random intercepts.

### *Convergence term*

We included the convergence term in our differenced models as the level of the dependent variable in the past time period, used to predict the forecast change in the dependent variable in the current time period. Convergence term was only used in forecasting GDP and GGE. Additionally, we had a prior belief on the coefficient of the convergence term (it must always be negative).

### *Up-weighting of recent years*

For each unique model in our ensemble, we allowed four other combinations of the same model, with the condition that the recent-most years of the in-sample data should have higher weights than the further past. We used a simple decay function with four decay parameters, and each of those up-weighting decay

functions are considered in the universe of ensembles. Weighting functions were not used for forecasting development assistance for health due to the very volatile and noisy nature of in-sample data.

### *Autoregressive residuals*

We also allowed for autocorrelations in our models by allowing the residual terms to have autoregressive processes of their own. This allowed us to evaluate models that could do a better job at explaining the in-sample secular trend. We tested up to three orders of auto-correlated models, and have only included this option in the GDP forecasts.

### *Health financing dependencies*

We also considered dependencies between the different types of health expenditures. It is well established that there is a relationship between GHES and DAH.[10] Moreover, it has been established that increased government health expenditure can reduce the amount of out-of-pocket expenditure.[11] We consider the lag of each other health spending source variable to affect each health spending source.

### *Summary of universe of model considered*

Figure 1 summarizes all combinations of models tested for the health expenditure variables. Green indicates a variable that was considered. Red indicates that we did not test a covariate due to prior beliefs about the health financing timeline. Black indicates that a certain combination was not possible to test. In addition to these covariates combinations, we also considered estimating parameters as fixed across all countries, random country-specific slopes, and country-specific coefficients by interacting the covariate with country dummies. In total, we considered 10,800 models. We did not have any prior beliefs on the coefficients when forecasting GDP and GGE.

Additionally, Table 4 displays the results of unit root tests on the covariates forecast in terms of p-values, and we see that none of our covariates have any unit roots at a statistically significant level. We analyzed the unit roots using four sets of unit root tests: the covariate-augmented Dickey-Fuller test (CADF), the Levin-Lin-Chu test (LLC), the Im-Pesaran-Shin test (IPS) and the Hadri Lagrange Multiplier stationarity test (Hadri LM). All four tests satisfied the conclusion of stationarity.[12–17]

**Figure 1. All combinations of covariates tested for health expenditure forecasting**

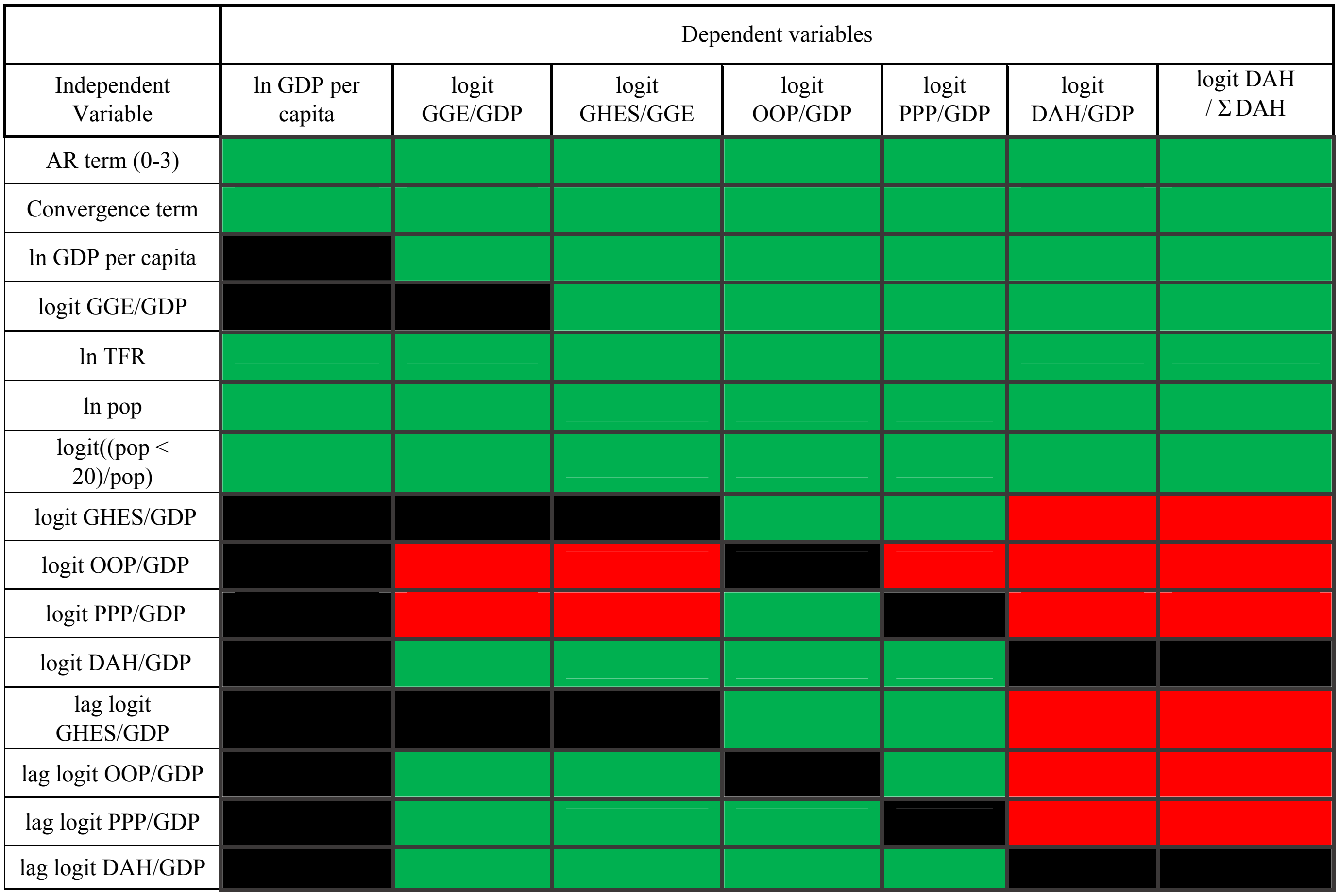


**Table 4. Unit root tests on the dependent variables**

| Unit root test | ln GDPpc | logit GGE/GDP | logit DAH / L2.GDP | logit DAH / Σ DAH | logit GHES / GDP | logit OOP / GDP | logit PPP / GDP |
|---|---|---|---|---|---|---|---|
| CADF | < 0.0001 | < 0.0001 | < 0.0001 | < 0.0001 | < 0.0001 | 0.0024 | < 0.0001 |
| LLC | 0.01373 | < 0.0001 | 0.0017 | < 0.0001 | < 0.0001 | < 0.0001 | < 0.0001 |
| IPS | < 0.0001 | < 0.0001 | < 0.0001 | < 0.0001 | < 0.0001 | 0.0005 | < 0.0001 |
| Hadri LM | < 0.0001 | < 0.0001 | < 0.0001 | < 0.0001 | < 0.0001 | < 0.0001 | < 0.0001 |

## Ensemble model selection

The steps we used in the selection of models for our ensemble forecasts are as follows:

1) We ran all possible combinations of model specifications using the in-sample data and produced forecast estimates at the mean level.

2) We applied a set of inclusion criteria to the estimated forecasts from the potential model, and recorded whether or not it passed all requirements. More information on these criteria are provided below.

3) Additionally, by leaving out the most recent years from our sample, we reran our models in order to assess their out-of-sample predictive validity (OOSPV). This allows us have a measure of which model had the best out-of-sample prediction for each country and out-of-sample year.

   The measure of OOSPV we used is country-year-model-specific root mean squared error (RMSE).

4) We reran the models that passed our inclusion criteria and applied four different types of uncertainty (outlined in section below) in order to generate approximately 10,000 draws of forecasts for each of our dependent variables.

5) For each year of in-sample data we left out in Step 3, we forecast the same number of years into the future:

   For example, when forecasting GDP series, we left out 10 years of in-sample data (2006 through 2015). When creating the forecasts for the year 2016, we assess the models that were run by leaving out the in-sample data for the year 2006. It should be noted that for all of the health expenditure variables, we use five years of in-sample data as opposed to 10 years for GDP and GGE. This is because our complete time series for the health expenditures go from 1995 through 2014, whereas GDP and GGE data exist for 1980 through 2015.

6) Following that, we forecast the draws for the year 2017 using the draws from the sub-models that were run by leaving out the in-sample data for the years 2006 and 2007, and so on. When forecasting the years 2025 through 2040, we assessed all of the models with out-of-sample predictive validity based on leaving out the years 2006 through 2015.

7) For each of the years in 2016 through 2024, we ranked each country's RMSE value (in ascending order) for that sub-model and year left out. The top 25% of the sub-models for each country were chosen to be included in the forecasts for a particular year. Therefore, for each country and year, we were able to collect 10,000 unique forecasts.

   From the years 2025 through 2040, we chose the same pool of sub-models in the ensemble, and so each of the countries had the same set of sub-models for those years.

8) Once we generated approximately 10,000 draws for each country and year, we needed a way to make sure that each draw across years for each country had some level of correlation, since our year-by-year forecasting method does not map sub-models to draws between the years forecast using single-year out-of-sample windows (i.e., 2016 through 2025 for GDP). We used the following strategy to achieve that:
   a. For each country, we recorded the Spearman's correlation coefficients for each draw across the years 2025 through 2040, and took the mean of all the correlations.

   b. We simulated a bivariate uniform distribution with the recorded mean correlation from step 8.a, using a Gaussian copula (which is imposed using Sklar's Theorem).[18,19]

   c. We recorded the ranks of the simulated distributions, and imposed them each year going backward from 2024 through 2016. Therefore, our complete time series data for all draws for each country and year will have the same rank correlation structure. The copula were simulated using R's VineCopula package.[20]

9) Finally, we computed the means and the 2.5$^{th}$ and 97.5$^{th}$ percentiles to generate our final ensemble forecasts.

*Development assistance for health modeling strategy*

We started from the belief that DAH is a supply-driven market. That is, DAH commitments are determined first by source countries on the "supply" side, not by recipient countries the "demand" side. With this in mind, we employed a unique three-step process for forecasting development assistance for health (DAH).

1. We extracted data and forecast the total amount of DAH provided between 2015 and 2040 using an ensemble model.

   Due to the anomalous level of growth in DAH disbursed in the decade of 2000–2010, we included a time dummy variable indicating whether the sample was part of that decade (and zero otherwise), with the prior belief of a positive coefficient on this indicator variable.

2. DAH received by each country, measured as a share of the total amount of DAH disbursed that year, was forecast using a second ensemble model and characteristics of the recipient. Potential covariates were the same as those described above.

3. We forecast transition of middle-income countries away from DAH as the transition to attaining high-income status. The transition was identified when the country's GDP per capita reached $18,108 per person. These countries were then excluded from that specific point onward for that model. We allow for uncertainty in graduation by performing this analysis over random GDP forecast draws. The amount $18,108 was determined by converting the World Bank's gross national income (GNI) of $12,736 into GDP. This conversion was made by regressing logged GDP per capita on logged GNI per capita for all countries in our dataset.

For the countries that did not transition to high-income status, the product of total DAH (predicted in the first step) and the share they would receive (predicted in the second step) provided an estimate of DAH received by a country for a given year, 2015 through 2040.

Let $DAH_{R,t}$ denote the DAH received before applying the graduation algorithm, and $DAH_{G,t}$ the DAH received after graduation. Therefore, for all recipient countries $i$, the new adjusted estimate of DAH received by a country for a given year between 2015 and 2040, inclusive, was given by:

$$DAH_{G,t} = \sum_i DAH_{R,i,t} \cdot \frac{DAH_{R,i,t}}{\sum_i DAH_{G,i,t}}$$

This three-part model helped us to ensure that *all* of the DAH supplied went to the countries demanding development assistance.

## Baseline model specification

Our baseline model specification was as follows. Using the example of GDP per capita as our dependent variable, the regression specification was of the form:

$$\Delta \ln GDP_{c,t} = \alpha_i + \sum_{i=1}^{3} \Delta \ln GDP_{c,t-i} + \gamma\Delta \ln POP_{c,t} + \zeta\Delta \text{ logit}\left(\frac{Pop < 20}{Pop}\right)_{c,t} + \eta\Delta \ln TFR_{c,t} + \epsilon_{c,t} + \sum_{i=1}^{3} \epsilon_{c,t-i}$$

where the error term $\epsilon_t$ was decomposed into two terms:

$$\delta_{c,t} = \left(\epsilon_{c,t} - \overline{\epsilon_t}\right)$$
$$\tau_t = \overline{\epsilon_t}$$

Each of the decomposed error terms is simulated as uncertainty (fundamental uncertainty) in either of two methods:

1) As respective random walk processes (only for GDP sub-models):

$$\tau_t \sim N(\widetilde{\tau_t}, [MAD(\tau_t - \tau_{t-1})]^2))$$
$$\delta_{c,t} \sim N(\widetilde{\delta_{c,t}}, \left[MAD\left(\delta_{c,t} - \delta_{c,t-1}\right)\right]^2)$$

   where the mean and variances of the normal distributions are the median and the squared median absolute deviations of each of the components respectively.

2) As a static random sample from a normal distribution with the respective mean and standard errors being the median and the median absolute deviation (MAD) of the observed country-specific residuals, respectively.

3) Furthermore, the standard errors of the fundamental uncertainty were capped using the MAD of the MADs across all models.

The details of simulating this vector of residuals are detailed in the section on fundamental uncertainty.

## Inclusion criteria

Our model selection consisted of three criteria:

1) Screening out models that estimated statistically insignificant relationships.
2) Screening out models that estimated relationships that contradict known relationships in health financing.

3) Screening out models that contradicted observed empirical norms in growth.

### *Statistically significant relationships*

We required that all estimated model parameters be statistically significant at the 10% level, with the exception of country-specific random intercepts. This meant that all included covariates must be statistically significant in order to pass this inclusion criterion. Insignificant parameters reduced the accuracy of our forecasts and added unnecessary uncertainty.

### *Expected direction on parameter estimates*

Table 5 details the relationships that we expect between different outcome variables and potential covariates. Models that did not produce the expected sign on the covariate were excluded from the ensemble model.

**Table 5. Covariates considered and expected relationships**

| | Dependent variable | | | | | | |
|---|---|---|---|---|---|---|---|
| **Independent variable** | ln GDPpc | logit GGE/GDP | logit GHES/GGE | logit OOP/GDP | logit PPP/GDP | logit DAH / GDP | logit DAH / Σ DAH |
| **AR term (1-6)** | No prior | No prior | No prior | No prior | No prior | No prior | No prior |
| **Convergence term** | - | - | - | - | - | - | - |
| **ln GDPpc** | Not possible | No prior | + | - | + | + | - |
| **logit GGE/GDP** | Not possible | No prior | No prior | No prior | No prior | No prior | No prior |
| **ln TFR** | No prior | No prior | No prior | No prior | No prior | No prior | No prior |
| **ln Pop** | No prior | No prior | No prior | No prior | No prior | No prior | + |
| **logit ((pop < 20)/pop)** | No prior | No prior | No prior | No prior | No prior | No prior | No prior |
| **logit GHES/GGE** | Not tested | Not tested | Not possible | No prior | Not tested | Not possible | Not possible |
| **logit OOP/GDP** | Not tested | Not tested | Not tested | Not possible | Not tested | Not possible | Not possible |
| **logit PPP/GDP** | Not tested | Not tested | Not tested | No prior | Not possible | Not possible | Not possible |
| **logit DAH/GDP** | Not tested | Not tested | - | Not tested | Not tested | Not possible | Not possible |

### *Empirically derived norms for allowed growth*

We excluded models that contradicted observed empirical norms in growth. To do this, we considered the observed percent growth in health expenditure (as a percent of GDP) in each year as a function of the level of health expenditure (as a percent of GDP) for all available data. The observed percent change of the fraction (health expenditure/GDP) is the right-hand side variable, and it is a function of how much a country is already allocating to health expenditure (the level of health expenditure/GDP).

The motivation behind this is demonstrated in Figures 2 through 7. Countries with low levels of health expenditure are more prone to large increases or decreases. As a country's health expenditure increases, the percent growth in the fraction attenuates as it becomes less likely that a country's health expenditure would change drastically year to year. This is observed in the pattern of the black dots in Figure 2.

**Figure 2. Observed percent growth as a function of government health expenditure quantity**

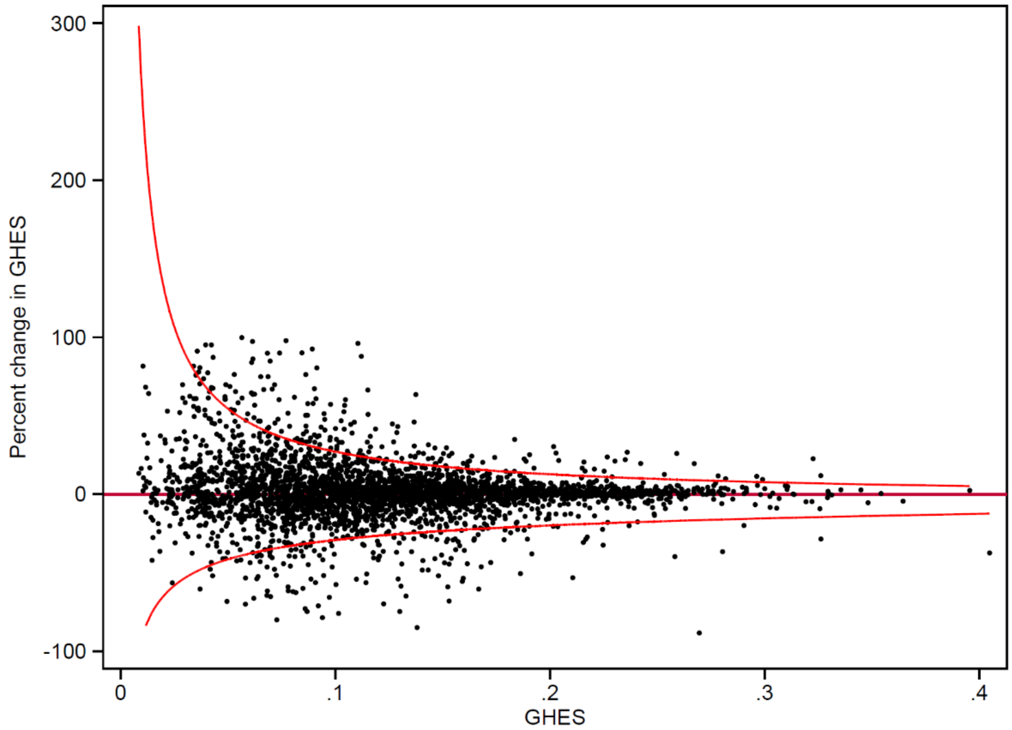


**Figure 3. Observed percent growth as a function of out-of-pocket expenditure quantity**

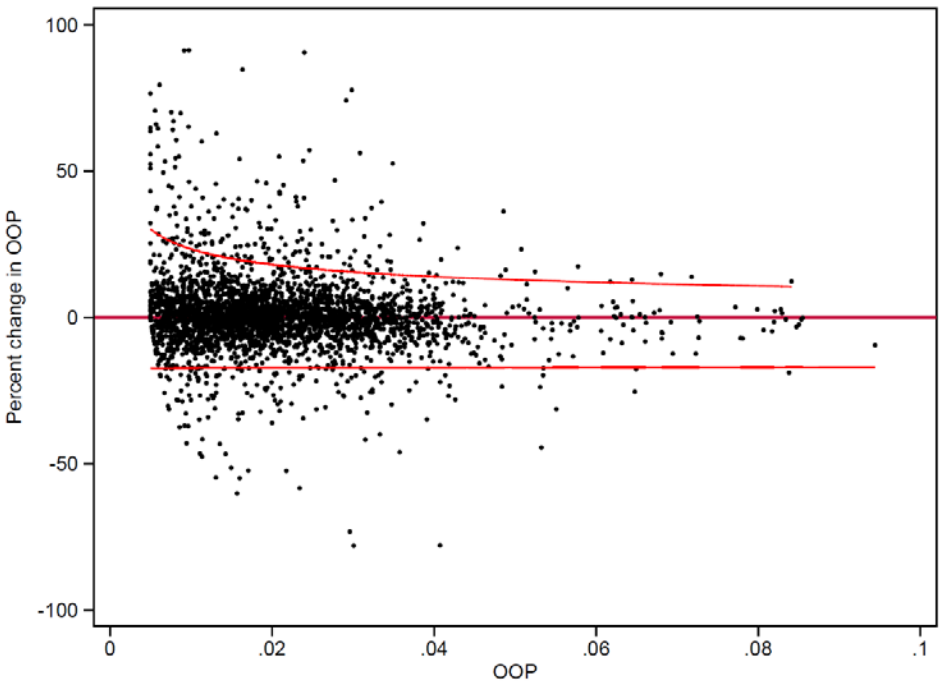

**Figure 4. Observed percent growth as a function of prepaid private expenditure quantity**

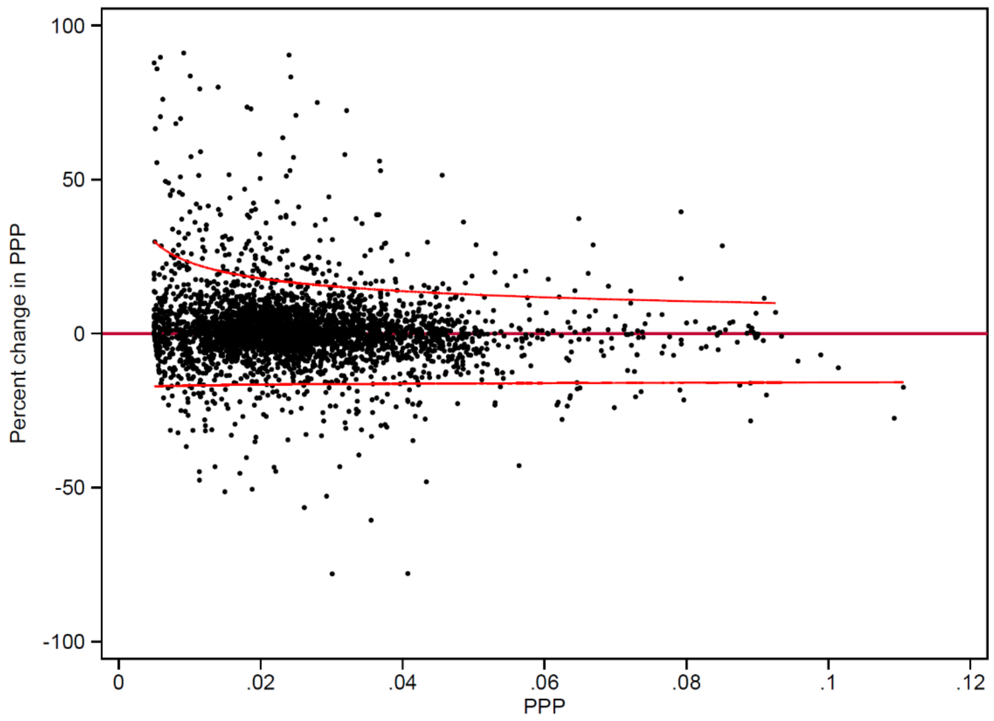


**Figure 5. Observed percent growth as a function of DAH disbursed**

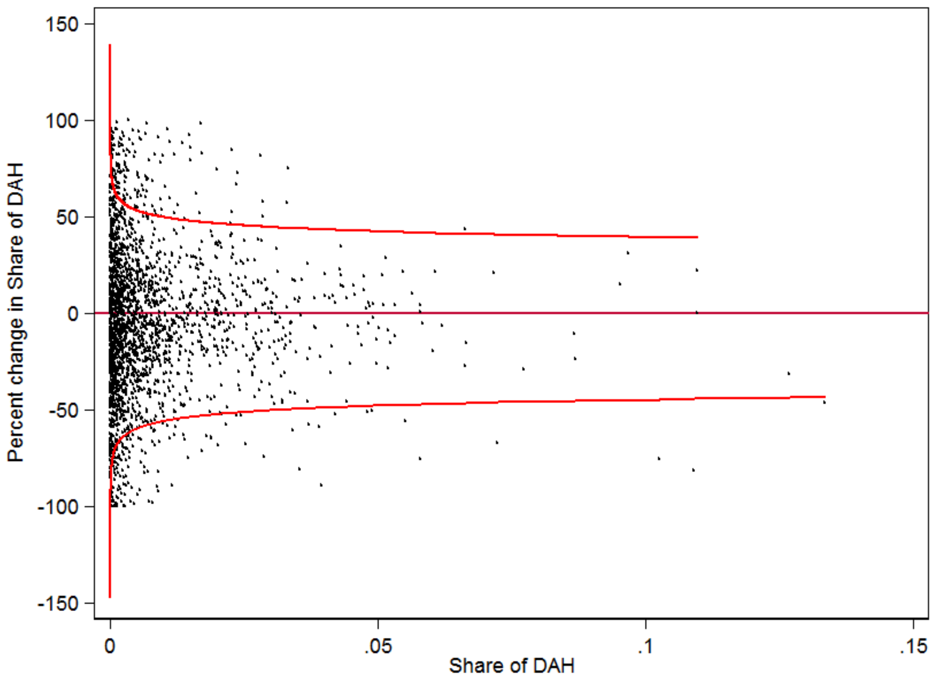

**Figure 6. Observed percent growth as a function of DAH received**

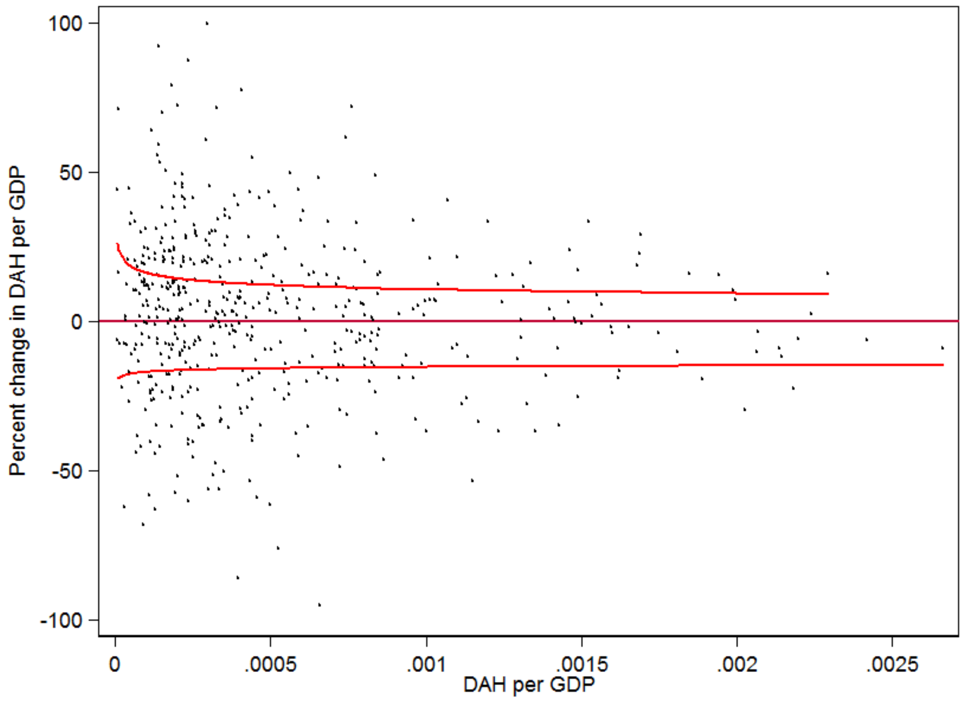


**Figure 7. Observed percent growth as a function of GGE per GDP**

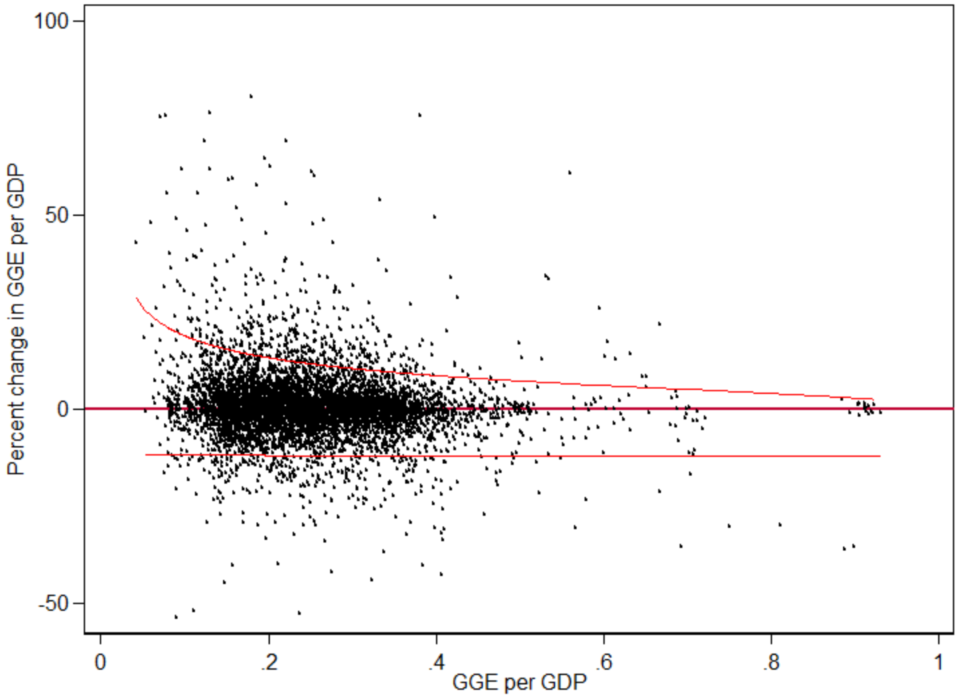

We used stochastic frontier analysis (SFA) as a method to estimate a boundary around a series of scattered points. In order to estimate an upper and lower bound for allowed percent growth, we first divided the observations into two categories: positive and negative percent change. For each category, we estimated a frontier of maximum percent change based on estimation of one-sided residual. Specifically, we implemented SFA with a half-normally distributed error term. The generalized version of a stochastic frontier analysis involves the maximum likelihood estimation of the following half normal stochastic frontier model:[21]

$$y_{it} = \alpha + x_{it}'\beta + \epsilon_{it}$$

$$\epsilon_{it} = v_{it} - u_{it}$$

$$v_{it} \sim \mathcal{N}(0, \sigma_v^2)$$

$$u_i \sim \mathcal{N}^+(0, \sigma_u^2)$$

This gave us an upper bound that is the greatest value observed in the data, allowing for some noise and measurement error. The data showed us that countries with greater spending per GDP have smaller percent changes, so we get a convex, decreasing boundary. That means, intuitively, countries with very little spending per GDP may have more volatility, but for countries already spending a lot on health, potential growth is more constrained. Most important, this reflected the data.

## Estimation and inclusion statistics

This analysis was executed on a high-performance 20,000-node computing cluster with a CentOS interface. All of the codes are written in Stata 13.1; R 3.3.2; and Linux bash scripts.[4,22–28] We assessed a total of 10,800 model variants. The number of models that pass our inclusion criteria for each variable are included in the table below, Table 6.

**Table 6. Number of models included in forecasts**

| | Gross domestic product | General government expenditure | Government health spending | Out-of-pocket health spending | Private pre-paid health spending | Development assistance for health | |
|---|---|---|---|---|---|---|---|
| | | | | | | Disbursed | Received |
| **Number of models tested** | 1,664 | 128 | 1,720 | 3,838 | 2,814 | 381 | 255 |
| **Number of models that passed exclusion criteria and are included in the ensemble** | 767 | 46 | 588 | 953 | 445 | 20 | 14 |

Table 7 reports the mean parameter estimate for each covariate, calculated from the models that pass our inclusion criteria. All parameter estimates are statistically significant at the 10% level, by virtue of the inclusion criteria. Parameters with values of zero indicates that sub-models containing those covariates were not included to forecast the respective dependent variable.

**Table 7. Mean parameter estimates of covariates included in the ensemble forecast by dependent variable**

| | GDP per cap | GGE per GDP | GHE-S | OOP | PPP | DAH disbursed | DAH received |
|---|---|---|---|---|---|---|---|
| **GDP per capita** | - | 0.52864 | 0.00019 | -0.23596 | 0.00776 | 1.62373 | -0.3512 |
| **GGE per GDP** | - | - | -0.14373 | 0.05175 | 0.02934 | -0.55923 | 0.00000 |
| **TFR** | 0.15999 | -0.08471 | 0.40304 | -0.06131 | 0.12031 | 2.63185 | -2.48478 |
| **% Pop < 20** | -0.03578 | 0.10104 | 0.54790 | 0.04684 | 0.19031 | -3.37962 | 0.34928 |
| **Pop** | 0.11532 | -0.15885 | 0.00000 | 0.00000 | 0.00000 | 10.48206 | 0.14264 |
| **GHE-S** | - | - | - | 0.00634 | -0.02993 | - | - |
| **OOP** | - | - | 0.05532 | - | -0.05998 | - | - |
| **PPP** | - | - | -0.02797 | 0.01245 | - | - | - |
| **DAH** | - | - | -0.14277 | 0.00801 | -0.02969 | - | - |

## Uncertainty

Since the mean forecast estimate is a function of its underlying distribution of estimates, the estimation of uncertainty is of great importance. Uncertainty intervals should be plausible; it is unreasonable to expect an upper bound of 100% of GDP going to health expenditure, as it is unprecedented and the economy could not function with such an expenditure pattern. The uncertainty interval is also policy-relevant, as it demonstrates that the future is ambiguous and amenable to policy intervention.

Four types of uncertainty were incorporated in the forecasting methodology:

1) Uncertainty in model specification
2) Uncertainty in parameter estimates
3) Uncertainty in past and forecast data
4) Fundamental uncertainty

### *Uncertainty in model specification*

Ensemble models provide a clear strategy to capture model specification uncertainty and reduce the need to find the perfect set of predictors. We described our ensemble modeling approach above. The final forecast is a result of many different models that pass empirically derived inclusion criteria.

### *Uncertainty in parameter estimates*

Uncertainty in parameter estimates was included by drawing parameter estimates from a multivariate normal distribution of the estimated model parameters as the mean and the model's variance-covariance matrix used to inform variance. We calculated 10,000 forecasts based on this estimated multivariate normal distribution around the model parameter estimates.

### *Uncertainty in past and forecast data*

We propagate uncertainty in GDP per capita forecasts by selecting a random draw to use in our forecasting calculation.

### *Fundamental uncertainty*

Fundamental uncertainty, also called systematic variance, captures general uncertainty about our forecast. In order to calculate this uncertainty, we calculate residuals (error term) for each model. For the GDP sub-models, we decomposed this total residual into a time component and a country-specific component. We

then simulated each of the decomposed residuals using a random walk process of autoregressive order 1 and a normal distribution. For the rest of the covariates, we took a random sample from a normal distribution using the specifications below.

In order to account for the skewness of the residuals, we used the medians and a capped squared median absolute deviations (MAD) for each of the residuals as the mean and variance of the normal distributions, respectively. The variance term was derived by analyzing all country-model-specific median absolute deviations of the model residuals, and then we took the MAD of all the MADs for each covariate to be forecast. Finally, we added these simulated residuals to each of the draws.

### *Putting all four types of uncertainty together*

1. We determined the number of models included in the ensemble based on the universe of potential models and the inclusion criteria.
2. We calculated the model-country-specific normal distribution of residuals and decomposed the residuals into a time- and country-specific component.
3. We determined the number of draws from each model included so that the total amount of draws exceeds 10,000; number of draws per model = ceiling of (10,000/# of models)
4. For each draw:
   a. We randomly selected a forecast GDP and GGE series from the 10,000 GDP draws (data uncertainty).
   b. We randomly selected a forecast total DAH series from the 10,000 total DAH draws (data uncertainty).
   c. We randomly drew from the variance-covariance matrix of the model (parameter estimate uncertainty).
   d. We simulated and added residuals based on the model-specific, time- and country-specific distribution of residuals (fundamental uncertainty).

We aggregated across the draws to estimate the mean and uncertainty interval (2.5$^{th}$ and 97.5$^{th}$ percentiles; model uncertainty).

## Additional tables

**Table 8. Classifications of countries by World Bank income groups**

| High-income | Upper-middle | Lower-middle | Low-income |
|---|---|---|---|
| **Andorra** | Albania | Armenia | Afghanistan |
| **Antigua and Barbuda** | Algeria | Bhutan | Bangladesh |
| **Australia** | Angola | Bolivia | Benin |
| **Austria** | Argentina | Cameroon | Burkina Faso |
| **Bahrain** | Azerbaijan | Cape Verde | Burundi |
| **Barbados** | Belarus | Congo | Cambodia |
| **Belgium** | Belize | Cote d'Ivoire | Central African Republic |
| **Brunei** | Bosnia and Herzegovina | Djibouti | Chad |
| **Canada** | Botswana | Egypt | Comoros |
| **Chile** | Brazil | El Salvador | Democratic Republic of the Congo |
| **Croatia** | Bulgaria | Federated States of Micronesia | Eritrea |
| **Cyprus** | China | Georgia | Ethiopia |
| **Czech Republic** | Colombia | Ghana | Guinea |
| **Denmark** | Costa Rica | Guatemala | Guinea-Bissau |
| **Equatorial Guinea** | Cuba | Guyana | Haiti |
| **Estonia** | Dominica | Honduras | Kenya |
| **Finland** | Dominican Republic | India | Kyrgyzstan |
| **France** | Ecuador | Indonesia | Liberia |
| **Germany** | Fiji | Kiribati | Madagascar |
| **Greece** | Gabon | Laos | Malawi |
| **Iceland** | Grenada | Lesotho | Mali |
| **Ireland** | Hungary | Mauritania | Mozambique |
| **Israel** | Iran | Moldova | Myanmar |
| **Italy** | Iraq | Mongolia | Nepal |
| **Japan** | Jamaica | Morocco | Niger |
| **Kuwait** | Jordan | Nicaragua | Rwanda |
| **Latvia** | Kazakhstan | Nigeria | Sierra Leone |
| **Lithuania** | Lebanon | Pakistan | Somalia |
| **Luxembourg** | Libya | Papua New Guinea | South Sudan |
| **Malta** | Macedonia | Paraguay | Tajikistan |
| **Netherlands** | Malaysia | Philippines | Tanzania |
| **New Zealand** | Maldives | Samoa | The Gambia |
| **Norway** | Marshall Islands | Sao Tome and Principe | Togo |
| **Oman** | Mauritius | Senegal | Uganda |
| **Poland** | Mexico | Solomon Islands | |
| **Portugal** | Montenegro | Sri Lanka | |
| **Qatar** | Namibia | Sudan | |
| **Russia** | Panama | Swaziland | |

| High-income | Upper-middle | Lower-middle | Low-income |
|---|---|---|---|
| **Saudi Arabia** | Peru | Syria | |
| **Singapore** | Romania | Timor-Leste | |
| **Slovakia** | Saint Lucia | Ukraine | |
| **Slovenia** | Vincent and the Grenadines | Uzbekistan | |
| **South Korea** | Serbia | Vanuatu | |
| **Spain** | Seychelles | Vietnam | |
| **Sweden** | South Africa | Yemen | |
| **Switzerland** | Suriname | Zambia | |
| **The Bahamas** | Thailand | | |
| **Trinidad and Tobago** | Tonga | | |
| **United Arab Emirates** | Tunisia | | |
| **United Kingdom** | Turkey | | |
| **United States** | Turkmenistan | | |
| **Uruguay** | Venezuela | | |

**Table 9. Classifications of countries by Global Burden of Disease geographical regions**

| High-income | Central Europe, Eastern Europe, and Central Asia | Sub-Saharan Africa | North Africa and Middle East | South Asia | Southeast Asia, East Asia, and Oceania | Latin America and Caribbean |
|---|---|---|---|---|---|---|
| **Andorra** | Albania | Angola | Afghanistan | Bangla desh | Cambodia | Antigua and Barbuda |
| **Argentina** | Armenia | Benin | Algeria | Bhutan | China | Barbados |
| **Australia** | Azerbaijan | Botswana | Bahrain | India | Federated States of Micronesia | Belize |
| **Austria** | Belarus | Burkina Faso | Egypt | Nepal | Fiji | Bolivia |
| **Belgium** | Bosnia and Herzegovina | Burundi | Iran | Pakista n | Indonesia | Brazil |
| **Brunei** | Bulgaria | Cameroon | Iraq | | Kiribati | Colombia |
| **Canada** | Croatia | Cape Verde | Jordan | | Laos | Costa Rica |
| **Chile** | Czech Republic | Central African Republic | Kuwait | | Malaysia | Cuba |
| **Cyprus** | Estonia | Chad | Lebanon | | Maldives | Dominica |
| **Denmark** | Georgia | Comoros | Libya | | Marshall Islands | Dominican Republic |
| **Finland** | Hungary | Congo | Morocco | | Mauritius | Ecuador |
| **France** | Kazakhstan | Cote d'Ivoire | Oman | | Myanmar | El Salvador |
| **Germany** | Kyrgyzstan | Democratic Republic of the Congo | Qatar | | Papua New Guinea | Grenada |
| **Greece** | Latvia | Djibouti | Saudi Arabia | | Philippines | Guatemala |
| **Iceland** | Lithuania | Equatorial Guinea | Sudan | | Samoa | Guyana |
| **Ireland** | Macedonia | Eritrea | Syria | | Seychelles | Haiti |
| **Israel** | Moldova | Ethiopia | Tunisia | | Solomon Islands | Honduras |
| **Italy** | Mongolia | Gabon | Turkey | | Sri Lanka | Jamaica |
| **Japan** | Montenegro | Ghana | United Arab Emirates | | Thailand | Mexico |
| **Luxembourg** | Poland | Guinea | Yemen | | Timor-Leste | Nicaragua |
| **Malta** | Romania | Guinea-Bissau | | | Tonga | Panama |
| **Netherlands** | Russia | Kenya | | | Vanuatu | Paraguay |
| **New Zealand** | Serbia | Lesotho | | | Vietnam | Peru |
| **Norway** | Slovakia | Liberia | | | | Saint Lucia |
| **Portugal** | Slovenia | Madagascar | | | | Saint Vincent and the Grenadines |
| **Singapore** | Tajikistan | Malawi | | | | Suriname |
| **South Korea** | Turkmenistan | Mali | | | | The Bahamas |
| **Spain** | Ukraine | Mauritania | | | | Trinidad and Tobago |
| **Sweden** | Uzbekistan | Mozambique | | | | Venezuela |
| **Switzerland** | | Namibia | | | | |
| **United Kingdom** | | Niger | | | | |
| **United States** | | Nigeria | | | | |

| High-income | Central Europe, Eastern Europe, and Central Asia | Sub-Saharan Africa | North Africa and Middle East | South Asia | Southeast Asia, East Asia, and Oceania | Latin America and Caribbean |
|---|---|---|---|---|---|---|
| **Uruguay** | | Rwanda | | | | |
| | | Sao Tome and Principe | | | | |
| | | Senegal | | | | |
| | | Sierra Leone | | | | |
| | | Somalia | | | | |
| | | South Africa | | | | |
| | | South Sudan | | | | |
| | | Swaziland | | | | |
| | | Tanzania | | | | |
| | | The Gambia | | | | |
| | | Togo | | | | |
| | | Uganda | | | | |
| | | Zambia | | | | |

**Table 10. Observed health spending in 2014 and expected health spending in 2030**

| | 2014 total health spending | | 2030 expected health spending | | 2030 expected health spending by source | | | |
|---|---|---|---|---|---|---|---|---|
| | Per capita ($) | Per GDP (%) | Per capita ($) | Per GDP(%) | Government as a share of total (%) | Prepaid private as a share of total (%) | Out-of-pocket as a share of total (%) | Development assistance as a share of total (%) |
| Global | 1279 | 8.3 | 1983 (1793, 2199) | 8.2 (7.4, 9.1) | 62.0 (57.9, 66.0) | 15.1 (13.3, 17.2) | 22.4 (19.7, 25.3) | 0.5 (0.3, 0.7) |
| | | | | | | | | |
| High-income | 5221 | 11.7 | 7334 (6786, 7815) | 12.5 (11.5, 13.3) | 64.2 (61.1, 66.7) | 22.7 (20.8, 25.2) | 13.1 (12.0, 14.4) | 0.0 (0.0, 0.0) |
| Upper-middle-income | 914 | 5.9 | 2072 (1698, 2583) | 6.4 (5.2, 7.9) | 66.0 (58.4, 73.8) | 7.2 (5.5, 9.2) | 26.7 (20.1, 33.3) | 0.1 (0.0, 0.2) |
| Lower-middle-income | 267 | 4.3 | 525 (485, 582) | 4.7 (4.3, 5.1) | 41.3 (36.7, 46.9) | 2.9 (2.5, 3.2) | 54.1 (48.8, 58.4) | 1.8 (1.0, 2.8) |
| Lower-income | 120 | 7.3 | 154 (133, 181) | 6.6 (5.8, 7.8) | 25.6 (20.8, 30.6) | 14.0 (11.4, 16.7) | 30.5 (25.5, 35.4) | 29.9 (20.3, 40.6) |
| | | | | | | | | |
| Central Europe, Eastern Europe, and Central Asia | 1364 | 6.7 | 1877 (1766, 2018) | 6.9 (6.5, 7.4) | 60.8 (56.8, 63.5) | 3.1 (2.7, 3.6) | 35.9 (33.2, 40.0) | 0.2 (0.1, 0.4) |
| High-income | 5460 | 12.3 | 7643 (7076, 8146) | 13.1 (12.1, 14.0) | 63.5 (60.3, 66.0) | 23.4 (21.5, 26.0) | 13.1 (11.9, 14.4) | 0.0 (0.0, 0.0) |
| Latin America and Caribbean | 1082 | 7.3 | 1534 (1350, 1745) | 8.2 (7.2, 9.3) | 56.0 (50.2, 61.6) | 15.4 (13.0, 18.6) | 28.2 (24.4, 32.5) | 0.4 (0.1, 0.7) |
| North Africa and Middle East | 870 | 5.2 | 1246 (1137, 1416) | 5.8 (5.3, 6.6) | 62.3 (58.4, 67.1) | 4.1 (3.5, 4.7) | 33.0 (28.7, 36.6) | 0.7 (0.4, 1.1) |
| South Asia | 223 | 4.2 | 529 (467, 619) | 4.8 (4.2, 5.6) | 38.5 (31.4, 47.8) | 2.2 (1.9, 2.6) | 58.5 (49.7, 65.3) | 0.8 (0.4, 1.3) |
| Southeast Asia, East Asia, and Oceania | 588 | 4.8 | 1867 (1436, 2471) | 5.6 (4.3, 7.4) | 68.5 (58.6, 78.0) | 5.0 (3.5, 7.0) | 26.3 (17.9, 35.5) | 0.1 (0.1, 0.3) |
| Sub-Saharan Africa | 218 | 5.9 | 259 (238, 286) | 5.6 (5.2, 6.2) | 36.9 (32.9, 41.0) | 16.8 (14.9, 18.7) | 31.0 (27.6, 34.4) | 15.3 (9.5, 22.2) |
| | | | | | | | | |
| Afghanistan | 159 | 9.7 | 201 (161, 268) | 10.2 (8.1, 13.6) | 16.2 (9.5, 32.5) | 0.5 (0.4, 0.7) | 51.3 (37.6, 62.8) | 32.0 (20.9, 47.9) |
| Albania | 642 | 5.9 | 1202 (1022, 1424) | 6.6 (5.6, 7.8) | 56.7 (50.0, 64.6) | 0.8 (0.6, 1.0) | 41.8 (34.2, 48.4) | 0.7 (0.0, 1.7) |
| Algeria | 1004 | 7.2 | 1567 (1248, 2146) | 9.1 (7.2, 12.4) | 78.9 (72.7, 85.5) | 0.6 (0.4, 0.9) | 20.4 (14.0, 26.6) | 0.0 (0.0, 0.1) |
| Andorra | 5723 | 8.1 | 7230 (5789, 8606) | 8.6 (6.9, 10.3) | 78.7 (73.5, 82.8) | 6.2 (4.9, 8.0) | 15.1 (12.0, 18.9) | 0.0 (0.0, 0.0) |
| Angola | 228 | 3 | 256 (169, 321) | 2.5 (1.7, 3.1) | 64.1 (46.6, 73.4) | 2.1 (1.5, 3.2) | 30.7 (22.1, 46.3) | 3.1 (1.5, 5.9) |
| Antigua and Barbuda | 1213 | 5.5 | 2165 (1727, 2767) | 7.4 (5.9, 9.4) | 74.9 (68.3, 81.1) | 7.0 (5.1, 9.1) | 18.2 (13.4, 23.5) | 0.0 (0.0, 0.0) |

| | 2014 total health spending | | 2030 expected health spending | | 2030 expected health spending by source | | | |
|---|---|---|---|---|---|---|---|---|
| | Per capita ($) | Per GDP (%) | Per capita ($) | Per GDP(%) | Government as a share of total (%) | Prepaid private as a share of total (%) | Out-of-pocket as a share of total (%) | Development assistance as a share of total (%) |
| Argentina | 1322 | 4.8 | 2177 (1769, 2985) | 5.7 (4.6, 7.8) | 62.3 (54.0, 73.5) | 11.7 (8.1, 14.9) | 26.0 (17.9, 33.1) | 0.0 (0.0, 0.0) |
| Armenia | 395 | 4.5 | 674 (549, 907) | 4.9 (4.0, 6.7) | 48.0 (38.3, 62.2) | 3.2 (2.2, 4.4) | 46.4 (33.5, 55.7) | 2.4 (0.7, 5.4) |
| Australia | 4032 | 9 | 5606 (5186, 6165) | 9.7 (9.0, 10.7) | 71.5 (68.1, 74.7) | 9.8 (8.5, 11.5) | 18.7 (16.1, 22.0) | 0.0 (0.0, 0.0) |
| Austria | 5471 | 11.2 | 7416 (6788, 8143) | 11.6 (10.6, 12.7) | 78.8 (76.3, 81.2) | 5.7 (4.9, 7.2) | 15.5 (13.5, 17.5) | 0.0 (0.0, 0.0) |
| Azerbaijan | 1030 | 5.9 | 1734 (1524, 1978) | 6.3 (5.5, 7.2) | 24.2 (18.6, 32.1) | 4.0 (3.3, 5.1) | 71.7 (64.0, 77.4) | 0.0 (0.0, 0.5) |
| Bahrain | 2258 | 4.8 | 3289 (2738, 4136) | 5.3 (4.4, 6.7) | 70.7 (64.6, 77.3) | 10.1 (7.6, 13.1) | 19.2 (14.6, 24.0) | 0.0 (0.0, 0.0) |
| Bangladesh | 92 | 2.9 | 173 (149, 198) | 2.8 (2.4, 3.2) | 26.9 (21.4, 34.3) | 1.8 (1.4, 2.2) | 65.3 (57.6, 71.5) | 6.1 (2.6, 10.9) |
| Barbados | 1116 | 7.5 | 1641 (1412, 1926) | 8.7 (7.5, 10.2) | 66.9 (60.8, 72.8) | 6.3 (5.0, 8.0) | 26.8 (21.6, 32.6) | 0.0 (0.0, 0.0) |
| Belarus | 1093 | 5.6 | 1825 (1432, 2308) | 7.0 (5.5, 8.9) | 67.5 (58.2, 76.4) | 0.7 (0.5, 1.1) | 31.7 (22.9, 40.9) | 0.0 (0.0, 0.1) |
| Belgium | 4751 | 10.6 | 6437 (5759, 7278) | 11.2 (10.0, 12.7) | 79.2 (76.5, 82.1) | 4.2 (3.4, 5.0) | 16.6 (14.2, 18.9) | 0.0 (0.0, 0.0) |
| Belize | 503 | 5.8 | 678 (593, 776) | 6.3 (5.5, 7.2) | 66.7 (61.6, 71.5) | 9.7 (8.0, 11.9) | 21.2 (17.8, 24.8) | 2.5 (1.1, 4.4) |
| Benin | 105 | 5.1 | 169 (134, 221) | 6.2 (4.9, 8.1) | 49.9 (38.6, 62.6) | 1.3 (0.7, 1.9) | 29.3 (21.6, 36.9) | 19.4 (10.9, 30.0) |
| Bhutan | 279 | 3.6 | 563 (397, 774) | 3.5 (2.5, 4.8) | 73.3 (62.4, 82.1) | 1.5 (1.0, 2.2) | 23.6 (15.5, 33.7) | 1.6 (0.5, 3.2) |
| Bolivia | 404 | 6.3 | 673 (565, 814) | 7.3 (6.1, 8.8) | 75.3 (70.1, 80.4) | 2.9 (2.1, 4.2) | 19.9 (15.6, 24.6) | 1.8 (0.9, 3.1) |
| Bosnia and Herzegovina | 992 | 9.5 | 1734 (1331, 2104) | 10.4 (8.0, 12.6) | 75.4 (67.9, 81.2) | 0.5 (0.4, 0.6) | 23.2 (17.6, 30.3) | 0.9 (0.0, 2.3) |
| Botswana | 903 | 5.5 | 1395 (1168, 1723) | 6.3 (5.2, 7.7) | 58.2 (49.4, 67.2) | 34.9 (27.5, 42.2) | 4.5 (3.4, 5.7) | 2.3 (0.0, 10.0) |
| Brazil | 1357 | 8.3 | 1994 (1657, 2402) | 10.0 (8.3, 12.1) | 51.7 (42.5, 60.4) | 26.6 (21.3, 32.9) | 21.6 (17.1, 27.2) | 0.0 (0.0, 0.1) |
| Brunei | 1811 | 2.6 | 2254 (1741, 3135) | 3.5 (2.7, 4.8) | 93.7 (90.8, 95.9) | 1.5 (1.0, 2.0) | 4.8 (2.9, 7.5) | 0.0 (0.0, 0.0) |
| Bulgaria | 1490 | 8.4 | 2659 (2116, 3624) | 9.7 (7.7, 13.2) | 58.3 (48.4, 70.3) | 0.7 (0.5, 1.2) | 41.0 (29.2, 50.8) | 0.0 (0.0, 0.0) |
| Burkina Faso | 83 | 5 | 108 (93, 127) | 5.0 (4.3, 5.9) | 39.9 (32.4, 46.8) | 1.0 (0.8, 1.4) | 38.3 (31.4, 45.3) | 20.8 (12.4, 31.4) |
| Burundi | 65 | 8.3 | 85 (62, 120) | 9.6 (7.0, 13.6) | 30.9 (19.3, 43.0) | 0.8 (0.5, 1.2) | 17.3 (11.4, 23.9) | 51.0 (35.6, 66.9) |
| Cambodia | 209 | 6.4 | 397 (352, 448) | 6.0 (5.3, 6.7) | 20.9 (14.5, 27.0) | 0.9 (0.8, 1.2) | 68.3 (60.9, 75.5) | 9.9 (4.6, 16.8) |
| Cameroon | 116 | 4 | 156 (135, 179) | 4.1 (3.5, 4.7) | 21.8 (16.2, 29.5) | 3.4 (2.8, 4.3) | 66.1 (58.4, 72.8) | 8.6 (4.7, 14.0) |
| Canada | 4576 | 10.3 | 5926 (5389, 6601) | 10.7 (9.7, 11.9) | 73.9 (70.9, 77.0) | 13.2 (11.4, 15.0) | 12.9 (11.0, 15.0) | 0.0 (0.0, 0.0) |

| | 2014 total health spending | | 2030 expected health spending | | 2030 expected health spending by source | | | |
|---|---|---|---|---|---|---|---|---|
| | Per capita ($) | Per GDP (%) | Per capita ($) | Per GDP(%) | Government as a share of total (%) | Prepaid private as a share of total (%) | Out-of-pocket as a share of total (%) | Development assistance as a share of total (%) |
| Cape Verde | 318 | 4.8 | 529 (412, 686) | 4.8 (3.8, 6.3) | 65.2 (54.9, 74.4) | 1.1 (0.8, 1.6) | 22.3 (16.6, 28.7) | 11.4 (5.3, 19.5) |
| Central African Republic | 35 | 5.7 | 46 (29, 77) | 9.4 (6.0, 15.8) | 11.9 (5.4, 18.6) | 0.6 (0.3, 0.9) | 22.7 (12.4, 34.7) | 64.8 (47.9, 80.6) |
| Chad | 89 | 3.8 | 111 (74, 150) | 3.9 (2.6, 5.3) | 50.2 (29.8, 65.1) | 1.5 (1.0, 2.3) | 37.0 (25.4, 53.1) | 11.3 (5.5, 20.3) |
| Chile | 1780 | 7.8 | 3217 (2622, 3793) | 8.8 (7.1, 10.3) | 54.3 (44.4, 61.7) | 17.0 (13.9, 21.0) | 28.8 (23.7, 35.4) | 0.0 (0.0, 0.0) |
| China | 697 | 5.1 | 2493 (1851, 3402) | 6.0 (4.5, 8.2) | 70.4 (59.5, 80.3) | 4.9 (3.3, 7.0) | 24.7 (15.8, 34.8) | 0.0 (0.0, 0.0) |
| Colombia | 975 | 7.2 | 1620 (1168, 2206) | 7.8 (5.7, 10.7) | 75.6 (66.5, 82.9) | 10.4 (7.2, 14.7) | 13.0 (8.9, 18.2) | 1.0 (0.0, 3.3) |
| Comoros | 111 | 7.1 | 121 (101, 148) | 8.6 (7.1, 10.5) | 24.8 (17.4, 36.1) | 18.2 (14.6, 22.1) | 40.5 (31.7, 49.2) | 16.5 (8.9, 27.4) |
| Congo | 312 | 5.2 | 424 (336, 543) | 6.1 (4.8, 7.8) | 82.7 (77.5, 87.3) | 0.8 (0.6, 1.1) | 15.0 (11.0, 20.0) | 1.4 (0.6, 2.5) |
| Costa Rica | 1418 | 9.3 | 2142 (1628, 2636) | 9.0 (6.8, 11.1) | 72.5 (63.9, 78.5) | 1.8 (1.4, 2.4) | 25.7 (20.0, 33.7) | 0.0 (0.0, 0.0) |
| Cote d'Ivoire | 179 | 5.3 | 242 (214, 275) | 5.4 (4.8, 6.1) | 28.1 (22.5, 34.8) | 8.3 (6.7, 10.1) | 51.8 (45.5, 57.8) | 11.7 (6.5, 18.8) |
| Croatia | 1734 | 7.8 | 2263 (2064, 2445) | 7.8 (7.1, 8.5) | 81.4 (78.5, 83.7) | 7.4 (6.4, 9.1) | 11.1 (9.3, 13.6) | 0.0 (0.0, 0.0) |
| Cuba | 1706 | 11.1 | 2326 (1635, 3134) | 11.3 (7.9, 15.2) | 95.0 (92.8, 96.5) | 0.5 (0.3, 0.6) | 4.4 (3.1, 6.4) | 0.1 (0.0, 0.3) |
| Cyprus | 2019 | 7.2 | 2864 (2520, 3352) | 8.0 (7.0, 9.4) | 51.4 (45.3, 58.8) | 4.4 (3.5, 5.6) | 44.2 (37.3, 49.9) | 0.0 (0.0, 0.0) |
| Czech Republic | 2384 | 7.4 | 3146 (2753, 3657) | 7.1 (6.3, 8.3) | 84.6 (81.8, 87.2) | 0.9 (0.7, 1.2) | 14.5 (12.0, 17.3) | 0.0 (0.0, 0.0) |
| Democratic Republic of the Congo | 46 | 4.5 | 67 (52, 86) | 5.1 (3.9, 6.6) | 30.6 (20.8, 43.2) | 1.0 (0.7, 1.5) | 36.9 (26.2, 47.2) | 31.5 (19.1, 46.4) |
| Denmark | 5075 | 10.8 | 6251 (5488, 6890) | 10.7 (9.4, 11.8) | 84.4 (81.9, 86.5) | 2.1 (1.8, 2.7) | 13.4 (11.5, 15.8) | 0.0 (0.0, 0.0) |
| Djibouti | 357 | 10.9 | 613 (486, 838) | 13.9 (11.0, 18.9) | 65.2 (56.2, 75.9) | 0.4 (0.3, 0.5) | 29.2 (20.1, 37.2) | 5.2 (2.6, 8.8) |
| Dominica | 599 | 5.5 | 859 (740, 1012) | 6.2 (5.3, 7.3) | 71.5 (65.9, 76.8) | 2.7 (2.1, 3.5) | 25.7 (20.8, 31.2) | 0.0 (0.0, 0.1) |
| Dominican Republic | 601 | 4.4 | 1211 (930, 1567) | 4.9 (3.7, 6.3) | 72.5 (63.8, 79.8) | 10.9 (7.9, 14.8) | 16.3 (11.3, 22.3) | 0.3 (0.0, 2.4) |
| Ecuador | 1071 | 9.2 | 1491 (1261, 1758) | 10.2 (8.6, 12.0) | 50.3 (41.9, 58.5) | 2.1 (1.6, 2.7) | 47.3 (39.3, 55.4) | 0.3 (0.2, 0.6) |
| Egypt | 581 | 5.4 | 903 (820, 1016) | 5.5 (4.9, 6.1) | 39.0 (34.4, 45.6) | 1.6 (1.3, 2.1) | 59.3 (52.8, 63.8) | 0.1 (0.1, 0.2) |
| El Salvador | 567 | 6.8 | 1018 (826, 1354) | 7.7 (6.3, 10.3) | 70.8 (63.7, 79.1) | 4.9 (3.2, 6.6) | 23.3 (16.5, 29.9) | 0.9 (0.3, 1.9) |
| Equatorial Guinea | 1411 | 3.7 | 1435 (1163, 1792) | 3.6 (2.9, 4.5) | 77.2 (69.2, 83.0) | 1.4 (1.1, 1.8) | 21.3 (15.8, 29.4) | 0.0 (0.0, 0.0) |
| Eritrea | 59 | 5.1 | 68 (53, 88) | 4.8 (3.7, 6.2) | 21.9 (13.6, 30.4) | 1.1 (0.8, 1.5) | 37.4 (27.7, 47.2) | 39.6 (25.9, 54.8) |

| | 2014 total health spending | | 2030 expected health spending | | 2030 expected health spending by source | | | |
|---|---|---|---|---|---|---|---|---|
| | Per capita ($) | Per GDP (%) | Per capita ($) | Per GDP(%) | Government as a share of total (%) | Prepaid private as a share of total (%) | Out-of-pocket as a share of total (%) | Development assistance as a share of total (%) |
| Estonia | 1830 | 6.4 | 3274 (2683, 4230) | 7.9 (6.5, 10.2) | 80.7 (75.1, 85.9) | 0.6 (0.5, 0.8) | 18.7 (13.6, 24.2) | 0.0 (0.0, 0.0) |
| Ethiopia | 85 | 5.5 | 149 (115, 197) | 4.9 (3.8, 6.5) | 33.6 (23.9, 43.3) | 1.1 (0.8, 1.5) | 30.5 (21.7, 39.5) | 34.9 (19.6, 51.3) |
| Federated States of Micronesia | 490 | 16.1 | 608 (359, 972) | 17.2 (10.1, 27.5) | 8.4 (3.7, 15.8) | 0.3 (0.2, 0.5) | 7.8 (4.4, 12.6) | 83.5 (72.8, 91.1) |
| Fiji | 399 | 4.5 | 558 (503, 614) | 4.6 (4.1, 5.0) | 64.1 (59.0, 68.5) | 8.1 (6.8, 9.7) | 22.9 (19.3, 27.8) | 4.9 (2.4, 8.7) |
| Finland | 3935 | 9.3 | 5061 (4654, 5562) | 9.5 (8.8, 10.5) | 78.4 (76.0, 80.9) | 3.2 (2.7, 3.8) | 18.4 (16.1, 20.7) | 0.0 (0.0, 0.0) |
| France | 4589 | 11.3 | 5963 (5487, 6689) | 11.6 (10.6, 13.0) | 79.5 (76.8, 82.2) | 14.3 (12.2, 16.7) | 6.2 (5.2, 7.3) | 0.0 (0.0, 0.0) |
| Gabon | 612 | 3.4 | 985 (799, 1248) | 4.7 (3.9, 6.0) | 77.5 (71.6, 83.0) | 6.1 (4.5, 8.3) | 15.7 (11.5, 20.5) | 0.7 (0.0, 2.0) |
| Georgia | 700 | 7.3 | 1236 (1026, 1427) | 8.9 (7.4, 10.3) | 20.9 (15.6, 29.0) | 31.5 (21.9, 38.4) | 45.9 (38.7, 54.6) | 1.8 (0.6, 3.7) |
| Germany | 5356 | 11.2 | 7612 (6630, 8575) | 12.0 (10.5, 13.5) | 78.8 (75.4, 81.6) | 8.6 (7.3, 10.1) | 12.6 (10.6, 14.9) | 0.0 (0.0, 0.0) |
| Ghana | 146 | 3.5 | 218 (177, 264) | 3.7 (3.0, 4.4) | 59.3 (49.9, 67.5) | 3.0 (2.3, 4.1) | 26.0 (20.5, 32.6) | 11.8 (6.4, 18.7) |
| Greece | 2170 | 8.1 | 2833 (2484, 3383) | 8.3 (7.3, 9.9) | 62.7 (57.5, 68.9) | 3.8 (3.0, 5.0) | 33.5 (27.9, 38.4) | 0.0 (0.0, 0.0) |
| Grenada | 737 | 6.1 | 1096 (967, 1259) | 6.3 (5.6, 7.2) | 49.2 (43.6, 56.4) | 2.4 (2.0, 2.9) | 48.3 (41.2, 53.8) | 0.1 (0.0, 0.3) |
| Guatemala | 466 | 6.2 | 594 (540, 648) | 6.2 (5.6, 6.7) | 37.1 (32.3, 41.8) | 8.6 (7.5, 10.0) | 51.7 (47.1, 56.3) | 2.5 (1.3, 4.2) |
| Guinea | 101 | 7.4 | 127 (100, 163) | 7.9 (6.2, 10.1) | 34.8 (24.1, 46.1) | 1.0 (0.5, 1.5) | 33.8 (25.6, 42.7) | 30.4 (18.3, 45.2) |
| Guinea-Bissau | 77 | 5.3 | 98 (75, 131) | 5.7 (4.4, 7.6) | 3.2 (1.8, 6.9) | 0.9 (0.6, 1.2) | 49.0 (35.6, 62.8) | 46.9 (32.2, 61.3) |
| Guyana | 438 | 5.4 | 685 (589, 812) | 5.8 (5.0, 6.9) | 56.6 (49.6, 64.1) | 2.9 (2.3, 3.7) | 35.6 (29.2, 42.2) | 4.9 (2.3, 8.7) |
| Haiti | 154 | 8.9 | 205 (164, 262) | 9.4 (7.5, 12.0) | 0.7 (0.4, 1.5) | 33.9 (25.8, 42.1) | 27.8 (21.0, 34.9) | 37.5 (24.1, 51.4) |
| Honduras | 420 | 8.8 | 568 (513, 654) | 8.8 (8.0, 10.1) | 49.5 (44.5, 56.4) | 5.1 (3.9, 6.6) | 41.9 (36.0, 46.7) | 3.5 (1.4, 6.1) |
| Hungary | 1855 | 7.2 | 2706 (2522, 3028) | 7.3 (6.8, 8.2) | 68.6 (64.9, 72.4) | 4.1 (3.5, 4.8) | 27.2 (23.8, 31.1) | 0.0 (0.0, 0.0) |
| Iceland | 3959 | 8.7 | 5491 (4824, 6314) | 9.2 (8.1, 10.6) | 82.6 (79.7, 85.4) | 0.5 (0.4, 0.6) | 16.9 (14.1, 19.7) | 0.0 (0.0, 0.0) |
| India | 253 | 4.5 | 629 (550, 747) | 5.1 (4.4, 6.0) | 38.7 (31.0, 48.7) | 2.0 (1.7, 2.4) | 59.0 (49.3, 66.5) | 0.3 (0.1, 0.5) |
| Indonesia | 265 | 2.5 | 509 (443, 588) | 2.6 (2.3, 3.0) | 45.5 (38.6, 53.1) | 2.9 (2.3, 3.7) | 51.2 (43.8, 57.9) | 0.4 (0.0, 1.0) |
| Iran | 1073 | 6.5 | 1558 (1263, 1874) | 7.3 (5.9, 8.8) | 44.9 (33.5, 54.2) | 5.8 (4.5, 7.7) | 49.2 (40.5, 60.1) | 0.0 (0.0, 0.0) |
| Iraq | 828 | 5.7 | 1018 (787, 1401) | 5.9 (4.6, 8.2) | 60.4 (49.6, 72.2) | 3.3 (2.2, 4.4) | 36.0 (25.1, 46.3) | 0.3 (0.0, 0.6) |

| | 2014 total health spending | | 2030 expected health spending | | 2030 expected health spending by source | | | |
|---|---|---|---|---|---|---|---|---|
| | Per capita ($) | Per GDP (%) | Per capita ($) | Per GDP(%) | Government as a share of total (%) | Prepaid private as a share of total (%) | Out-of-pocket as a share of total (%) | Development assistance as a share of total (%) |
| Ireland | 4006 | 7.6 | 5989 (4758, 7222) | 7.8 (6.2, 9.4) | 66.6 (58.2, 73.0) | 15.8 (12.5, 20.4) | 17.6 (13.8, 22.5) | 0.0 (0.0, 0.0) |
| Israel | 2722 | 7.7 | 3747 (3312, 4249) | 8.4 (7.4, 9.5) | 61.5 (56.4, 66.6) | 11.8 (10.0, 14.1) | 26.7 (22.7, 30.8) | 0.0 (0.0, 0.0) |
| Italy | 3311 | 9 | 4154 (3805, 4502) | 8.8 (8.1, 9.6) | 77.1 (73.0, 80.6) | 0.9 (0.8, 1.2) | 22.0 (18.4, 26.1) | 0.0 (0.0, 0.0) |
| Jamaica | 477 | 5.4 | 773 (650, 955) | 7.0 (5.9, 8.6) | 59.9 (52.7, 68.0) | 16.3 (12.7, 20.0) | 22.2 (17.5, 26.8) | 1.6 (0.7, 3.0) |
| Japan | 3816 | 10.2 | 5729 (4452, 6820) | 11.7 (9.1, 13.9) | 85.2 (80.9, 88.2) | 2.4 (1.9, 3.3) | 12.4 (9.8, 16.1) | 0.0 (0.0, 0.0) |
| Jordan | 839 | 7.4 | 1097 (982, 1226) | 7.4 (6.6, 8.3) | 66.6 (61.6, 71.1) | 9.0 (7.6, 10.9) | 20.6 (16.9, 25.2) | 3.7 (1.9, 6.3) |
| Kazakhstan | 1143 | 4.3 | 1545 (1343, 1817) | 4.2 (3.6, 4.9) | 54.5 (48.3, 61.7) | 1.2 (0.9, 1.4) | 44.3 (37.3, 50.5) | 0.0 (0.0, 0.0) |
| Kenya | 197 | 6.4 | 237 (194, 302) | 5.9 (4.9, 7.6) | 36.8 (28.3, 50.0) | 4.9 (3.7, 6.0) | 25.7 (19.6, 31.9) | 32.6 (20.6, 45.3) |
| Kiribati | 168 | 9.6 | 184 (81, 281) | 9.9 (4.4, 15.2) | 71.9 (43.5, 86.7) | 0.6 (0.3, 1.2) | 3.0 (1.6, 6.1) | 24.6 (10.7, 50.8) |
| Kuwait | 2075 | 3 | 3208 (2309, 4950) | 4.2 (3.0, 6.5) | 88.9 (83.9, 93.4) | 1.3 (0.8, 1.7) | 9.8 (5.8, 14.6) | 0.0 (0.0, 0.0) |
| Kyrgyzstan | 236 | 6.9 | 315 (272, 369) | 7.4 (6.4, 8.6) | 51.2 (43.7, 58.8) | 1.3 (1.0, 1.7) | 35.6 (29.3, 42.0) | 11.9 (6.6, 19.3) |
| Laos | 113 | 2 | 186 (144, 234) | 1.5 (1.2, 1.9) | 32.3 (22.1, 43.9) | 3.0 (2.1, 4.2) | 41.3 (30.3, 52.6) | 23.5 (12.3, 37.0) |
| Latvia | 1427 | 5.9 | 2036 (1833, 2247) | 5.8 (5.2, 6.4) | 62.4 (57.0, 67.4) | 1.9 (1.6, 2.8) | 35.7 (30.7, 41.1) | 0.0 (0.0, 0.0) |
| Lebanon | 1060 | 6.4 | 1484 (1222, 1825) | 6.3 (5.2, 7.8) | 49.2 (39.7, 59.8) | 16.0 (12.5, 20.1) | 34.4 (25.8, 42.9) | 0.3 (0.0, 1.5) |
| Lesotho | 319 | 11.6 | 521 (371, 667) | 12.3 (8.8, 15.8) | 66.7 (53.0, 76.7) | 0.4 (0.3, 0.7) | 13.4 (9.7, 18.9) | 19.5 (10.3, 31.7) |
| Liberia | 345 | 39.3 | 287 (257, 333) | 27.1 (24.3, 31.4) | 0.7 (0.2, 2.0) | 0.2 (0.2, 0.3) | 11.2 (9.1, 13.4) | 87.9 (85.4, 90.2) |
| Libya | 751 | 5 | 781 (534, 1147) | 6.8 (4.7, 10.0) | 79.2 (66.3, 88.0) | 0.8 (0.5, 1.1) | 19.9 (11.4, 32.8) | 0.1 (0.0, 0.2) |
| Lithuania | 1830 | 6.5 | 2904 (2579, 3381) | 6.6 (5.9, 7.7) | 66.9 (62.1, 72.0) | 0.8 (0.7, 1.1) | 32.3 (27.2, 37.0) | 0.0 (0.0, 0.0) |
| Luxembourg | 7105 | 6.9 | 10593 (9569, 12306) | 7.4 (6.7, 8.6) | 84.6 (82.2, 87.1) | 5.5 (4.5, 7.1) | 9.9 (8.0, 11.6) | 0.0 (0.0, 0.0) |
| Macedonia | 887 | 6.5 | 1368 (1240, 1504) | 6.8 (6.2, 7.5) | 61.7 (56.7, 66.7) | 0.7 (0.6, 0.9) | 37.4 (32.4, 42.4) | 0.1 (0.0, 0.4) |
| Madagascar | 52 | 3.7 | 65 (54, 80) | 4.2 (3.5, 5.2) | 38.2 (30.2, 45.7) | 1.3 (0.9, 1.7) | 30.6 (23.9, 37.6) | 29.9 (18.6, 43.2) |
| Malawi | 148 | 12.9 | 184 (148, 233) | 13.4 (10.8, 17.0) | 43.1 (33.0, 52.8) | 12.5 (9.3, 16.0) | 8.9 (6.7, 11.5) | 35.5 (23.1, 49.3) |
| Malaysia | 1047 | 4.1 | 1783 (1576, 2102) | 4.1 (3.6, 4.8) | 55.3 (50.0, 62.7) | 9.3 (7.5, 11.4) | 35.4 (29.0, 40.4) | 0.0 (0.0, 0.0) |
| Maldives | 1980 | 13.5 | 3623 (2656, 5154) | 13.1 (9.6, 18.6) | 77.7 (69.8, 85.1) | 2.2 (1.5, 3.2) | 20.0 (13.3, 27.4) | 0.0 (0.0, 0.0) |

| | 2014 total health spending | | 2030 expected health spending | | 2030 expected health spending by source | | | |
|---|---|---|---|---|---|---|---|---|
| | Per capita ($) | Per GDP (%) | Per capita ($) | Per GDP(%) | Government as a share of total (%) | Prepaid private as a share of total (%) | Out-of-pocket as a share of total (%) | Development assistance as a share of total (%) |
| Mali | 162 | 7.4 | 229 (193, 275) | 7.3 (6.2, 8.8) | 31.4 (23.3, 41.5) | 9.9 (6.9, 13.8) | 41.9 (34.0, 49.6) | 16.8 (9.7, 26.4) |
| Malta | 3058 | 9.7 | 5997 (5097, 7328) | 12.1 (10.3, 14.8) | 74.9 (69.8, 80.4) | 1.8 (1.4, 2.3) | 23.3 (18.1, 28.2) | 0.0 (0.0, 0.0) |
| Marshall Islands | 599 | 17.2 | 679 (495, 851) | 15.7 (11.5, 19.7) | 64.3 (50.3, 74.6) | 2.4 (1.7, 3.4) | 12.7 (9.6, 17.4) | 20.7 (10.9, 34.2) |
| Mauritania | 153 | 3.7 | 204 (171, 251) | 4.0 (3.3, 4.9) | 50.0 (41.6, 60.1) | 1.5 (1.1, 1.9) | 41.3 (32.6, 49.4) | 7.1 (3.8, 11.8) |
| Mauritius | 880 | 4.6 | 1942 (1454, 2542) | 5.5 (4.1, 7.2) | 60.1 (47.5, 70.8) | 1.0 (0.7, 1.5) | 38.9 (28.4, 51.3) | 0.0 (0.0, 0.0) |
| Mexico | 1088 | 6.3 | 1413 (1217, 1611) | 6.7 (5.8, 7.7) | 53.3 (45.8, 59.2) | 4.6 (3.7, 5.8) | 42.0 (36.5, 49.3) | 0.0 (0.0, 0.1) |
| Moldova | 527 | 10.3 | 711 (620, 822) | 10.5 (9.1, 12.1) | 46.5 (39.3, 53.6) | 9.1 (7.4, 11.6) | 39.8 (33.4, 46.8) | 4.7 (1.0, 10.8) |
| Mongolia | 575 | 4.7 | 1078 (837, 1406) | 4.7 (3.7, 6.2) | 55.2 (43.7, 67.3) | 1.2 (0.8, 1.6) | 42.6 (30.8, 53.9) | 1.0 (0.0, 5.1) |
| Montenegro | 1015 | 6.6 | 1613 (1373, 2074) | 7.5 (6.4, 9.6) | 59.4 (52.3, 68.9) | 2.4 (1.7, 3.4) | 38.1 (29.1, 44.9) | 0.1 (0.0, 0.7) |
| Morocco | 505 | 5.9 | 765 (700, 833) | 5.6 (5.2, 6.1) | 30.5 (26.3, 34.5) | 8.5 (7.2, 10.1) | 60.5 (56.2, 64.7) | 0.6 (0.3, 1.1) |
| Mozambique | 92 | 7.8 | 96 (62, 142) | 5.3 (3.4, 7.8) | 11.4 (4.9, 22.1) | 1.0 (0.6, 1.5) | 15.0 (9.5, 22.3) | 72.6 (58.5, 83.4) |
| Myanmar | 121 | 2.5 | 394 (273, 613) | 3.3 (2.3, 5.1) | 58.8 (43.1, 75.5) | 1.7 (1.0, 2.6) | 32.1 (18.7, 45.4) | 7.4 (2.5, 14.9) |
| Namibia | 936 | 9.3 | 1437 (1277, 1692) | 9.8 (8.7, 11.5) | 57.7 (52.2, 64.7) | 29.7 (24.4, 34.2) | 6.4 (5.2, 7.9) | 6.2 (3.4, 10.2) |
| Nepal | 138 | 5.8 | 226 (197, 259) | 5.6 (4.9, 6.5) | 35.0 (29.5, 41.8) | 6.2 (4.8, 8.1) | 47.5 (40.6, 53.5) | 11.3 (4.3, 19.4) |
| Netherlands | 5234 | 10.7 | 7799 (6370, 9036) | 12.2 (10.0, 14.2) | 89.3 (86.6, 91.2) | 6.0 (4.8, 7.7) | 4.7 (3.7, 6.2) | 0.0 (0.0, 0.0) |
| New Zealand | 4050 | 11 | 5496 (4595, 6193) | 11.4 (9.5, 12.9) | 82.5 (78.8, 85.1) | 7.1 (5.9, 8.8) | 10.4 (8.6, 12.8) | 0.0 (0.0, 0.0) |
| Nicaragua | 450 | 9.1 | 652 (518, 753) | 9.3 (7.4, 10.7) | 53.8 (42.5, 60.9) | 3.9 (3.0, 5.3) | 36.7 (30.5, 46.1) | 5.6 (2.2, 9.6) |
| Niger | 66 | 6.7 | 81 (66, 101) | 6.8 (5.6, 8.5) | 35.9 (26.3, 48.1) | 0.8 (0.6, 1.1) | 47.7 (37.3, 57.2) | 15.6 (8.2, 27.2) |
| Nigeria | 225 | 3.7 | 287 (245, 343) | 3.8 (3.2, 4.5) | 23.4 (15.1, 34.4) | 1.4 (1.1, 1.8) | 68.2 (58.1, 76.5) | 6.9 (3.7, 11.5) |
| Norway | 6537 | 10 | 9758 (8486, 11459) | 11.6 (10.1, 13.6) | 85.6 (83.1, 88.1) | 3.2 (2.6, 4.1) | 11.2 (9.2, 13.3) | 0.0 (0.0, 0.0) |
| Oman | 1467 | 3.5 | 2507 (1908, 4034) | 4.5 (3.4, 7.2) | 93.3 (90.7, 96.1) | 1.9 (1.1, 2.7) | 4.8 (2.7, 7.0) | 0.0 (0.0, 0.0) |
| Pakistan | 132 | 2.7 | 212 (184, 250) | 2.9 (2.6, 3.5) | 41.7 (34.7, 51.1) | 5.6 (4.4, 7.1) | 47.8 (39.6, 54.6) | 4.9 (2.6, 8.1) |
| Panama | 1743 | 8 | 3094 (2659, 3563) | 8.0 (6.9, 9.2) | 73.9 (69.1, 78.7) | 5.2 (4.2, 6.5) | 20.9 (16.6, 25.2) | 0.0 (0.0, 0.0) |
| Papua New Guinea | 108 | 4.4 | 168 (139, 206) | 4.7 (3.9, 5.7) | 71.2 (61.9, 79.1) | 2.0 (1.5, 2.5) | 10.1 (7.6, 13.1) | 16.7 (9.3, 26.2) |

| | 2014 total health spending | | 2030 expected health spending | | 2030 expected health spending by source | | | |
|---|---|---|---|---|---|---|---|---|
| | Per capita ($) | Per GDP (%) | Per capita ($) | Per GDP(%) | Government as a share of total (%) | Prepaid private as a share of total (%) | Out-of-pocket as a share of total (%) | Development assistance as a share of total (%) |
| Paraguay | 863 | 9.8 | 1374 (1146, 1760) | 10.8 (9.0, 13.8) | 51.4 (42.7, 62.5) | 4.3 (3.2, 5.5) | 43.9 (33.8, 52.1) | 0.3 (0.2, 0.6) |
| Peru | 626 | 5.2 | 942 (807, 1158) | 5.3 (4.6, 6.5) | 64.4 (58.2, 71.7) | 6.2 (4.8, 8.1) | 29.2 (22.9, 34.9) | 0.3 (0.0, 0.5) |
| Philippines | 330 | 4.7 | 559 (494, 624) | 5.2 (4.6, 5.8) | 39.7 (32.7, 45.9) | 10.4 (8.8, 12.5) | 48.7 (43.0, 55.0) | 1.1 (0.6, 2.0) |
| Poland | 1629 | 6.3 | 2836 (2528, 3134) | 5.9 (5.3, 6.5) | 71.5 (67.0, 75.9) | 5.7 (4.6, 7.4) | 22.9 (18.6, 26.9) | 0.0 (0.0, 0.0) |
| Portugal | 2697 | 9.3 | 3774 (3110, 4600) | 9.8 (8.1, 12.0) | 65.9 (58.1, 73.0) | 6.9 (5.2, 9.0) | 27.2 (21.1, 34.1) | 0.0 (0.0, 0.0) |
| Qatar | 2663 | 2.2 | 3785 (2922, 5426) | 2.7 (2.1, 3.9) | 88.4 (84.1, 92.4) | 6.4 (4.1, 9.1) | 5.2 (3.1, 8.2) | 0.0 (0.0, 0.0) |
| Romania | 1077 | 5.5 | 2258 (1703, 3063) | 6.8 (5.1, 9.2) | 84.2 (78.8, 89.1) | 0.8 (0.6, 1.1) | 15.0 (10.3, 20.2) | 0.0 (0.0, 0.0) |
| Russia | 1877 | 7.1 | 2287 (2100, 2623) | 7.5 (6.9, 8.6) | 52.9 (46.0, 57.4) | 2.9 (2.3, 3.7) | 44.3 (39.7, 51.4) | 0.0 (0.0, 0.0) |
| Rwanda | 158 | 9.4 | 217 (165, 289) | 8.5 (6.4, 11.3) | 1.0 (0.4, 2.3) | 26.3 (18.7, 34.4) | 26.1 (18.6, 34.3) | 46.6 (31.8, 61.1) |
| Saint Lucia | 755 | 6.7 | 1023 (897, 1212) | 6.8 (6.0, 8.1) | 54.9 (48.7, 62.4) | 0.9 (0.7, 1.2) | 40.5 (33.7, 46.4) | 3.7 (1.3, 7.0) |
| Saint Vincent and the Grenadines | 917 | 8.8 | 1203 (968, 1545) | 8.7 (7.0, 11.2) | 48.9 (38.6, 61.2) | 1.7 (1.2, 2.9) | 46.4 (34.9, 56.4) | 2.9 (1.0, 5.6) |
| Samoa | 365 | 7.2 | 433 (338, 643) | 6.7 (5.2, 9.9) | 84.4 (77.8, 90.6) | 0.8 (0.5, 1.0) | 6.4 (4.0, 8.5) | 8.4 (4.0, 14.6) |
| Sao Tome and Principe | 251 | 7.9 | 317 (241, 416) | 8.1 (6.2, 10.6) | 43.6 (31.0, 56.9) | 6.1 (4.0, 9.2) | 11.5 (7.9, 16.6) | 38.8 (24.5, 54.1) |
| Saudi Arabia | 2320 | 4.4 | 3355 (2554, 5027) | 5.3 (4.0, 8.0) | 80.9 (74.8, 88.0) | 6.0 (3.7, 8.2) | 13.1 (8.1, 18.1) | 0.0 (0.0, 0.0) |
| Senegal | 121 | 5.2 | 153 (130, 184) | 5.3 (4.5, 6.4) | 43.9 (35.7, 52.6) | 1.0 (0.8, 1.4) | 33.3 (26.7, 39.8) | 21.8 (12.9, 32.9) |
| Serbia | 1392 | 10.3 | 1864 (1714, 2037) | 10.4 (9.6, 11.4) | 61.3 (56.5, 65.2) | 0.5 (0.4, 0.7) | 38.1 (34.2, 42.9) | 0.1 (0.0, 0.2) |
| Seychelles | 853 | 3.3 | 1599 (1118, 2226) | 4.0 (2.8, 5.5) | 95.7 (93.2, 97.3) | 2.6 (1.6, 4.7) | 1.7 (1.0, 3.1) | 0.0 (0.0, 0.0) |
| Sierra Leone | 255 | 13.5 | 250 (214, 311) | 15.7 (13.4, 19.5) | 6.4 (4.3, 8.8) | 8.7 (6.5, 10.8) | 48.3 (38.3, 57.5) | 36.6 (28.2, 48.8) |
| Singapore | 3981 | 4.8 | 6990 (5335, 9135) | 6.0 (4.6, 7.9) | 50.7 (37.0, 62.9) | 1.8 (1.3, 2.6) | 47.5 (35.7, 60.7) | 0.0 (0.0, 0.0) |
| Slovakia | 2203 | 7.7 | 3798 (3306, 4375) | 8.0 (7.0, 9.2) | 76.5 (71.8, 81.3) | 0.6 (0.5, 0.8) | 22.8 (18.1, 27.5) | 0.0 (0.0, 0.0) |
| Slovenia | 2845 | 9.1 | 3970 (3482, 4776) | 9.4 (8.2, 11.3) | 72.9 (68.7, 77.7) | 15.5 (12.5, 18.8) | 11.7 (9.4, 13.7) | 0.0 (0.0, 0.0) |
| Solomon Islands | 107 | 5.8 | 111 (75, 157) | 4.9 (3.3, 7.0) | 52.4 (34.7, 69.4) | 1.1 (0.7, 1.6) | 4.8 (3.2, 7.2) | 41.7 (24.7, 59.8) |
| Somalia | 33 | 6.9 | 36 (27, 50) | 6.9 (5.2, 9.5) | 25.1 (16.4, 33.4) | 1.1 (0.7, 1.7) | 28.9 (20.2, 37.5) | 44.9 (30.2, 61.1) |
| South Africa | 1172 | 8.9 | 1499 (1346, 1684) | 9.7 (8.7, 10.9) | 51.4 (45.9, 56.9) | 40.4 (35.7, 45.5) | 5.6 (4.6, 6.8) | 2.5 (1.2, 4.5) |

| | 2014 total health spending | | 2030 expected health spending | | 2030 expected health spending by source | | | |
|---|---|---|---|---|---|---|---|---|
| | Per capita ($) | Per GDP (%) | Per capita ($) | Per GDP(%) | Government as a share of total (%) | Prepaid private as a share of total (%) | Out-of-pocket as a share of total (%) | Development assistance as a share of total (%) |
| South Korea | 2507 | 7.1 | 4838 (4088, 5783) | 9.0 (7.6, 10.8) | 63.3 (56.4, 70.2) | 6.2 (4.8, 8.2) | 30.4 (24.5, 36.6) | 0.0 (0.0, 0.0) |
| South Sudan | 94 | 3.6 | 120 (84, 182) | 5.1 (3.6, 7.7) | 25.9 (13.1, 48.1) | 1.0 (0.6, 1.5) | 28.2 (17.7, 39.2) | 44.9 (26.1, 63.5) |
| Spain | 3096 | 9 | 4245 (3808, 4645) | 9.0 (8.0, 9.8) | 71.0 (66.1, 75.0) | 4.6 (3.9, 6.1) | 24.4 (20.4, 29.2) | 0.0 (0.0, 0.0) |
| Sri Lanka | 402 | 3.5 | 911 (716, 1180) | 3.8 (3.0, 5.0) | 59.1 (48.8, 69.3) | 1.8 (1.2, 2.5) | 38.8 (29.2, 48.7) | 0.3 (0.0, 1.5) |
| Sudan | 334 | 8.3 | 457 (380, 543) | 8.0 (6.6, 9.5) | 22.3 (15.8, 29.3) | 1.0 (0.7, 1.3) | 74.9 (67.7, 81.6) | 1.8 (0.9, 3.1) |
| Suriname | 731 | 4.3 | 940 (765, 1171) | 4.2 (3.4, 5.2) | 67.8 (60.0, 75.1) | 16.3 (12.2, 21.1) | 15.3 (11.3, 20.2) | 0.5 (0.0, 2.3) |
| Swaziland | 745 | 9.5 | 1132 (923, 1430) | 11.5 (9.4, 14.5) | 69.0 (60.0, 77.4) | 6.8 (5.1, 8.8) | 8.6 (6.4, 11.2) | 15.6 (8.5, 24.6) |
| Sweden | 5446 | 11.8 | 8048 (6984, 9231) | 13.1 (11.4, 15.0) | 86.2 (83.4, 88.6) | 0.6 (0.5, 0.8) | 13.1 (10.8, 15.9) | 0.0 (0.0, 0.0) |
| Switzerland | 7831 | 12.8 | 9702 (8612, 10687) | 13.4 (11.9, 14.7) | 65.3 (60.7, 69.0) | 10.6 (9.3, 12.2) | 24.0 (21.1, 27.9) | 0.0 (0.0, 0.0) |
| Syria | 562 | 3.4 | 736 (618, 908) | 3.7 (3.1, 4.5) | 49.2 (41.0, 59.4) | 3.3 (2.4, 4.3) | 47.0 (37.2, 55.0) | 0.6 (0.2, 1.1) |
| Tajikistan | 200 | 7.3 | 309 (266, 362) | 8.9 (7.7, 10.5) | 34.3 (26.9, 43.2) | 5.3 (3.4, 9.5) | 52.1 (44.2, 59.4) | 8.3 (4.5, 13.6) |
| Tanzania | 166 | 6.4 | 239 (194, 303) | 6.2 (5.0, 7.8) | 29.1 (20.8, 42.0) | 22.7 (16.8, 28.6) | 20.4 (15.3, 25.7) | 27.8 (16.8, 41.0) |
| Thailand | 633 | 4.1 | 1113 (861, 1390) | 4.3 (3.4, 5.4) | 80.7 (74.7, 85.4) | 9.1 (6.8, 12.3) | 10.3 (7.2, 13.9) | 0.0 (0.0, 0.4) |
| The Bahamas | 1996 | 7.7 | 2658 (2387, 3054) | 8.6 (7.7, 9.8) | 48.4 (42.7, 55.1) | 23.8 (20.2, 27.6) | 27.8 (23.4, 33.1) | 0.0 (0.0, 0.0) |
| The Gambia | 151 | 9.2 | 174 (138, 228) | 10.2 (8.1, 13.4) | 48.1 (35.7, 59.3) | 0.5 (0.4, 0.7) | 12.7 (9.1, 16.7) | 38.7 (25.4, 54.0) |
| Timor-Leste | 105 | 1.9 | 216 (139, 329) | 3.0 (2.0, 4.6) | 56.9 (34.8, 74.9) | 1.7 (1.1, 2.6) | 5.6 (3.1, 9.5) | 35.8 (18.5, 58.3) |
| Togo | 81 | 5.5 | 114 (99, 134) | 6.1 (5.2, 7.1) | 38.0 (30.6, 46.9) | 7.2 (5.9, 8.8) | 41.1 (34.3, 47.5) | 13.6 (7.7, 21.4) |
| Tonga | 253 | 5.3 | 399 (279, 594) | 6.4 (4.5, 9.5) | 72.9 (59.8, 84.2) | 0.8 (0.5, 1.3) | 9.5 (6.0, 13.5) | 16.7 (7.7, 29.0) |
| Trinidad and Tobago | 1823 | 5.8 | 2518 (2216, 2919) | 6.3 (5.5, 7.3) | 55.1 (48.5, 62.0) | 7.0 (5.7, 8.7) | 37.8 (31.6, 44.5) | 0.0 (0.0, 0.0) |
| Tunisia | 791 | 6.9 | 1099 (992, 1232) | 7.2 (6.5, 8.1) | 59.8 (55.0, 64.6) | 4.5 (3.7, 5.5) | 35.6 (31.1, 40.4) | 0.1 (0.1, 0.2) |
| Turkey | 1040 | 5.3 | 1748 (1556, 2032) | 5.7 (5.1, 6.6) | 79.1 (75.9, 82.5) | 3.0 (2.5, 3.7) | 17.9 (14.9, 20.8) | 0.0 (0.0, 0.0) |
| Turkmenistan | 396 | 2.3 | 925 (763, 1132) | 2.7 (2.2, 3.3) | 64.4 (56.6, 71.7) | 6.7 (5.2, 8.5) | 28.8 (22.5, 35.8) | 0.0 (0.0, 0.0) |
| Uganda | 347 | 18.1 | 313 (262, 370) | 11.6 (9.7, 13.7) | 2.9 (1.3, 5.0) | 51.7 (42.8, 59.5) | 25.0 (20.5, 30.1) | 20.4 (12.1, 30.8) |
| Ukraine | 659 | 7 | 673 (584, 781) | 7.5 (6.5, 8.7) | 49.4 (41.3, 55.2) | 0.9 (0.7, 1.4) | 48.1 (42.3, 56.3) | 1.5 (0.4, 3.7) |

| | 2014 total health spending | | 2030 expected health spending | | 2030 expected health spending by source | | | |
|---|---|---|---|---|---|---|---|---|
| | Per capita ($) | Per GDP (%) | Per capita ($) | Per GDP(%) | Government as a share of total (%) | Prepaid private as a share of total (%) | Out-of-pocket as a share of total (%) | Development assistance as a share of total (%) |
| United Arab Emirates | 2561 | 3.6 | 3290 (2724, 4287) | 4.2 (3.4, 5.4) | 74.4 (68.4, 81.2) | 9.3 (6.6, 12.2) | 16.3 (11.7, 21.3) | 0.0 (0.0, 0.0) |
| United Kingdom | 3749 | 9.1 | 5002 (4276, 5803) | 9.3 (7.9, 10.8) | 83.1 (80.0, 85.8) | 7.3 (6.0, 8.7) | 9.7 (7.9, 11.9) | 0.0 (0.0, 0.0) |
| United States | 9237 | 16.6 | 12448 (11293, 13528) | 17.7 (16.0, 19.2) | 50.7 (45.7, 54.9) | 38.7 (35.1, 43.0) | 10.6 (9.3, 12.1) | 0.0 (0.0, 0.0) |
| Uruguay | 1837 | 8.6 | 2766 (2289, 3130) | 8.9 (7.4, 10.1) | 73.3 (67.5, 77.3) | 12.7 (10.5, 16.0) | 14.0 (11.3, 17.5) | 0.0 (0.0, 0.0) |
| Uzbekistan | 397 | 5.9 | 802 (648, 1024) | 7.2 (5.8, 9.2) | 63.7 (55.3, 72.6) | 1.8 (1.3, 2.3) | 33.6 (25.2, 41.6) | 0.9 (0.4, 1.7) |
| Vanuatu | 149 | 5.4 | 214 (145, 331) | 7.3 (5.0, 11.3) | 64.8 (47.3, 81.0) | 0.8 (0.5, 1.2) | 4.2 (2.5, 6.1) | 30.3 (15.0, 48.0) |
| Venezuela | 1010 | 5.3 | 1125 (988, 1277) | 5.7 (5.0, 6.5) | 33.6 (27.0, 40.8) | 6.3 (5.2, 8.0) | 60.0 (53.2, 66.4) | 0.0 (0.0, 0.0) |
| Vietnam | 398 | 7 | 919 (740, 1123) | 7.6 (6.1, 9.2) | 62.8 (54.0, 71.2) | 5.8 (4.6, 7.3) | 30.4 (22.8, 38.3) | 1.0 (0.3, 2.2) |
| Yemen | 233 | 5.8 | 229 (179, 299) | 7.0 (5.5, 9.1) | 12.7 (6.2, 21.0) | 1.4 (1.0, 1.9) | 69.9 (56.3, 81.0) | 16.0 (7.6, 30.0) |
| Zambia | 216 | 5.4 | 287 (232, 363) | 5.6 (4.5, 7.1) | 40.5 (30.8, 50.6) | 1.1 (0.7, 1.5) | 27.2 (20.7, 34.2) | 31.2 (19.1, 45.2) |

## FRONTIER ANALYSIS

Frontier analysis is an econometric method for determining the efficiency with which a country (or other unit) produces an output. By benchmarking the country's performance against the observed performance of others, the frontier describes the maximum potential output that one could achieve. In the present study, we used frontier analysis in two ways: 1) to describe the potential total health spending a country could achieve given their level of GDP per capita, and 2) to describe the potential government health spending a country could achieve under different policy scenarios. We provide a review of frontier methods we considered for this study and a more detailed explanation of how the results were calculated.

### Approaches to frontier analysis

Methods for calculating a frontier generally fall into two categories: stochastic frontier analysis and data envelopment analysis.[29] Stochastic frontier analysis (SFA) can be understood intuitively as a modification to ordinary least squares regression. The frontier is defined by a set of parameters $\beta_0 \ldots \beta_n$, generally following the form

$$y_i = \beta_0 + \sum_n \beta_n x_{ni} + v_i - u_i$$

where $y$ is the output of interest, $x$ is an input, $v$ represents random error, and $u$ represents inefficiency. The random error term $v_i$ has the same interpretation as the error term in OLS regression and is assumed to be normally distributed. The inefficiency term $u_i$ describes the difference between each point and its efficient maximum, and may follow any distribution bounded by zero. Together, $v_i$ minus $u_i$ forms a compound error term that describes the departure of each point from the frontier. The inclusion of $-u_i$ pushes the fitted line higher on the y-axis, such that it describes a frontier rather than the central tendency of the data. Note that the analogy with ordinary least squares regression only applies to linear models; non-linear models require maximum likelihood estimation.

Data envelopment analysis (DEA) is a non-parametric alternative to SFA. An advantage of DEA is that it does not require a researcher to specify the functional form that relates the variables to each other (e.g., linear, Cobb-Douglas). It uses a deterministic algorithm, and does not attempt to describe the data using a probability distribution. Examples of different DEA algorithms are shown in Figure 8.[29] The main limitation of DEA is that, because it is non-parametric, it does not account for stochastic variation. Points high on the y-axis are assumed to represent a high level of the output, not random noise.

Figure 8. Comparison of DEA methods

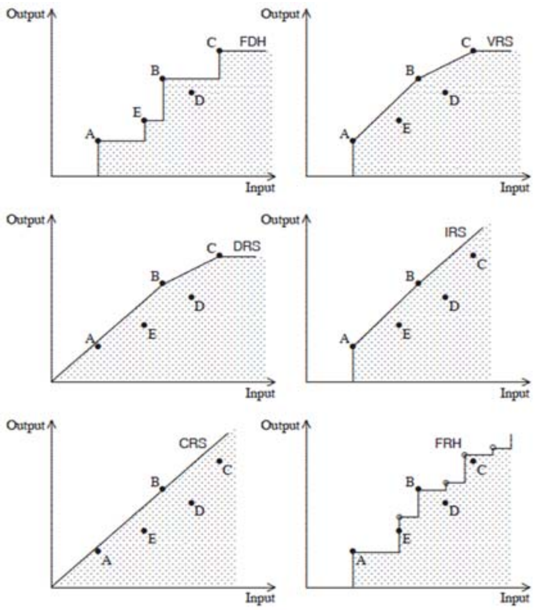


Source: Bogetoft and Otto (2011), “Benchmarking with DEA, SFA, and R”

*Key*

***FDH** – Free Disposability Hull assumes unnecessary input and unwanted outputs can be discarded. It does not assume that the hull is convex; **VRS** – Variable Returns to Scale assumes variable returns as inputs increase in scale, and the hull is convex; **DRS** – Decreasing Returns to Scale assumes decreasing returns as inputs increase in scale, and the hull is convex; **IRS** – Increasing Returns to Scale assumes increasing returns as inputs increase in scale, and the hull is convex; **CRS** – Constant Returns to Scale assumes constant returns as inputs increase in scale, and the hull is convex; **FRH** – Free Replicability Hull is used when peer units have disparate input combinations and it does not assume that the hull is convex*

For the present analysis, we used stochastic frontier analysis with the inefficiency term $u_i$ following a half-normal distribution. DEA was not suitable because the assumption of no stochastic variation does not hold for our data. The measures of interest, gross domestic product and health expenditure, inherently contain a degree of measurement error. Moreover, the relationship between our variables is linear, which is captured adequately with a linear functional form of SFA. Regarding the distribution of the inefficiency term, we considered using the truncated normal distribution because of its relative flexibility. It estimates two parameters (mean and variance) rather than one, as is the case with the half-normal and exponential distributions. The half-normal distribution is merely a special case of truncated normal in which the mean is zero. For each frontier, we ran models with truncated normal and found that the mean of the inefficiency term was not statistically different from zero. Because the extra flexibility of the truncated normal distribution was not needed, we proceeded with the half-normal distribution.

## Analyses

To estimate a country's potential increase in total health expenditure, we fit a frontier with log-scale gross domestic product per capita (GDP) as the input and log-scale total health expenditure per capita as the output. The potential increase in total health expenditure is defined as the difference between each point and the frontier (after exponentiating both values). By using this approach, we assert that a below-average country could spend as much as an average country at its level of GDP, even in the absence of inefficiency. The "frontier" package in R is used to estimate the frontier.[30]

To estimate a country's potential increase in government health expenditure under different policy scenarios, we fit two frontiers. Figure 9 shows an example of these frontiers for India. First, we used GDP as the input and general government expenditure (GGE) as the output. The difference between each point and the frontier is the potential increase in GGE at the country's level of GDP (Plot 1, red line). For the second frontier, we used GGE as the input and government health expenditure (GHE) as the output. The difference between each point and the frontier is the potential increase in GHE at the country's level of general government expenditure (Plot 2, blue line). All differences were taken after exponentiating the values.

**Figure 9.** Potential government health expenditure; policy scenarios for India (illustrative example)

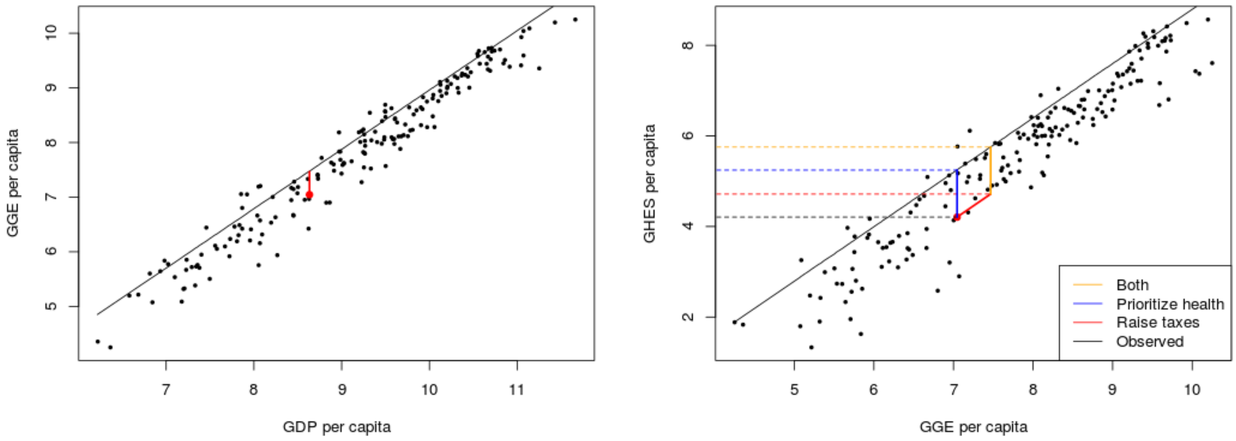


Plot 1 Plot 2

Together, these two frontiers can be used to model three policy scenarios and their effect on government health expenditure:

1) Potential increase due to prioritizing the health sector, calculated as the potential GHE at a country's current level of general government expenditure (Plot 2, blue line).
2) Potential increase due to increasing general government expenditure, calculated as the expected level of GHE at a country's potential level of general government expenditure (Plot 2, red line).
3) Potential increase due to both prioritizing the health sector and increasing general government expenditure, calculated as the potential GHE at a country's potential level of general government expenditure (Plot 2, red line and orange line combined).

## Robustness analysis

To examine the robustness of our results, we tested SFA with different distributional assumptions for the inefficiency term. The results are shown in Figure 10. The half-normal and truncated normal models produced similar frontiers. This result is consistent with Wald tests that assess whether the mean of the truncated normal efficiency distribution is equal to zero. None of the tests showed a statistically significant difference. With a mean of zero, the truncated normal model becomes half-normal, which is what we used in the final analysis.

**Figure 10.** Comparison of frontier methods

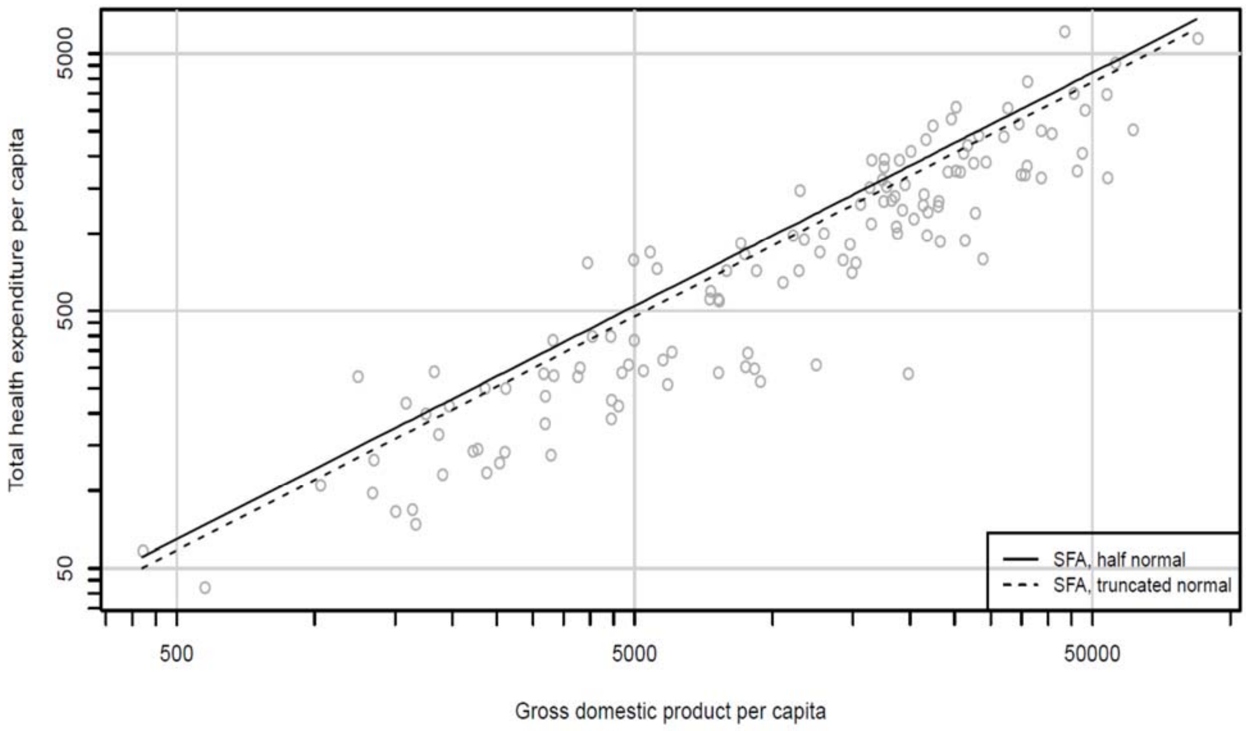

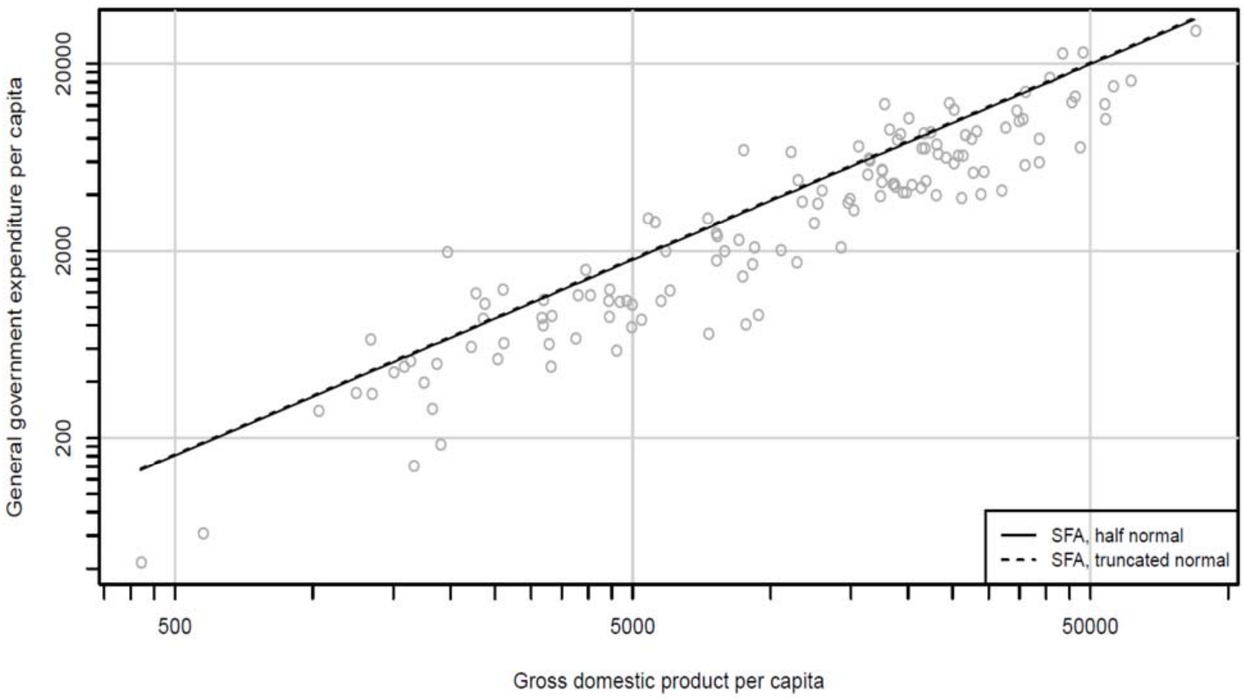


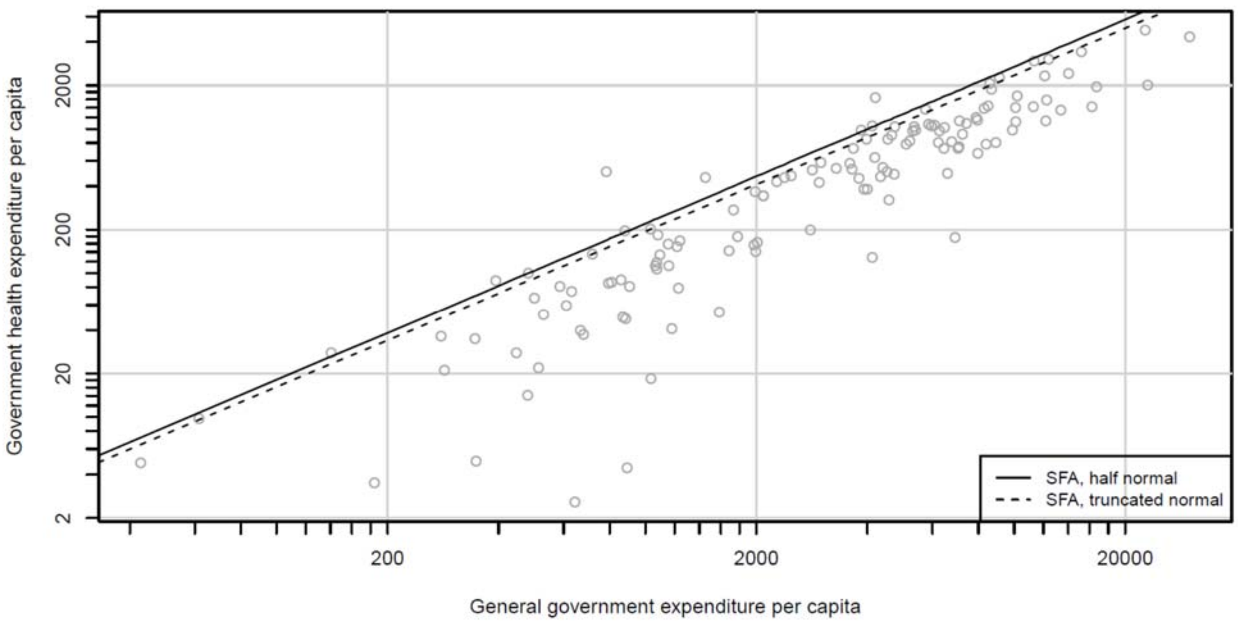


Additionally, we include figures that highlight the results of the policy simulation analysis for each low- and middle-income country. See figures after References.

**Author List:**

Joseph Dieleman; Madeline Campbell; Abigail Chapin; Erika Eldrenkamp; Victoria Y. Fan; Annie Haakenstad; Jennifer Kates; Taylor Matyasz; Angela Micah; Alex Reynolds; Nafis Sadat; Reed Sorensen; Kaja M Abbas; Semaw Ferede Abera; Aliasghar Ahmad Kiadaliri; Muktar Beshir Ahmed; Khurshid Alam; Reza Alizadeh-Navaei; Ala'a Alkerwi; Erfan Amini; Walid Ammar; Carl Abelardo T Antonio; Tesfay Mehari Atey; Leticia Avila-Burgos; Ashish Awasthi; Aleksandra Barac; Tezera Moshago Berheto; Addisu Shunu Beyene; Tariku Jibat Beyene; Charles Birungi; Habtamu Mellie Bizuayehu; Nicholas J. K. Breitborde; Lucero Cahuana-Hurtado; Ruben Estanislao Castro; Ferran Catalia-Lopez; Koustuv Dalal; Samath D. Dharmaratne; Manisha Dubey; AndrÃ© Faro; Andrea B Feigl; Florian Fischer; Joseph Robert Anderson Fitchett; Nataliya Foigt; Ababi Zergaw Giref; Rahul Gupta; Samer Hamidi; Hilda L Harb; Simon I. Hay; Delia Hendrie; Masako Horino; Mikk Jürisson; Mihajlo B Jakovljevic; Mehdi Javanbakht; Denny John; Jost B Jonas; Seyed M. Karimi; Young-Ho Khang; Jagdish Khubchandani; Yun Jin Kim; Jonas M. Kinge; Kristopher J. Krohn; Ricky Leung; Zhiyin Li; Hassan Magdy Abd El Razek; Mohammed Magdy Abd El Razek; Azeem Majeed; Reza Malekzadeh; Deborah Carvalho Malta; Atte Meretoja; Ted R Miller; Erkin M Mirrakhimov; Shafiu Mohammed; Gedefaw Molla; Vinay Nangia; Stefano Olgiati; Mayowa O Owolabi; Tejas Patel; Angel J Paternina Caicedo; David M Pereira; Julian Perelman; Suzanne Polinder; Anwar Rafay; Vafa Rahimi-Movaghar; Rajesh Kumar Rai; Usha Ram; Chhabi Lal Ranabhat; Hirbo Shore Roba; Miloje Savic; Matthew T Schneider; Sadaf G Sepanlou; Braden J Te Ao; Azeb Gebresilassie Tesema; Alan J Thomson; Ruoyan Tobe-Gai; Roman Topor-Madry; Eduardo A Undurraga; Veronica Vargas; Tommi Vasankari; Francesco S. Violante; Tissa Wijeratne; Gelin Xu; Naohiro Yonemoto; Mustafa Z Younis; Chuanhua Yu; Zoubida Zaidi; Maysaa El Sayed Zaki; Christopher J L Murray.

**Affiliations List:**

Institute for Health Metrics and Evaluation, University of Washington, Seattle, WA, USA (Prof J Dieleman PhD, M Campbell BS, A Chapin BA, E Eldrenkamp BA, A Haakenstad MA, Z Li MPS, T Matyasz MS, A Micah PhD, A Reynolds BA, N Sadat MA, M T Schneider MPH, R Sorensen MPH, Prof S I Hay DSc, K J Krohn BA, Prof C J L Murray DPhil); University of Hawaii at Manoa, Honolulu, HI, USA (V Y Fan ScD); François-Xavier Bagnoud Center for Health and Human Rights (V Y Fan ScD), Harvard University, Boston, MA, USA (J R A Fitchett MD); Center for Global Development, Washington, DC, USA (V Y Fan ScD); Kaiser Family Foundation, Washington, DC, USA (J Kates PhD); Virginia Tech, Blacksburg, VA, USA (Prof K M Abbas PhD); School of Public Health, College of Health Sciences (S F Abera MSc), Mekelle University, Mekelle, Ethiopia (T M Atey MS, A G Tesema MPH); Food Security and Institute for Biological Chemistry and Nutrition, University of Hohenheim, Stuttgart, Germany (S F Abera MSc); Department of Clinical Sciences Lund, Orthopedics, Clinical Epidemiology Unit, Lund University, Lund, Sweden (A Ahmad Kiadaliri PhD); College of Health Sciences, Department of Epidemiology, ICT and e-Learning Coordinator, Jimma University, Jimma, Ethiopia (M B Ahmed MPH); Murdoch Childrens Research Institute, The University of Melbourne, Melbourne, VIC, Australia (K Alam PhD); The University of Sydney, Sydney, NSW, Australia (K Alam PhD); Gastrointestinal Cancer Research Center, Mazandaran University of Medical Sciences, Sari, Iran (R Alizadeh-Navaei PhD); Luxembourg Institute of Health (LIH), Strassen, Luxembourg (A Alkerwi PhD); Uro-Oncology Research Center (E Amini MD), Non-communicable Diseases Research Center, Endocrinology and Metabolism Research Institute (E Amini MD), Digestive Diseases Research Institute (Prof R Malekzadeh MD, S G Sepanlou PhD), Sina Trauma and Surgery Research Center (Prof V Rahimi-Movaghar MD), Tehran University of Medical Sciences, Tehran, Iran; Ministry of Public Health, Beirut, Lebanon (W Ammar PhD, H L Harb MPH); Department of Health Policy and

Administration, College of Public Health, University of the Philippines Manila, Manila, Philippines (C A T Antonio MD); National Institute of Public Health, Cuernavaca, Mexico (L Avila-Burgos PhD); Sanjay Gandhi Postgraduate Institute of Medical Sciences, Lucknow, India (A Awasthi MSc); Faculty of Medicine, University of Belgrade, Belgrade, Serbia (A Barac PhD); College of Health Sciences, School of Public Health, Wolaita Sodo University, Wolaita Sodo, Ethiopia (T M Berheto MPH); College of Health and Medical Science (A S Beyene MPH), College of Health and Medical Sciences (H S Roba MPH), Haramaya University, Harar, Ethiopia; Addis Ababa University, Addis Ababa, Ethiopia (T J Beyene MS, A Z Giref PhD); Wageningen University, Wageningen, Netherlands (T J Beyene MS); University College London, London, UK (C Birungi MS); Debre Markos University, Debre Markos, Ethiopia (H M Bizuayehu MPH); Ohio State University, Columbus, OH, USA (Prof N J K Breitborde PhD); National Institute of Public Health, Cuernavaca, Mexico (L Cahuana-Hurtado PhD); Universidad Diego Portales, Santiago, Chile (R E Castro PhD); Department of Medicine, University of Valencia/INCLIVA Health Research Institute and CIBERSAM, Valencia, Spain (F Catalá-López PhD); Clinical Epidemiology Program, Ottawa Hospital Research Institute, Ottawa, ON, Canada (F Catalá-López PhD); Centre for Injury Prevention and Safety Promotion, School of Health and Medical Sciences, Orebro University, Orebro, Sweden (Prof K Dalal PhD); Department of Community Medicine, Faculty of Medicine, University of Peradeniya, Peradeniya, Sri Lanka (S D Dharmaratne MD); International Institute for Population Sciences, Mumbai, India (M Dubey MPhil, Prof U Ram PhD); Federal University of Sergipe, Aracaju, Brazil (Prof A Faro PhD); Department of Global Health and Population, TH Chan School of Public Health (A B Feigl ScD), Harvard University, Boston, MA, USA (J R A Fitchett MD); School of Public Health, Bielefeld University, Bielefeld, Germany (F Fischer PhD); Institute of Gerontology, Academy of Medical Science, Kyiv, Ukraine (N Foigt PhD); West Virginia Bureau for Public Health, Charleston, WV, USA (R Gupta MD); Hamdan Bin Mohammed Smart University, Dubai, United Arab Emirates (S Hamidi DrPH); Oxford Big Data Institute, Li Ka Shing Centre for Health Information and Discovery, University of Oxford, Oxford, UK (Prof S I Hay DSc); Centre for Population Health Research (D Hendrie MA), Curtin University, Bentley, WA, Australia; Department of Health and Human Services, Nevada Division of Public and Behavioral Health, Carson City, NV, USA (M Horino MPH); Institute of Family Medicine and Public Health, University of Tartu, Tartu, Estonia (M Jürisson MD); Faculty of Medical Sciences, University of Kragujevac, Kragujevac, Serbia (Prof M B Jakovljevic PhD); University of Aberdeen, Aberdeen, UK (M Javanbakht PhD); International Center for Research on Women, New Delhi, India (D John MPH); Department of Ophthalmology, Medical Faculty Mannheim, Ruprecht-Karls-University Heidelberg, Mannheim, Germany (Prof J B Jonas MD); University of Washington Tacoma, Tacoma, WA, USA (S M Karimi PhD); College of Medicine, Seoul National University, Seoul, South Korea (Prof Y Khang MD); Ball State University, Muncie, IN, USA (J Khubchandani PhD); Southern University College, Skudai, Malaysia (Y J Kim PhD); Norwegian Institute of Public Health, Oslo, Norway (J M Kinge PhD, M Savic PhD); State University of New York, Albany, Rensselaer, NY, USA (R Leung PhD); Mansoura Faculty of Medicine, Mansoura, Egypt (H Magdy Abd El Razek MBBCH); Aswan University Hospital, Aswan Faculty of Medicine, Aswan, Egypt (M Magdy Abd El Razek MBBCh); Imperial College London, London, UK (Prof A Majeed MD); Universidade Federal de Minas Gerais, Belo Horizonte, Brazil (Prof D C Malta PhD); Department of Medicine (A Meretoja PhD), University of Melbourne, Melbourne, VIC, Australia (Prof T Wijeratne MD); Department of Neurology, Helsinki University Hospital, Helsinki, Finland (A Meretoja PhD); Pacific Institute for Research & Evaluation, Calverton, MD, USA (T R Miller PhD); Centre for Population Health (T R Miller PhD), Curtin University, Perth, WA, Australia; Kyrgyz State Medical Academy, Bishkek, Kyrgyzstan (Prof E M Mirrakhimov PhD); National Center of Cardiology and Internal Disease, Bishkek, Kyrgyzstan (Prof E M

Mirrakhimov PhD); Health Systems and Policy Research Unit, Ahmadu Bello University, Zaria, Nigeria (S Mohammed PhD); Institute of Public Health, Heidelberg University, Heidelberg, Germany (S Mohammed PhD); Federal Ministry of Health, Addis Ababa, Ethiopia (G Molla MD); Suraj Eye Institute, Nagpur, India (V Nangia MD); State University of Bergamo, Bergamo, Italy (S Olgiati PhD); Department of Medicine, Ibadan, Nigeria (M O Owolabi Dr Med); Blossom Specialist Medical Center, Ibadan, Nigeria (M O Owolabi Dr Med); Mount Sinai Health System, New York, NY, USA (T Patel MD); Universidad de Cartagena, Cartagena, Colombia (A J Paternina Caicedo MD); Public Health Dynamics Laboratory, University of Pittsburgh, Pittsburgh, PA, USA (A J Paternina Caicedo MD); REQUIMTE/LAQV, Laboratório de Farmacognosia, Departamento de Química, Faculdade de Farmácia, Universidade do Porto, Porto, Portugal (Prof D M Pereira PhD); National School of Public Health, Lisbon, Portugal (Prof J Perelman PhD); Department of Public Health, Erasmus MC, University Medical Center Rotterdam, Rotterdam, Netherlands (S Polinder PhD); Contech International Health Consultants, Lahore, Pakistan (A Rafay MS); Contech School of Public Health, Lahore, Pakistan (A Rafay MS); Society for Health and Demographic Surveillance, Suri, India (R K Rai MPH); Wonju College of Medicine (C L Ranabhat PhD), Yonsei University, Wonju, South Korea; Institute for Poverty Alleviation and International Development (C L Ranabhat PhD), Yonsei University, Seoul, South Korea; Auckland University of Technology, Auckland, New Zealand (B J Te Ao MPH); Adaptive Knowledge Management, Victoria, BC, Canada (A J Thomson PhD); National Center for Child Health and Development, Tokyo, Japan (R Tobe-Gai PhD); Institute of Public Health, Faculty of Health Sciences, Jagiellonian University Medical College, Krakow, Poland (R Topor-Madry PhD); Faculty of Health Sciences, Wroclaw Medical University, Wroclaw, Poland (R Topor-Madry PhD); Brandeis University, Waltham, MA, USA (E A Undurraga PhD); Universidad Alberto Hurtado, Santiago, Chile (V Vargas PhD); UKK Institute for Health Promotion Research, Tampere, Finland (Prof T Vasankari PhD); University of Bologna, Bologna, Italy (Prof F S Violante MD); Western Health, Footscray, VIC, Australia (Prof T Wijeratne MD); Department of Neurology, Jinling Hospital, Nanjing University School of Medicine, Nanjing, China (Prof G Xu PhD); Department of Biostatistics, School of Public Health, Kyoto University, Kyoto, Japan (N Yonemoto MPH); Jackson State University, Jackson, MS, USA (Prof M Z Younis DrPH); Department of Epidemiology and Biostatistics, School of Public Health (Prof C Yu PhD), Global Health Institute (Prof C Yu PhD), Wuhan University, Wuhan, China; University Hospital, Setif, Algeria (Prof Z Zaidi PhD); Faculty of Medicine, Mansoura University, Mansoura, Egypt (Prof M E Zaki PhD);

# REFERENCES


1 Dieleman JL, Schneider MT, Haakenstad A, *et al.* Development assistance for health: past trends, associations, and the future of international financial flows for health. *The Lancet* 2016; **387**: 2536–44.

2 Honaker J, King G. What to do about missing values in time-series cross-section data. *Am J Polit Sci*; **54**: 561–81.

3 James SL, Gubbins P, Murray CJ, Gakidou E. Developing a comprehensive time series of GDP per capita for 210 countries from 1950 to 2015. *Popul Health Metr* 2012; **10**: 12.

4 Honaker J, King G, Blackwell M. Amelia II: A program for missing data. *J Stat Softw*; **45**: 1–47.

5 Dieleman JL, Templin T, Sadat N, *et al.* National spending on health by source for 184 countries between 2013 and 2040. *The Lancet* 2016; **387**: 2521–35.

6 Hoeting JA, Madigan D, Raftery AE, Volinsky CT. Bayesian model averaging: a tutorial. *Stat Sci* 1999; **14**: 382–401.

7 Foreman KJ, Lozano R, Lopez AD, Murray CJ. Modeling causes of death: an integrated approach using CODEm. *Popul Health Metr* 2012; **10**: 1.

8 Duan Q, Ajami NK, Gao X, Sorooshian S. Multi-model ensemble hydrologic prediction using Bayesian model averaging. *Adv Water Resour* 2007; **30**: 1371–86.

9 Krishnamurti TN, Kishtawal CM, Zhang Z, *et al.* Multimodel ensemble forecasts for weather and seasonal climate. *J Clim* 2000; **13**: 4196–216.

10 Hanlon M, Graves CM, Brooks BP, *et al.* Regional variation in the allocation of development assistance for health. *Glob Health* 2014; **10**: 8.

11 Fan VY, Savedoff WD. The health financing transition: a conceptual framework and empirical evidence. *Soc Sci Med* 2014; **105**: 112–21.

12 Hansen BE. Rethinking the univariate approach to unit root testing: using covariates to increase power. *Econom Theory* 1995; **11**: 1148–71.

13 Levin A, Lin C, Chu CJ. Unit root tests in panel data: Asymptotic and finite-sample properties. *J Econom* 2002; **108**: 1–24.

14 Im KS, Pesaran MH, Shin Y. Testing for unit roots in heterogeneous panels. *J Econom* 2003; **115**: 53–74.

15 Hadri K. Testing for stationarity in heterogeneous panel data. *Econom J*; **3**: 148–61.

16 Lupi C. Unit root CADF testing with R. *J Stat Softw*; **32**: 1–19.

17 Croissant Y, Millo G. Panel data econometrics in R: the plm package. *J Stat Softw* 2008; **27**. http://www.jstatsoft.org/v27/i02/.

18 Sklar A. Fonctions de répartition à n dimensions et leurs marges. *Publ Inst Stat Univ Paris Fr*; : 229–31.

19 Embrechts P, McNeil A, Straumann D. Correlation and Dependence in Risk Management: Properties and Pitfalls. 1999. http://citeseerx.ist.psu.edu/viewdoc/download?doi=10.1.1.73.6352&rep=rep1&type=pdf.

20 Schepsmeier U, Stoeber J, Brechmann EC, Graeler B, Nagler T, Erhardt T. VineCopula: statistical inference of vine copulas. 2016 https://CRAN.R-project.org/package=VineCopula.

21 Pitt MM, Lee L-F. The measurement and sources of technical inefficiency in the Indonesian weaving industry. *J Dev Econ* 1981; **9**: 43–64.

22 R Core Team. R: A language and environment for statistical computing. Vienna, Austria: R Foundation for Statistical Computing, 2016 https://www.R-project.org/.

23 Wickham H, Francois R. dplyr: a grammar of data manipulation. 2016 https://CRAN.R-project.org/package=dplyr.

24 Wickham H. ggplot2: elegant graphics for data analysis. New York: Springer-Verlag, 2009.

25 Urbanek S, Horner J. Cairo: R graphics device using cairo graphics library for creating high-quality bitmap (PNG, JPEG, TIFF), vector (PDF, SVG, PostScript) and display (X11 and Win32) output. 2015 https://CRAN.R-project.org/package=Cairo.

26 StataCorp. Stata statistical software: release 13. College Station, TX: StataCorp LP.

27 Garbuszus JM, Jeworutzki S. readstata13: import ‘Stata’ data files. 2016 https://CRAN.R-project.org/package=readstata13.

28 Dowle M, Srinivasan A. data.table: extension of ‘data.frame’. 2016 https://CRAN.R-project.org/package=data.table.

29 Bogetoft P, Otto L. Benchmarking with DEA, SFA, and R. Springer New York, 2011.

30 Coelli T, Henningsen A. frontier: stochastic frontier analysis. 2013 http://CRAN.R-Project.org/package=frontier.

# Country policy scenario figures

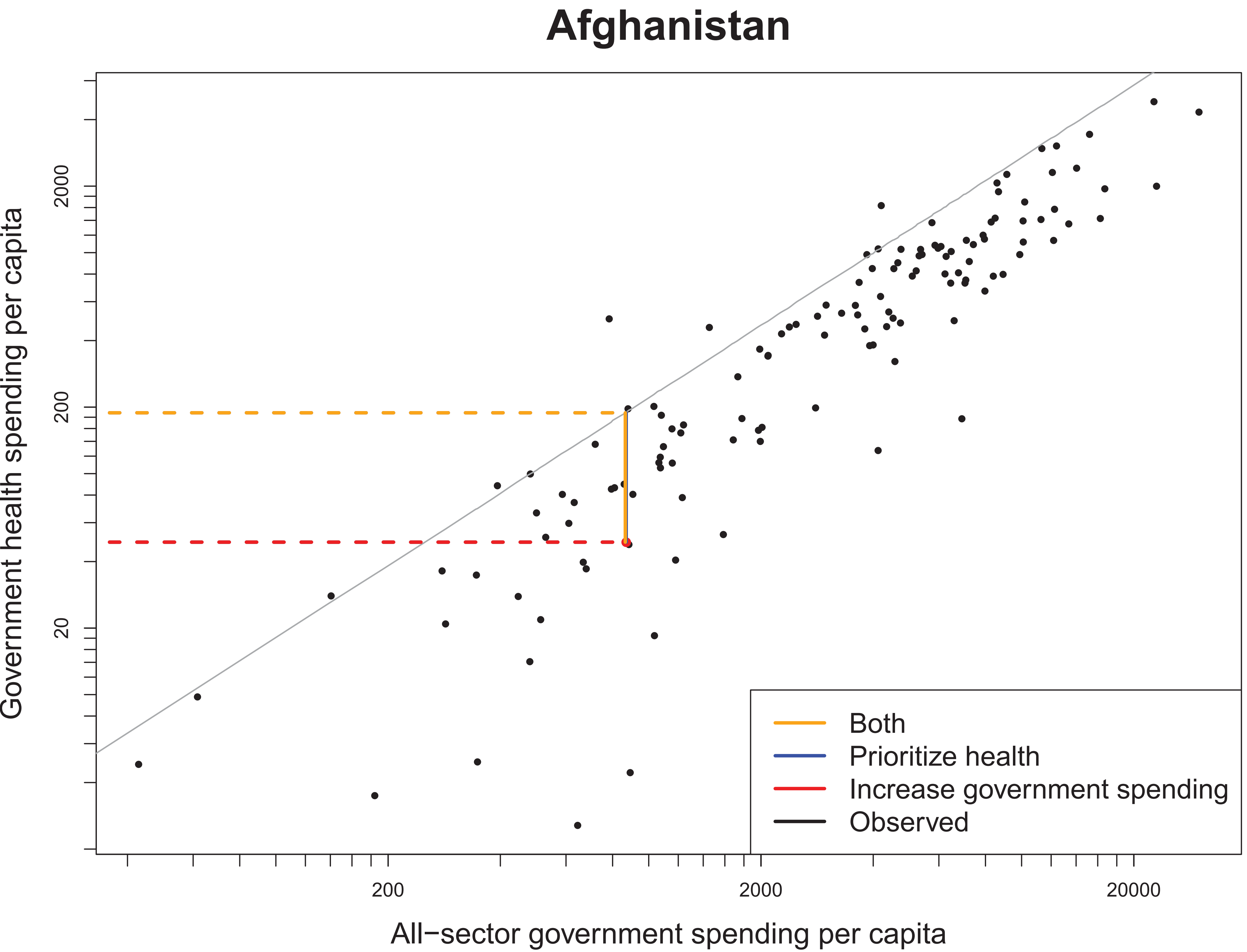

Afghanistan
Both
Prioritize health
Increase government spending
Observed
Government health spending per capita
20
200
2000
All−sector government spending per capita
200
2000
20000

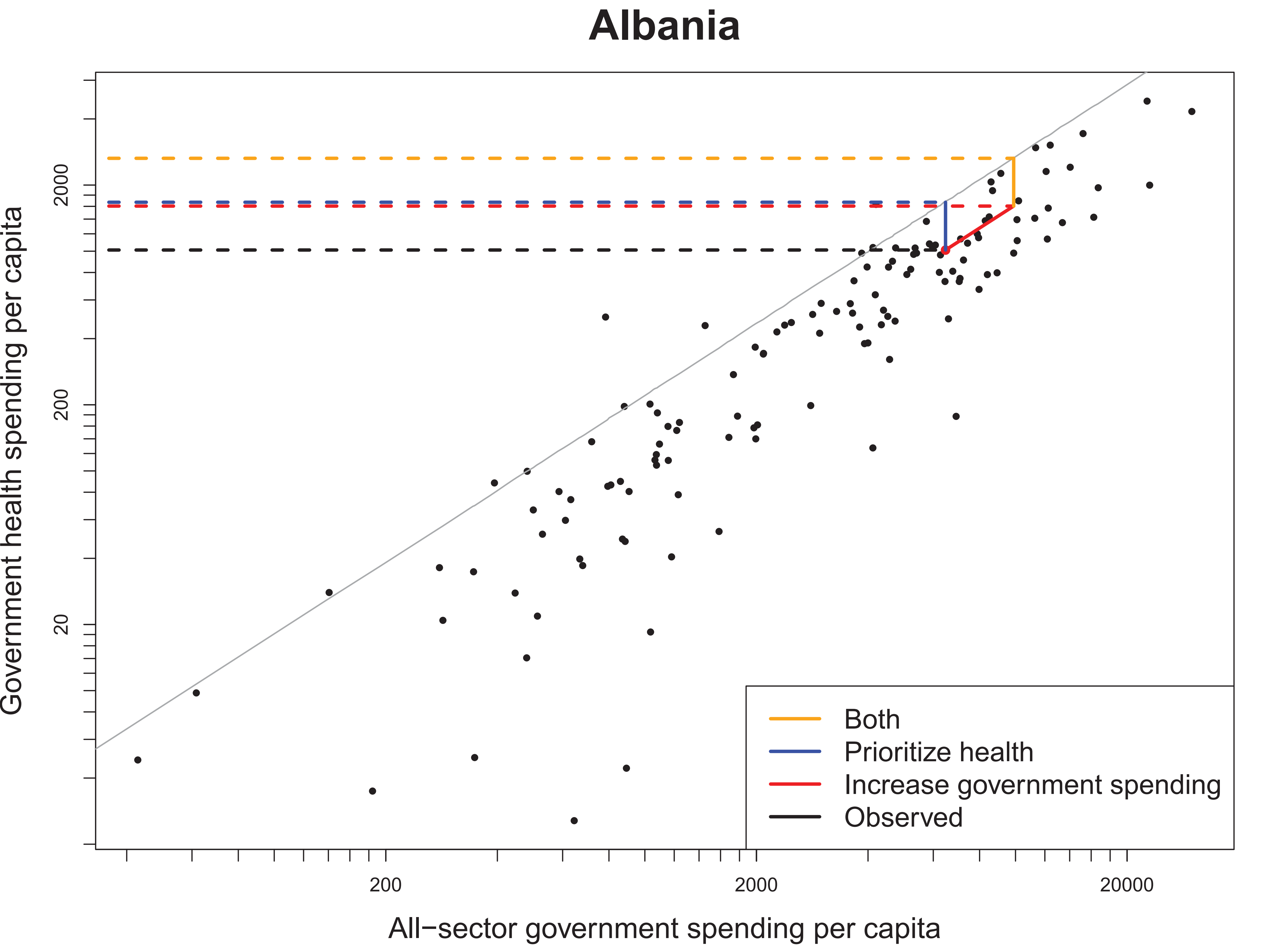
Albania
Both
Prioritize health
Increase government spending
Observed
Government health spending per capita
20
200
2000
All−sector government spending per capita
200
2000
20000

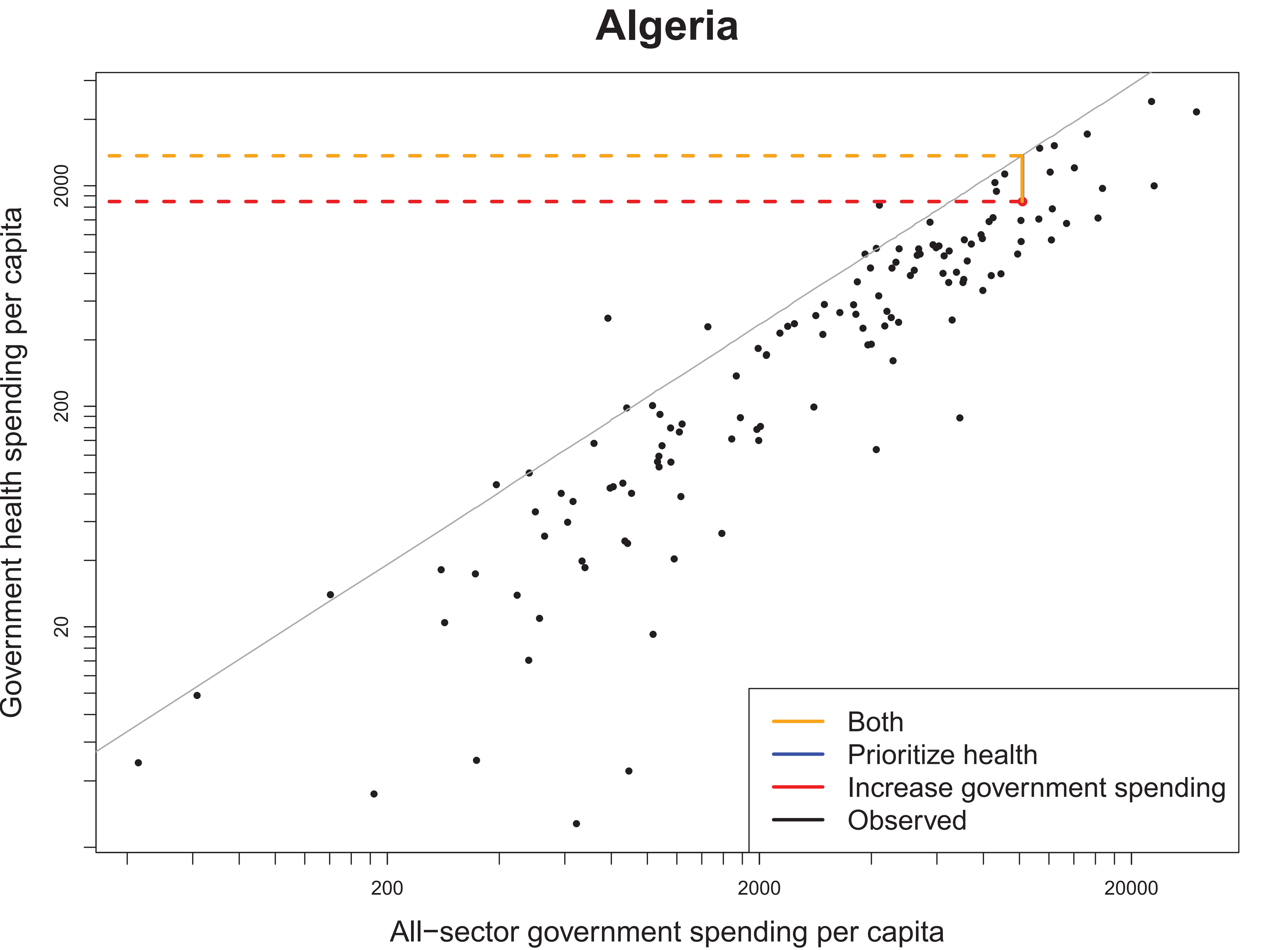

Algeria
Government health spending per capita
All−sector government spending per capita
20
200
2000
200
2000
20000
Both
Prioritize health
Increase government spending
Observed

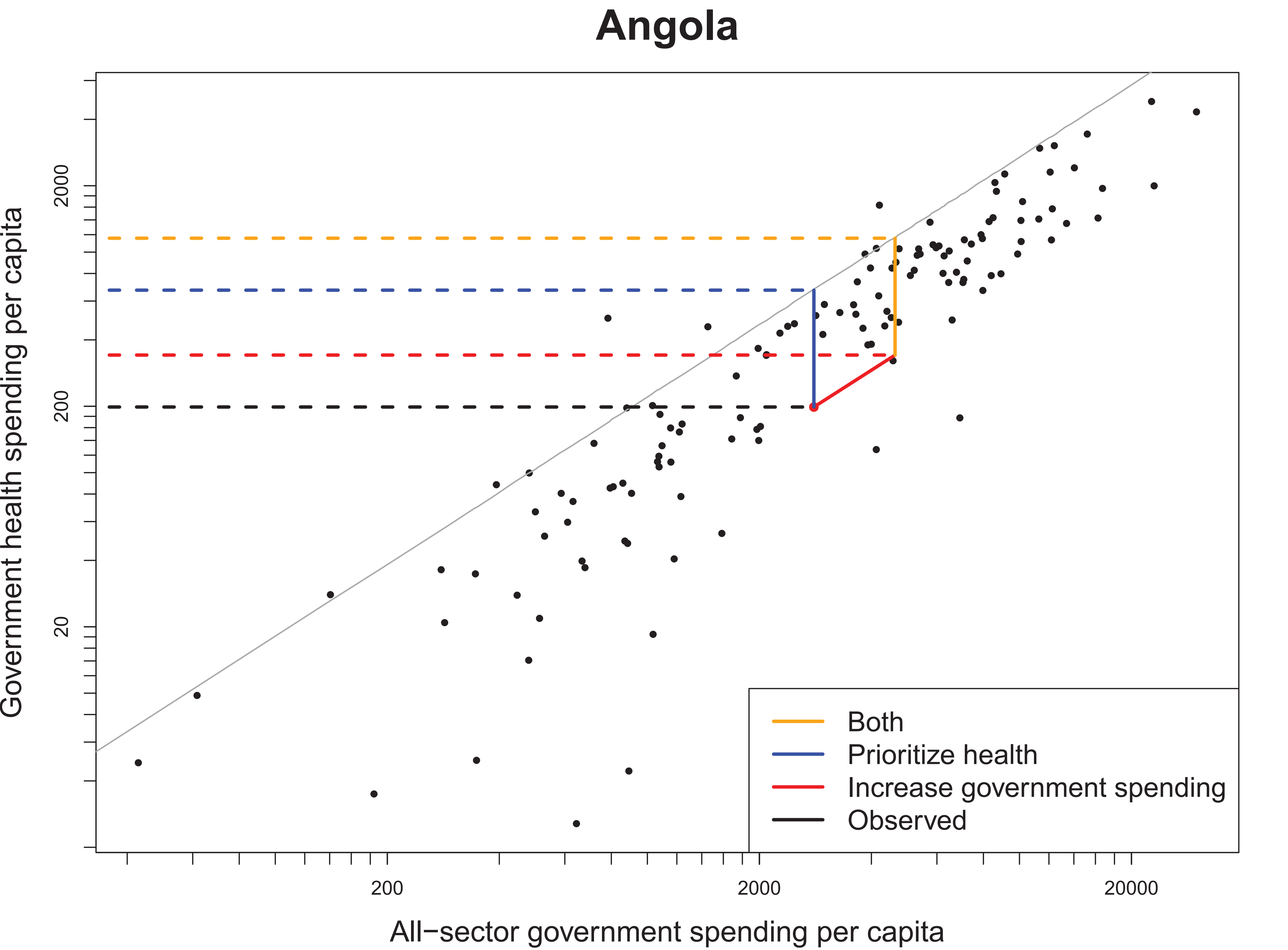

Angola
Government health spending per capita
2000
200
20
200
2000
20000
All−sector government spending per capita
Both
Prioritize health
Increase government spending
Observed

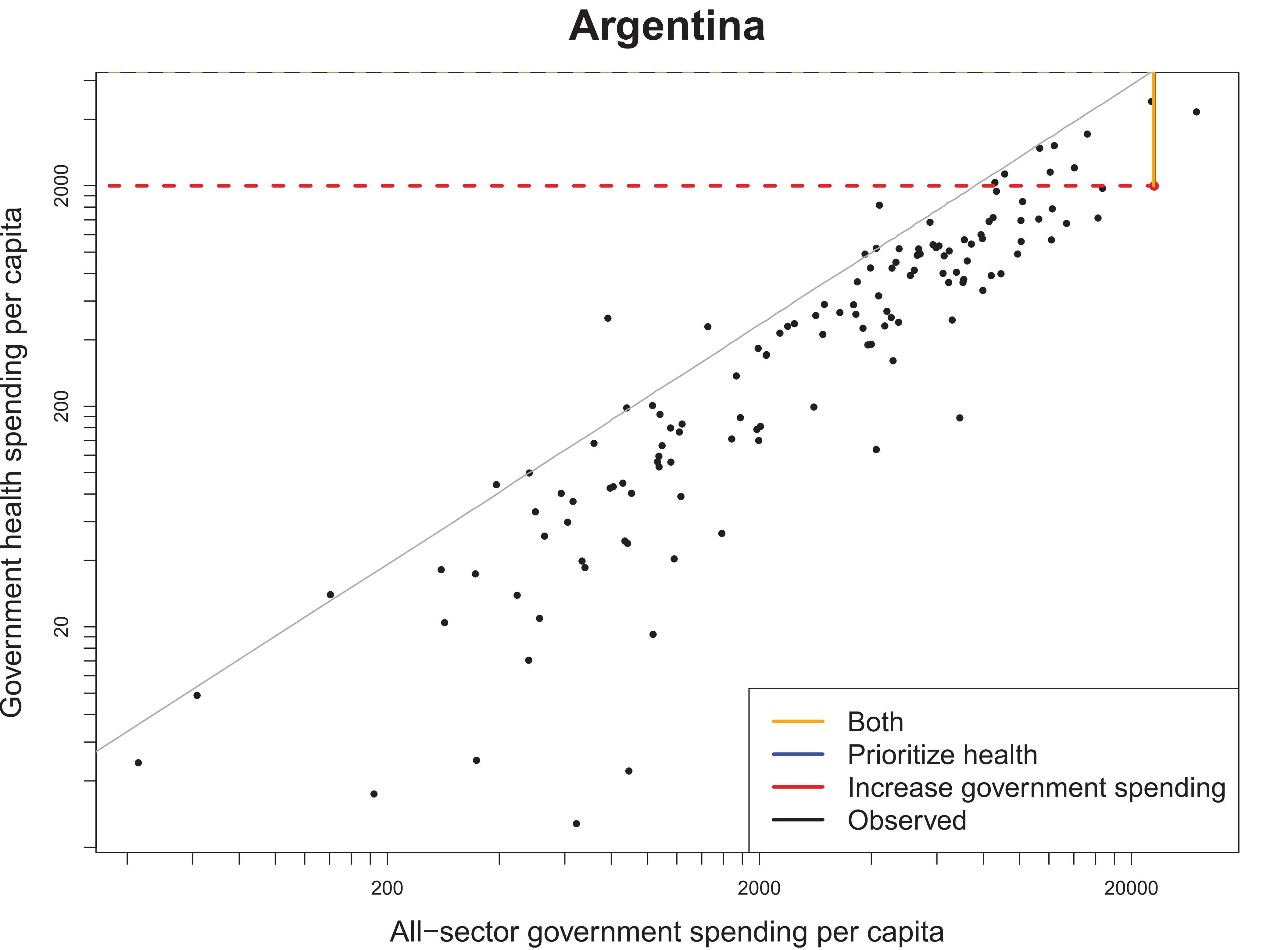

Argentina
Government health spending per capita
All−sector government spending per capita
20
200
2000
200
2000
20000
Both
Prioritize health
Increase government spending
Observed

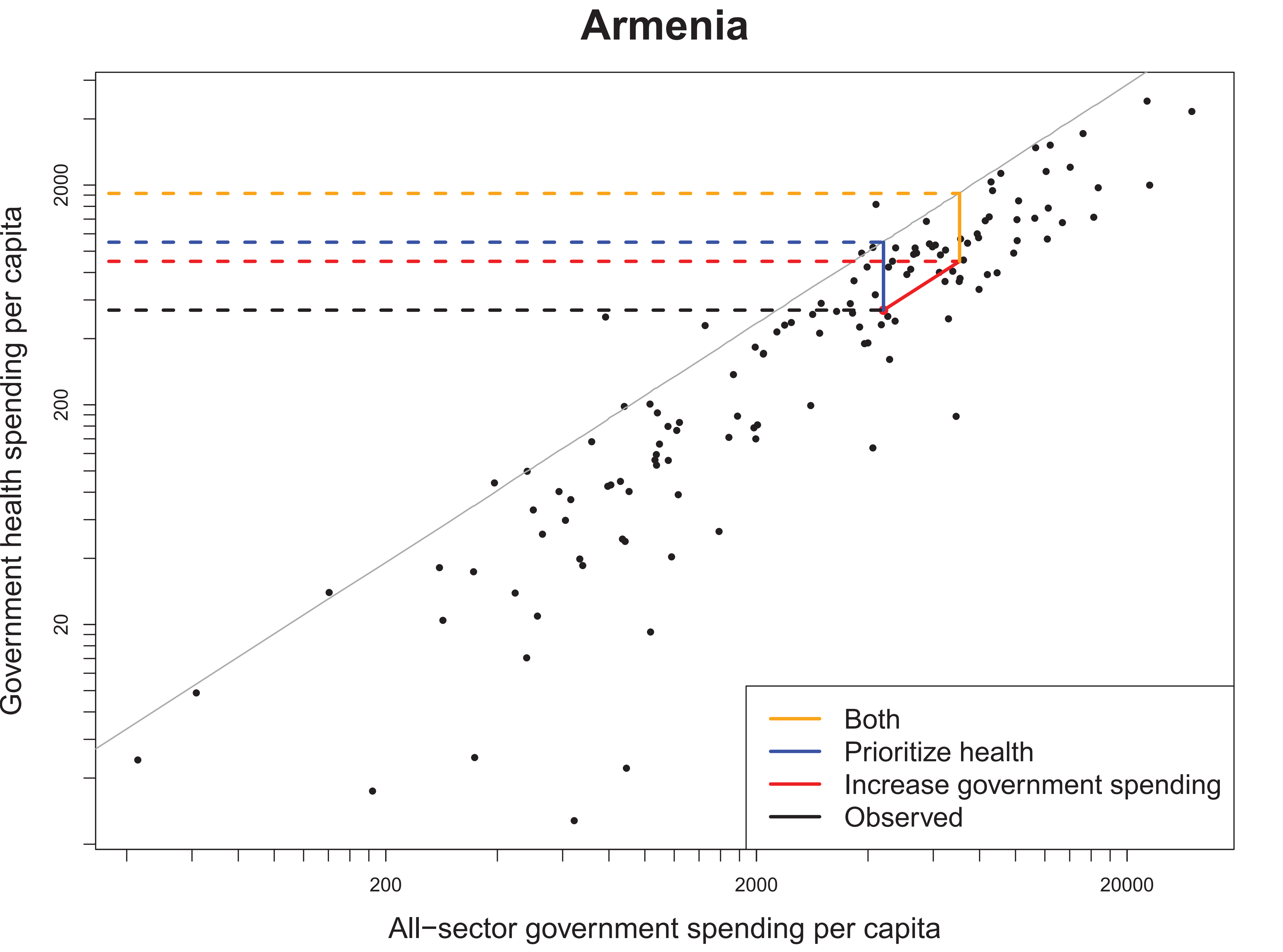
Armenia
Government health spending per capita
20
200
2000
All−sector government spending per capita
200
2000
20000
Both
Prioritize health
Increase government spending
Observed

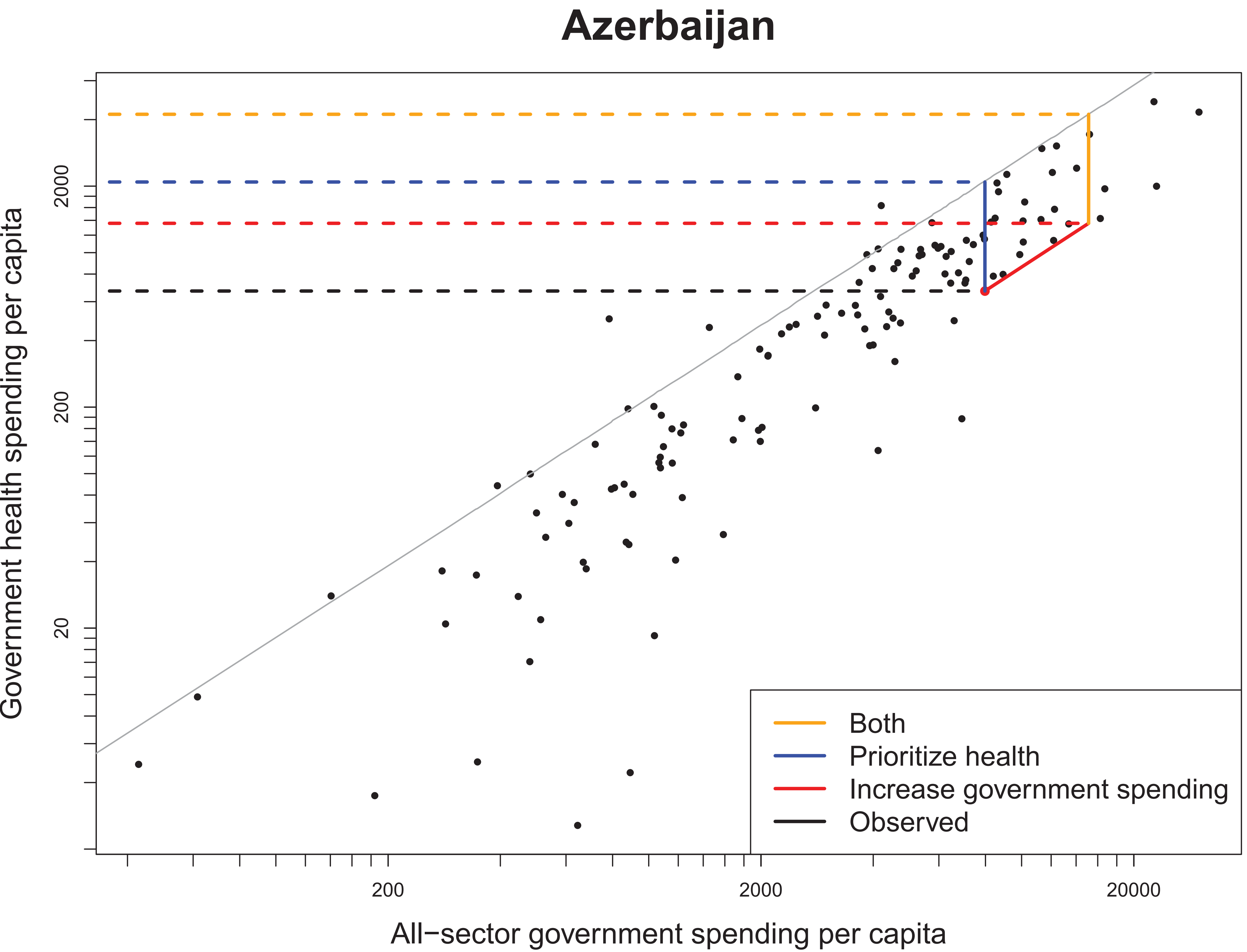

Azerbaijan
Government health spending per capita
All−sector government spending per capita
20
200
2000
200
2000
20000
Both
Prioritize health
Increase government spending
Observed

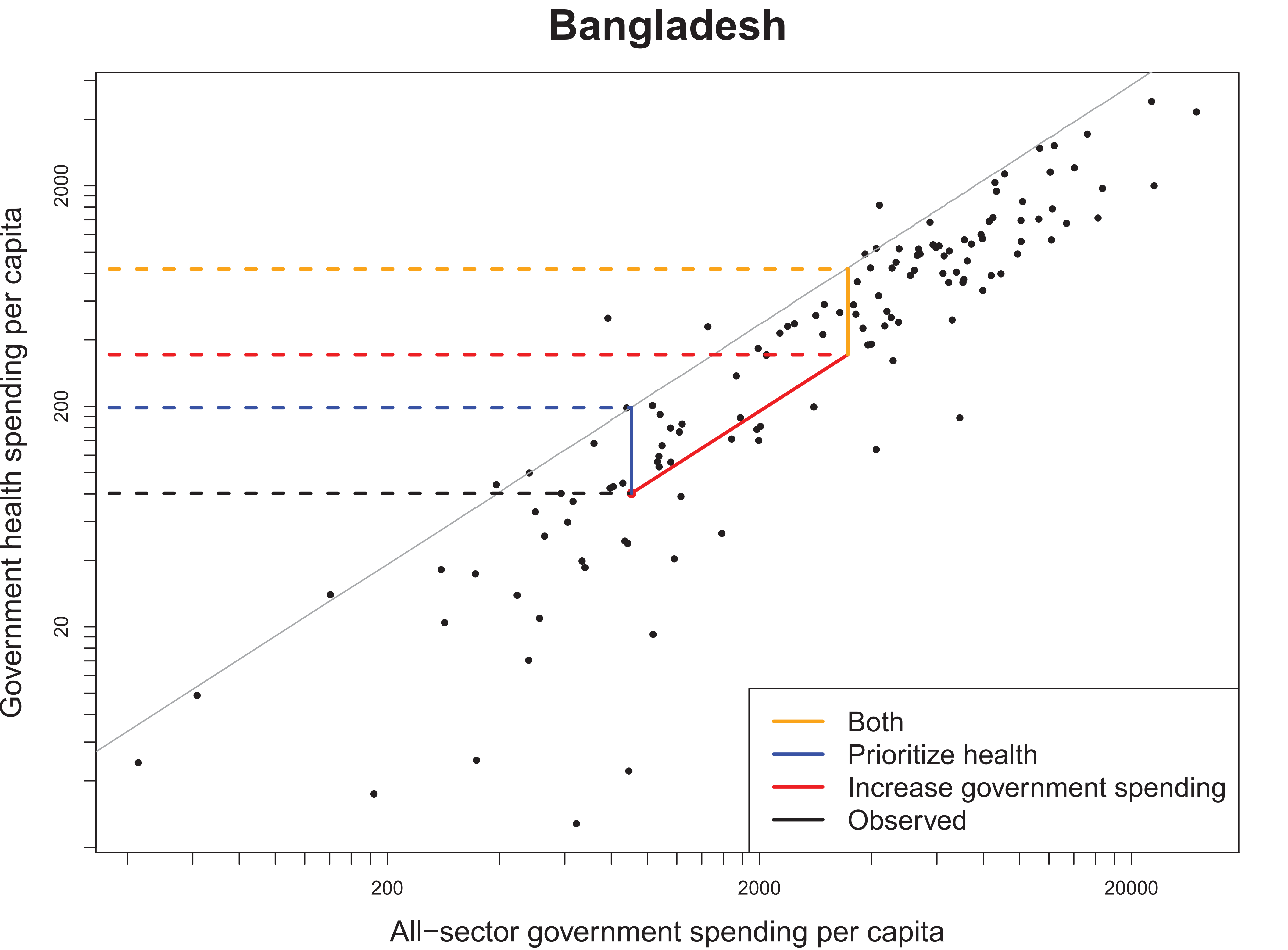

Bangladesh
Government health spending per capita
2000
200
20
200
2000
20000
All−sector government spending per capita
Both
Prioritize health
Increase government spending
Observed

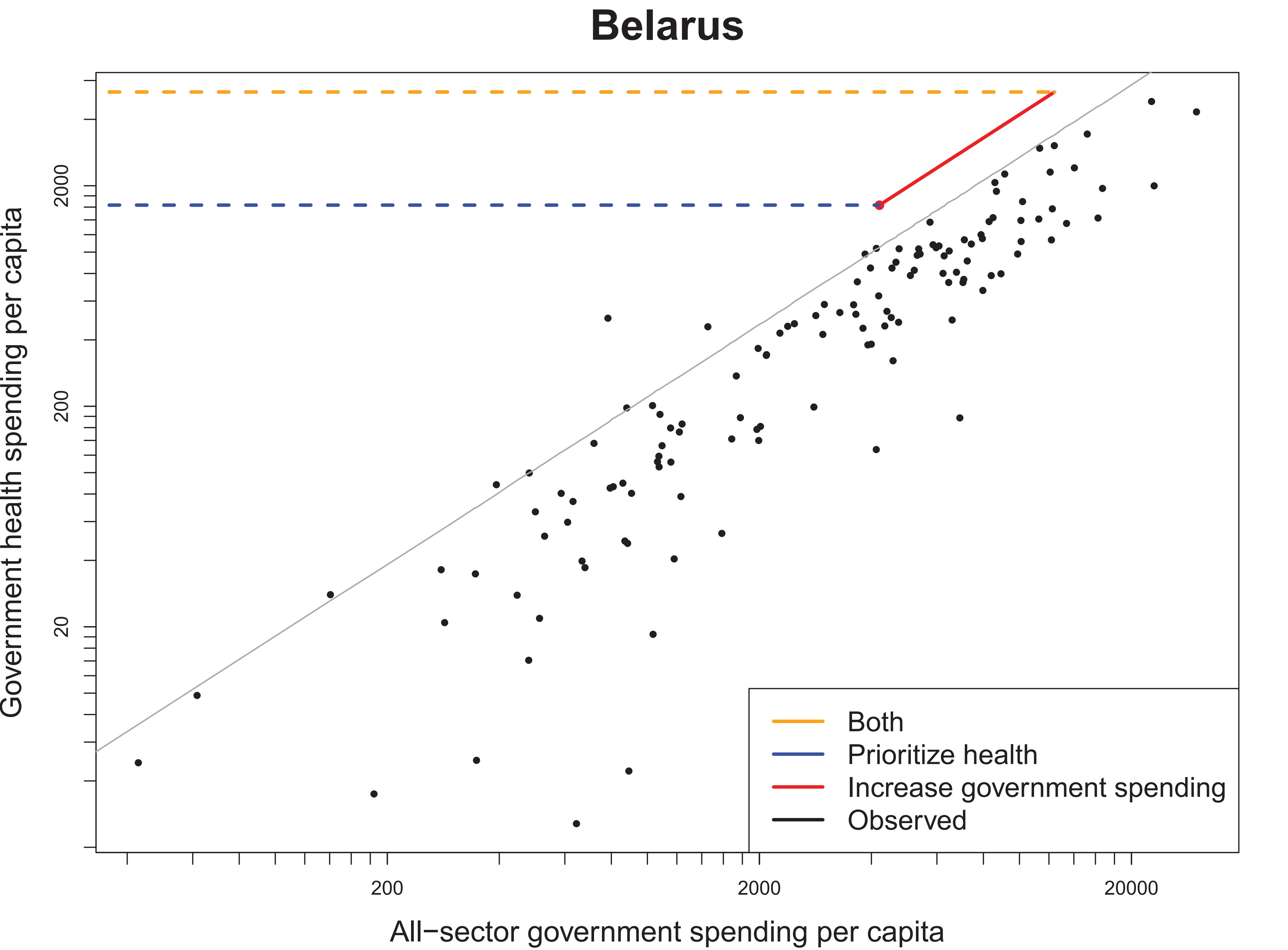
Belarus
Government health spending per capita
All−sector government spending per capita
20
200
2000
20000
Both
Prioritize health
Increase government spending
Observed

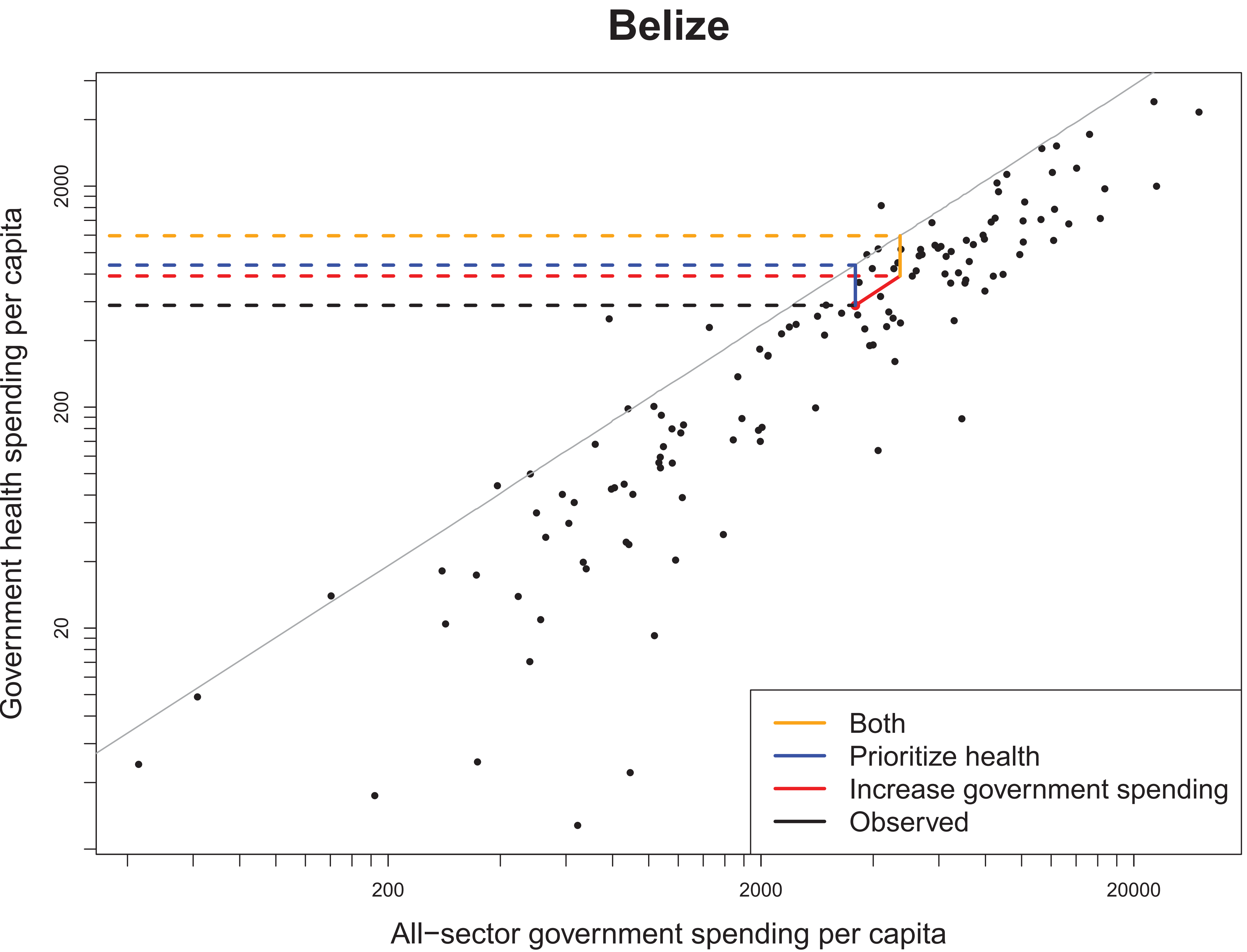

Belize
Government health spending per capita
20
200
2000
All−sector government spending per capita
200
2000
20000
Both
Prioritize health
Increase government spending
Observed

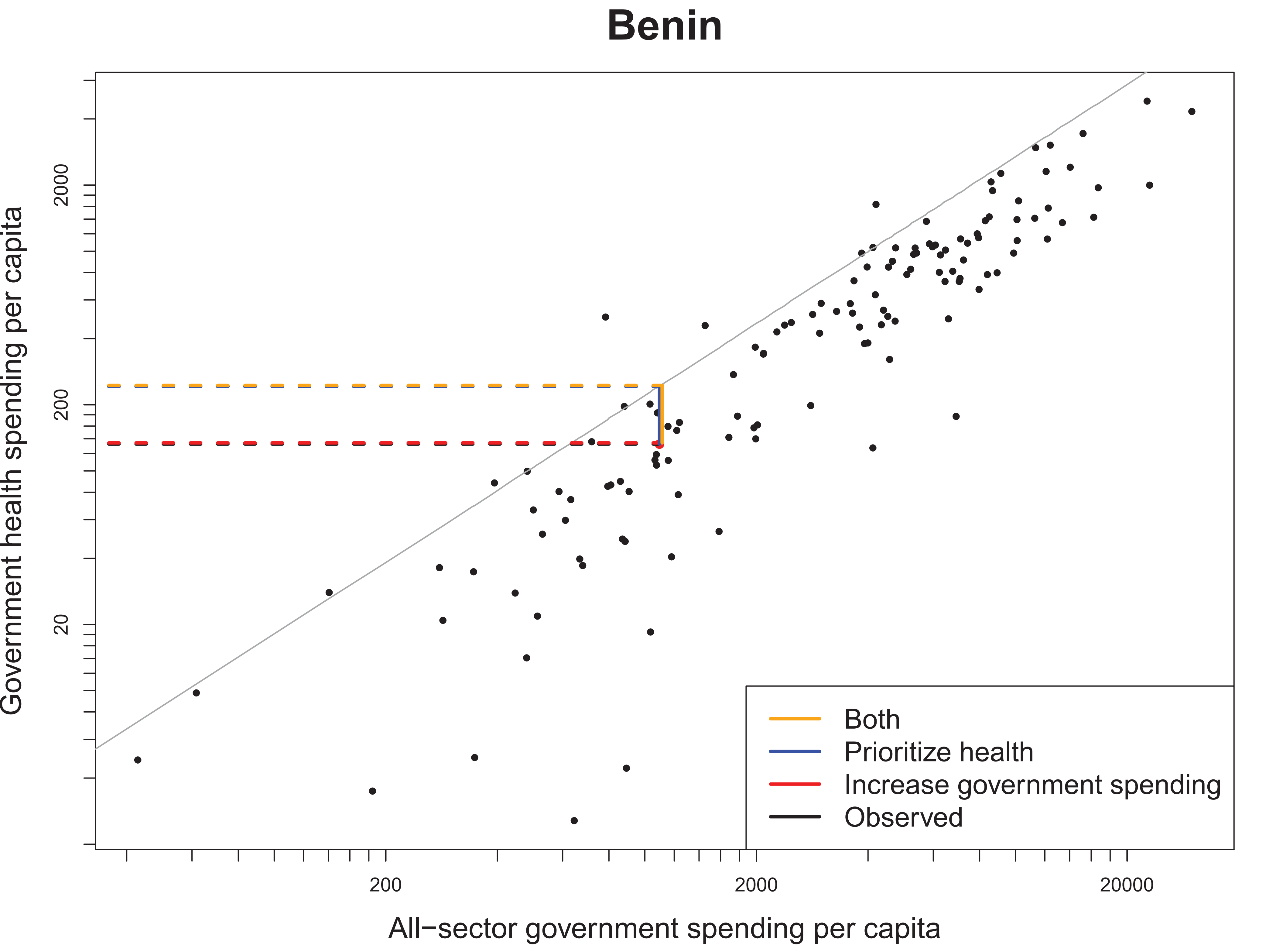
Benin
Government health spending per capita
20
200
2000
200
2000
20000
All−sector government spending per capita
Both
Prioritize health
Increase government spending
Observed

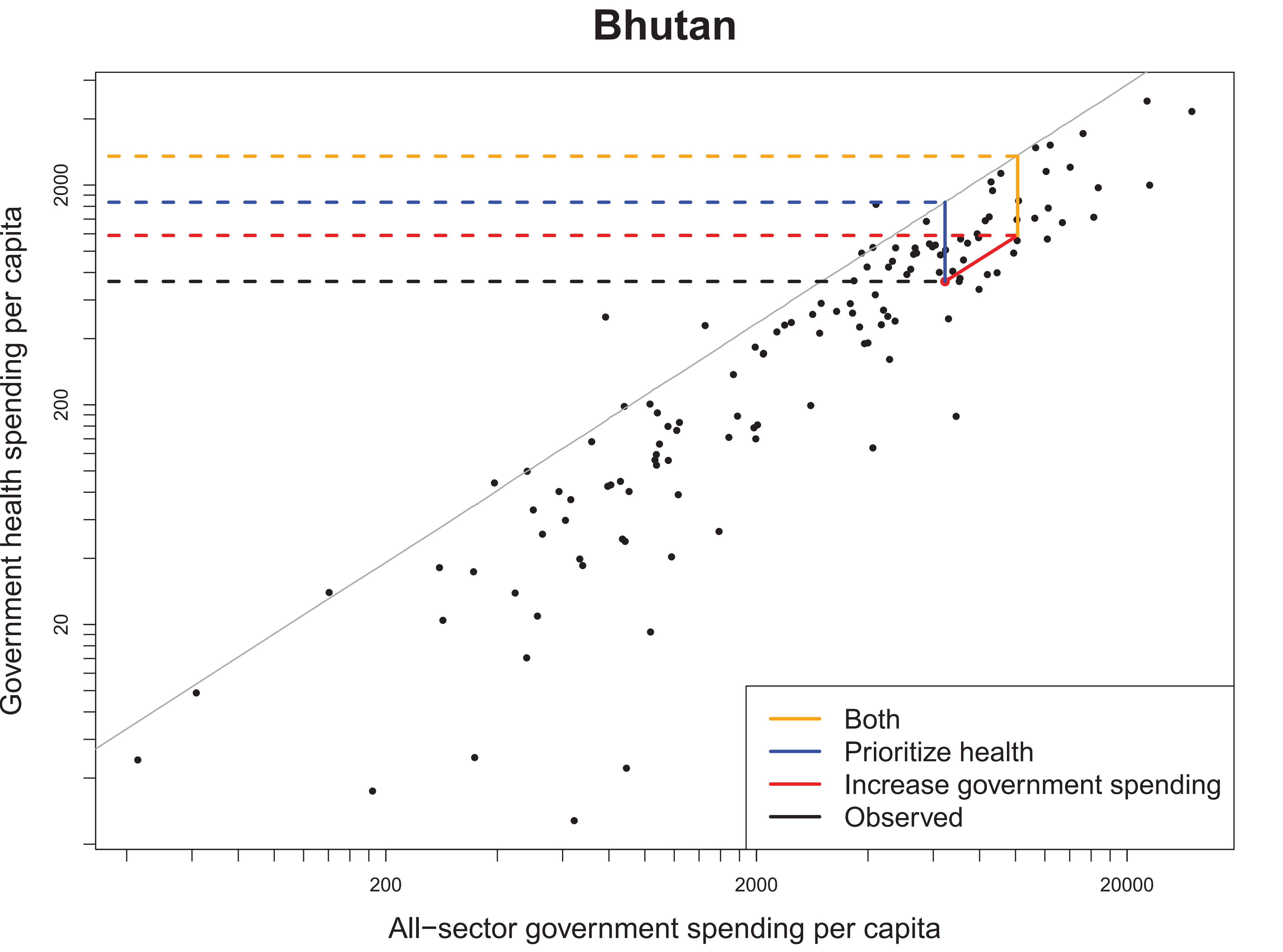

Bhutan
Government health spending per capita
All−sector government spending per capita
20
200
2000
20000
Both
Prioritize health
Increase government spending
Observed

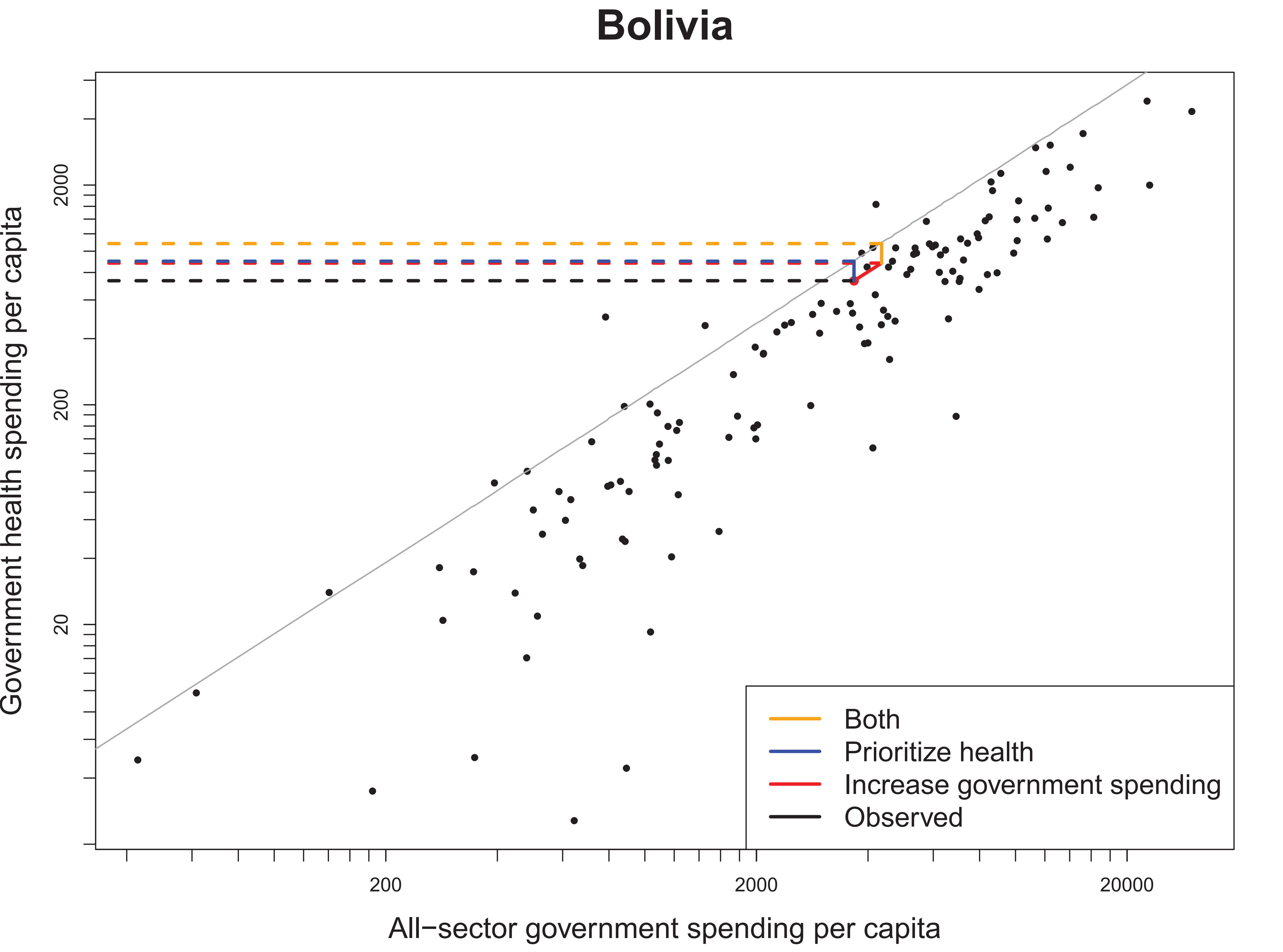

Bolivia
Government health spending per capita
All−sector government spending per capita
20
200
2000
200
2000
20000
Both
Prioritize health
Increase government spending
Observed

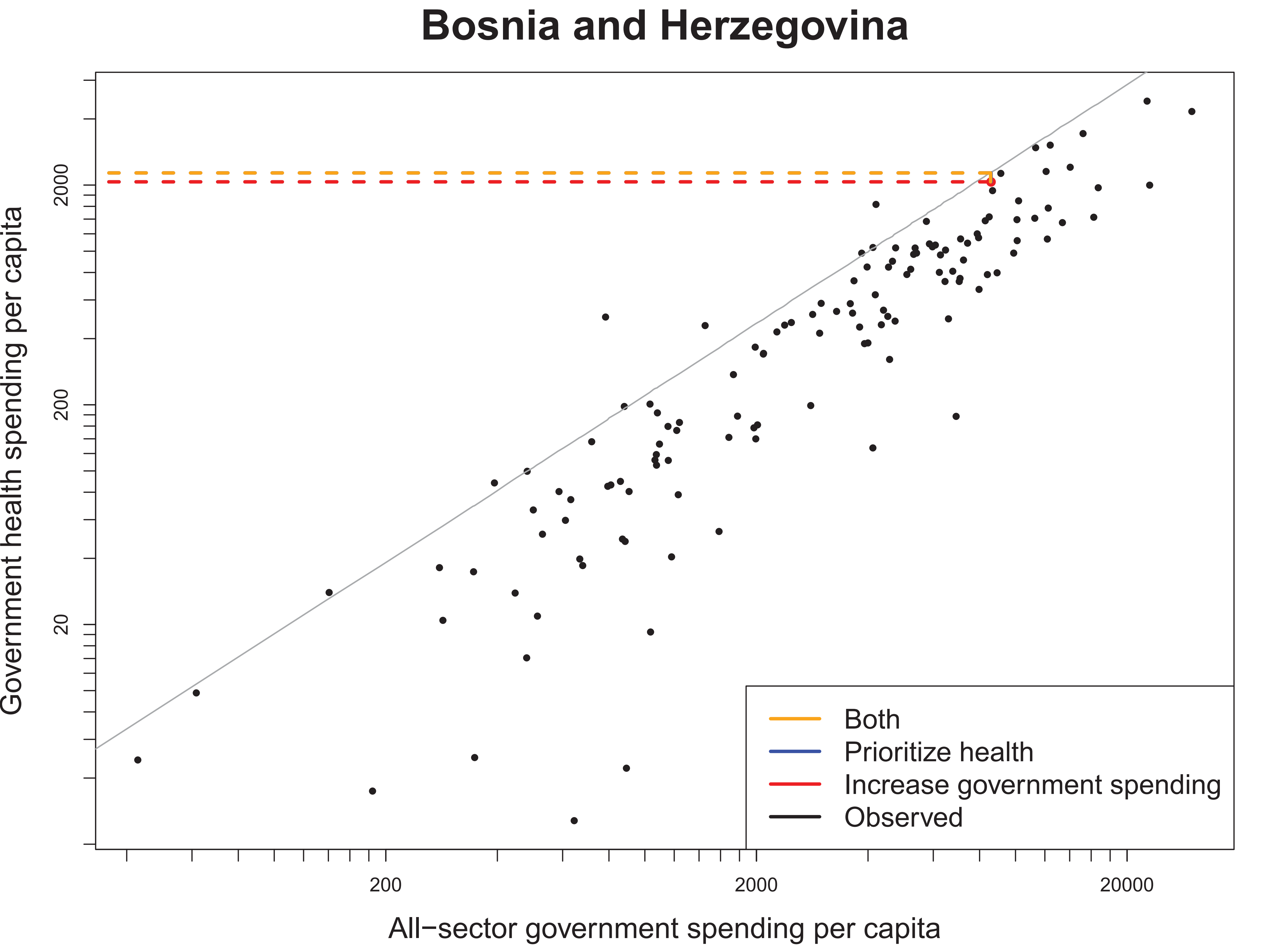

Bosnia and Herzegovina
Government health spending per capita
20
200
2000
All−sector government spending per capita
200
2000
20000
Both
Prioritize health
Increase government spending
Observed

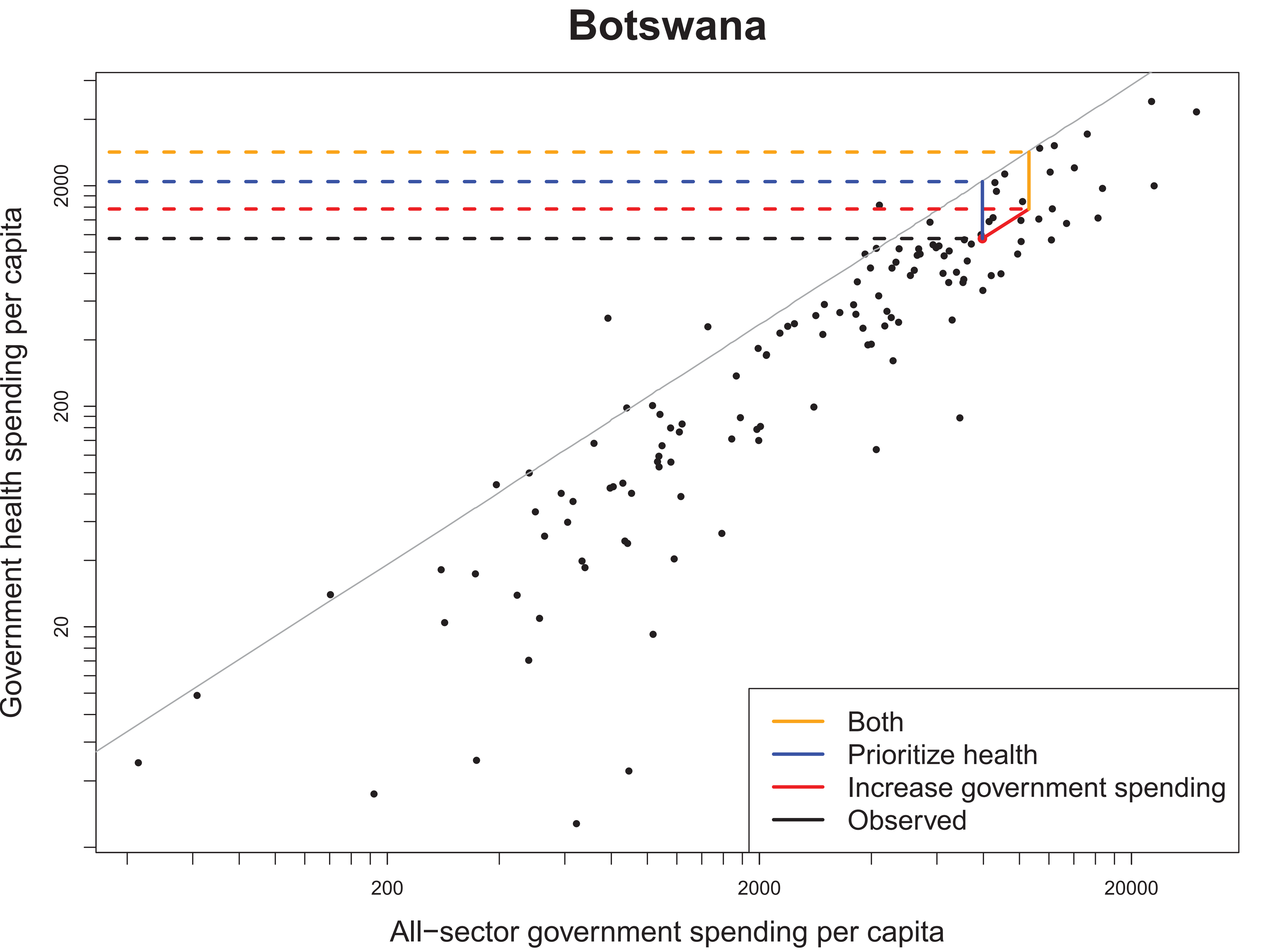

Botswana
Government health spending per capita
All−sector government spending per capita
20
200
2000
20000
Both
Prioritize health
Increase government spending
Observed

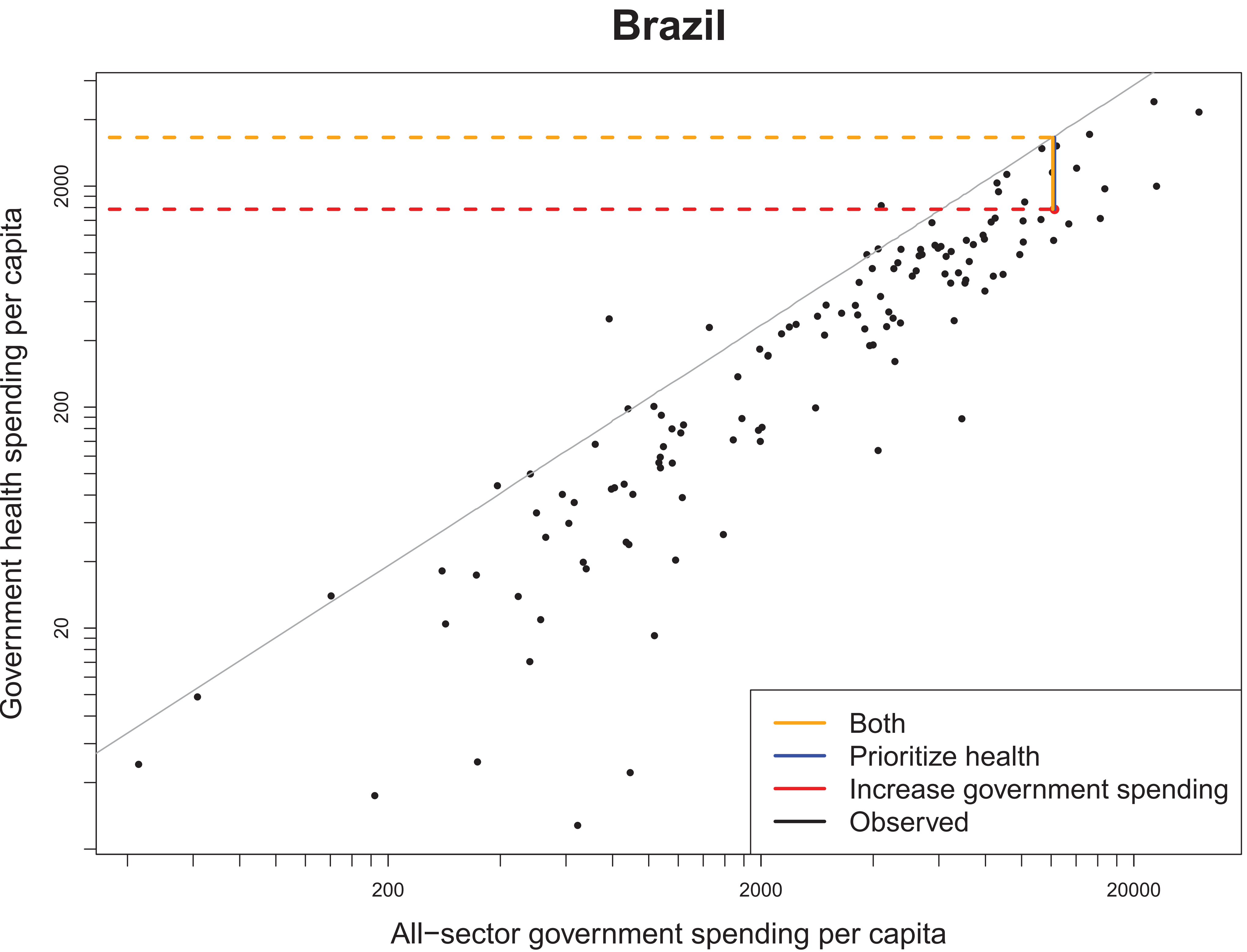

Brazil
Government health spending per capita
20
200
2000
All−sector government spending per capita
200
2000
20000
Both
Prioritize health
Increase government spending
Observed

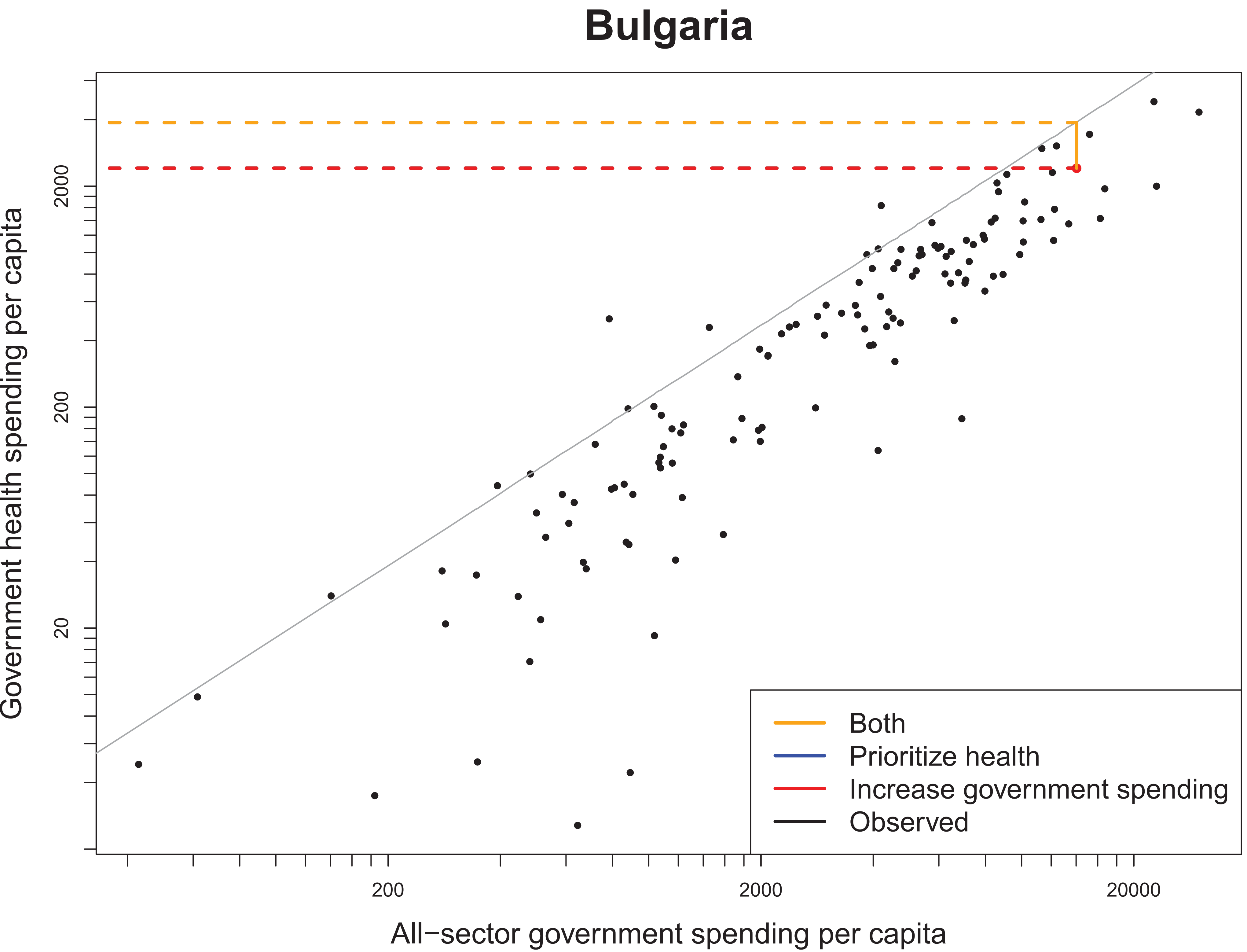
Bulgaria
Government health spending per capita
20
200
2000
All−sector government spending per capita
200
2000
20000
Both
Prioritize health
Increase government spending
Observed

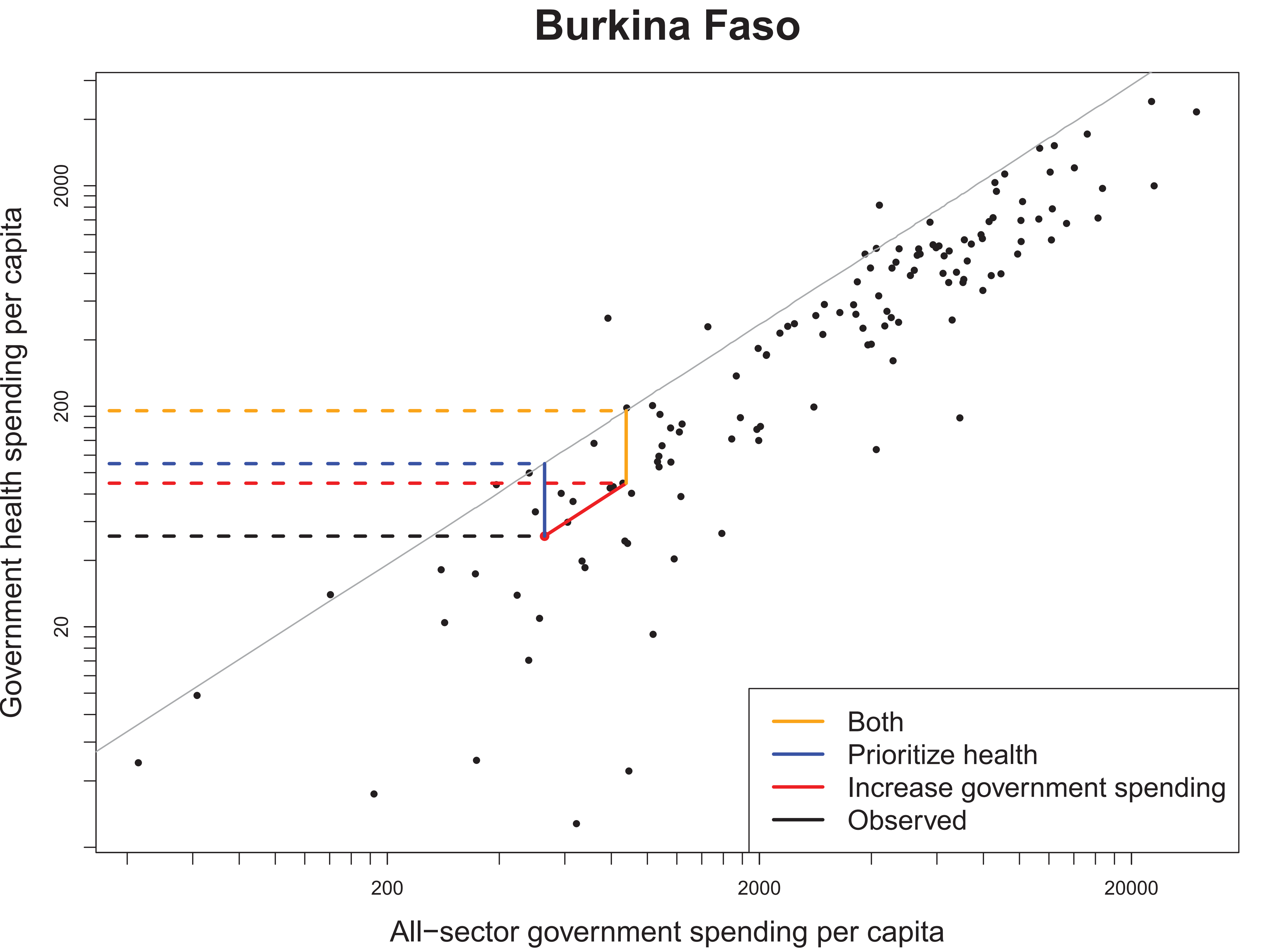

Burkina Faso
Government health spending per capita
All−sector government spending per capita
20
200
2000
200
2000
20000
Both
Prioritize health
Increase government spending
Observed

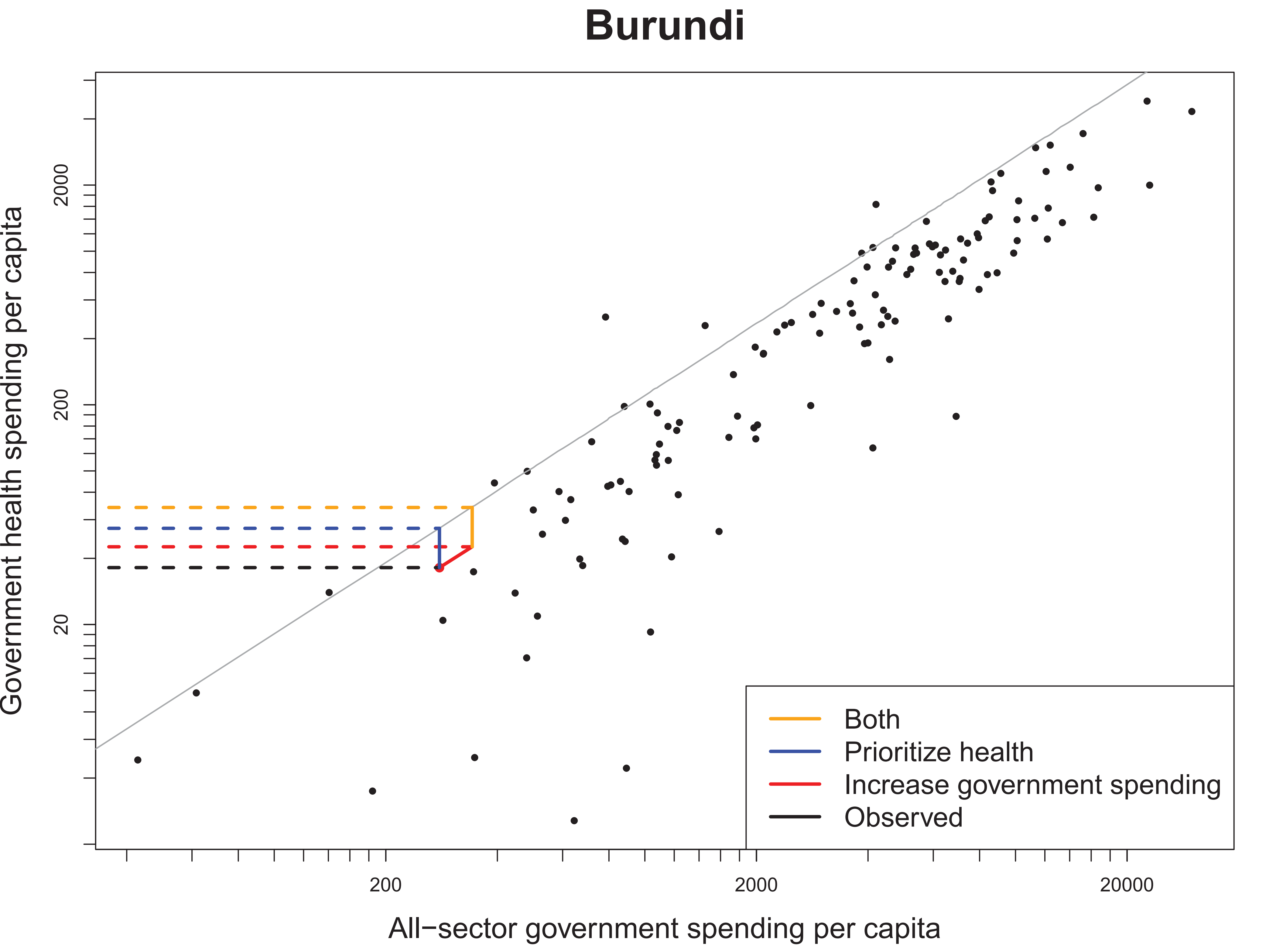

Burundi
Government health spending per capita
All−sector government spending per capita
20
200
2000
20000
Both
Prioritize health
Increase government spending
Observed

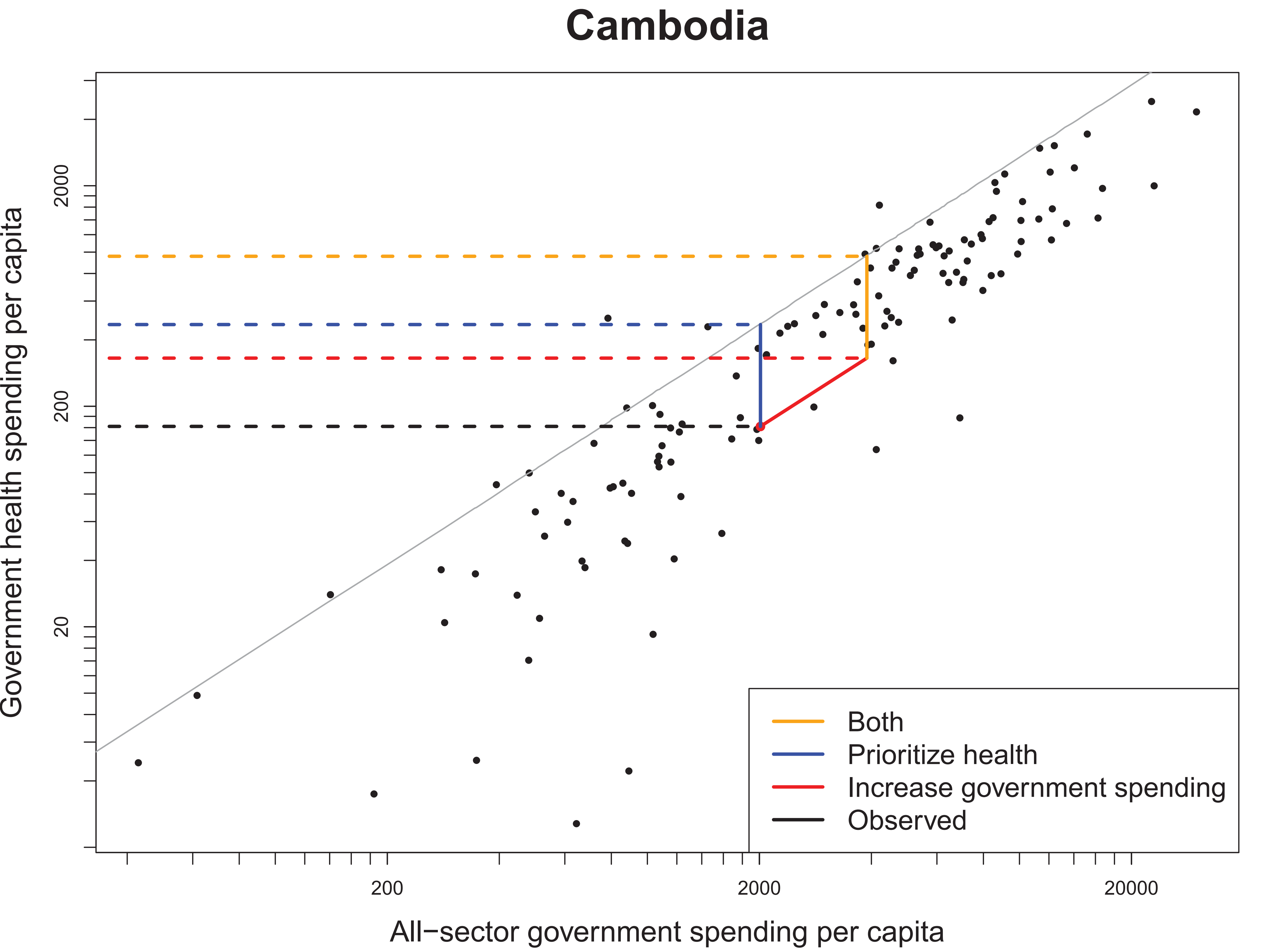

Cambodia
Government health spending per capita
2000
200
20
All−sector government spending per capita
200
2000
20000
Both
Prioritize health
Increase government spending
Observed

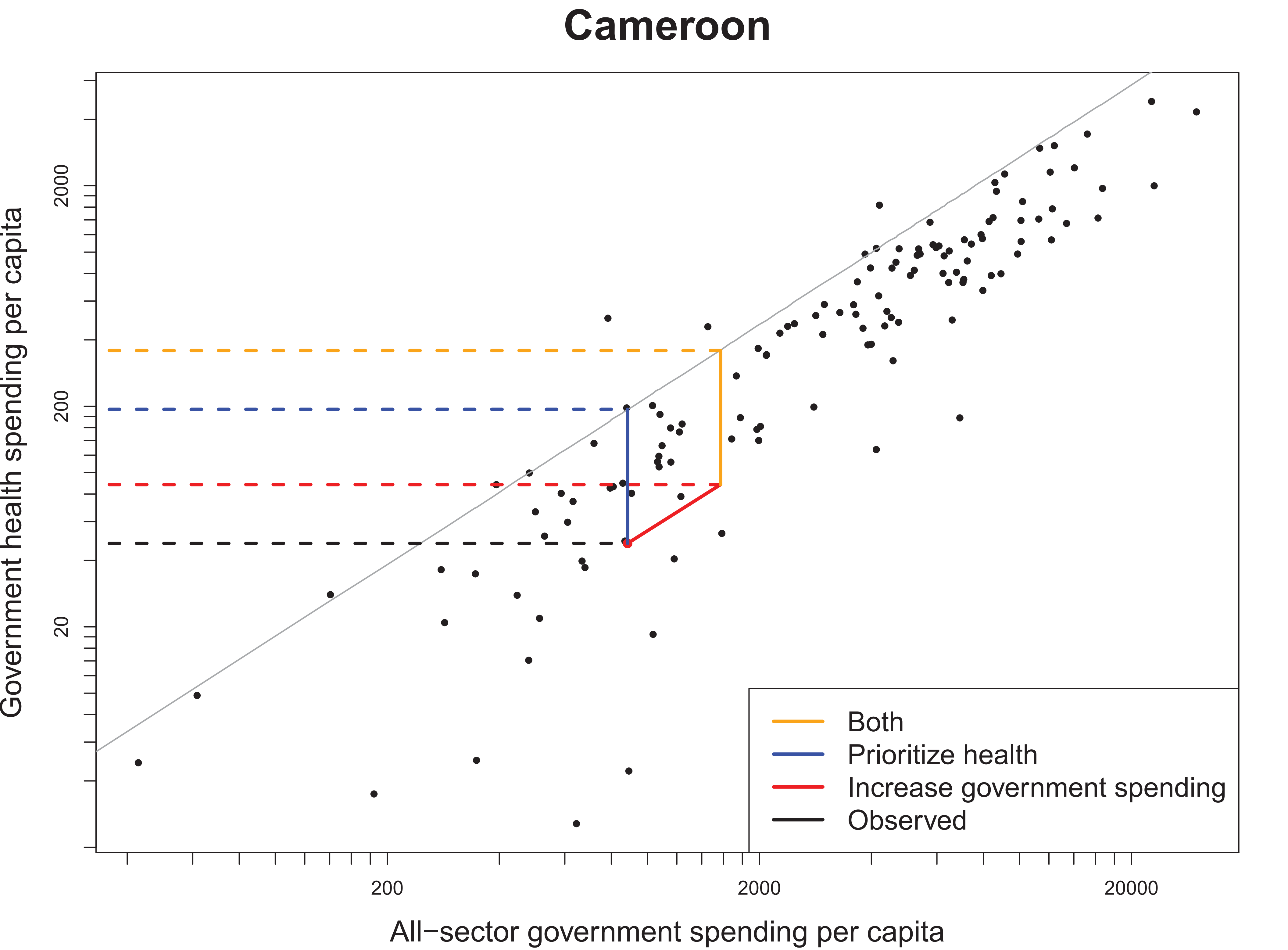

Cameroon
Government health spending per capita
All−sector government spending per capita
20
200
2000
20000
Both
Prioritize health
Increase government spending
Observed

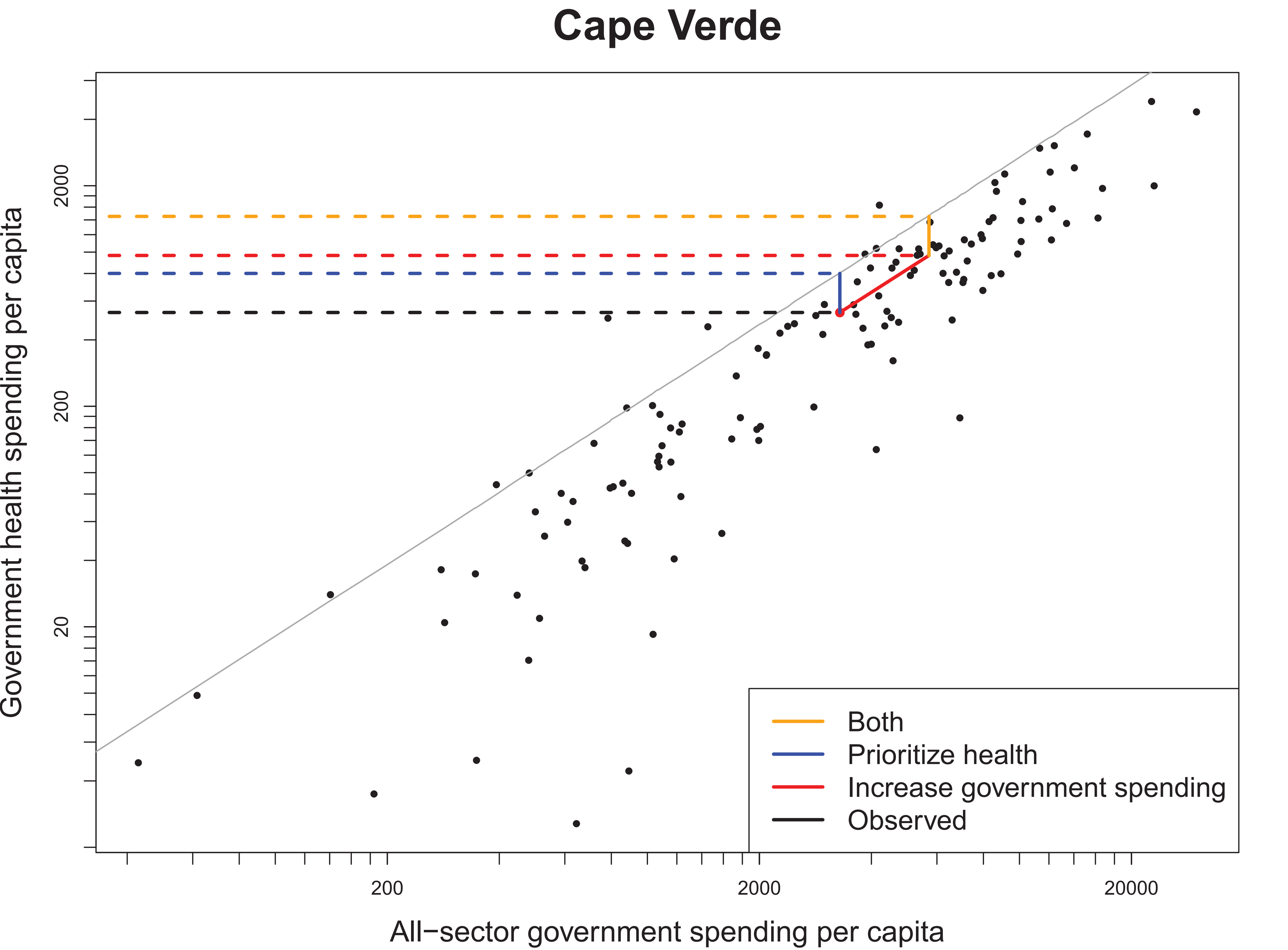
Cape Verde
Government health spending per capita
All−sector government spending per capita
20
200
2000
200
2000
20000
Both
Prioritize health
Increase government spending
Observed

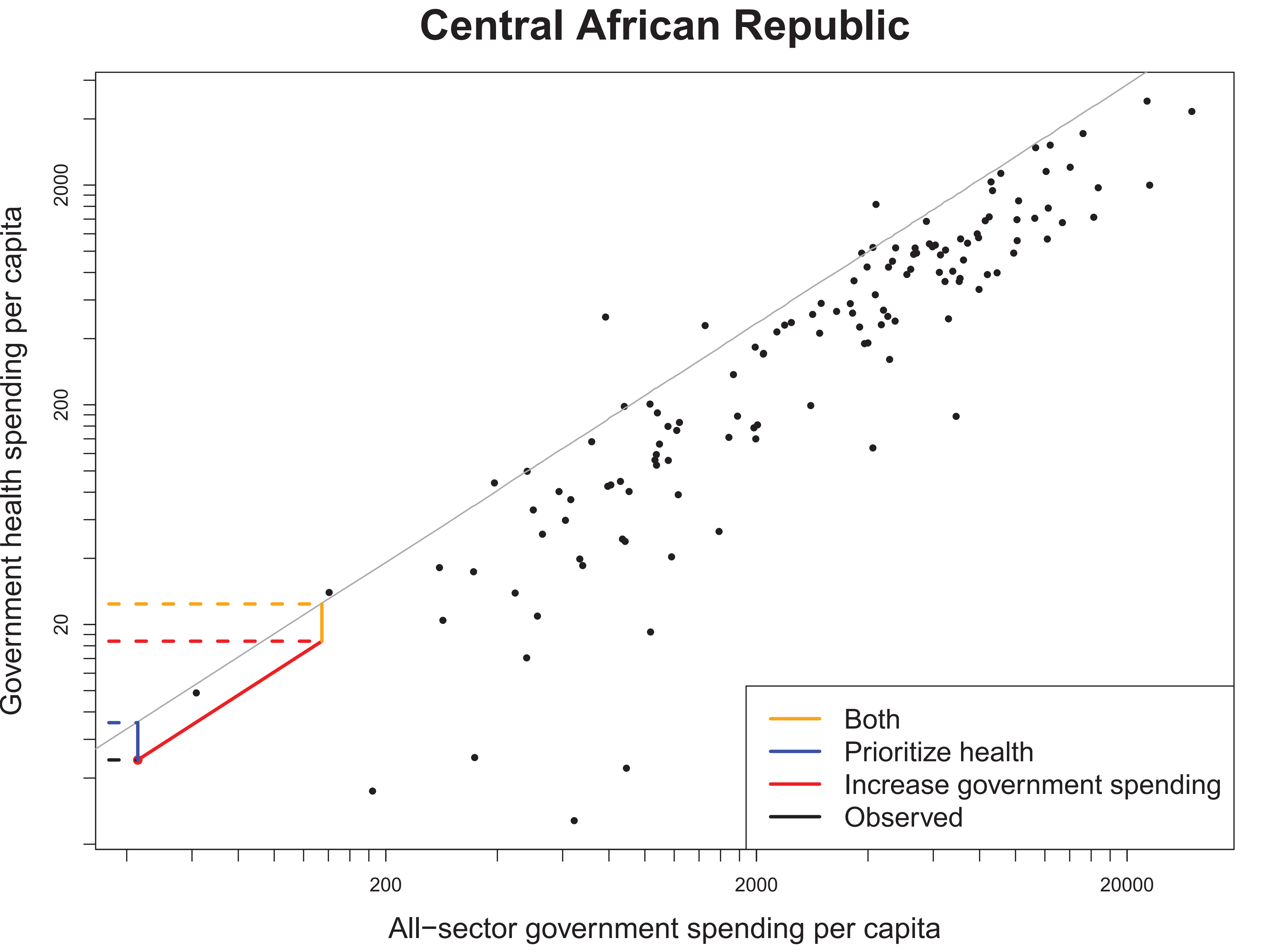

Central African Republic
Government health spending per capita
2000
200
20
All−sector government spending per capita
200
2000
20000
Both
Prioritize health
Increase government spending
Observed

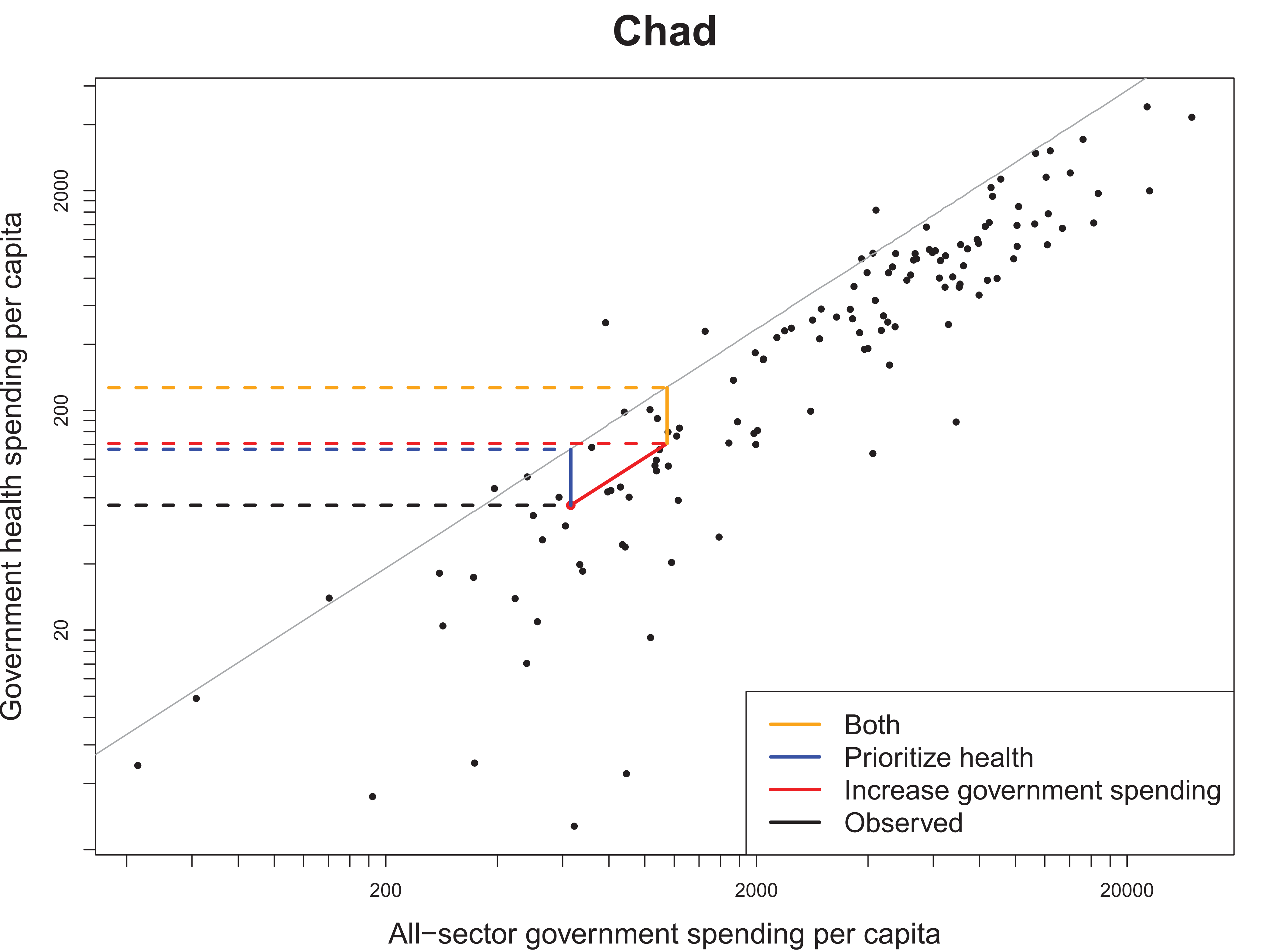

Chad
Government health spending per capita
20
200
2000
All−sector government spending per capita
200
2000
20000
Both
Prioritize health
Increase government spending
Observed

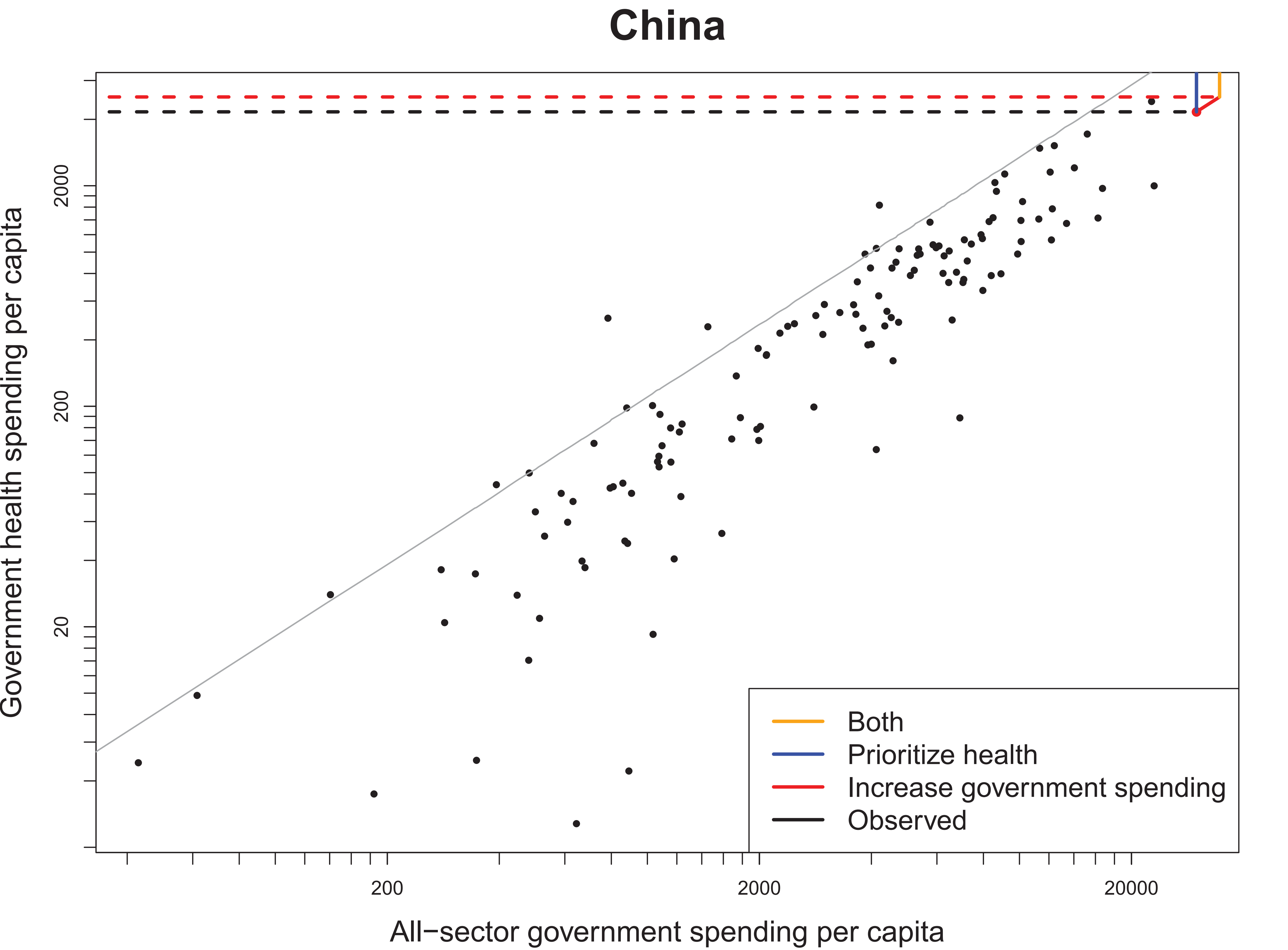

China
Government health spending per capita
All−sector government spending per capita
20
200
2000
200
2000
20000
Both
Prioritize health
Increase government spending
Observed

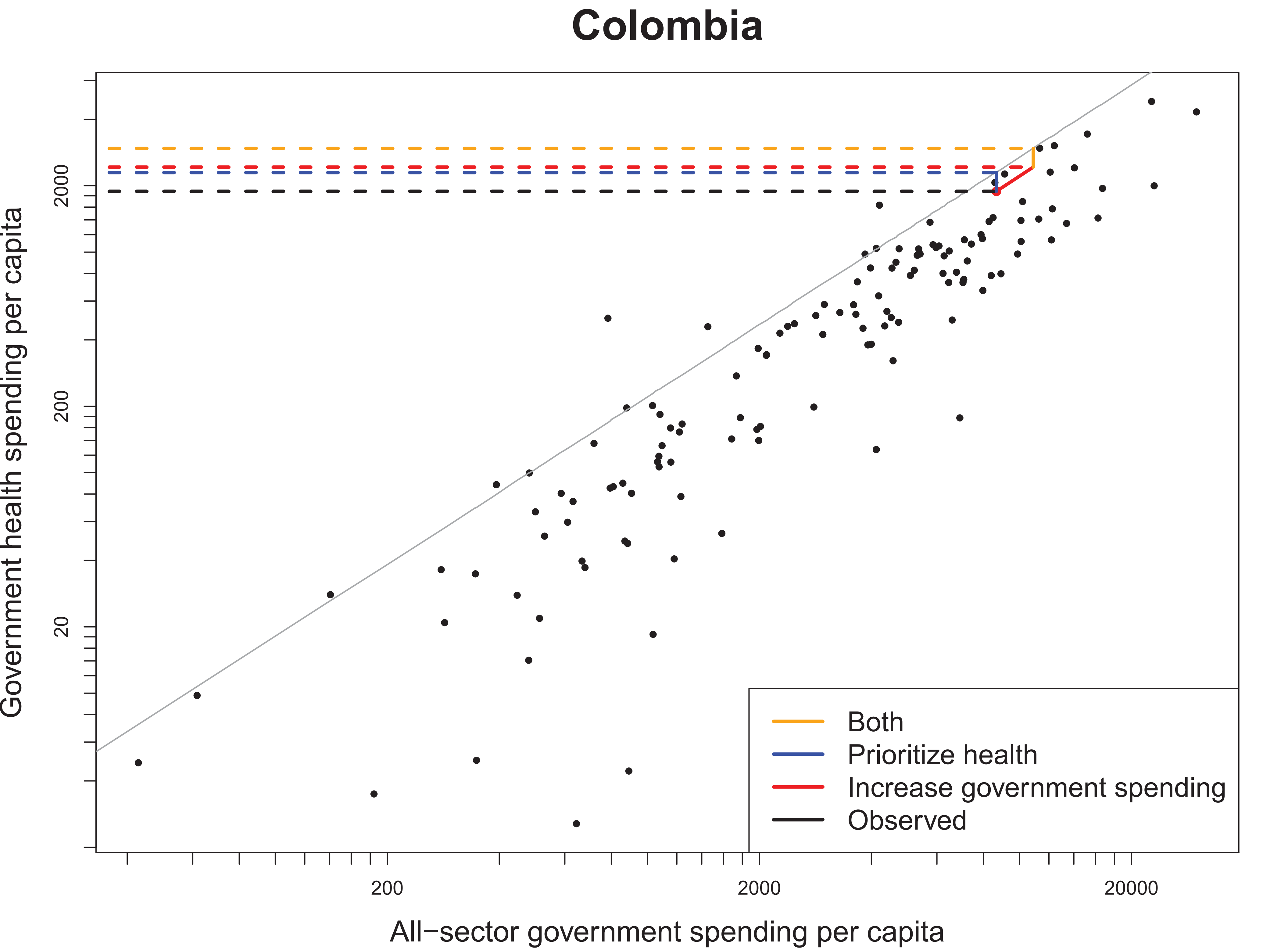
Colombia
Government health spending per capita
All−sector government spending per capita
20
200
2000
20000
Both
Prioritize health
Increase government spending
Observed

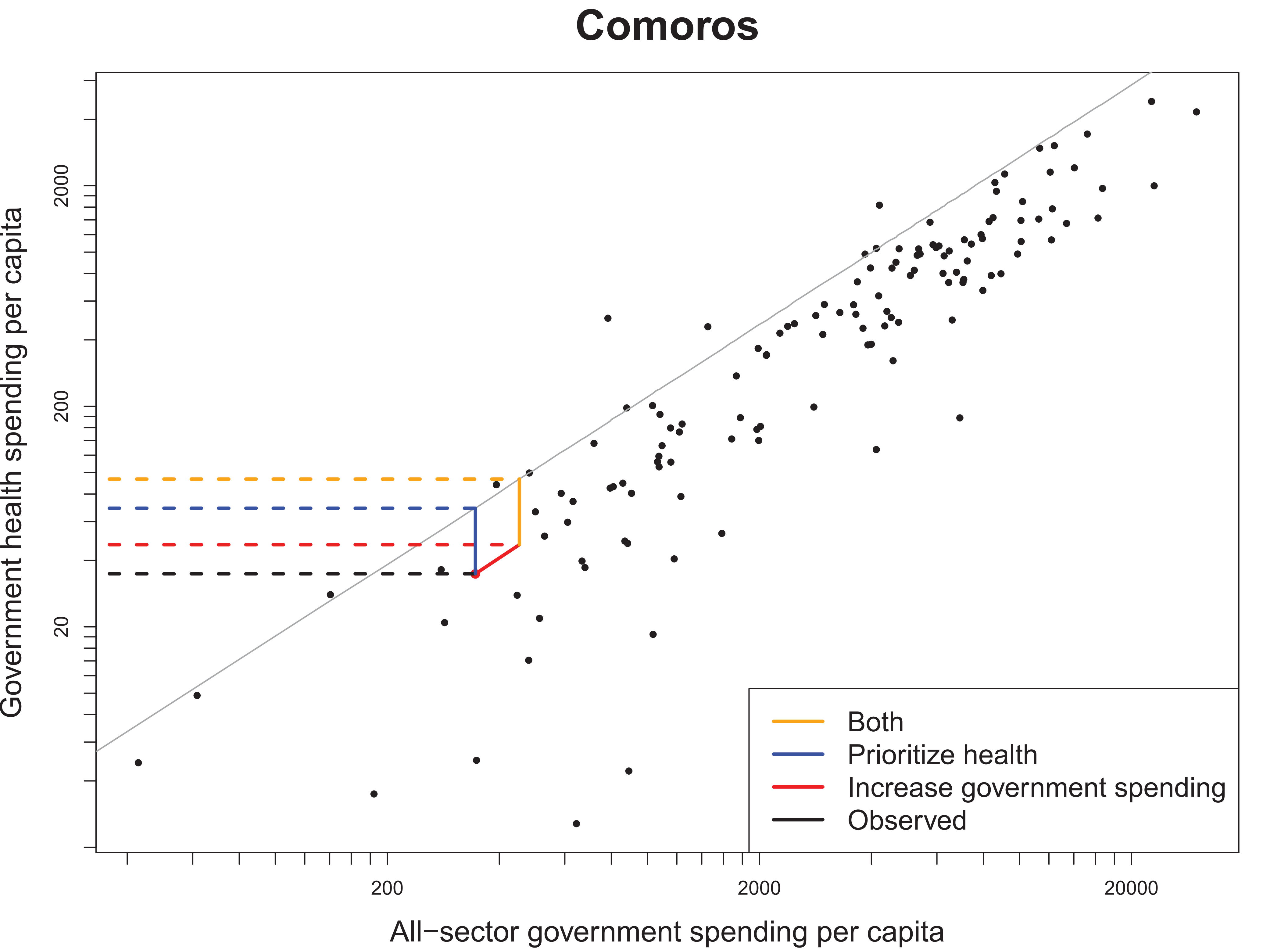

Comoros
Government health spending per capita
20
200
2000
All−sector government spending per capita
200
2000
20000
Both
Prioritize health
Increase government spending
Observed

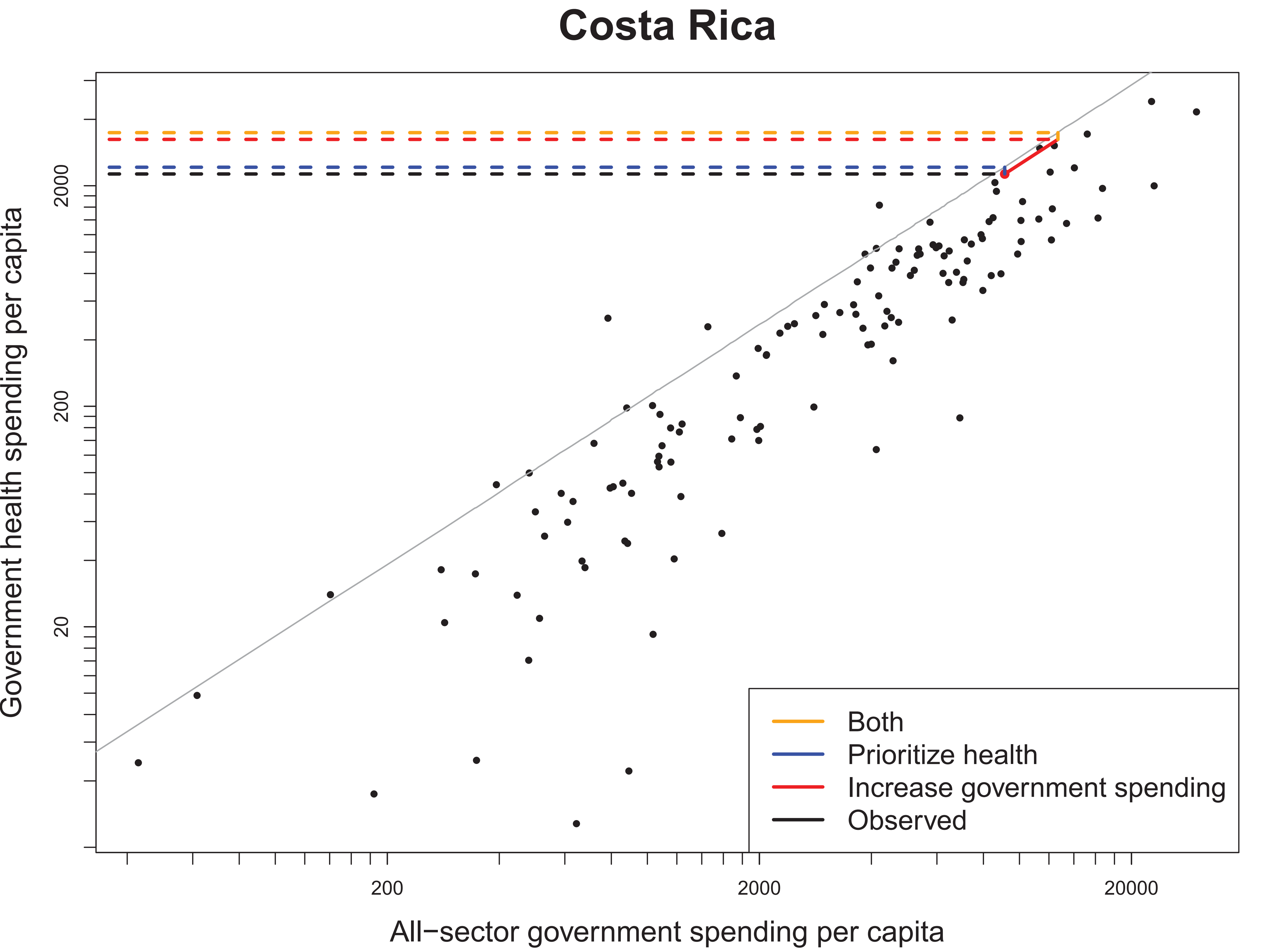
Costa Rica
Government health spending per capita
All−sector government spending per capita
20
200
2000
20000
Both
Prioritize health
Increase government spending
Observed

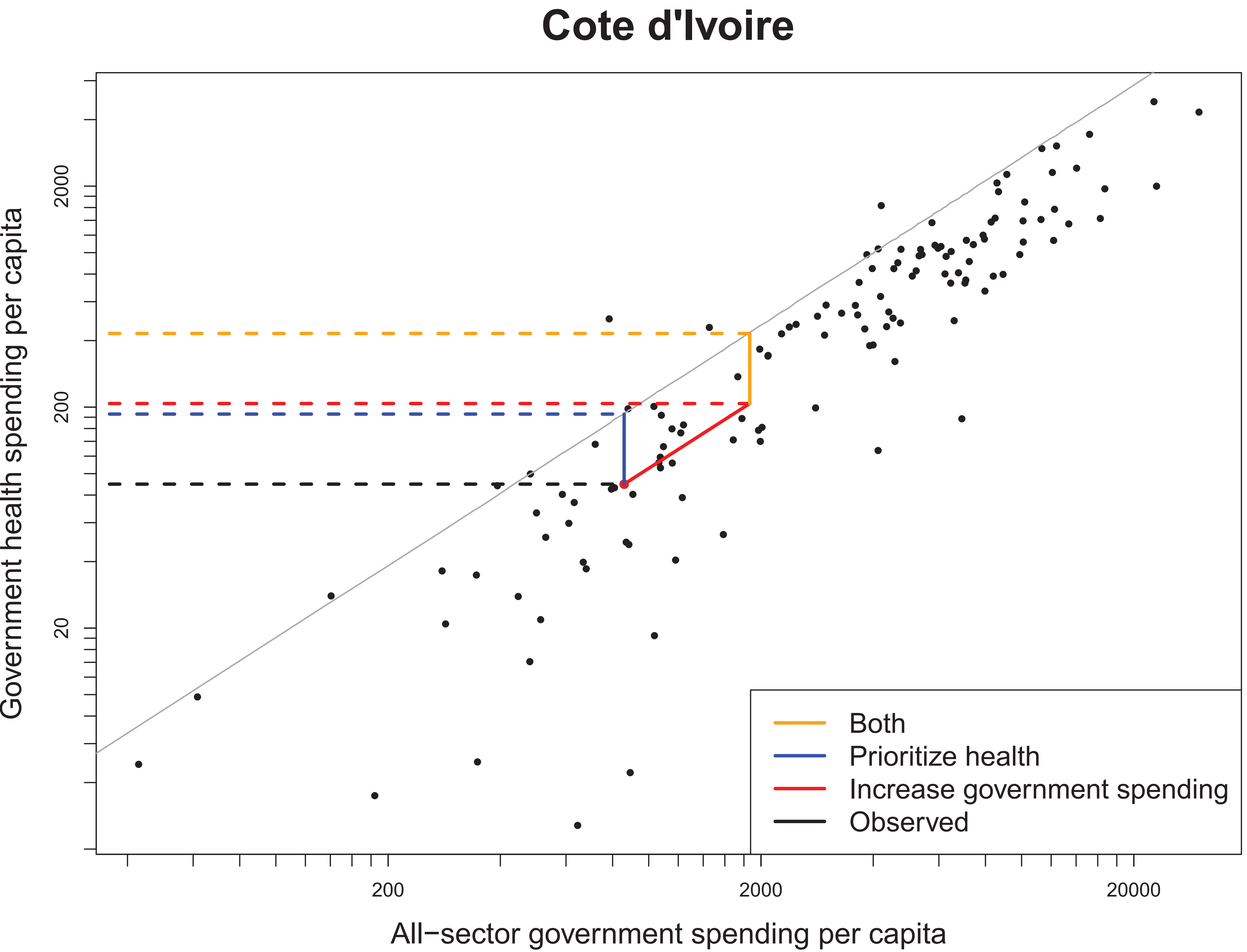

Cote d'Ivoire
Government health spending per capita
20
200
2000
All−sector government spending per capita
200
2000
20000
Both
Prioritize health
Increase government spending
Observed

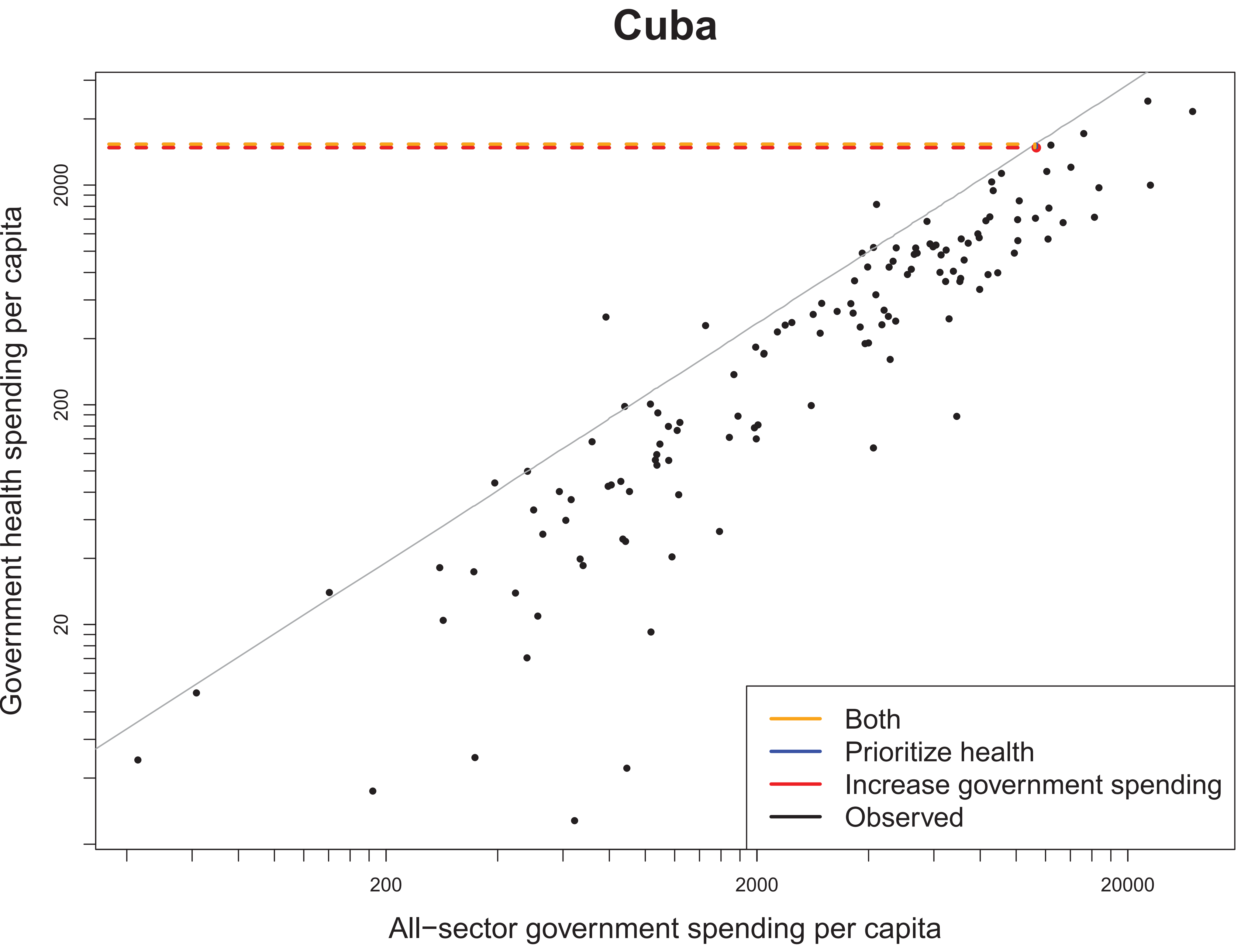

Cuba
Government health spending per capita
2000
200
20
All−sector government spending per capita
200
2000
20000
Both
Prioritize health
Increase government spending
Observed

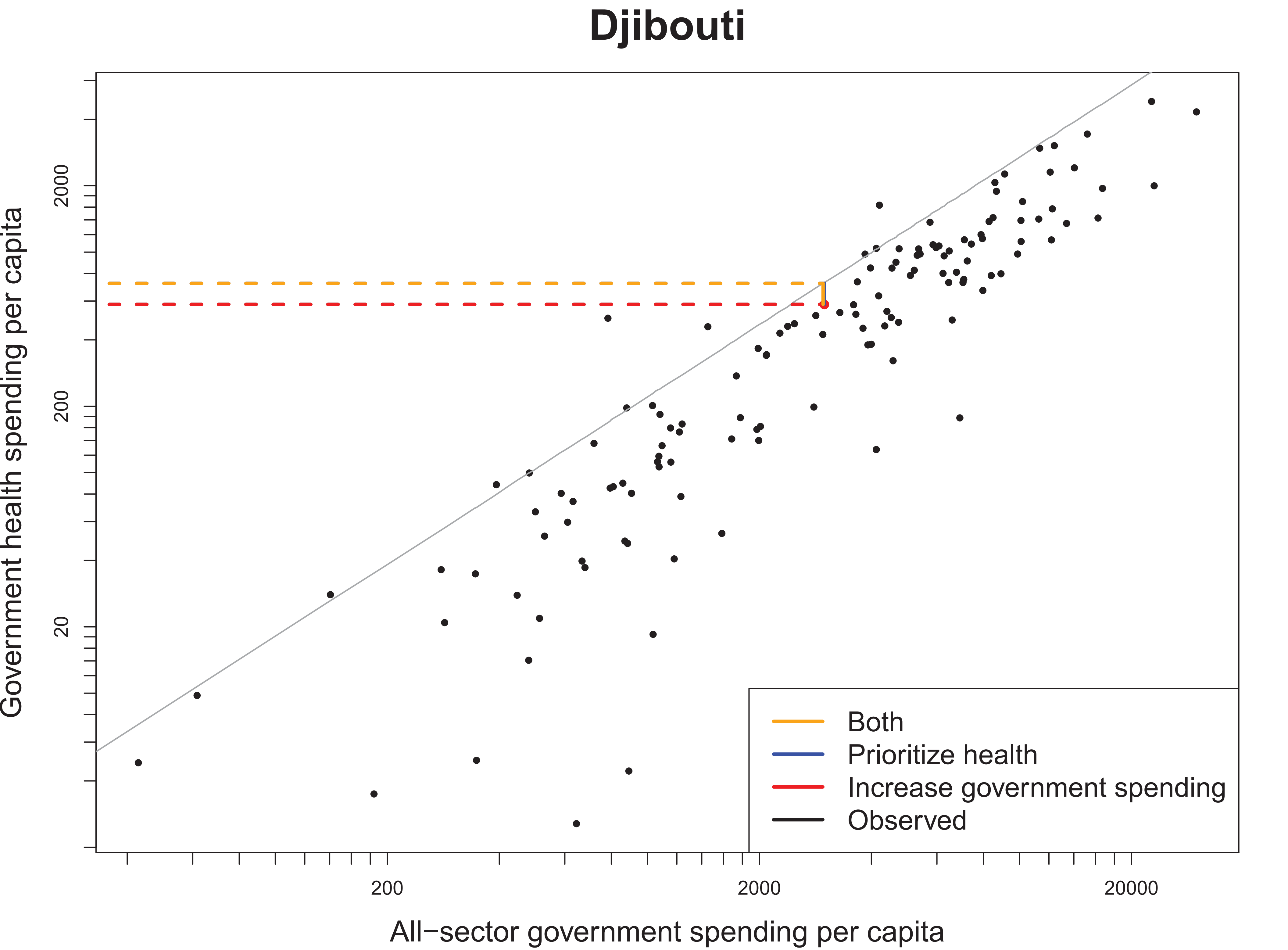
Djibouti
Government health spending per capita
20
200
2000
200
2000
20000
All−sector government spending per capita
Both
Prioritize health
Increase government spending
Observed

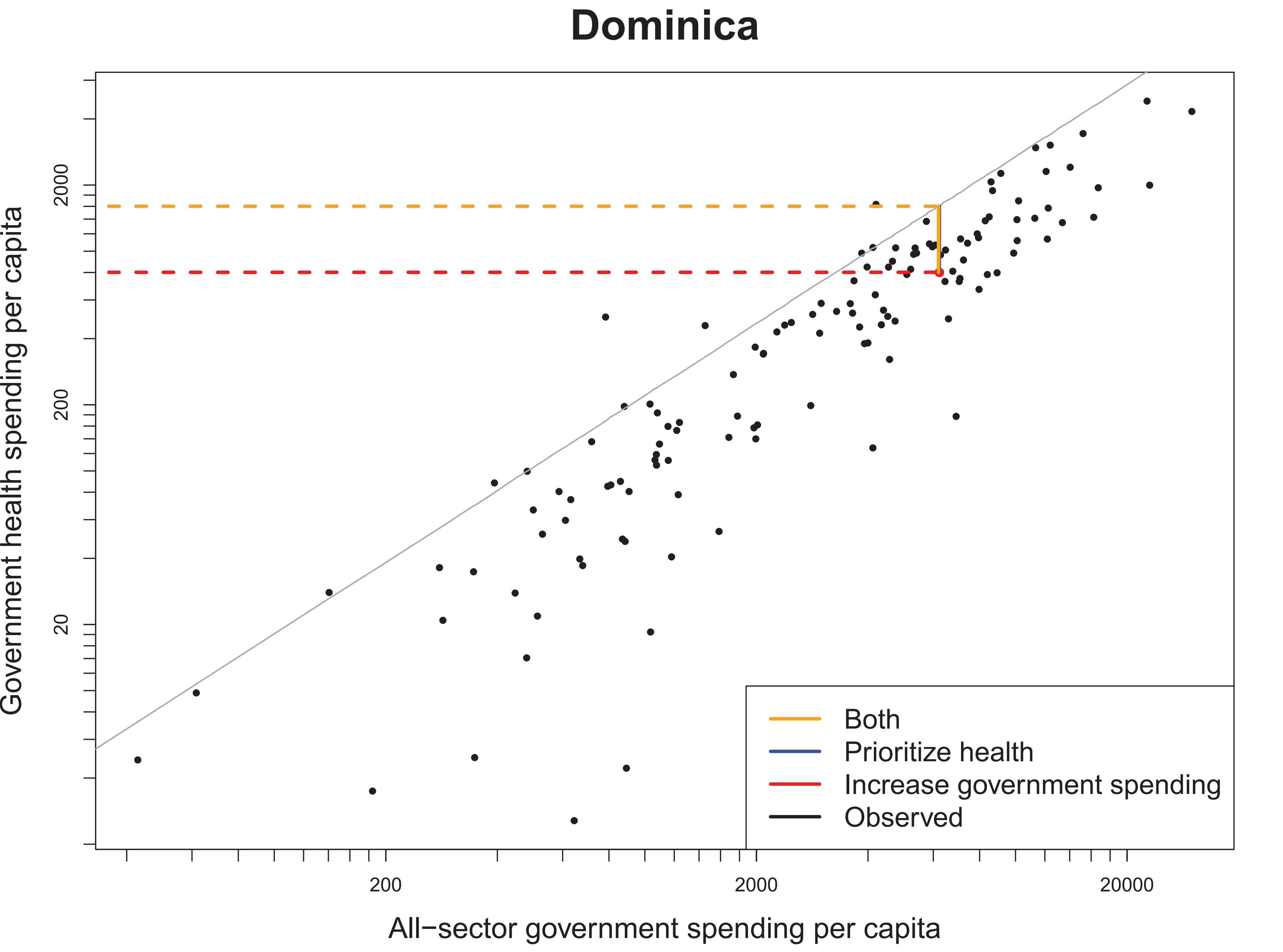

Dominica
Government health spending per capita
All−sector government spending per capita
20
200
2000
200
2000
20000
Both
Prioritize health
Increase government spending
Observed

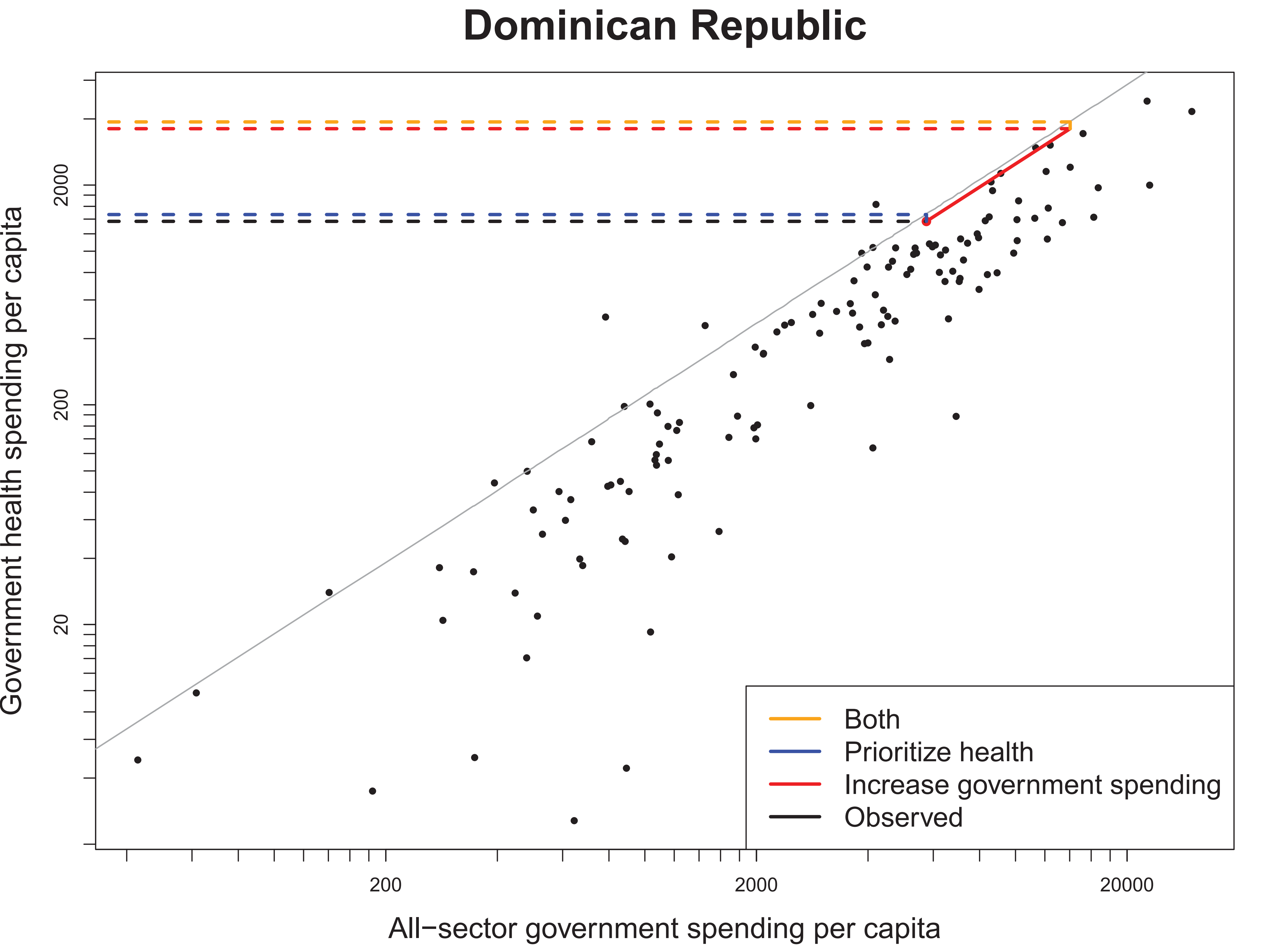

Dominican Republic
Government health spending per capita
All−sector government spending per capita
20
200
2000
200
2000
20000
Both
Prioritize health
Increase government spending
Observed

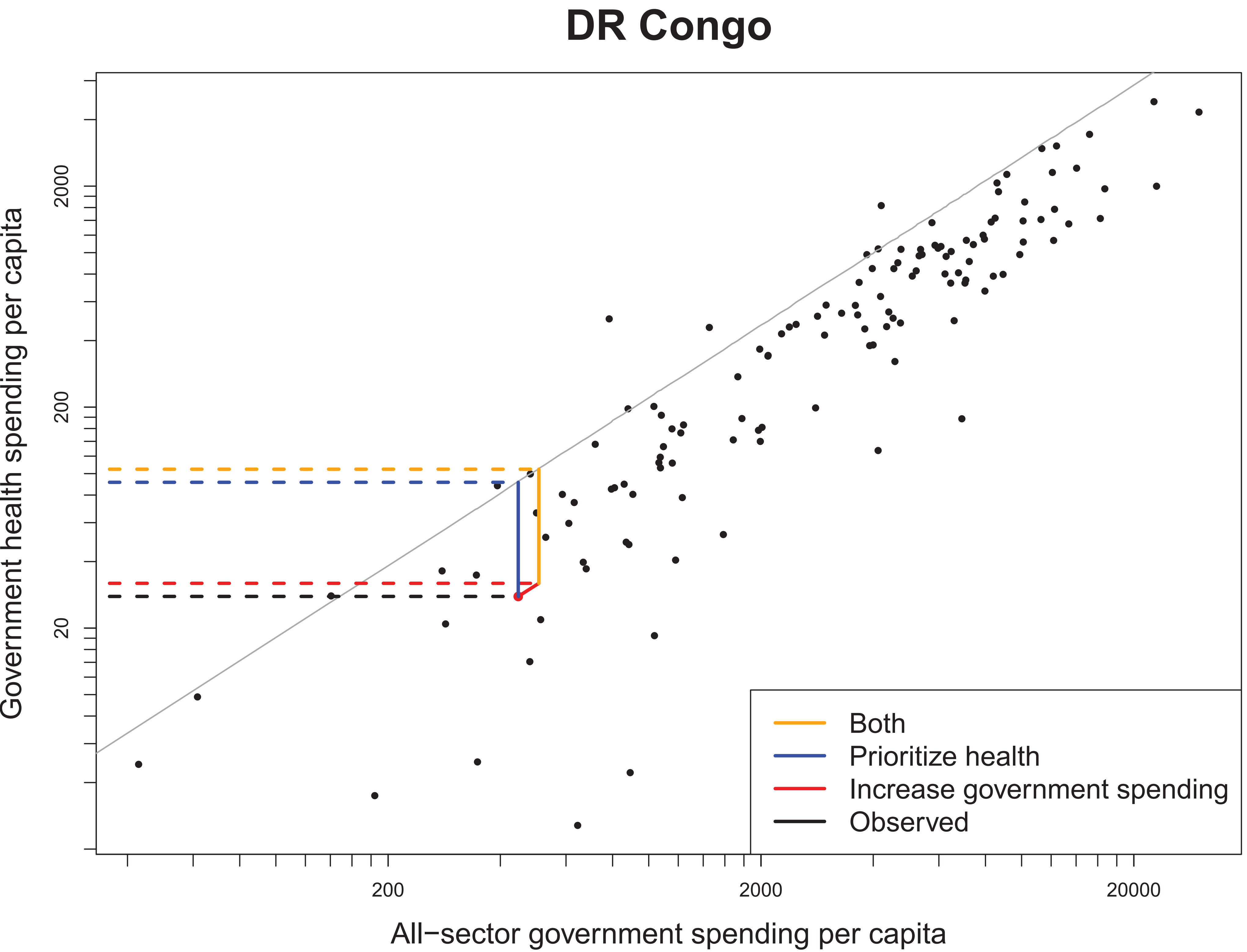

DR Congo
Government health spending per capita
20
200
2000
All−sector government spending per capita
200
2000
20000
Both
Prioritize health
Increase government spending
Observed

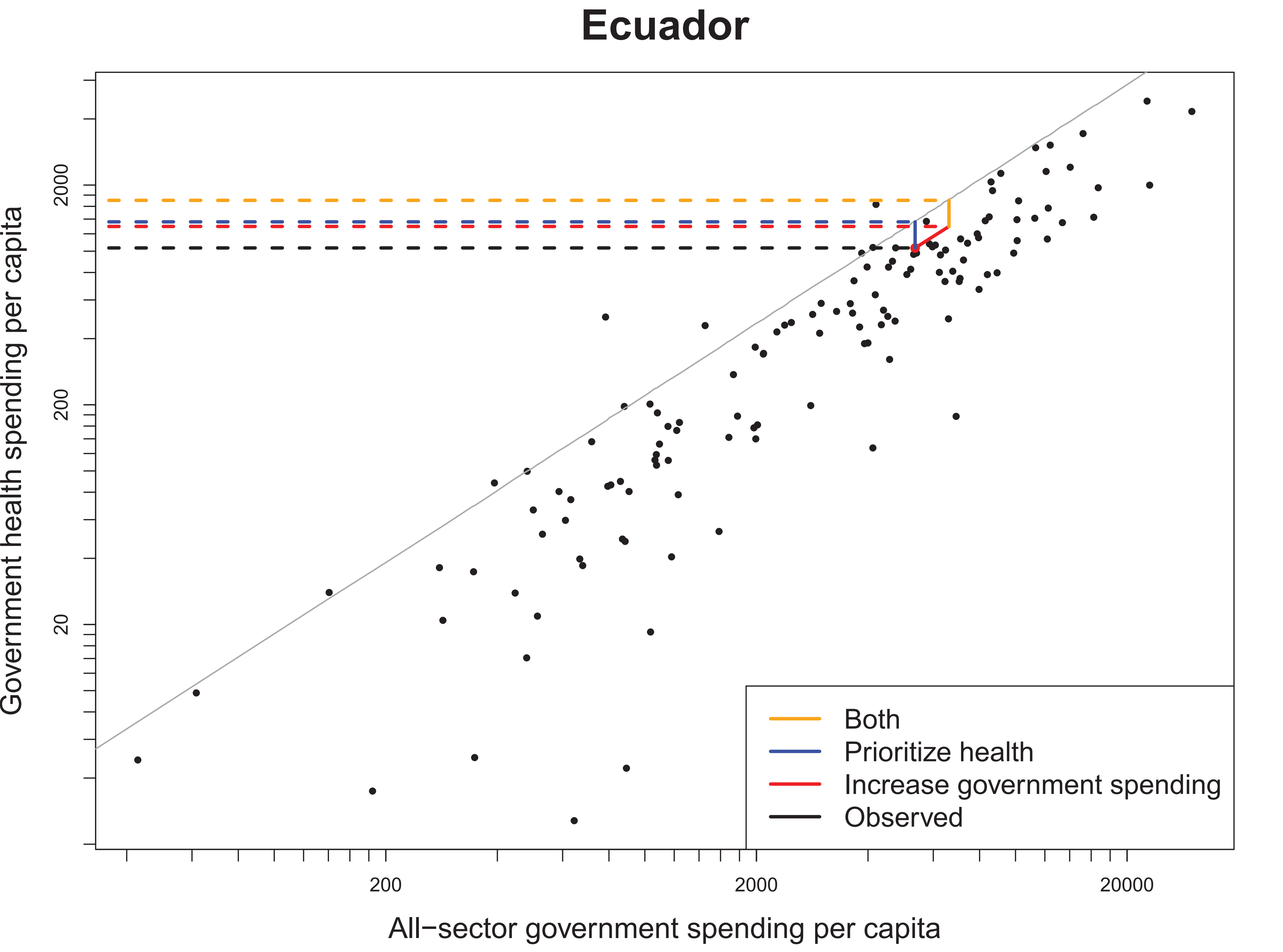
Ecuador
Government health spending per capita
All−sector government spending per capita
20
200
2000
20000
Both
Prioritize health
Increase government spending
Observed

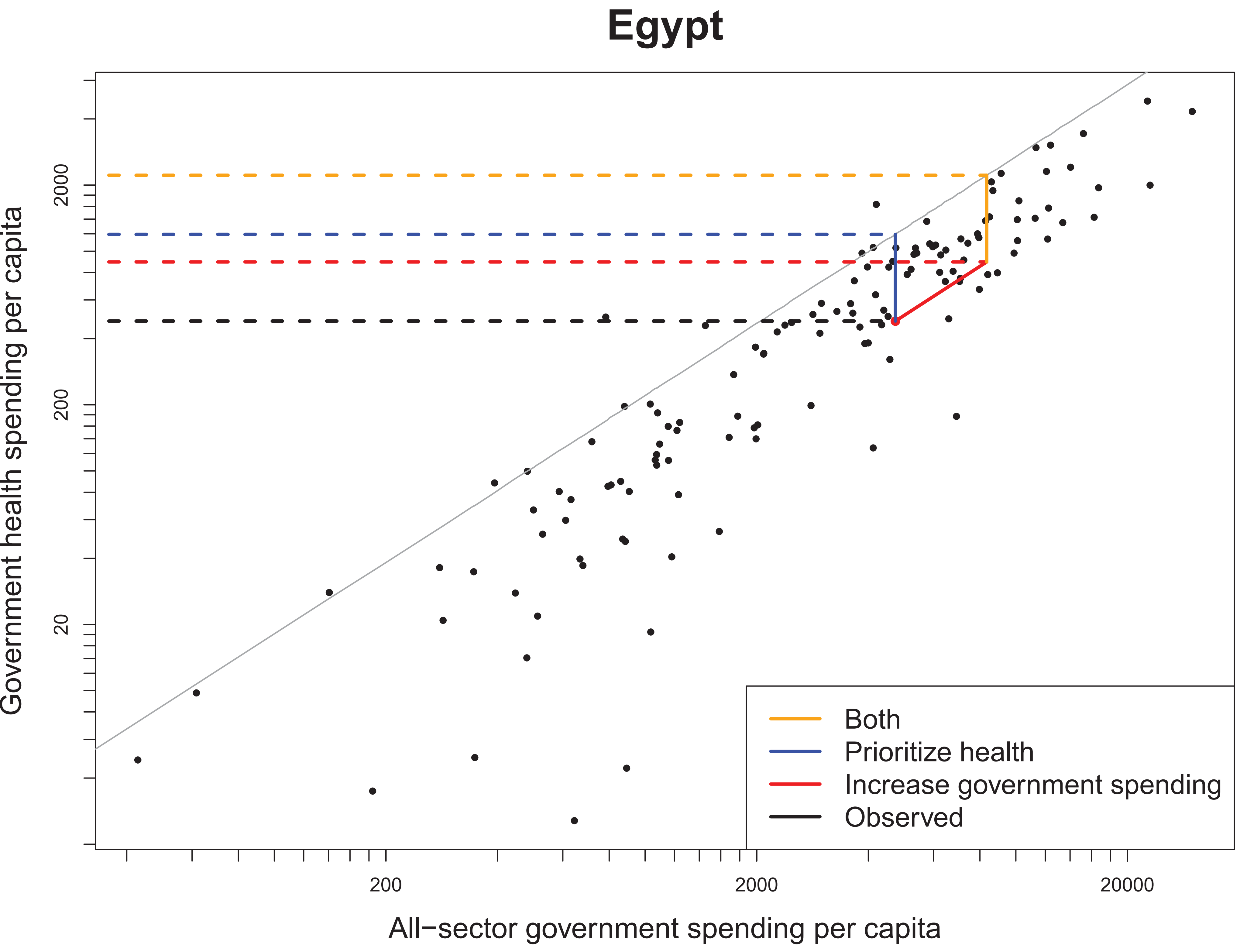

Egypt
Government health spending per capita
All−sector government spending per capita
20
200
2000
200
2000
20000
Both
Prioritize health
Increase government spending
Observed

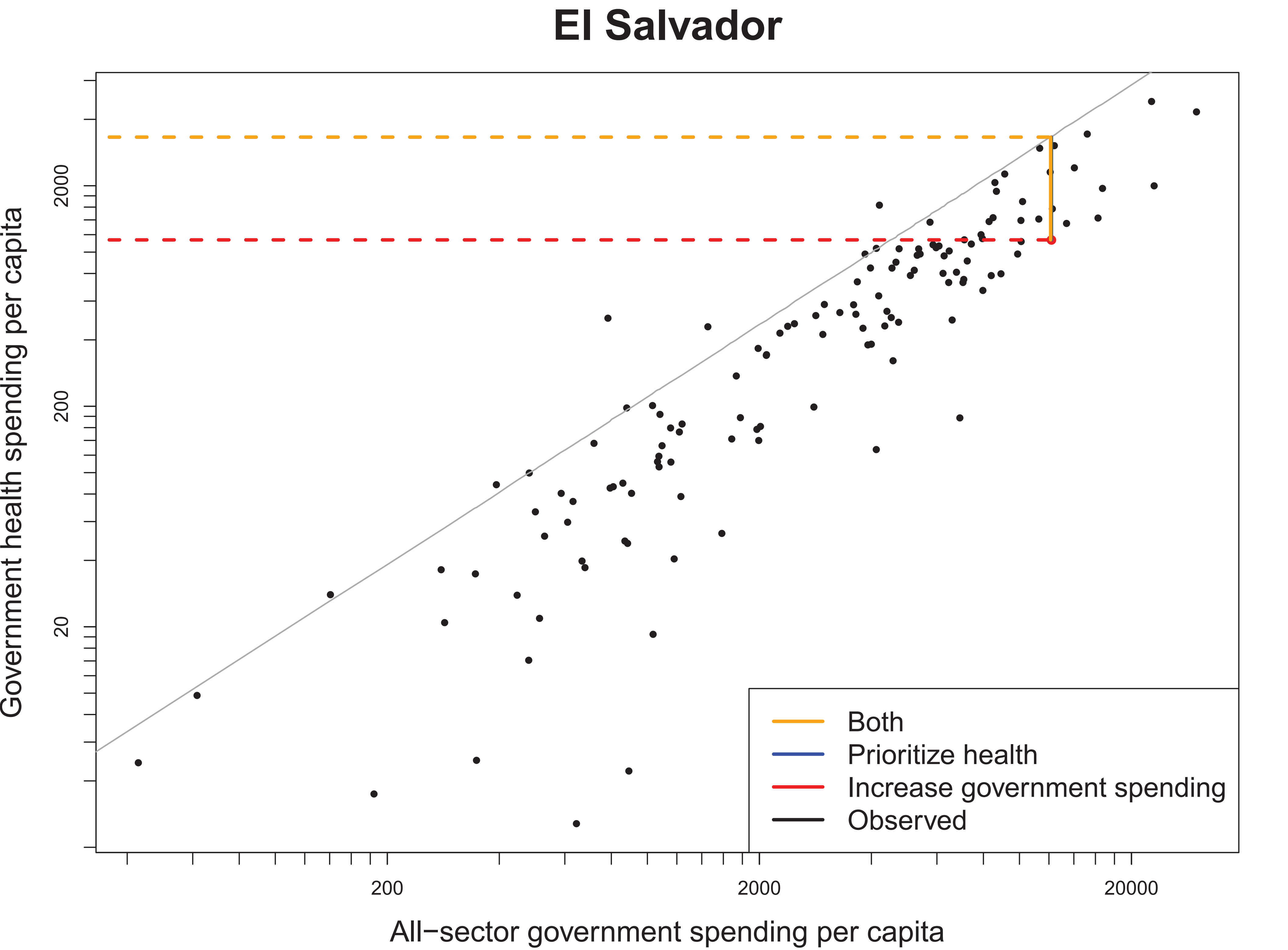

El Salvador
Both
Prioritize health
Increase government spending
Observed
Government health spending per capita
20
200
2000
All−sector government spending per capita
200
2000
20000

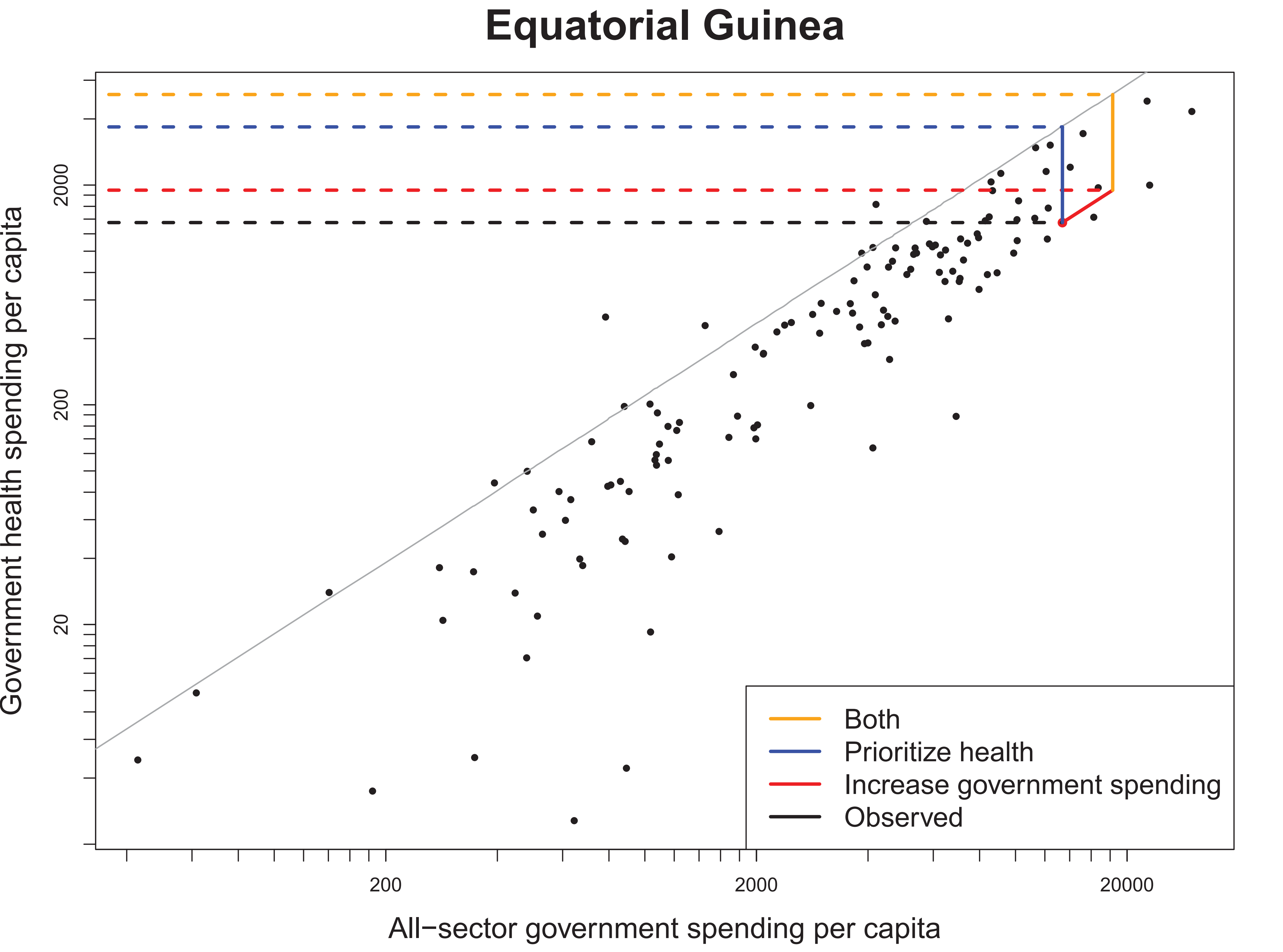
Equatorial Guinea
Government health spending per capita
20
200
2000
200
2000
20000
All−sector government spending per capita
Both
Prioritize health
Increase government spending
Observed

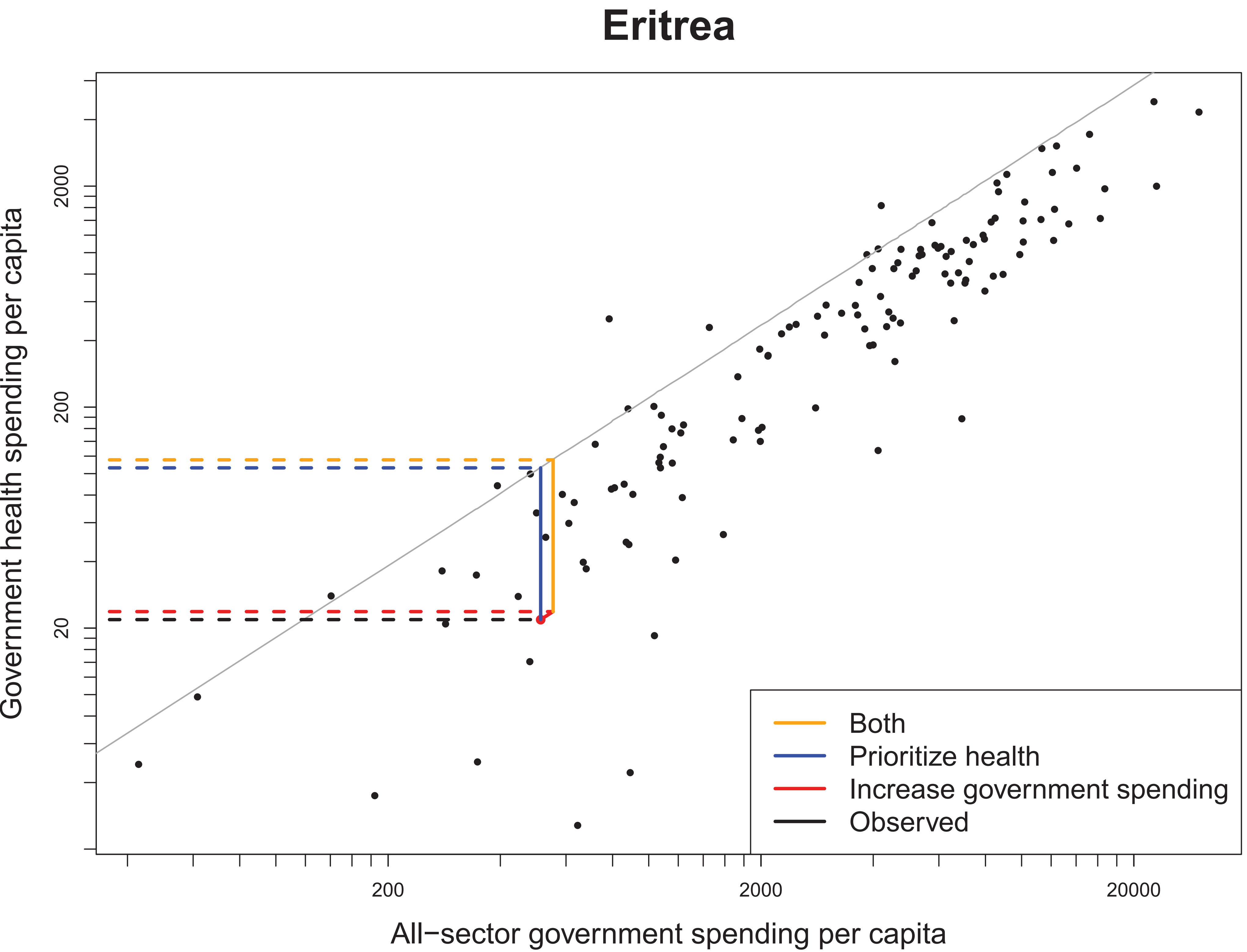

Eritrea
Government health spending per capita
All−sector government spending per capita
20
200
2000
20000
Both
Prioritize health
Increase government spending
Observed

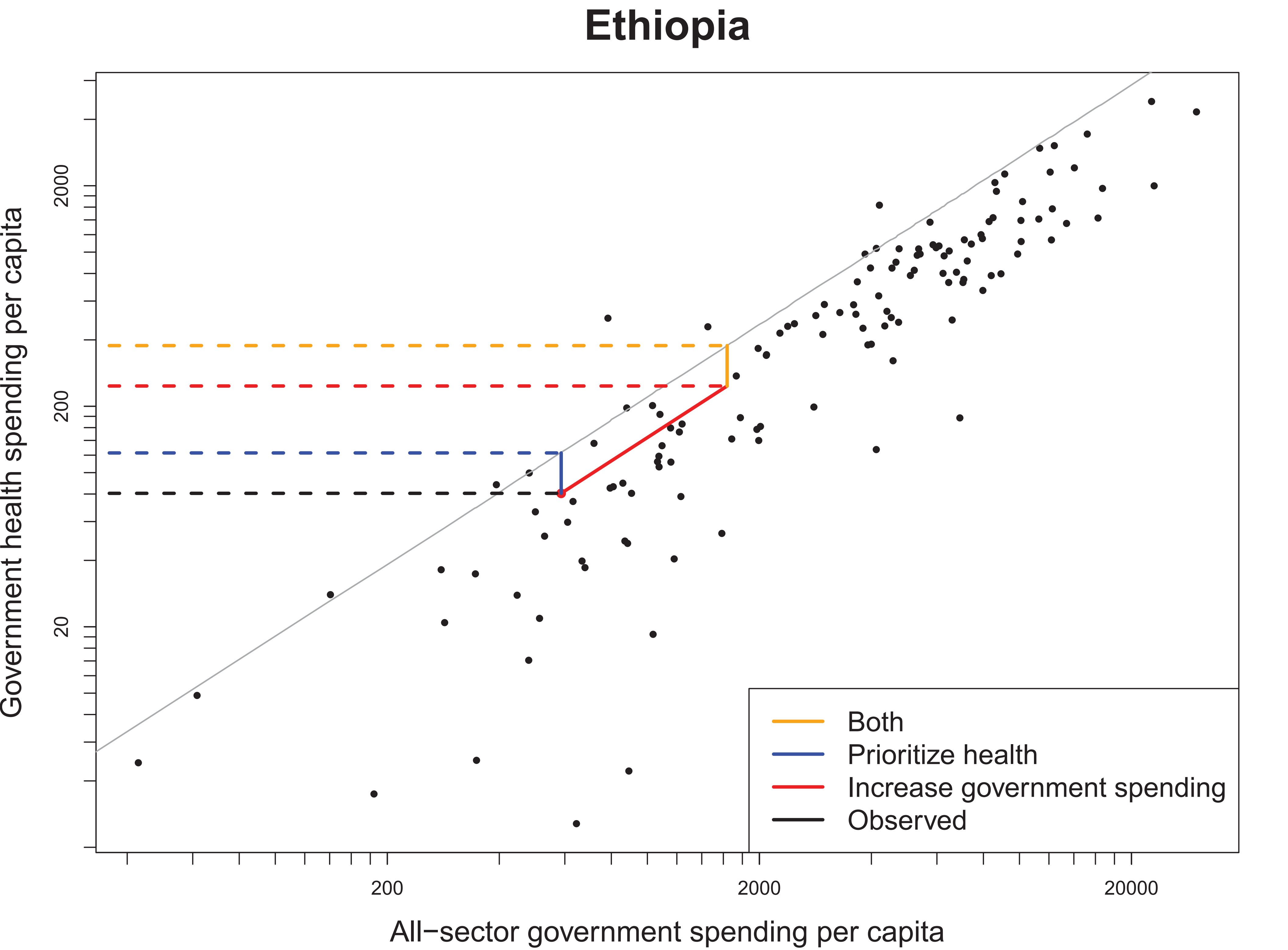

Ethiopia
Government health spending per capita
2000
200
20
All−sector government spending per capita
200
2000
20000
Both
Prioritize health
Increase government spending
Observed

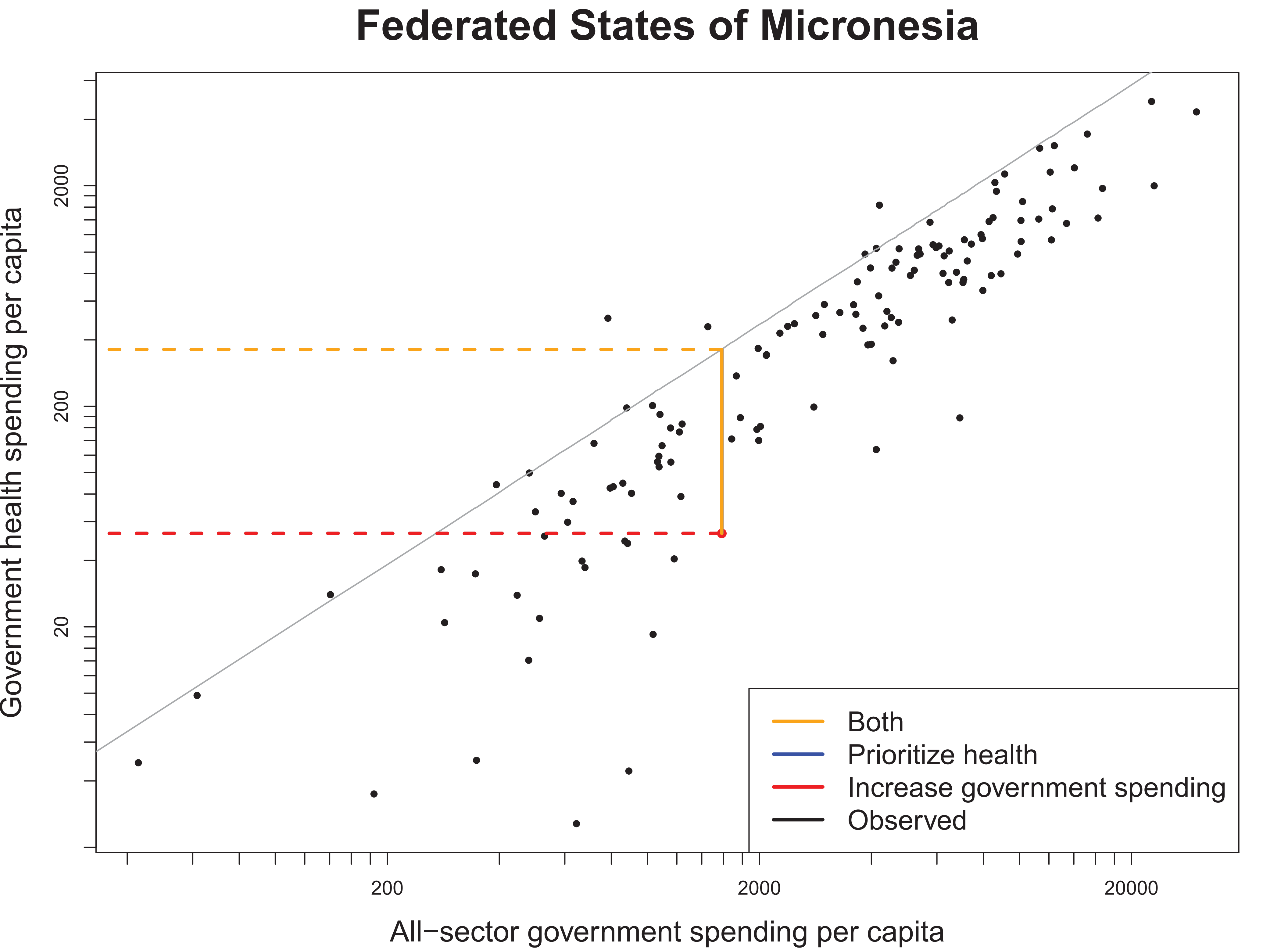

Federated States of Micronesia
Government health spending per capita
20
200
2000
All−sector government spending per capita
200
2000
20000
Both
Prioritize health
Increase government spending
Observed

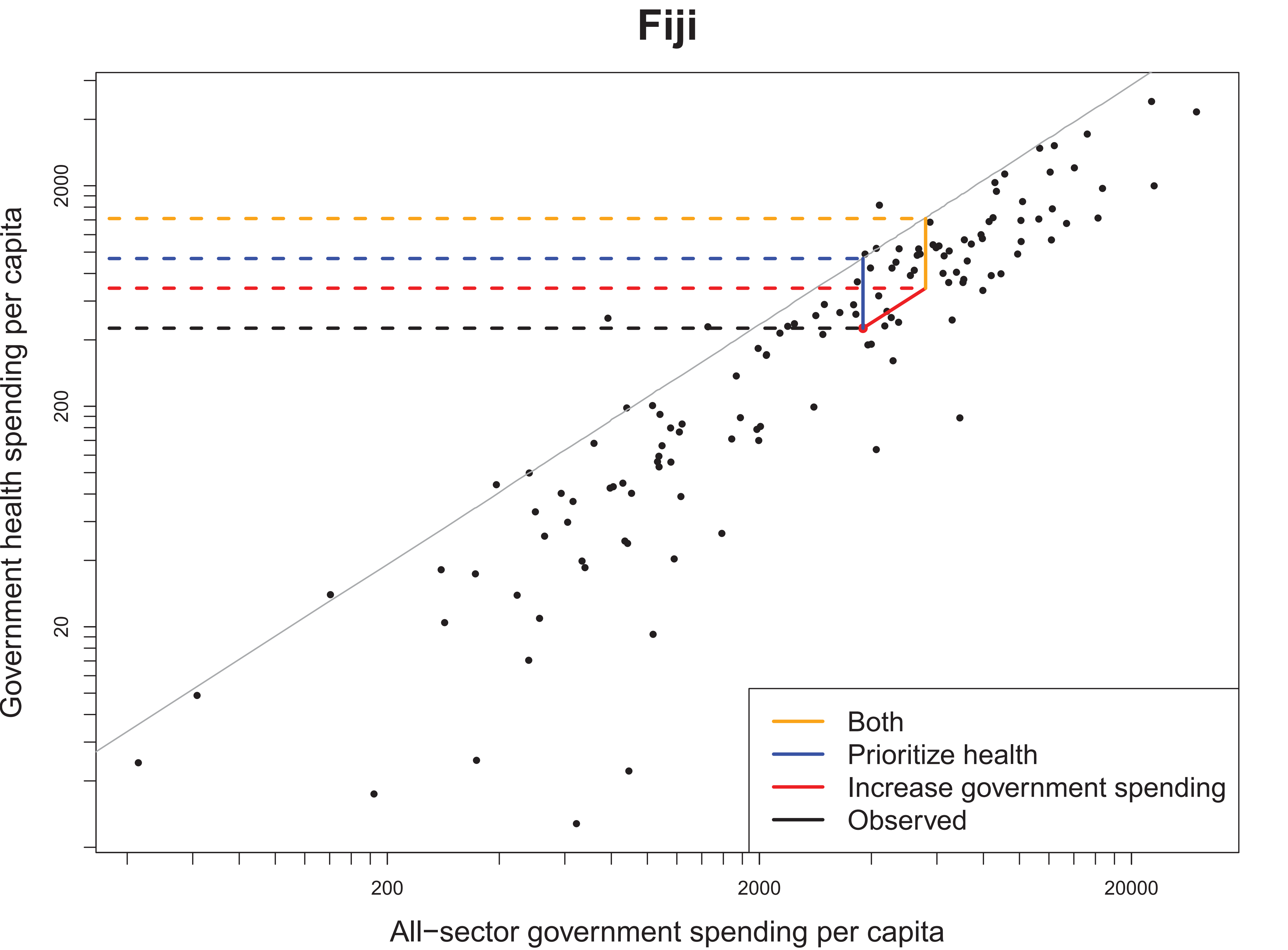

Fiji
Government health spending per capita
20
200
2000
All−sector government spending per capita
200
2000
20000
Both
Prioritize health
Increase government spending
Observed

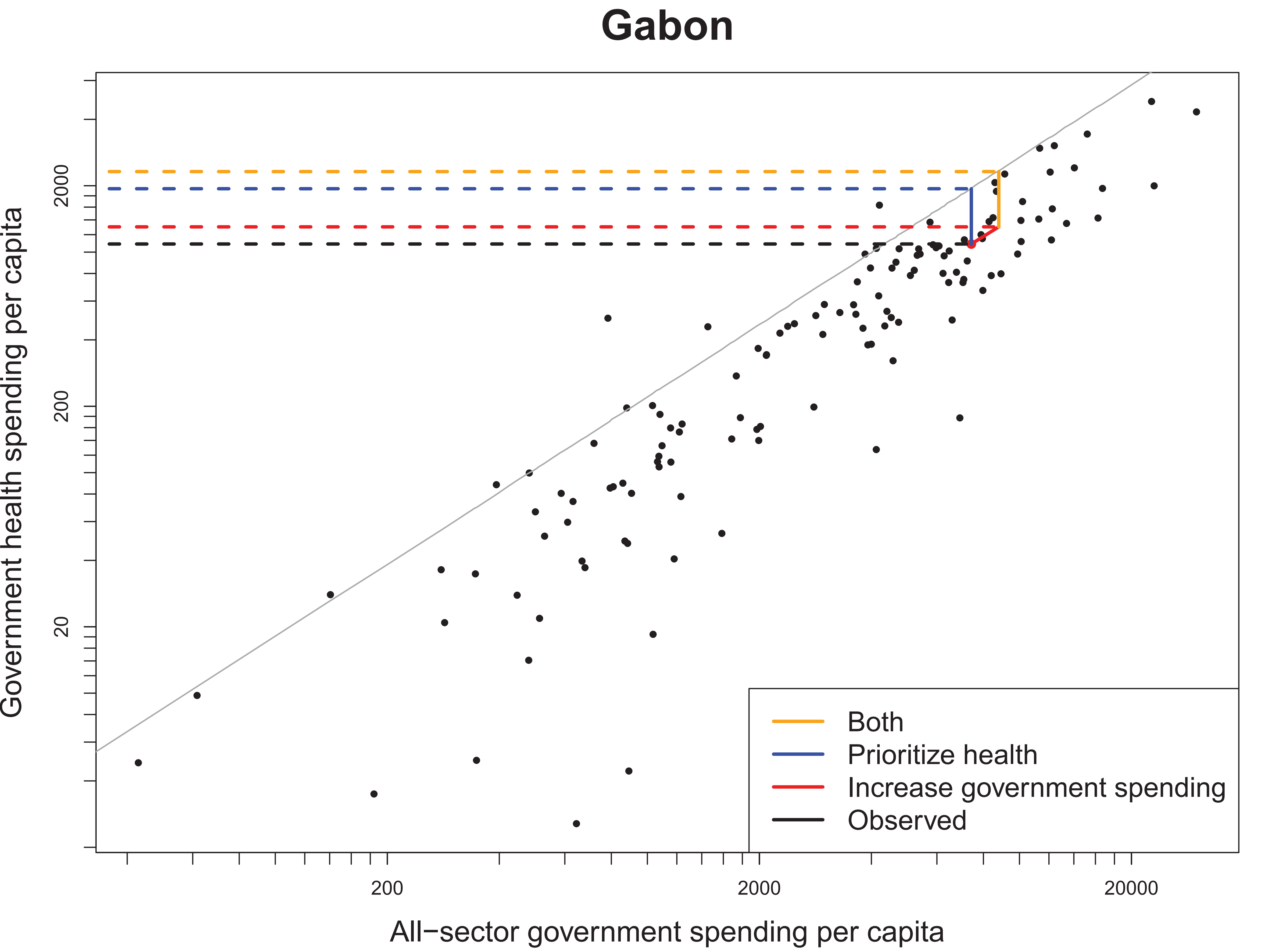

Gabon
Government health spending per capita
2000
200
20
All−sector government spending per capita
200
2000
20000
Both
Prioritize health
Increase government spending
Observed

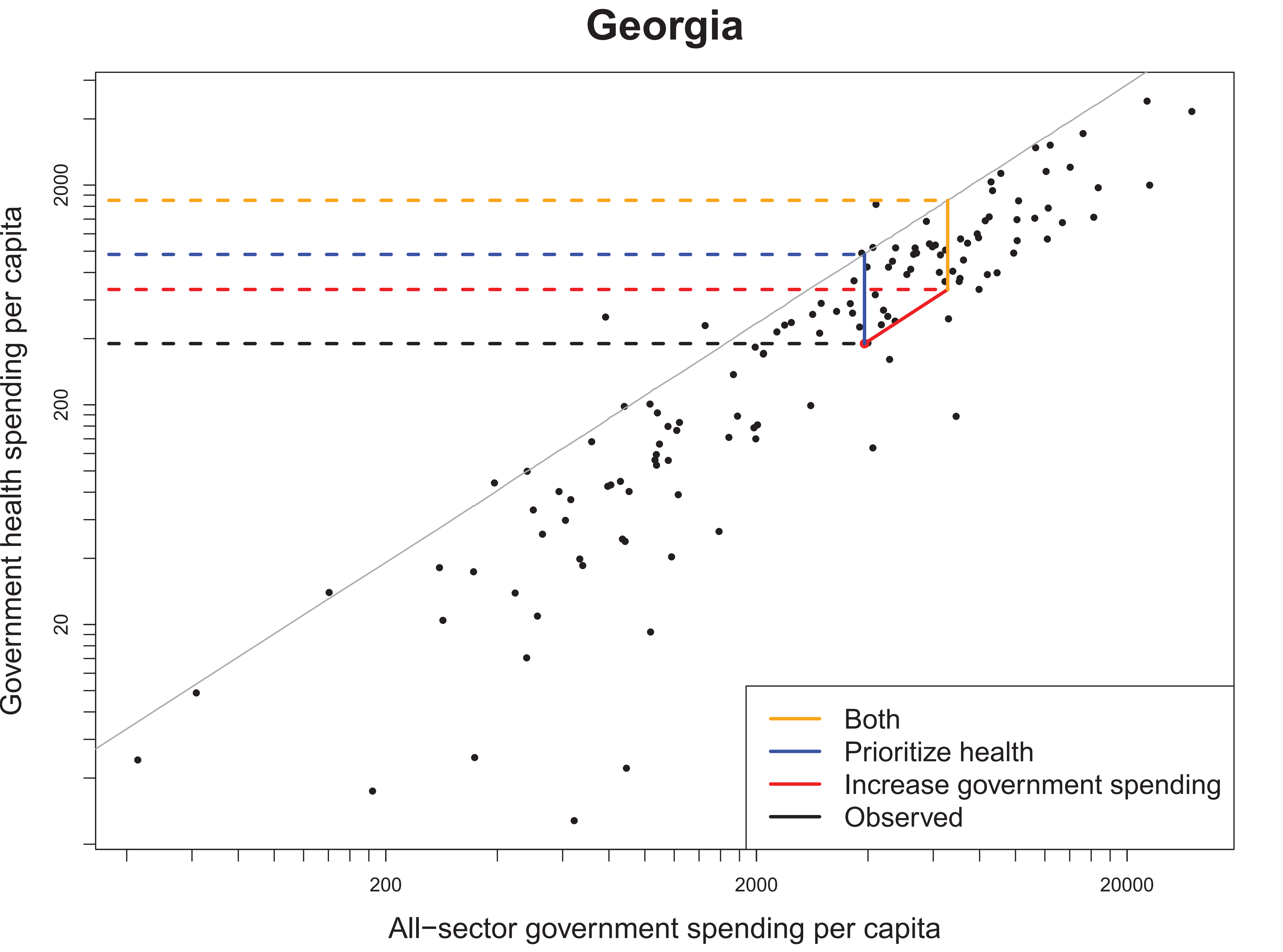
Georgia
Both
Prioritize health
Increase government spending
Observed
Government health spending per capita
20
200
2000
All−sector government spending per capita
200
2000
20000

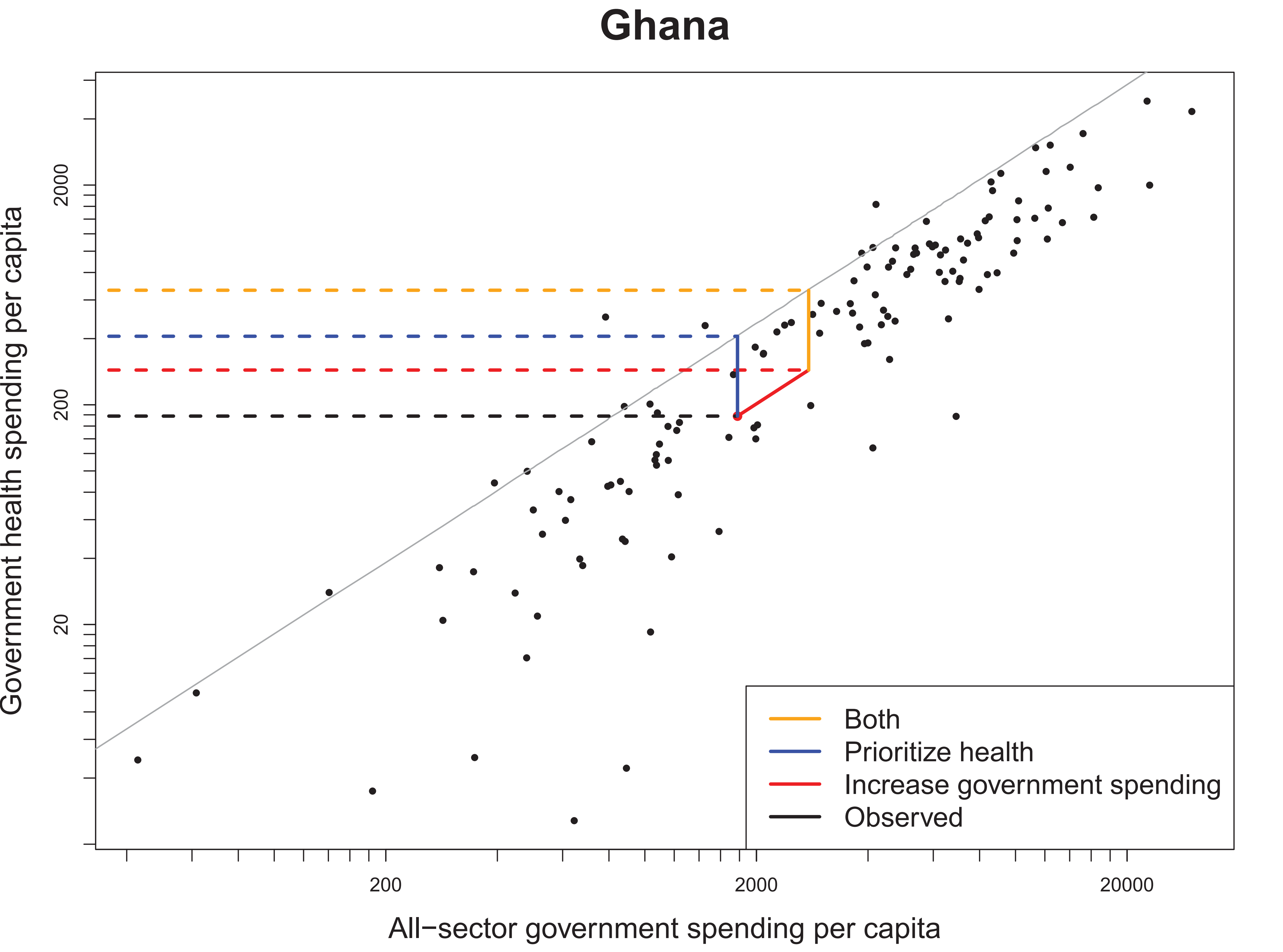

Ghana
Government health spending per capita
All−sector government spending per capita
20
200
2000
200
2000
20000
Both
Prioritize health
Increase government spending
Observed

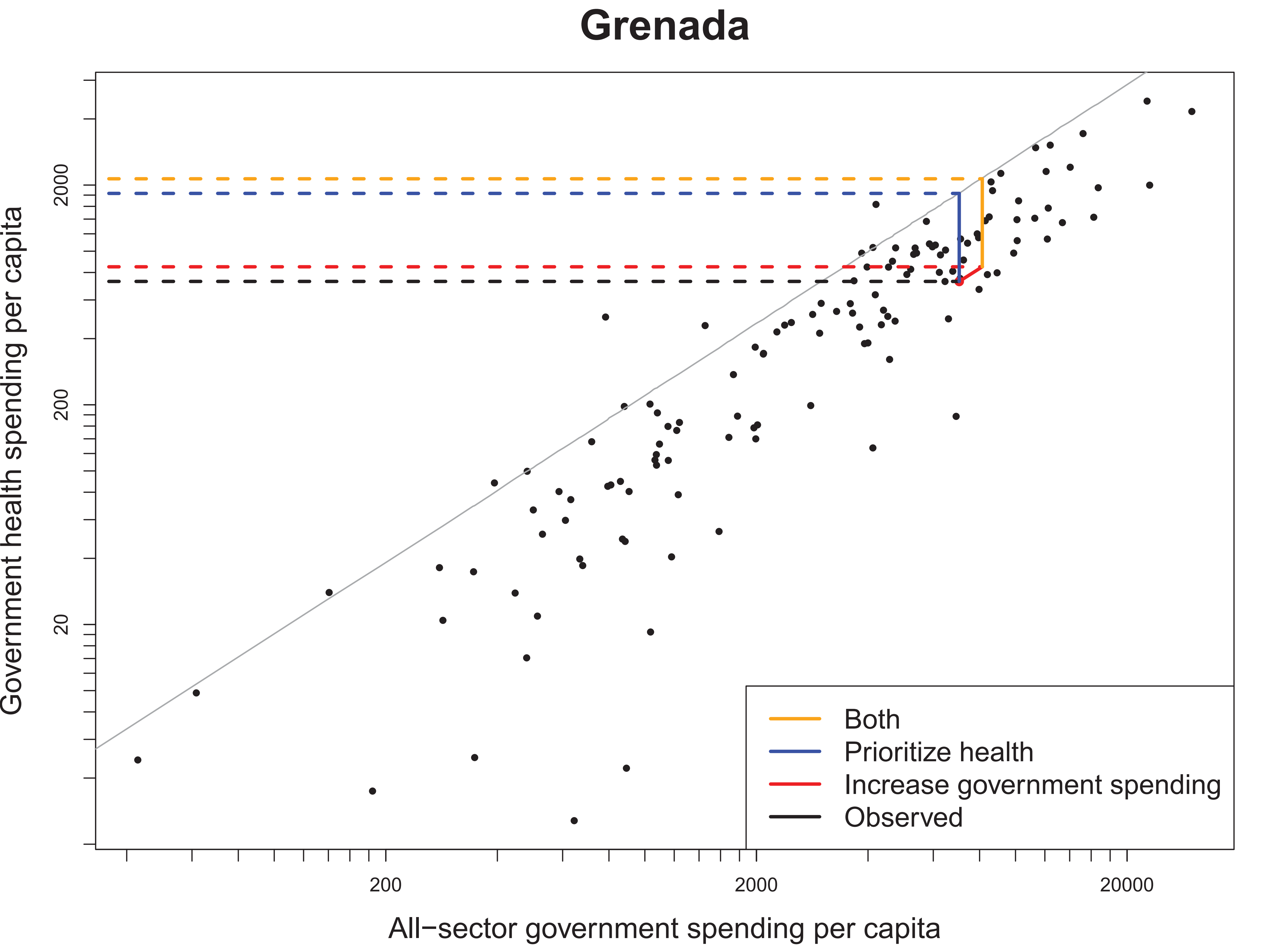
Grenada
Government health spending per capita
2000
200
20
All−sector government spending per capita
200
2000
20000
Both
Prioritize health
Increase government spending
Observed

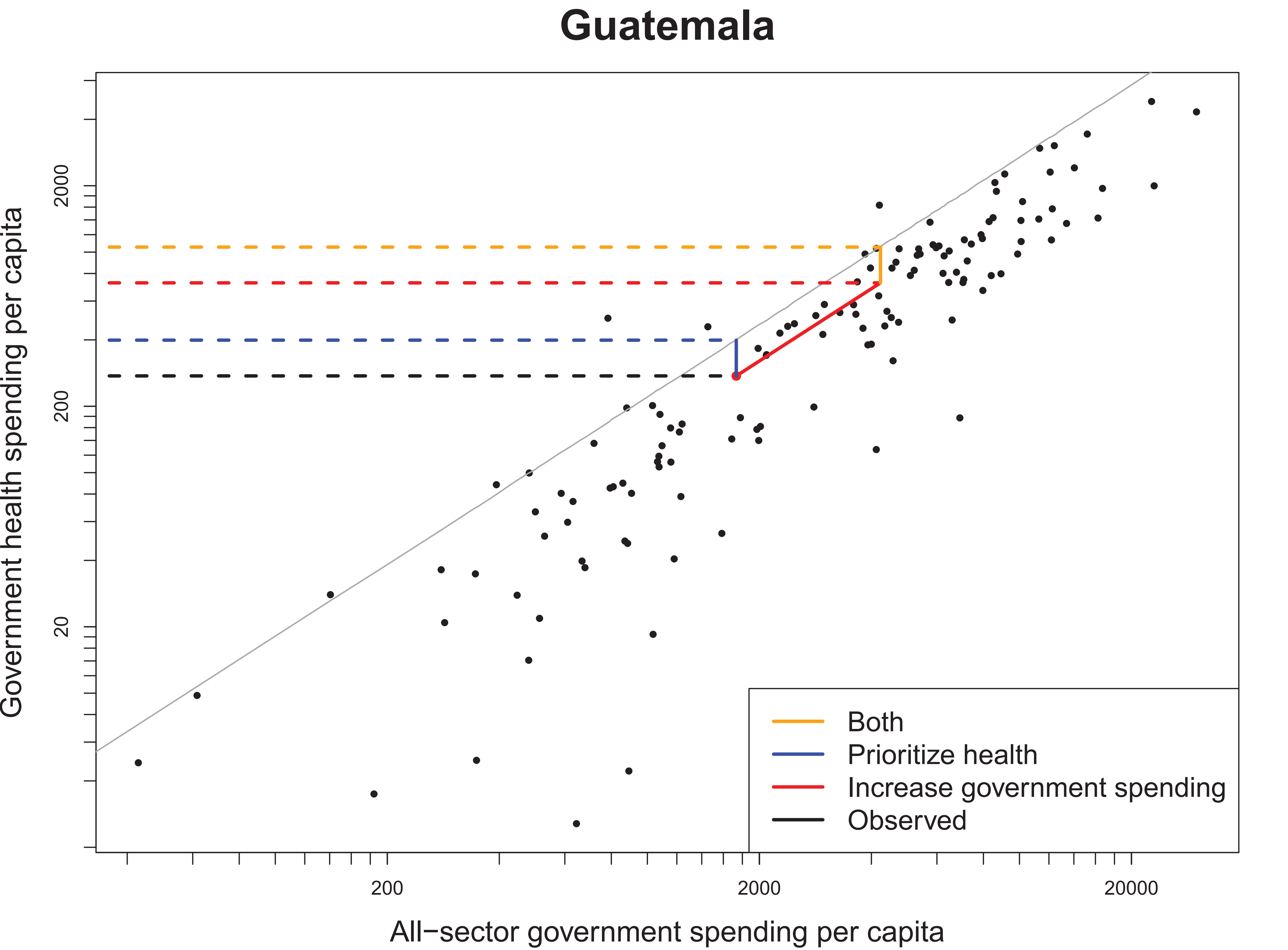
Guatemala
Government health spending per capita
20
200
2000
All−sector government spending per capita
200
2000
20000
Both
Prioritize health
Increase government spending
Observed

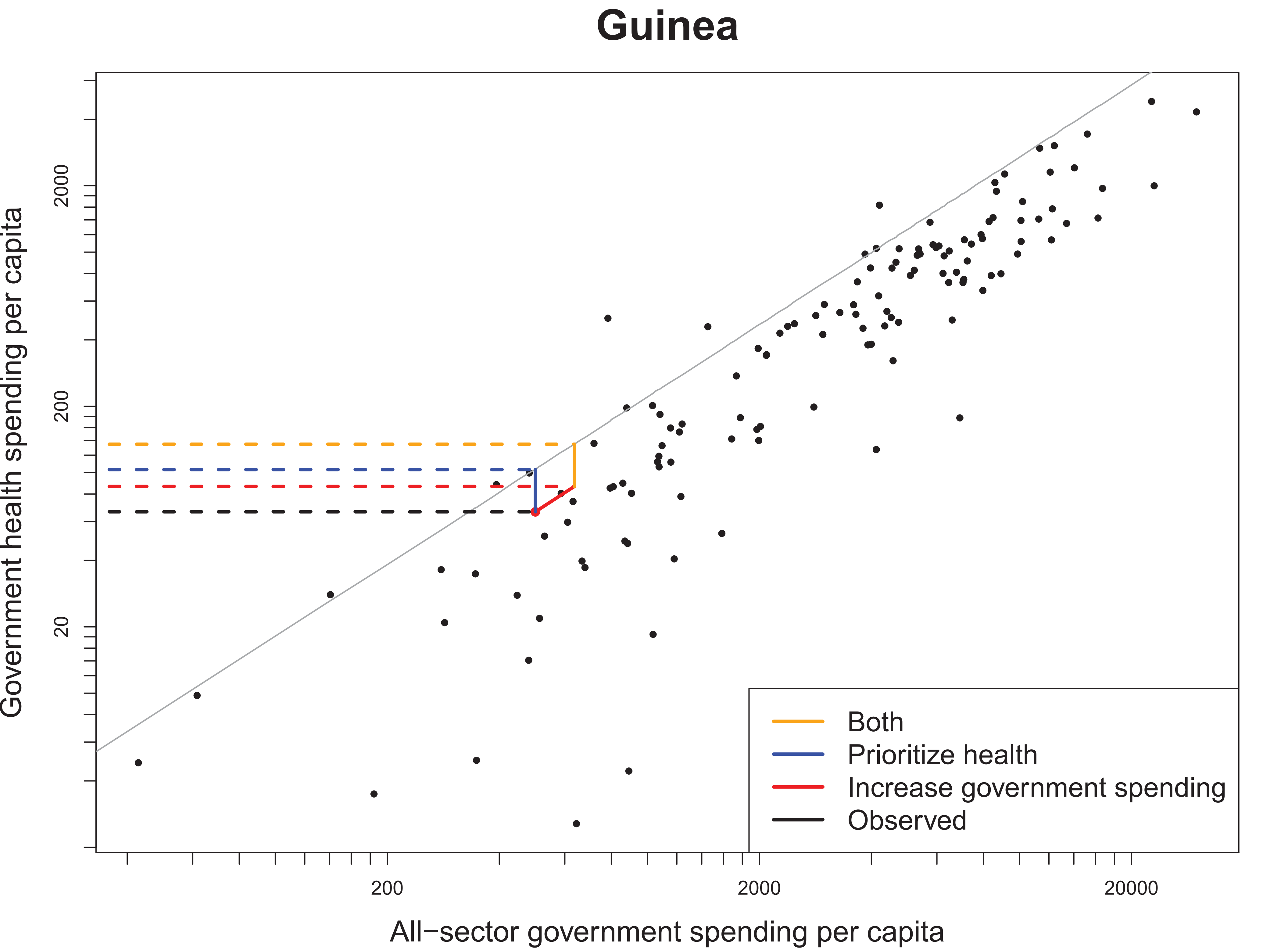

Guinea
2000
200
20
200
2000
20000
Government health spending per capita
All−sector government spending per capita
Both
Prioritize health
Increase government spending
Observed

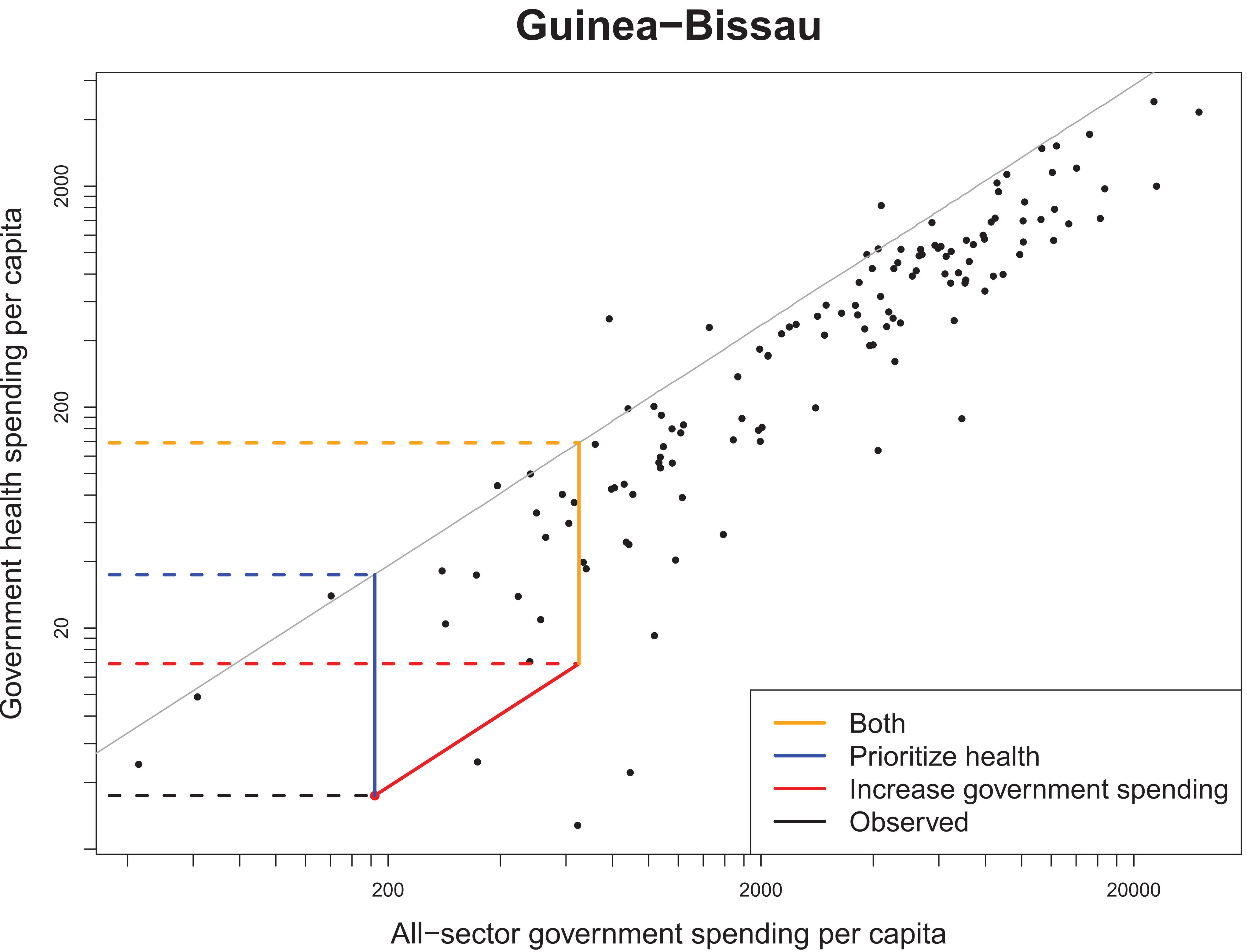

Guinea−Bissau
Government health spending per capita
20
200
2000
All−sector government spending per capita
200
2000
20000
Both
Prioritize health
Increase government spending
Observed

# Guyana

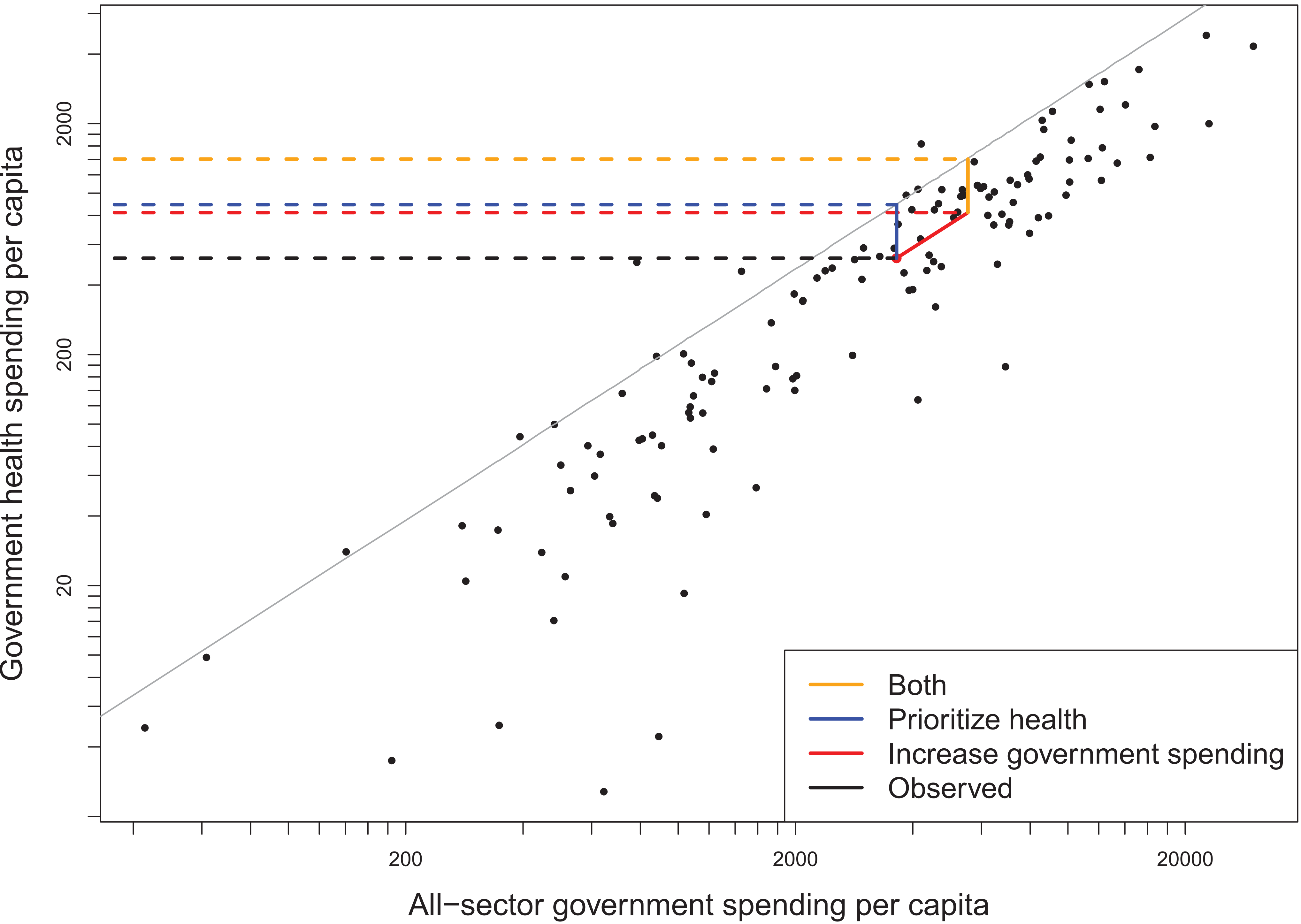

Government health spending per capita
2000
200
20
200
2000
20000
All−sector government spending per capita
Both
Prioritize health
Increase government spending
Observed

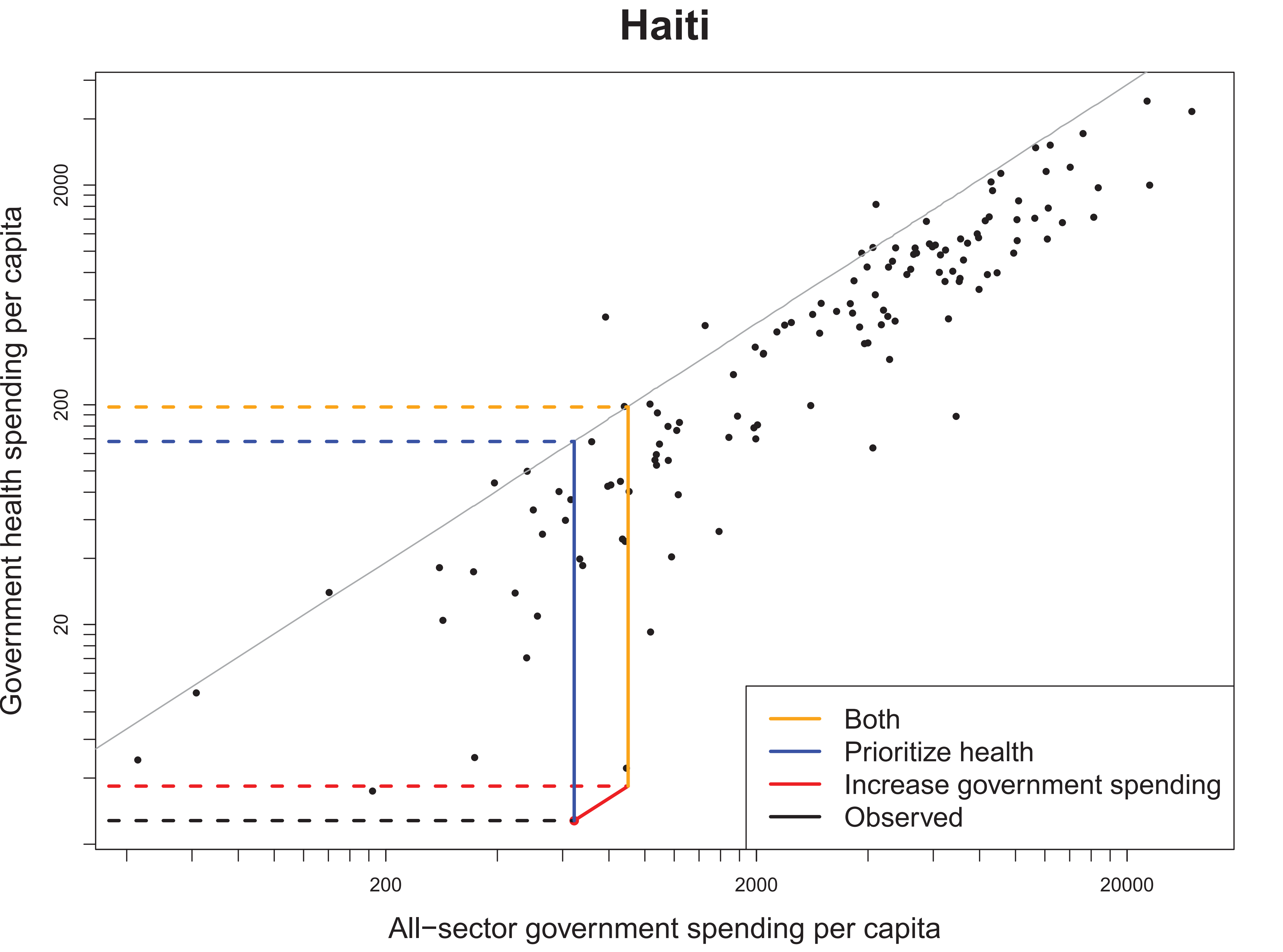

Haiti
Both
Prioritize health
Increase government spending
Observed
Government health spending per capita
20
200
2000
All−sector government spending per capita
200
2000
20000

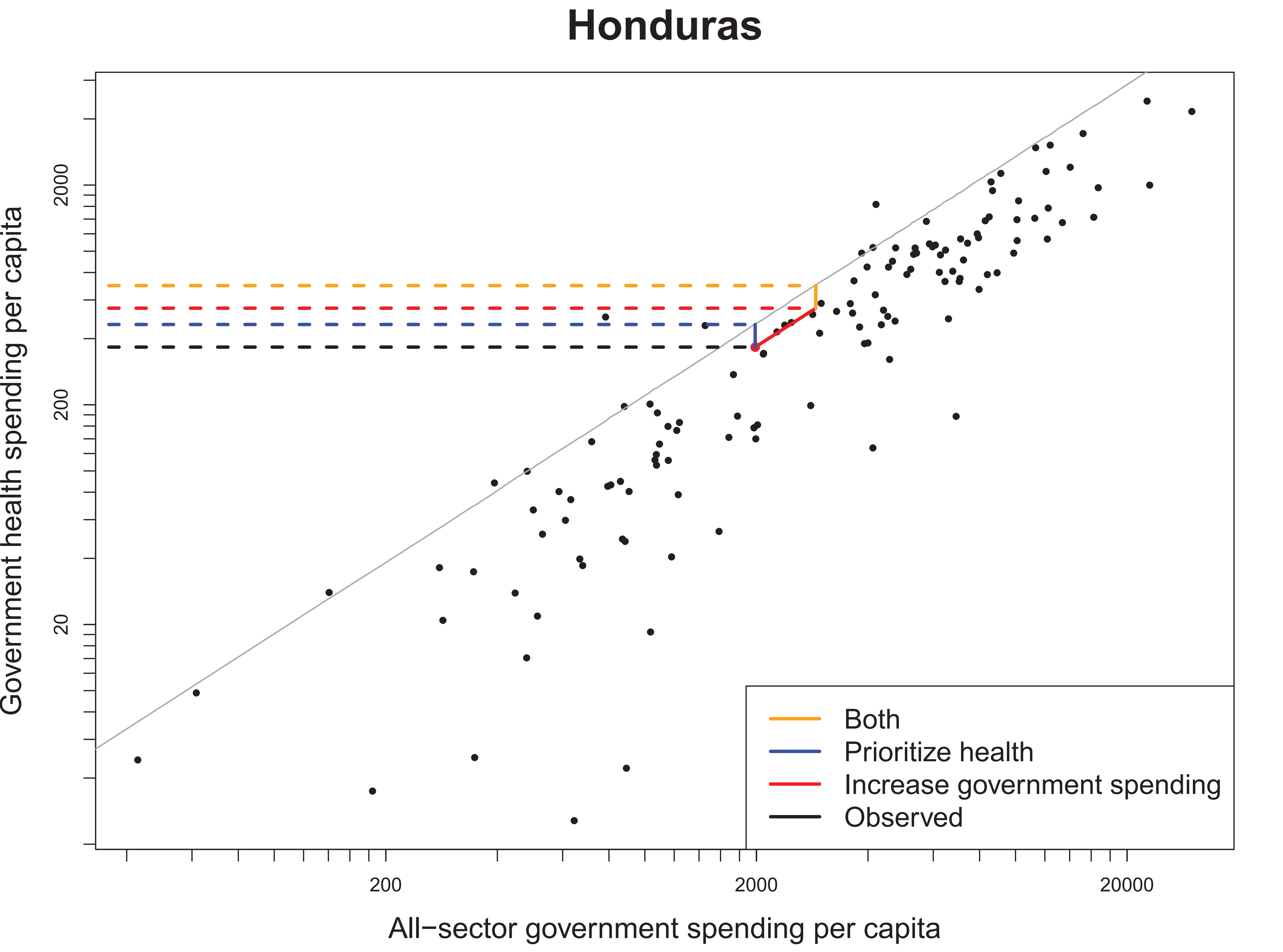

Honduras
Government health spending per capita
All−sector government spending per capita
20
200
2000
200
2000
20000
Both
Prioritize health
Increase government spending
Observed

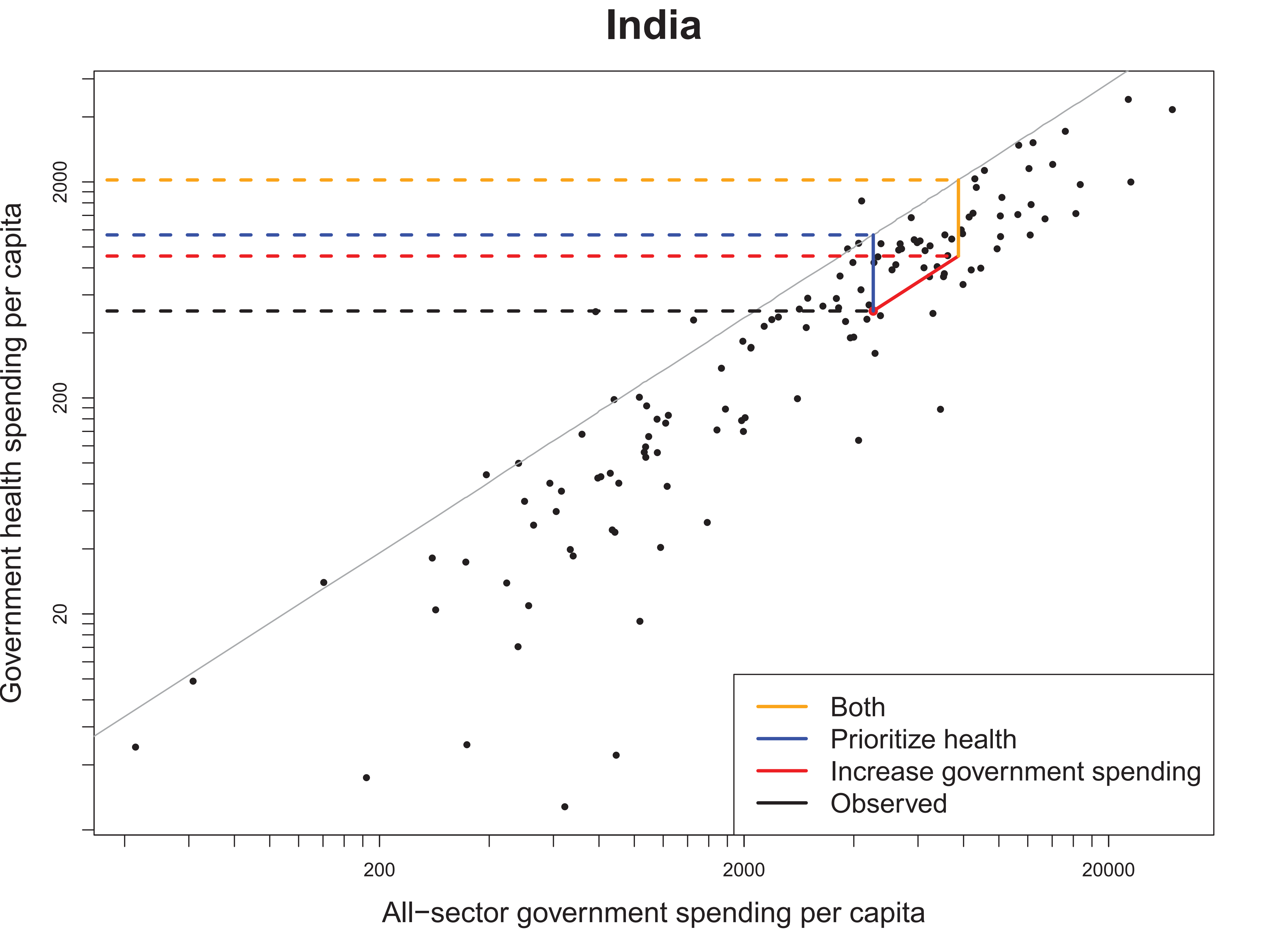

India
Government health spending per capita
All−sector government spending per capita
20
200
2000
200
2000
20000
Both
Prioritize health
Increase government spending
Observed

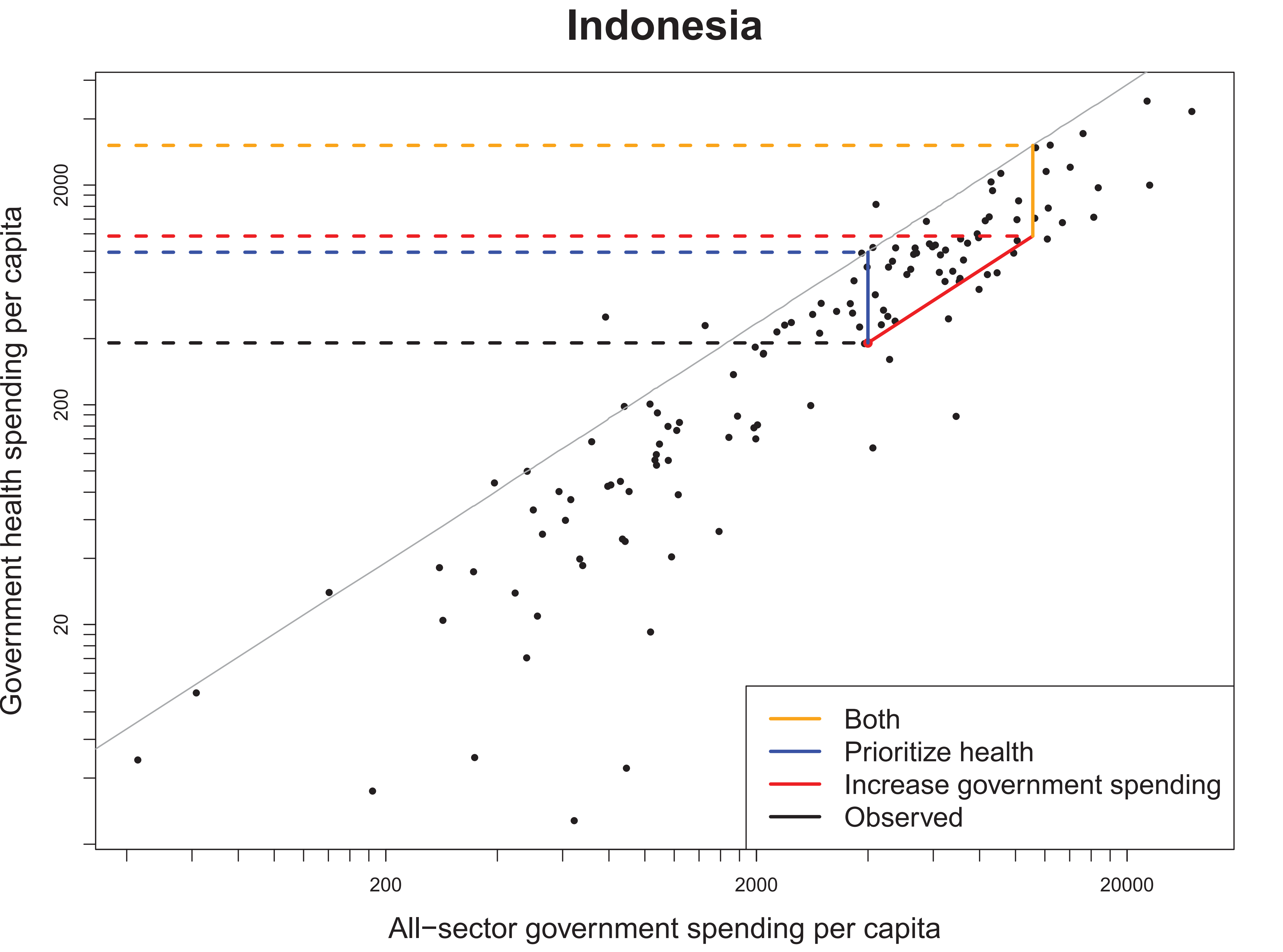

Indonesia
Government health spending per capita
All−sector government spending per capita
20
200
2000
200
2000
20000
Both
Prioritize health
Increase government spending
Observed

# Iran

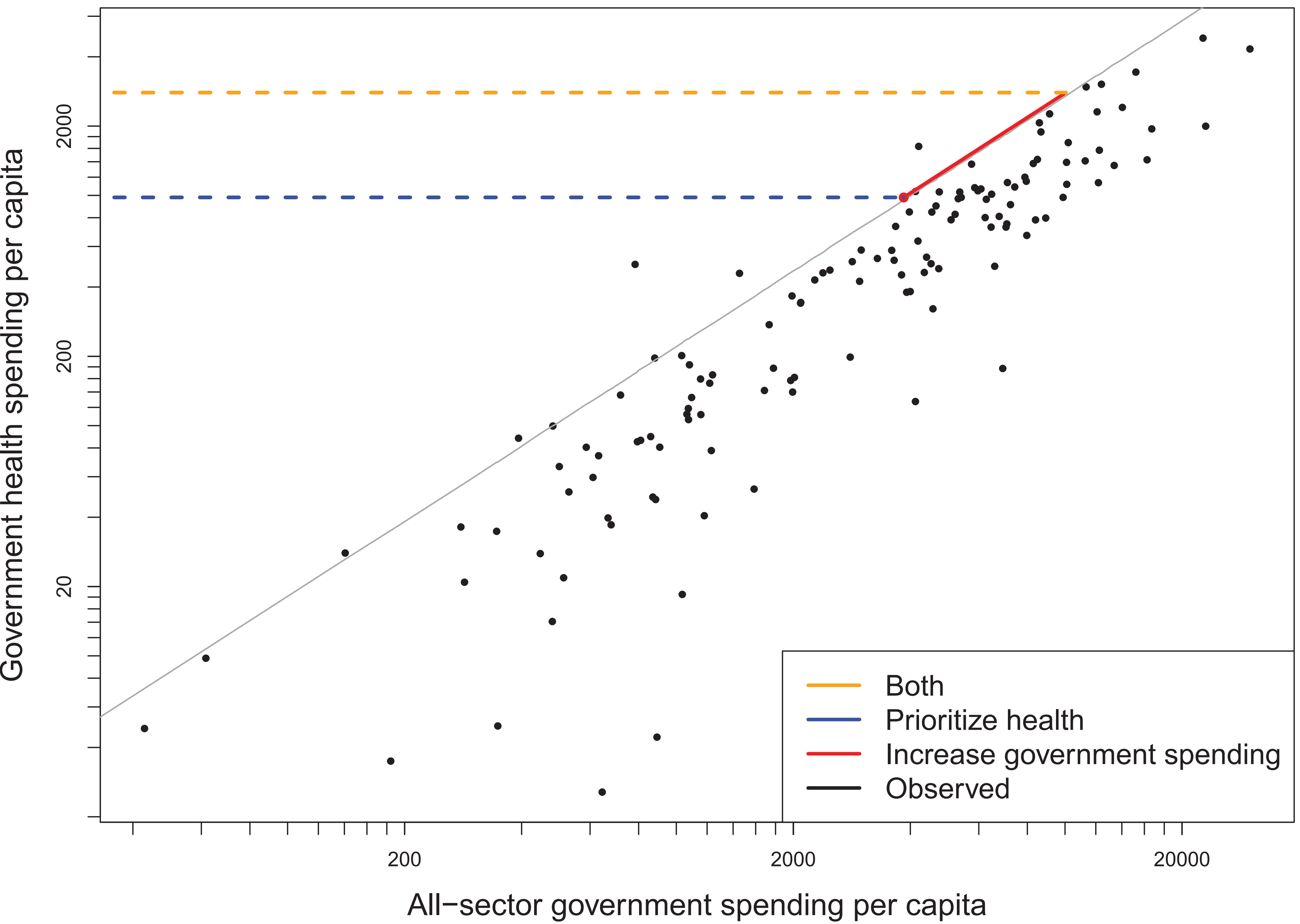

Government health spending per capita
20
200
2000
All−sector government spending per capita
200
2000
20000
Both
Prioritize health
Increase government spending
Observed

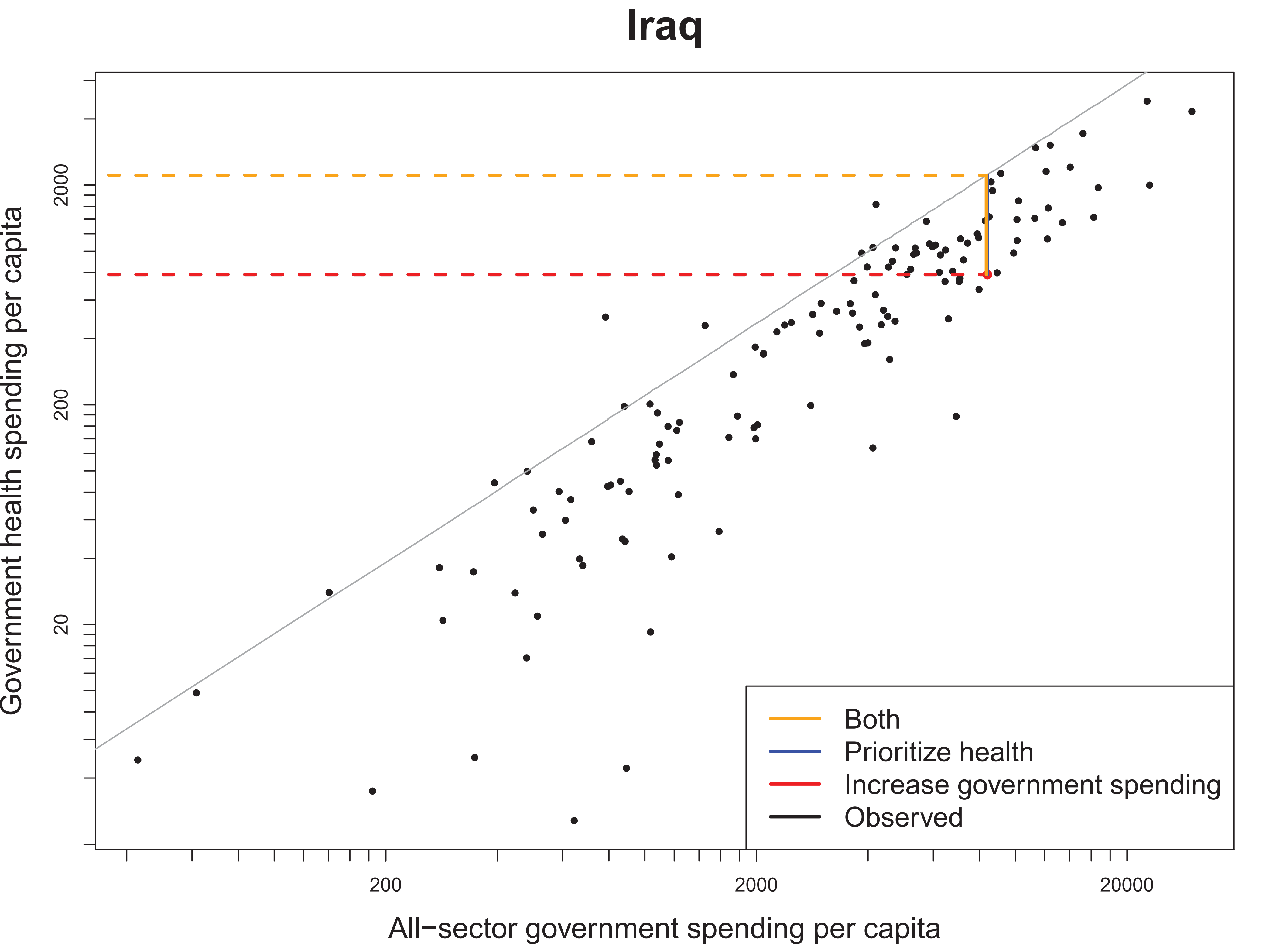

Iraq
Government health spending per capita
20
200
2000
All−sector government spending per capita
200
2000
20000
Both
Prioritize health
Increase government spending
Observed

# Jamaica

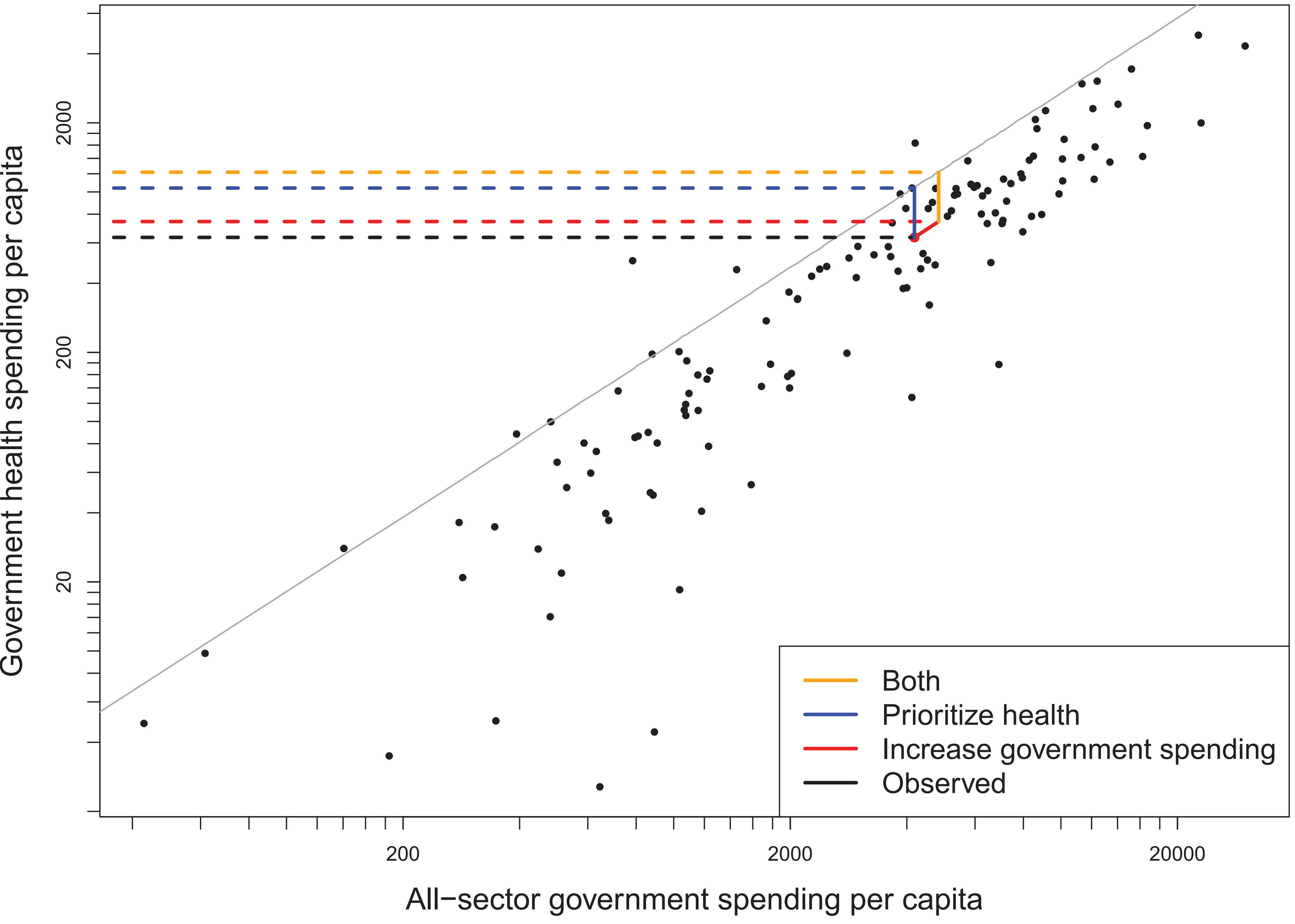

Government health spending per capita
2000
200
20
200
2000
20000
All−sector government spending per capita
Both
Prioritize health
Increase government spending
Observed

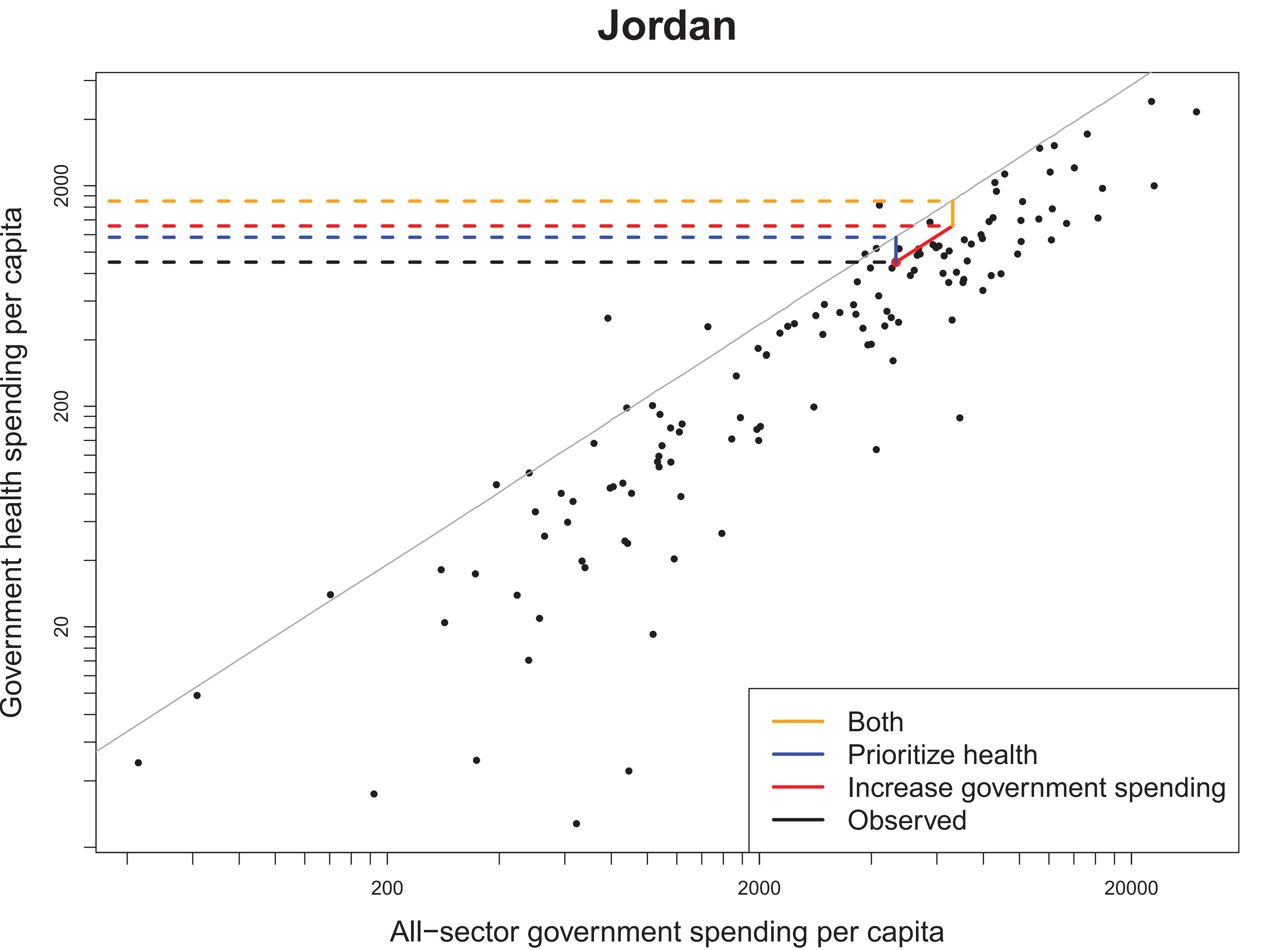

Jordan
Government health spending per capita
20
200
2000
All−sector government spending per capita
200
2000
20000
Both
Prioritize health
Increase government spending
Observed

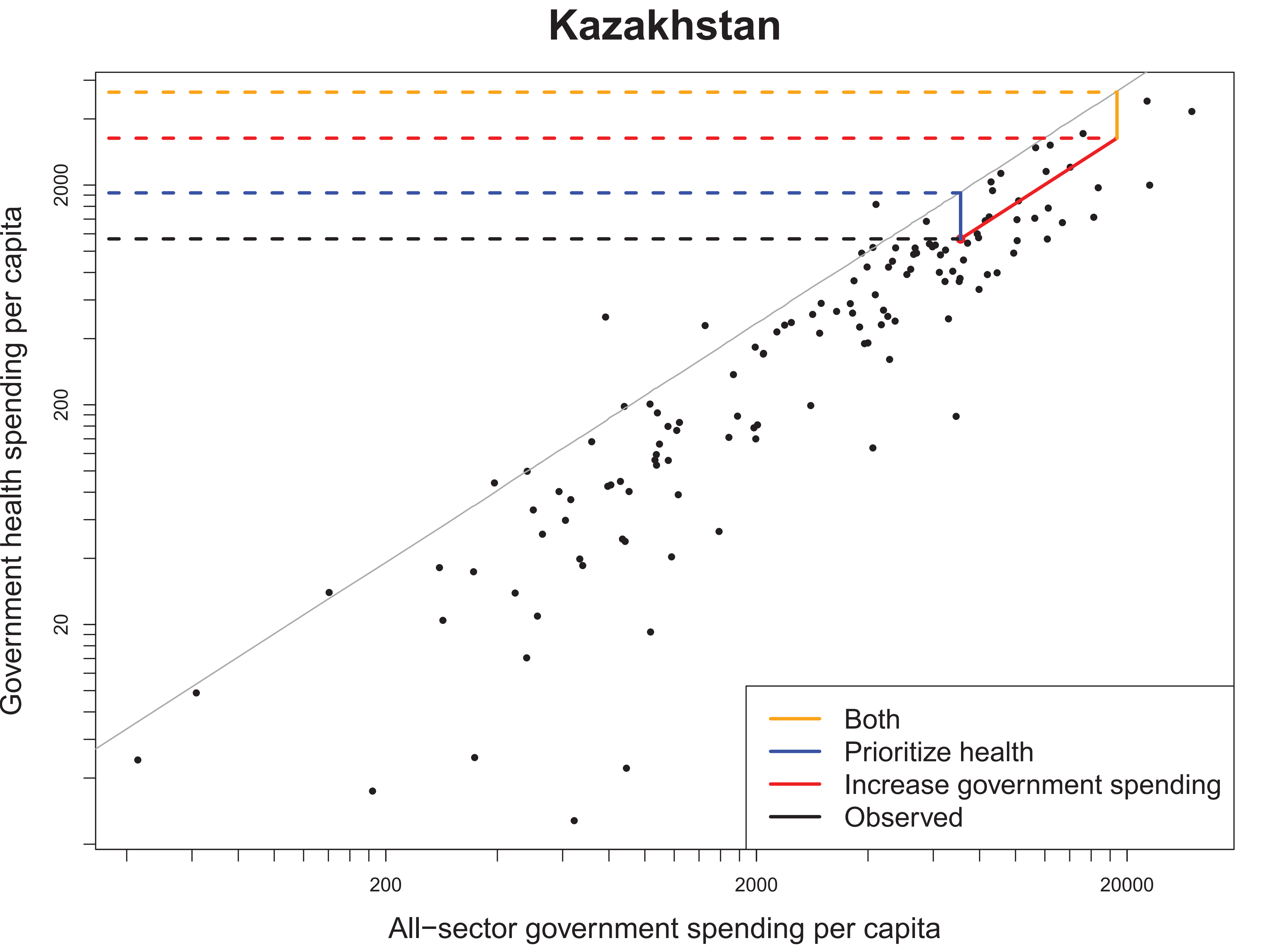

Kazakhstan
Government health spending per capita
2000
200
20
200
2000
20000
All−sector government spending per capita
Both
Prioritize health
Increase government spending
Observed

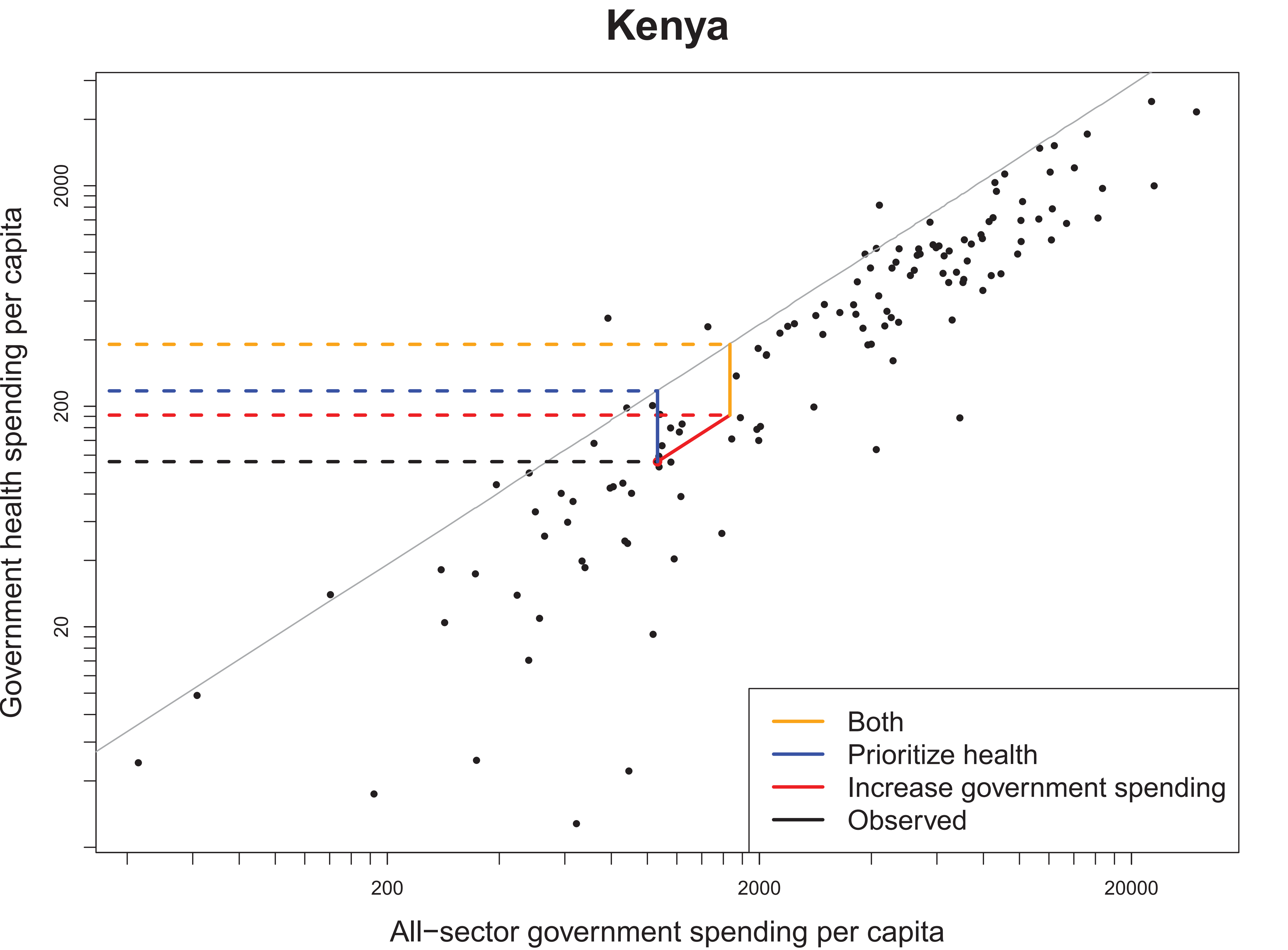

Kenya
Government health spending per capita
All−sector government spending per capita
20
200
2000
200
2000
20000
Both
Prioritize health
Increase government spending
Observed

# Kiribati

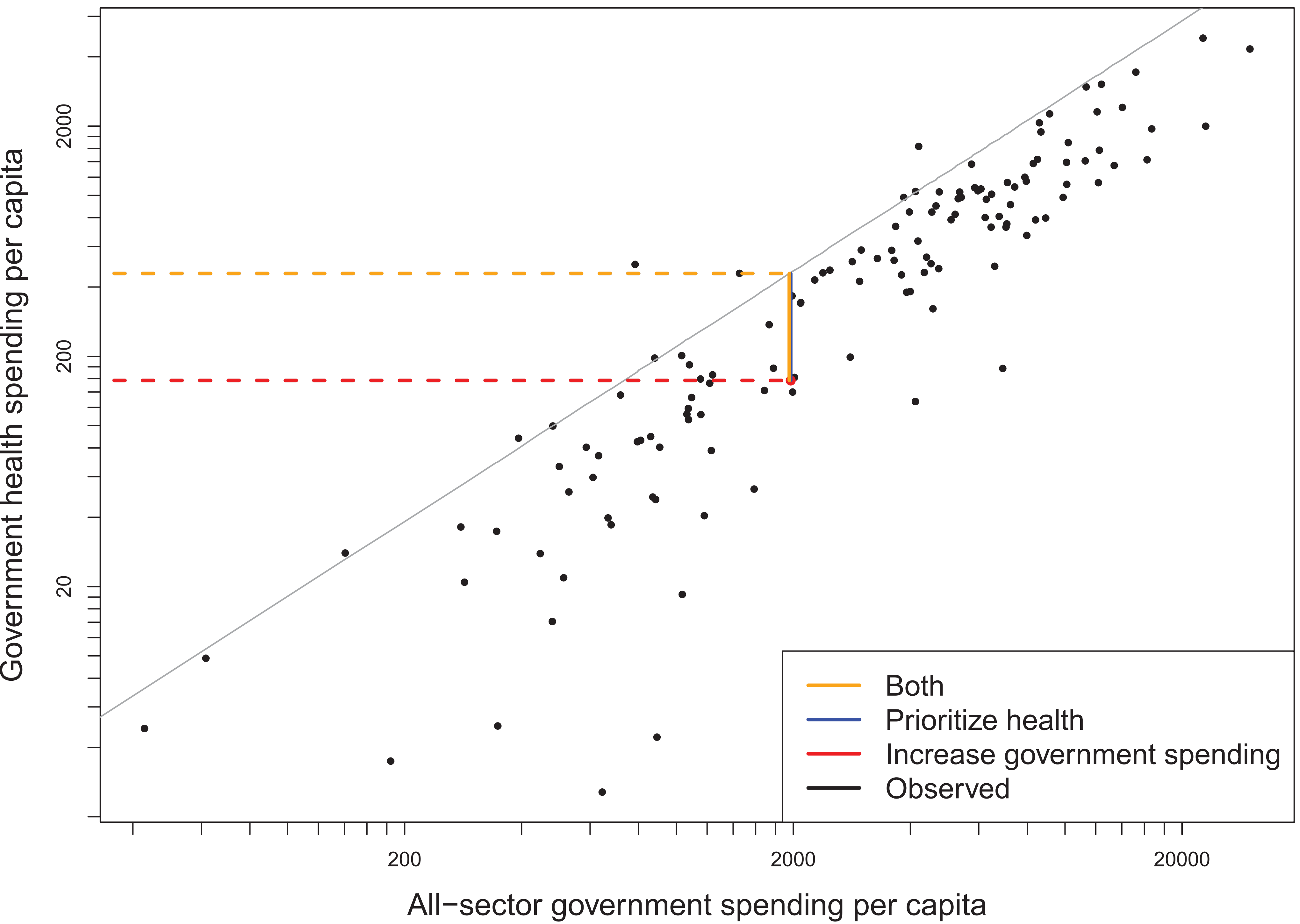

Government health spending per capita
2000
200
20
200
2000
20000
All−sector government spending per capita
Both
Prioritize health
Increase government spending
Observed

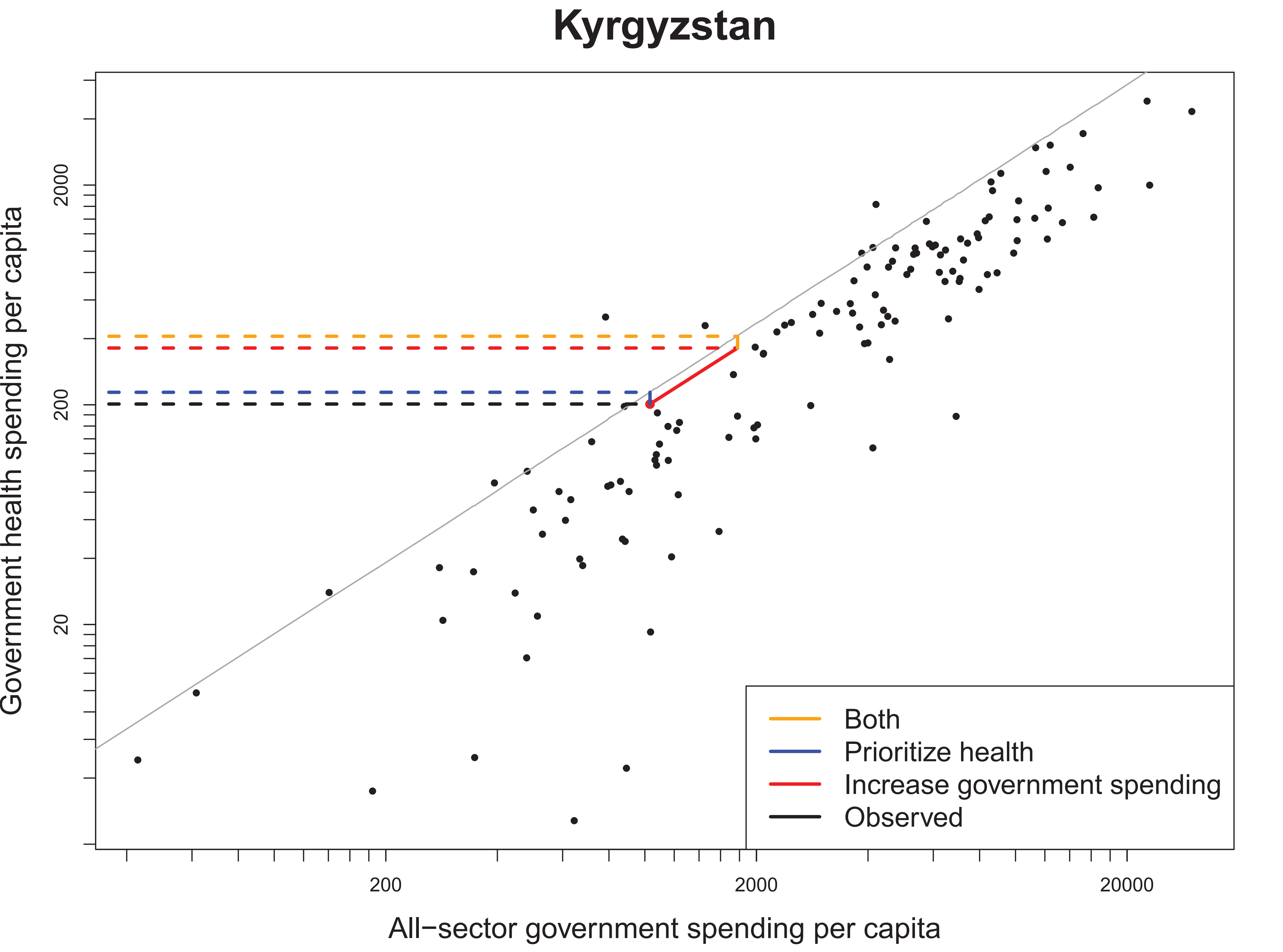

Kyrgyzstan
Government health spending per capita
2000
200
20
200
2000
20000
All−sector government spending per capita
Both
Prioritize health
Increase government spending
Observed

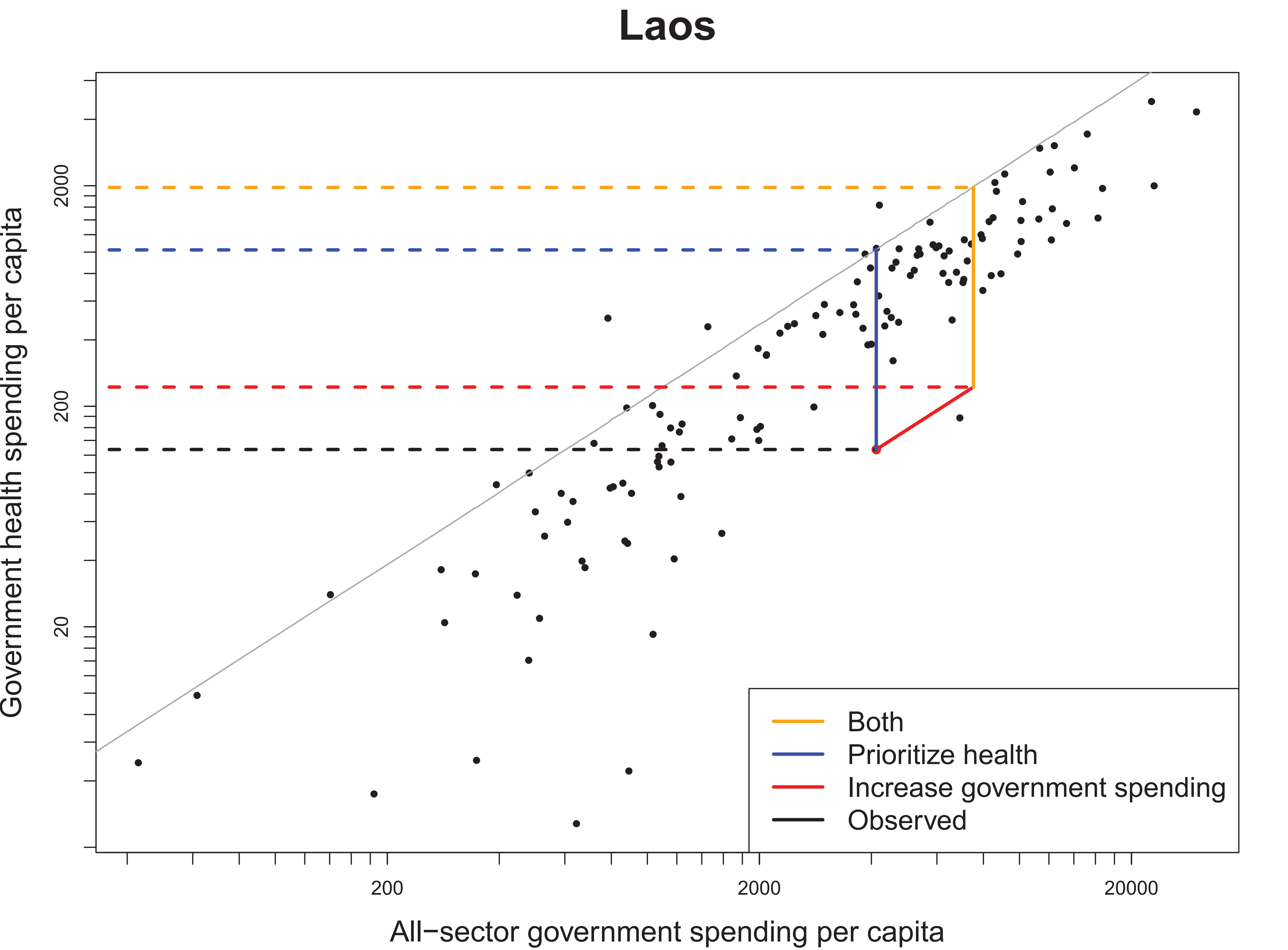

Laos
Government health spending per capita
All−sector government spending per capita
20
200
2000
200
2000
20000
Both
Prioritize health
Increase government spending
Observed

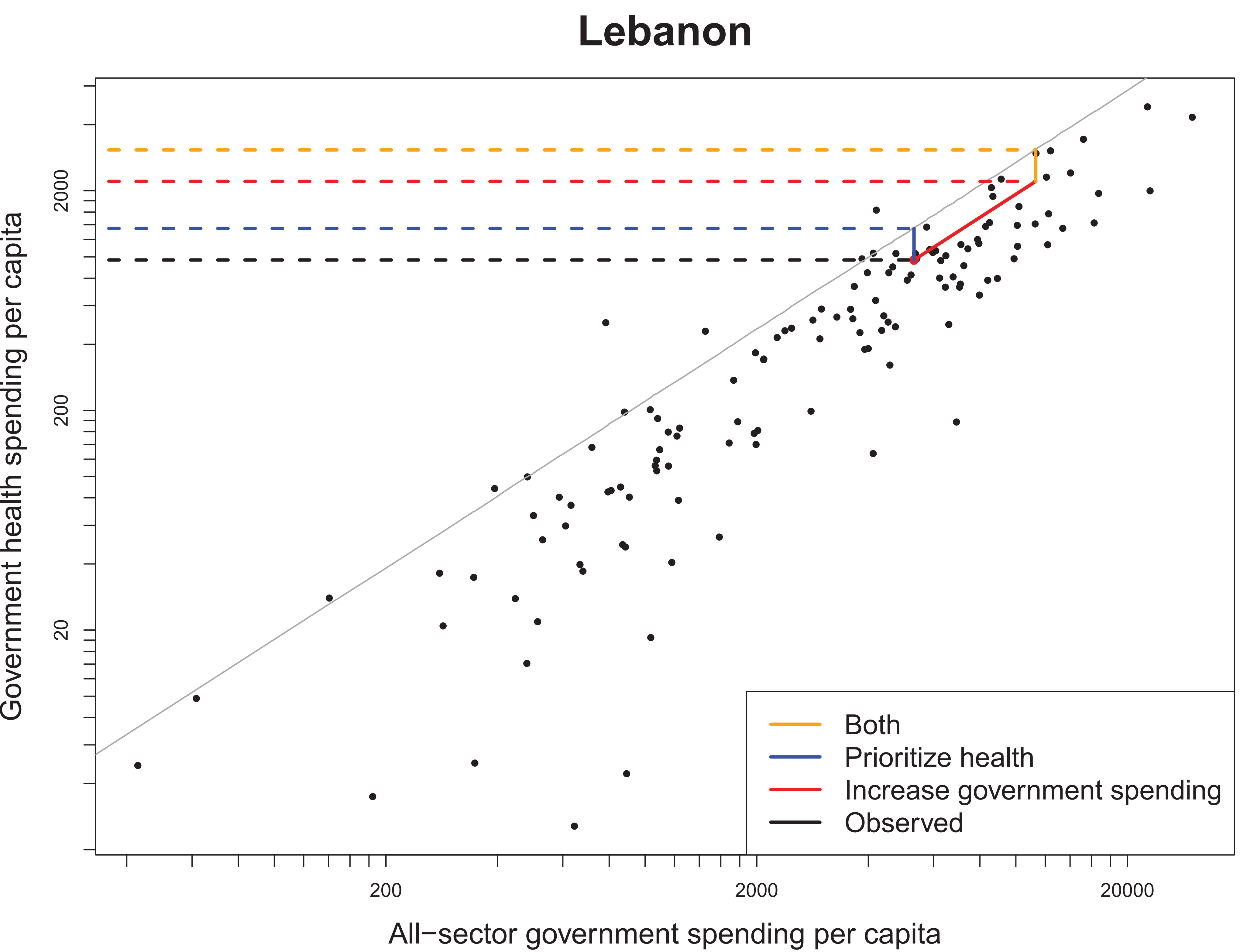

Lebanon
Government health spending per capita
20
200
2000
All−sector government spending per capita
200
2000
20000
Both
Prioritize health
Increase government spending
Observed

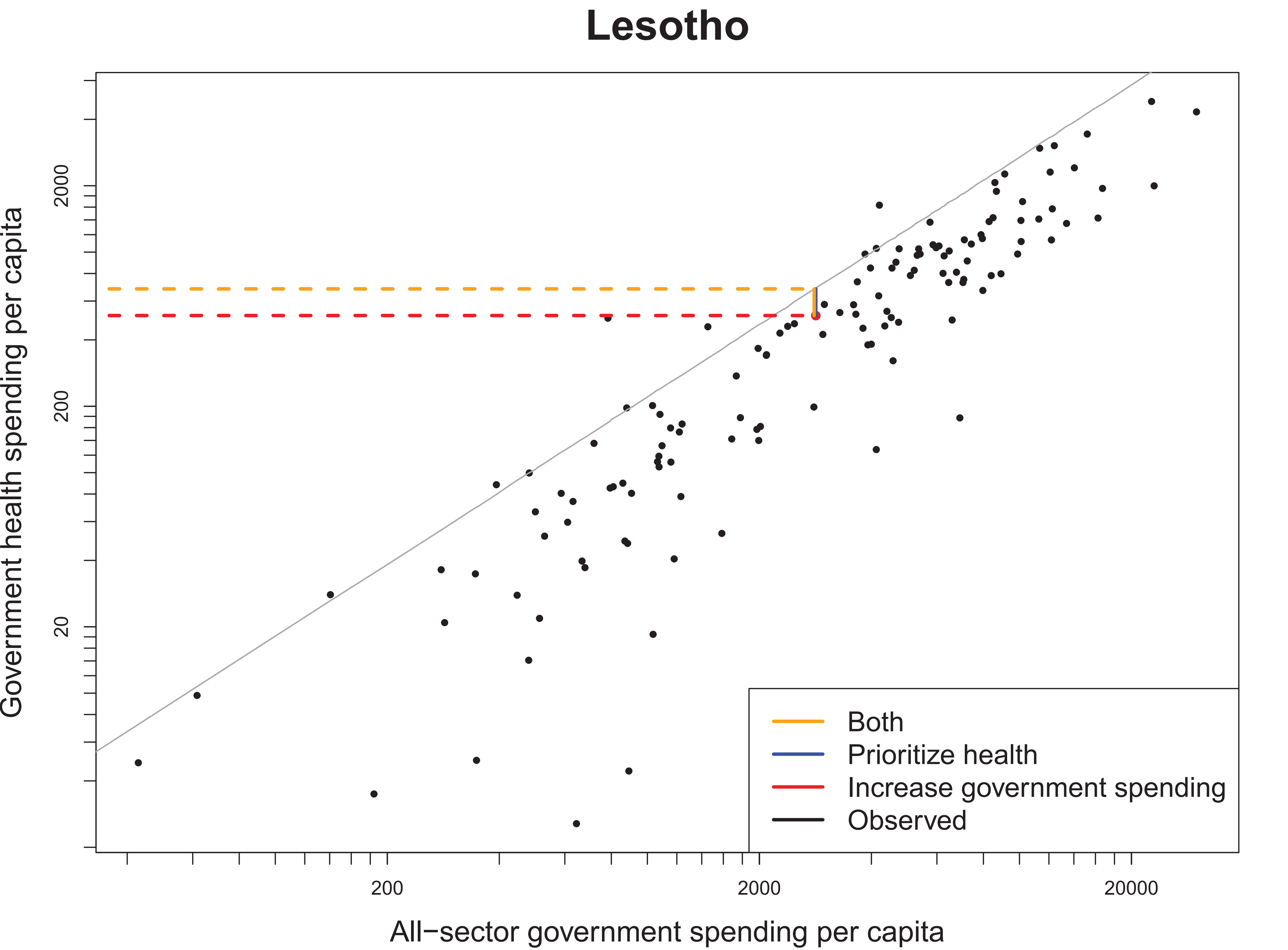

Lesotho
Government health spending per capita
All−sector government spending per capita
20
200
2000
200
2000
20000
Both
Prioritize health
Increase government spending
Observed

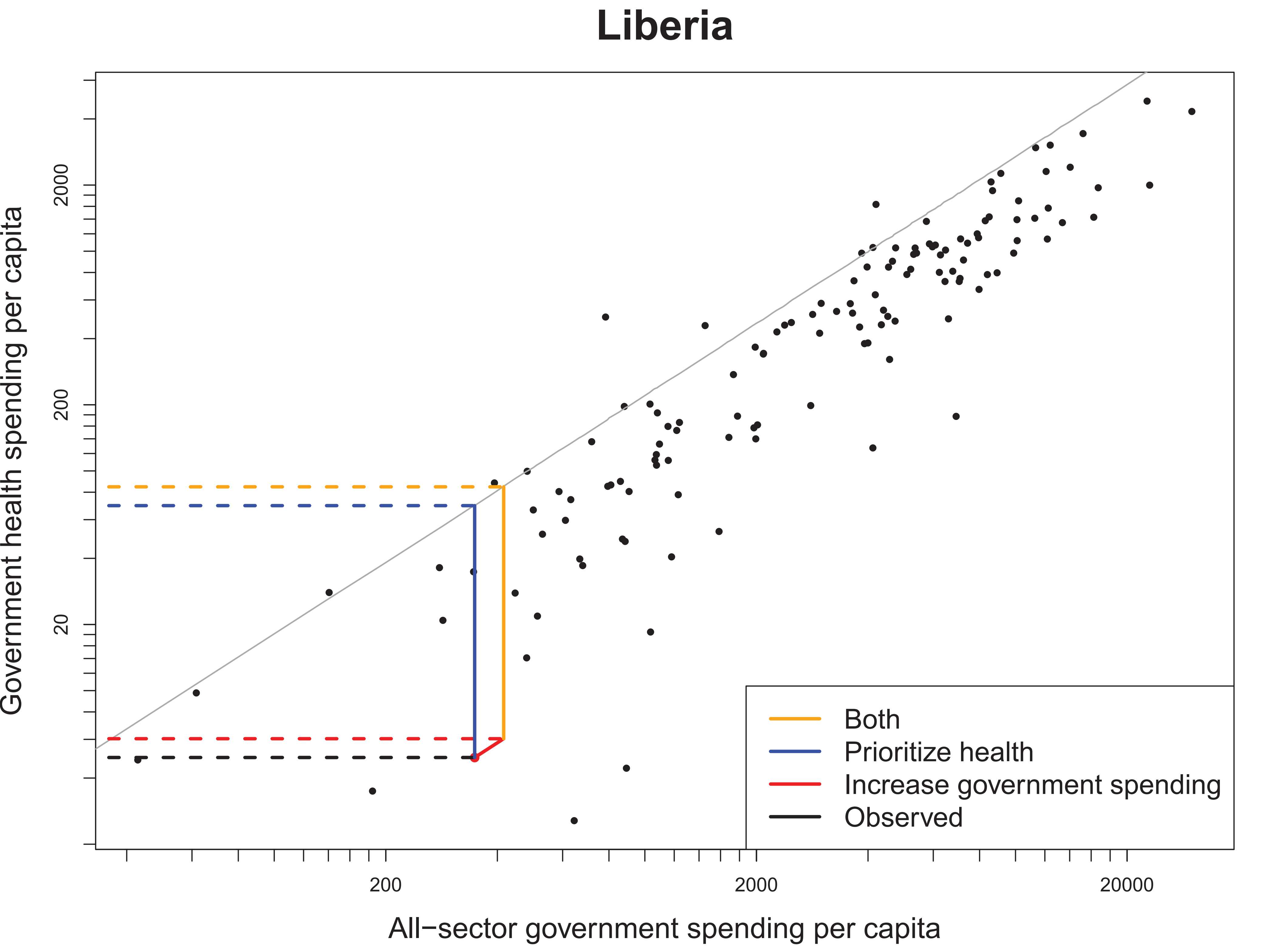
Liberia
Government health spending per capita
20
200
2000
All−sector government spending per capita
200
2000
20000
Both
Prioritize health
Increase government spending
Observed

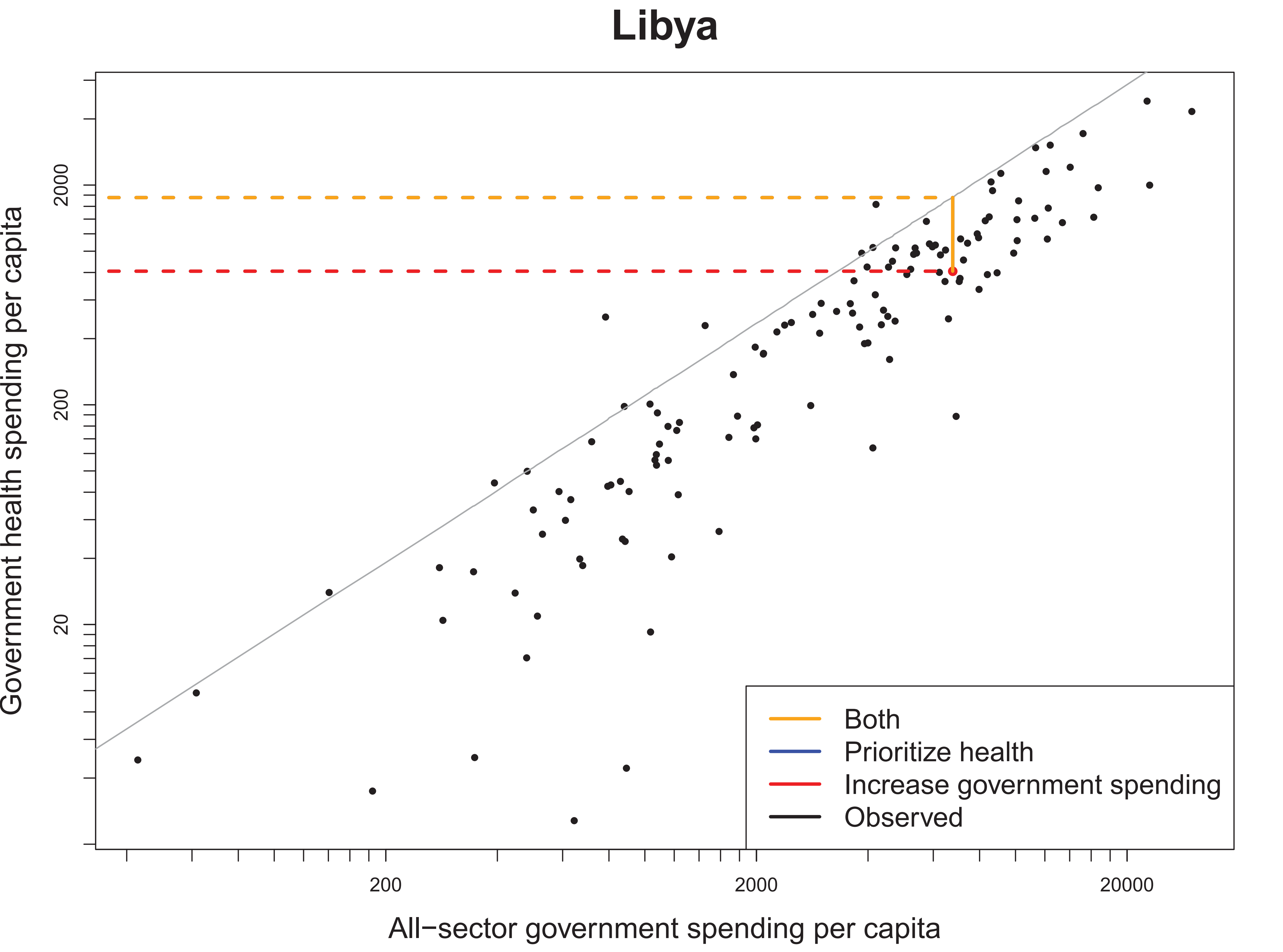
Libya
Government health spending per capita
All−sector government spending per capita
20
200
2000
200
2000
20000
Both
Prioritize health
Increase government spending
Observed

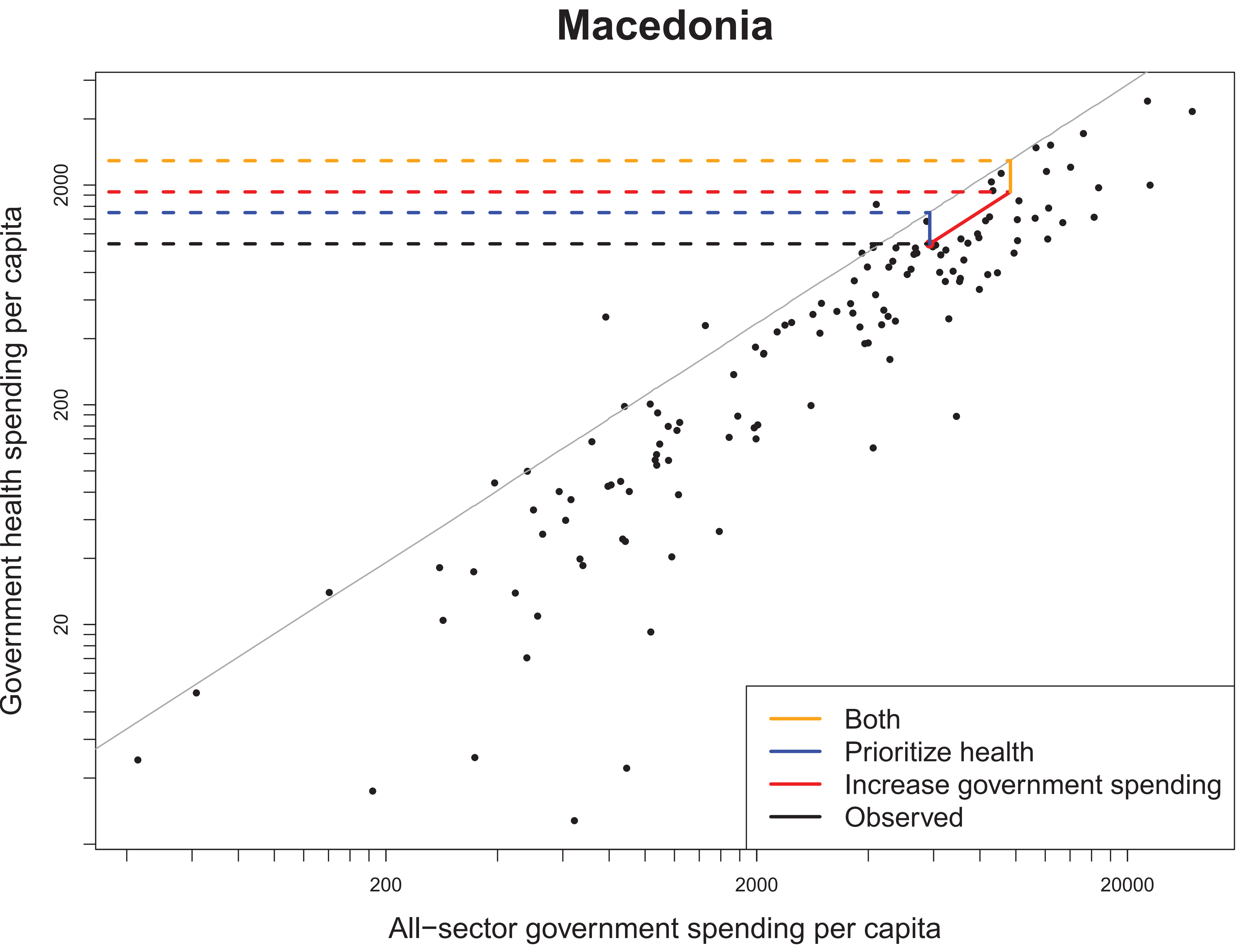
Macedonia
Government health spending per capita
20
200
2000
All−sector government spending per capita
200
2000
20000
Both
Prioritize health
Increase government spending
Observed

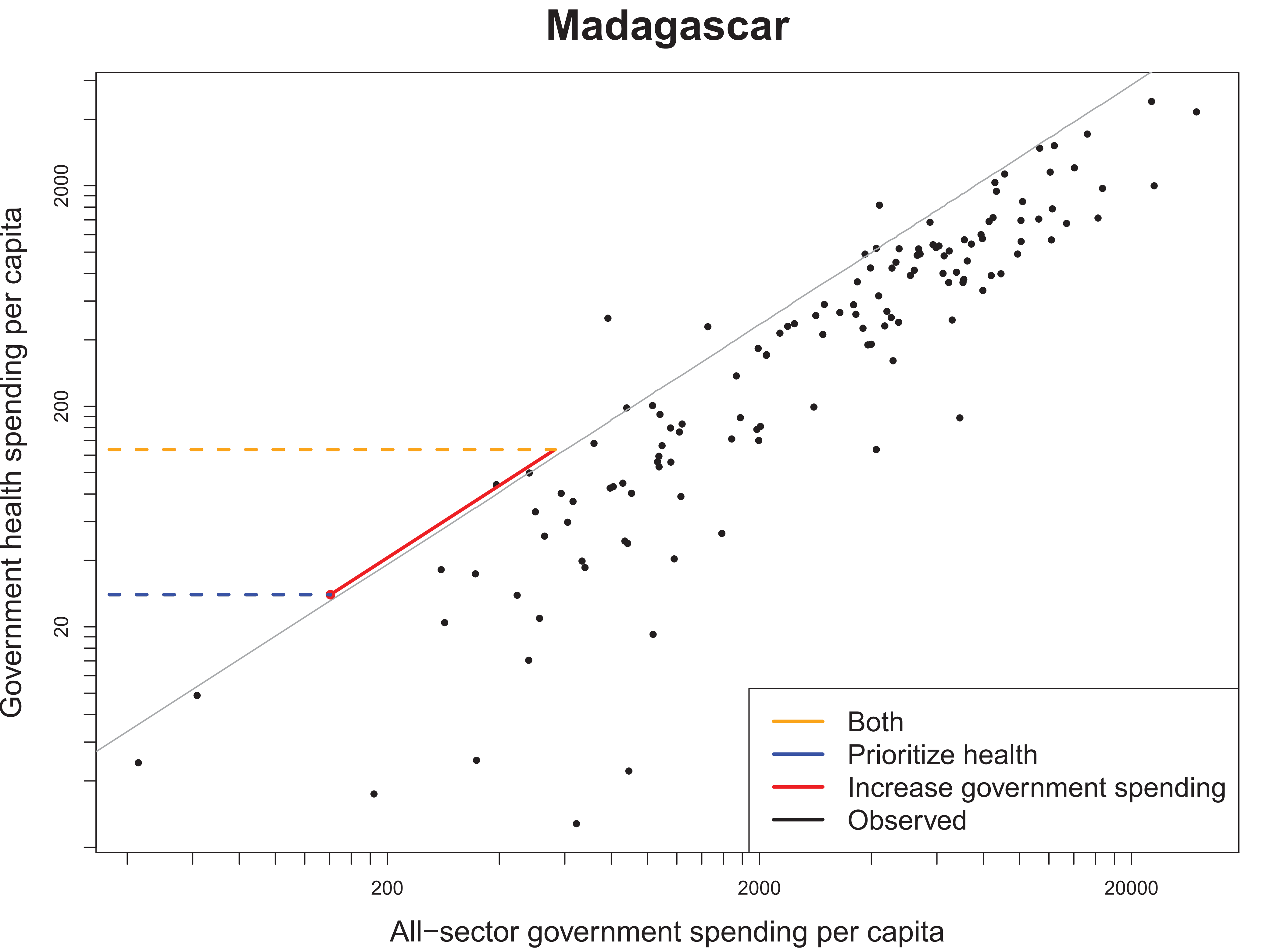

Madagascar
Government health spending per capita
20
200
2000
All−sector government spending per capita
200
2000
20000
Both
Prioritize health
Increase government spending
Observed

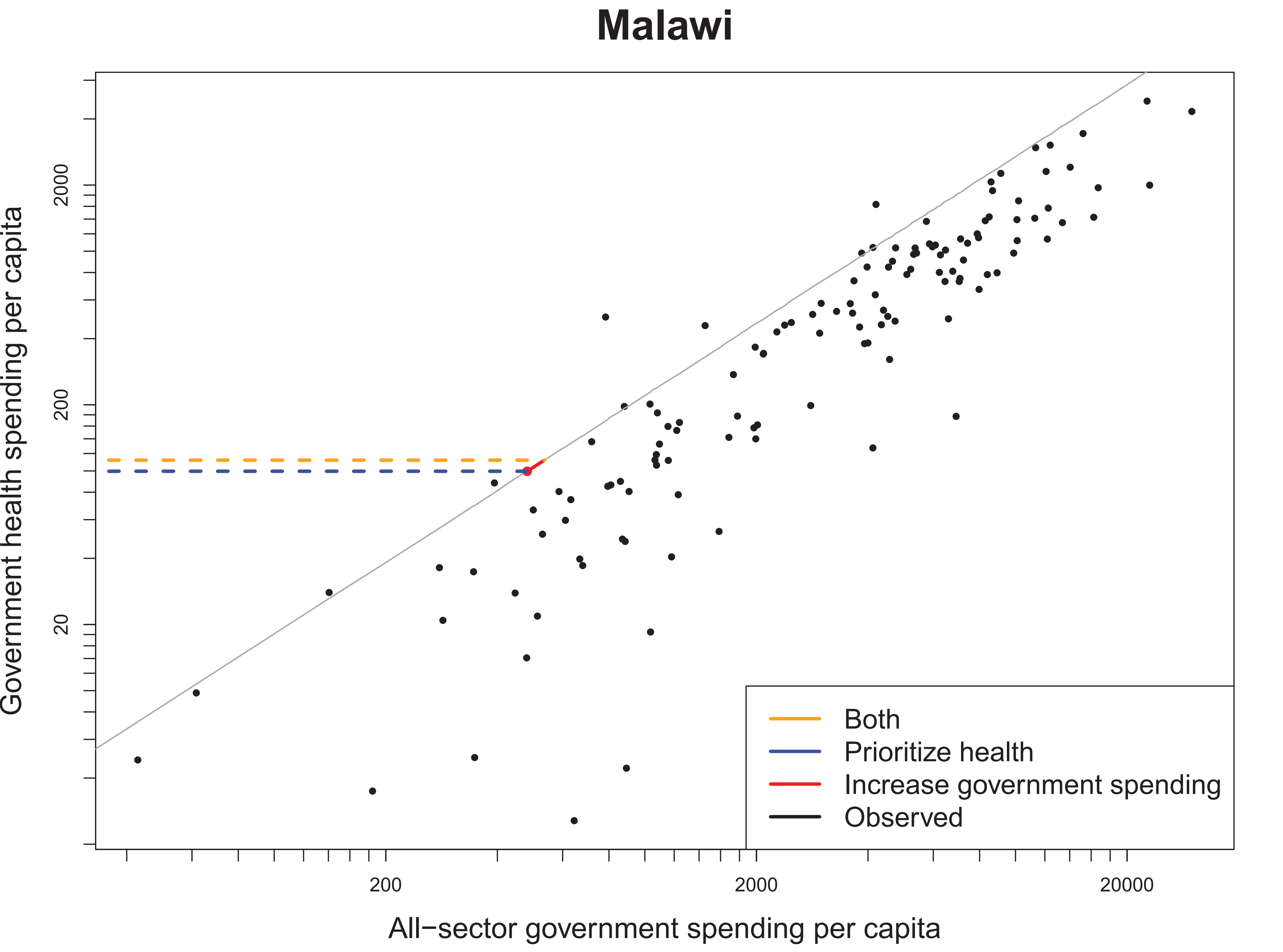

Malawi
Government health spending per capita
2000
200
20
All−sector government spending per capita
200
2000
20000
Both
Prioritize health
Increase government spending
Observed

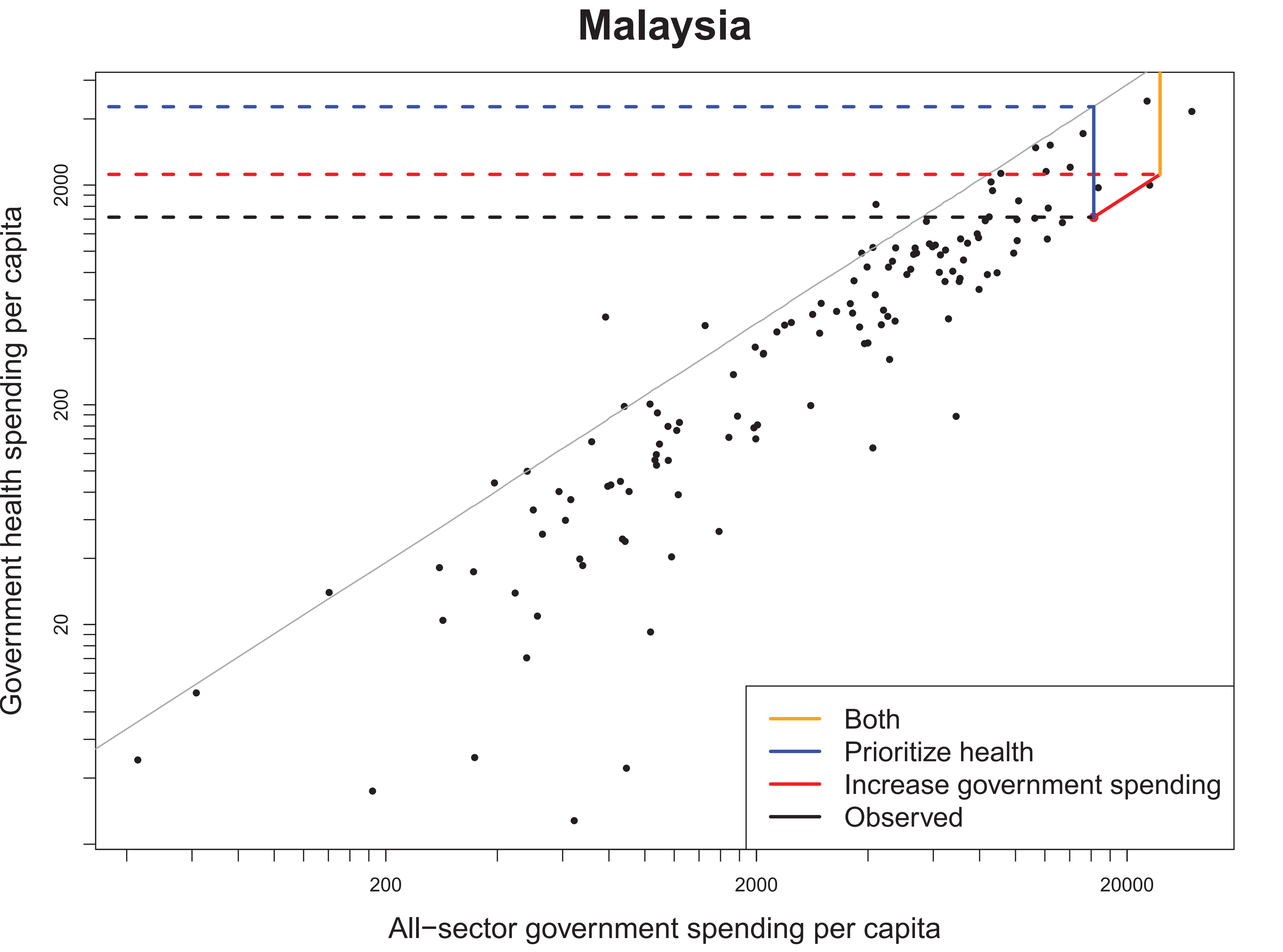

Malaysia
Both
Prioritize health
Increase government spending
Observed
2000
200
20
200
2000
20000
Government health spending per capita
All−sector government spending per capita

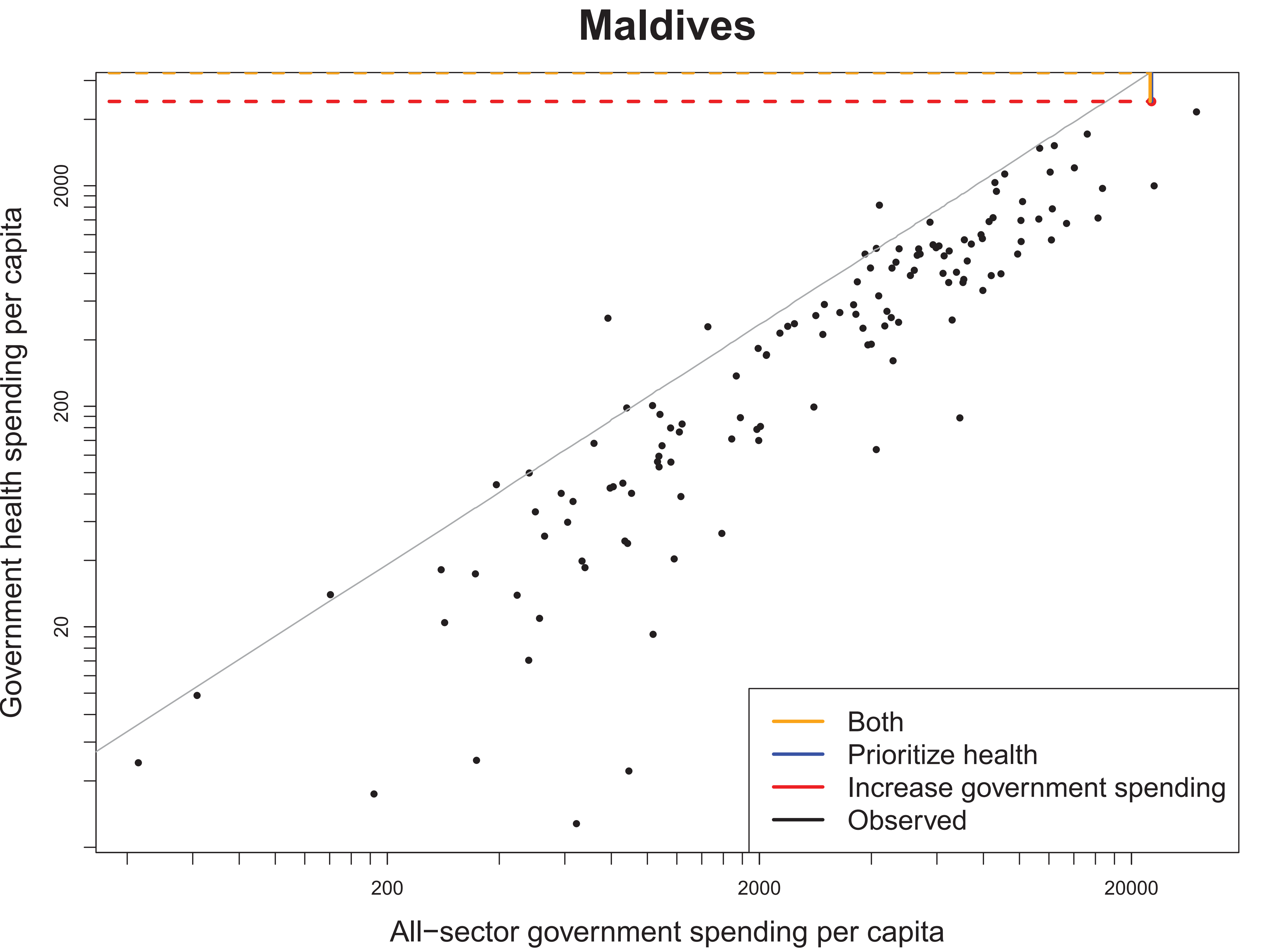

Maldives
Government health spending per capita
All−sector government spending per capita
20
200
2000
20000
Both
Prioritize health
Increase government spending
Observed

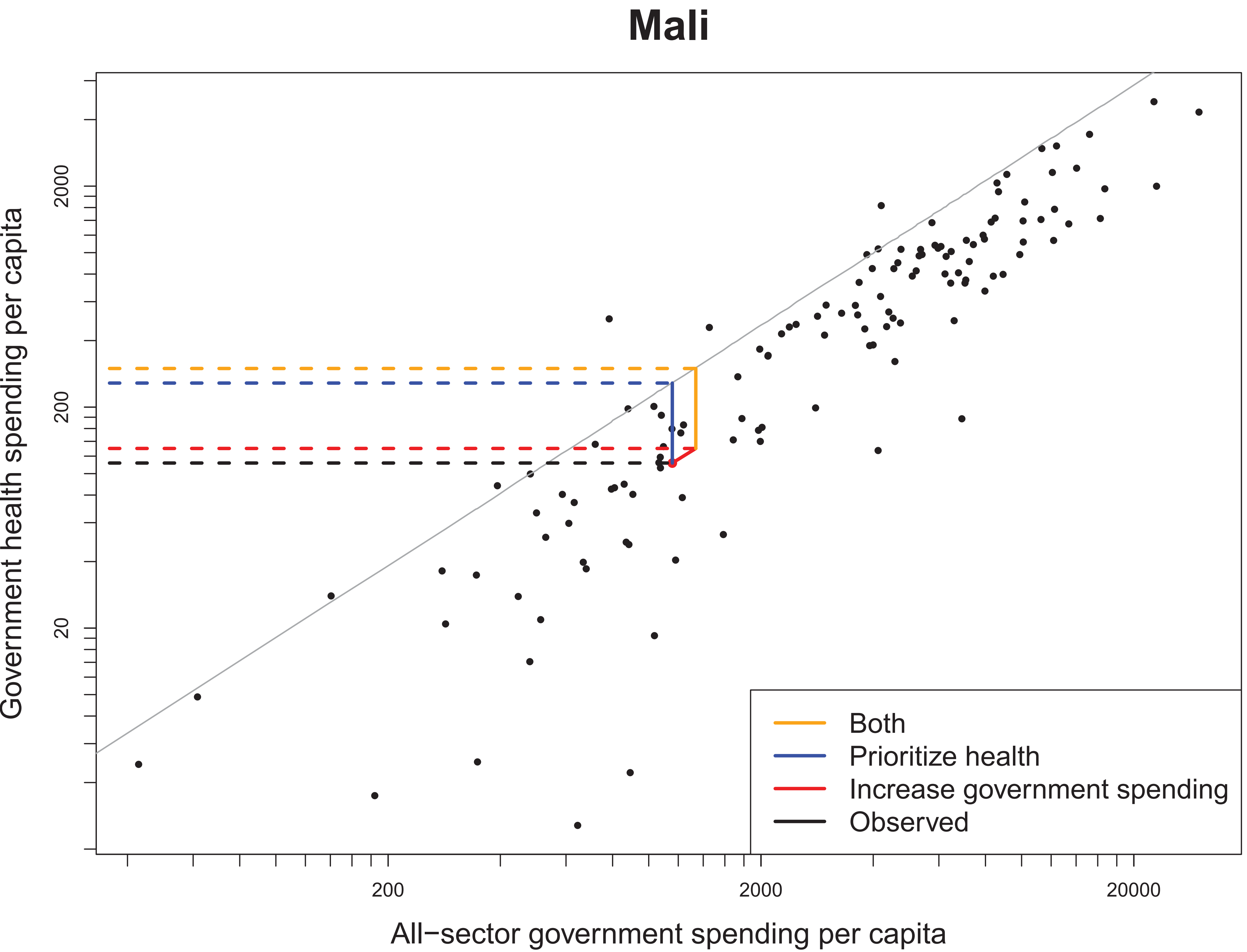
Mali
Government health spending per capita
20
200
2000
All−sector government spending per capita
200
2000
20000
Both
Prioritize health
Increase government spending
Observed

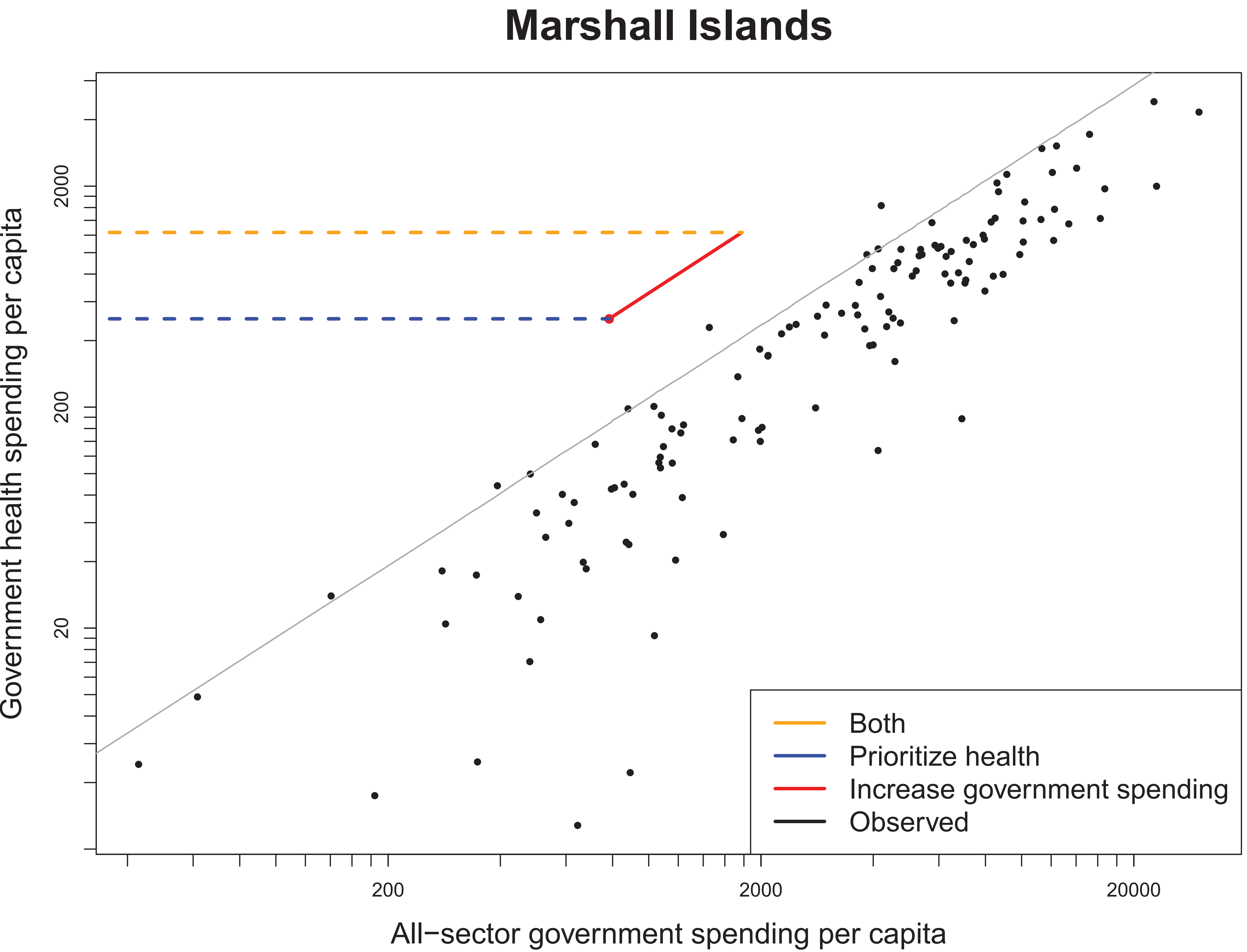
Marshall Islands
Government health spending per capita
All−sector government spending per capita
20
200
2000
20000
Both
Prioritize health
Increase government spending
Observed

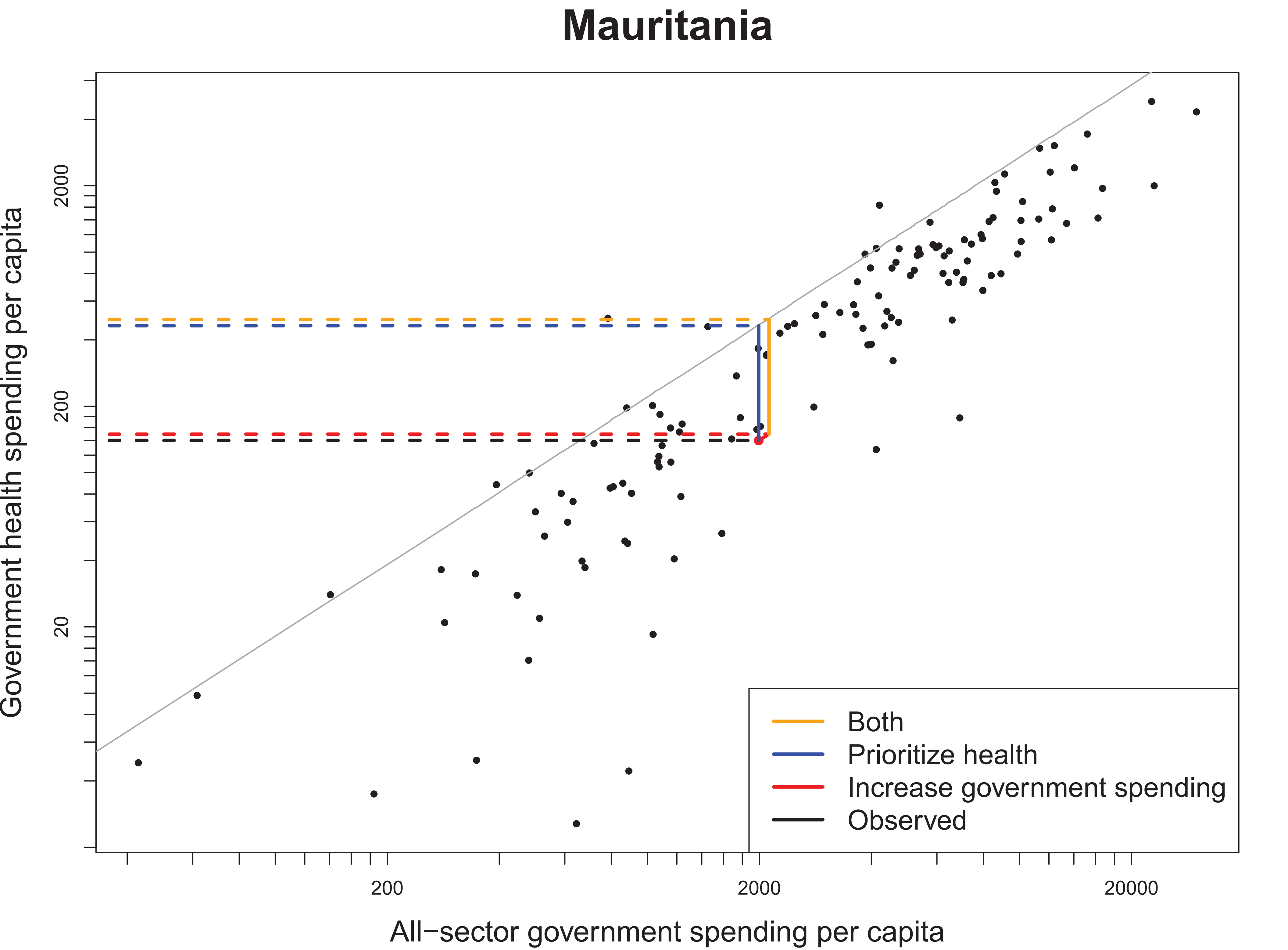
Mauritania
Both
Prioritize health
Increase government spending
Observed
Government health spending per capita
All−sector government spending per capita
20
200
2000
200
2000
20000

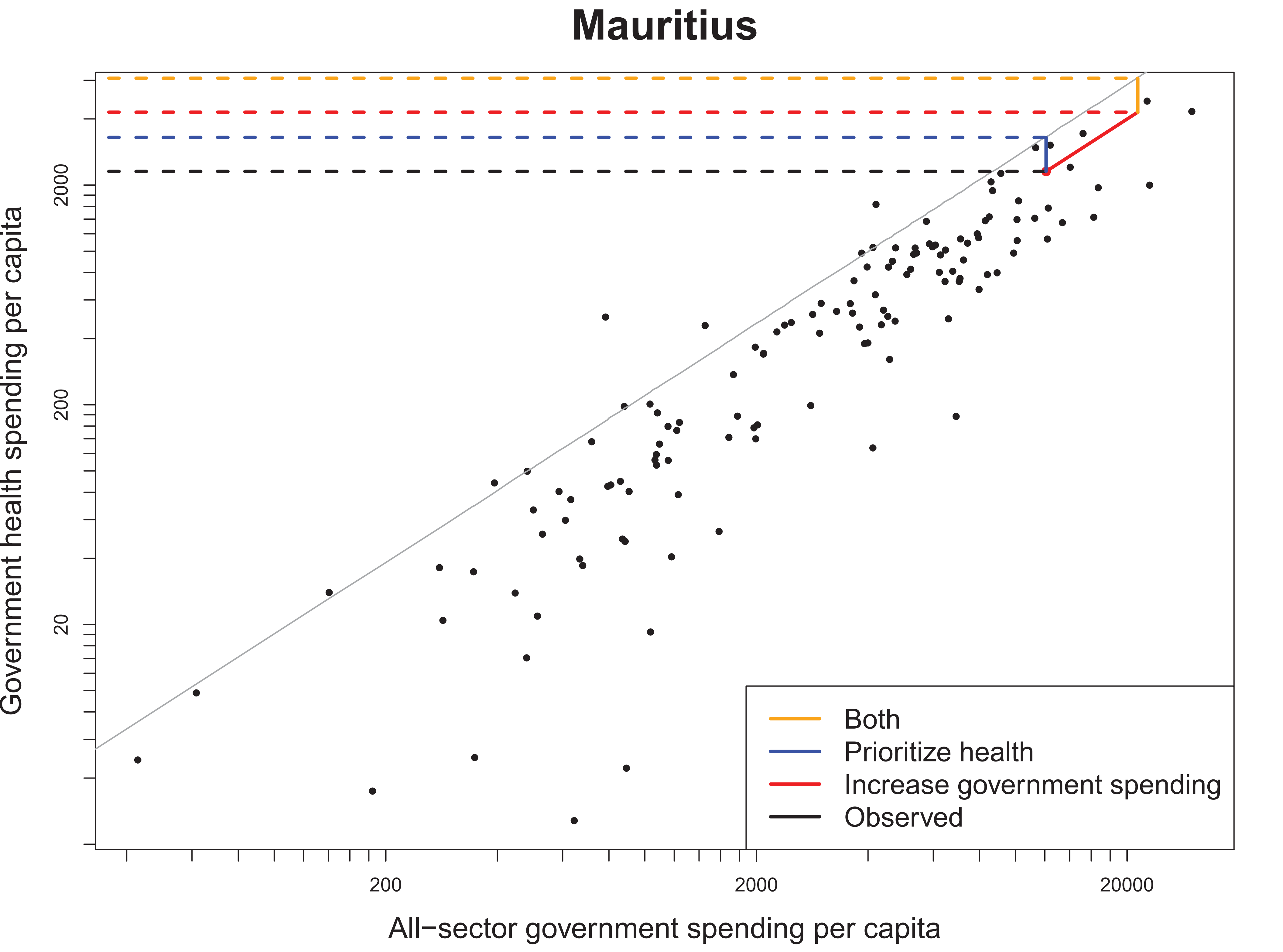

Mauritius
Both
Prioritize health
Increase government spending
Observed
Government health spending per capita
20
200
2000
All−sector government spending per capita
200
2000
20000

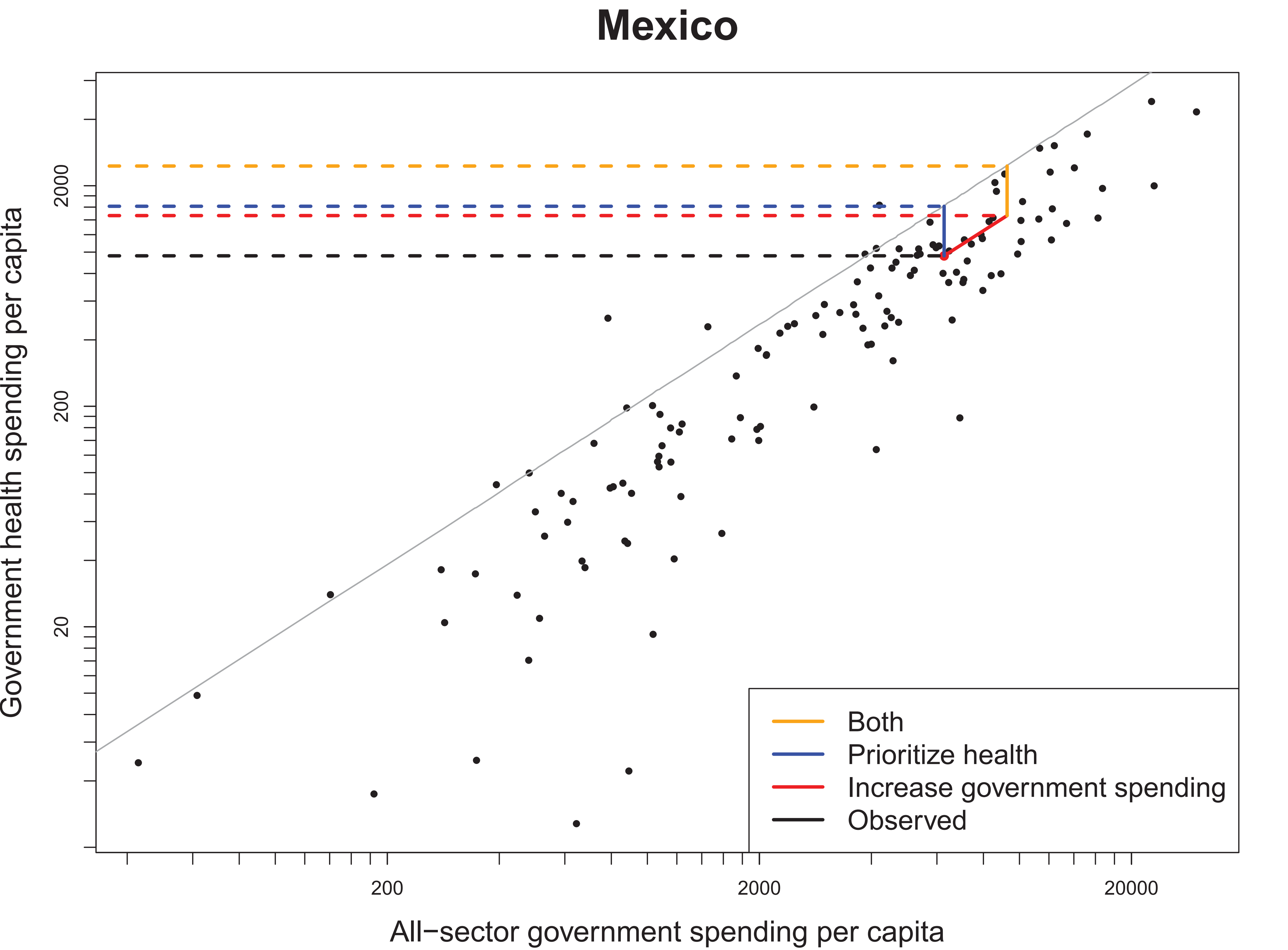
Mexico
Government health spending per capita
All−sector government spending per capita
20
200
2000
200
2000
20000
Both
Prioritize health
Increase government spending
Observed

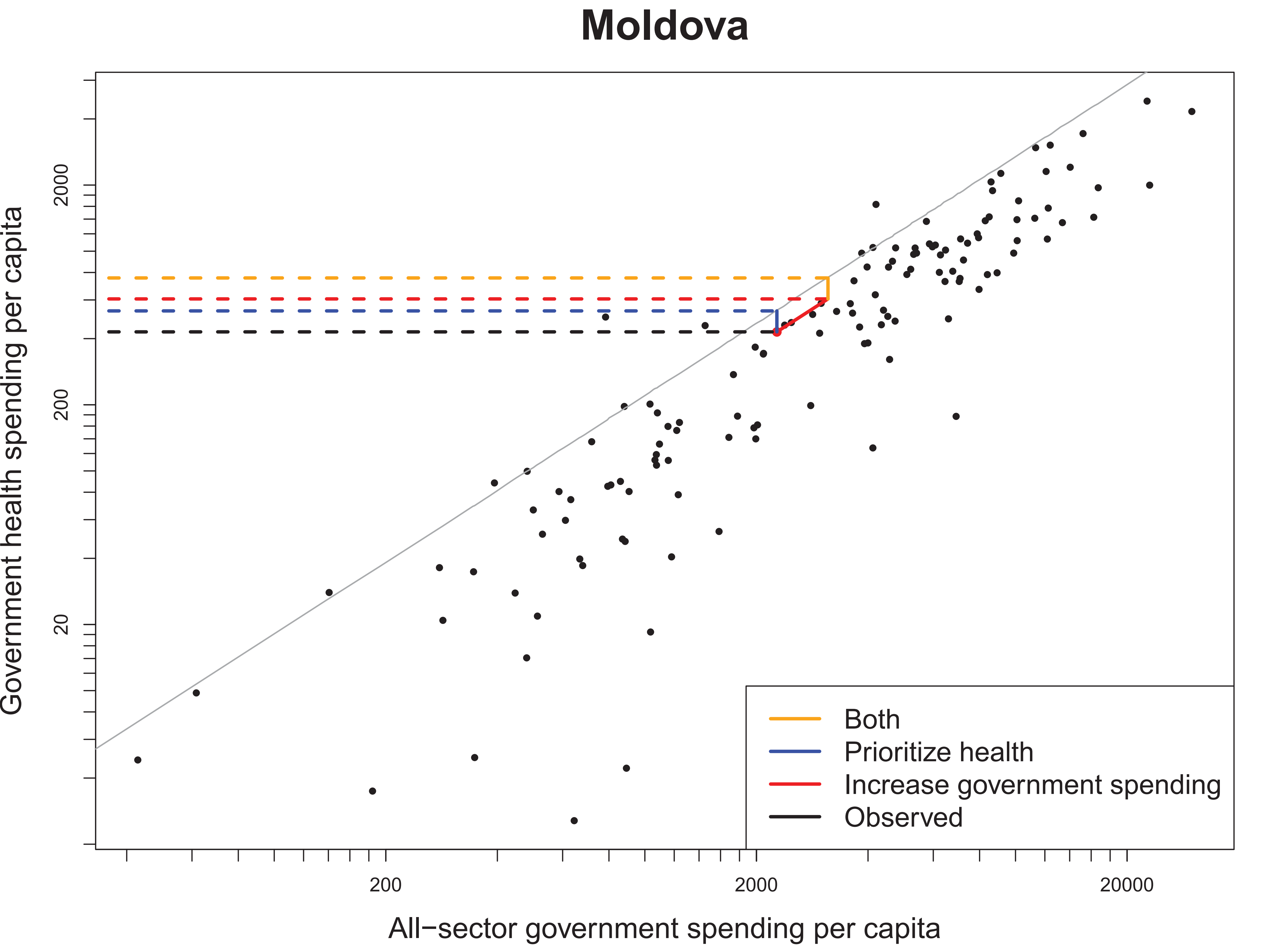

Moldova
Government health spending per capita
All−sector government spending per capita
20
200
2000
200
2000
20000
Both
Prioritize health
Increase government spending
Observed

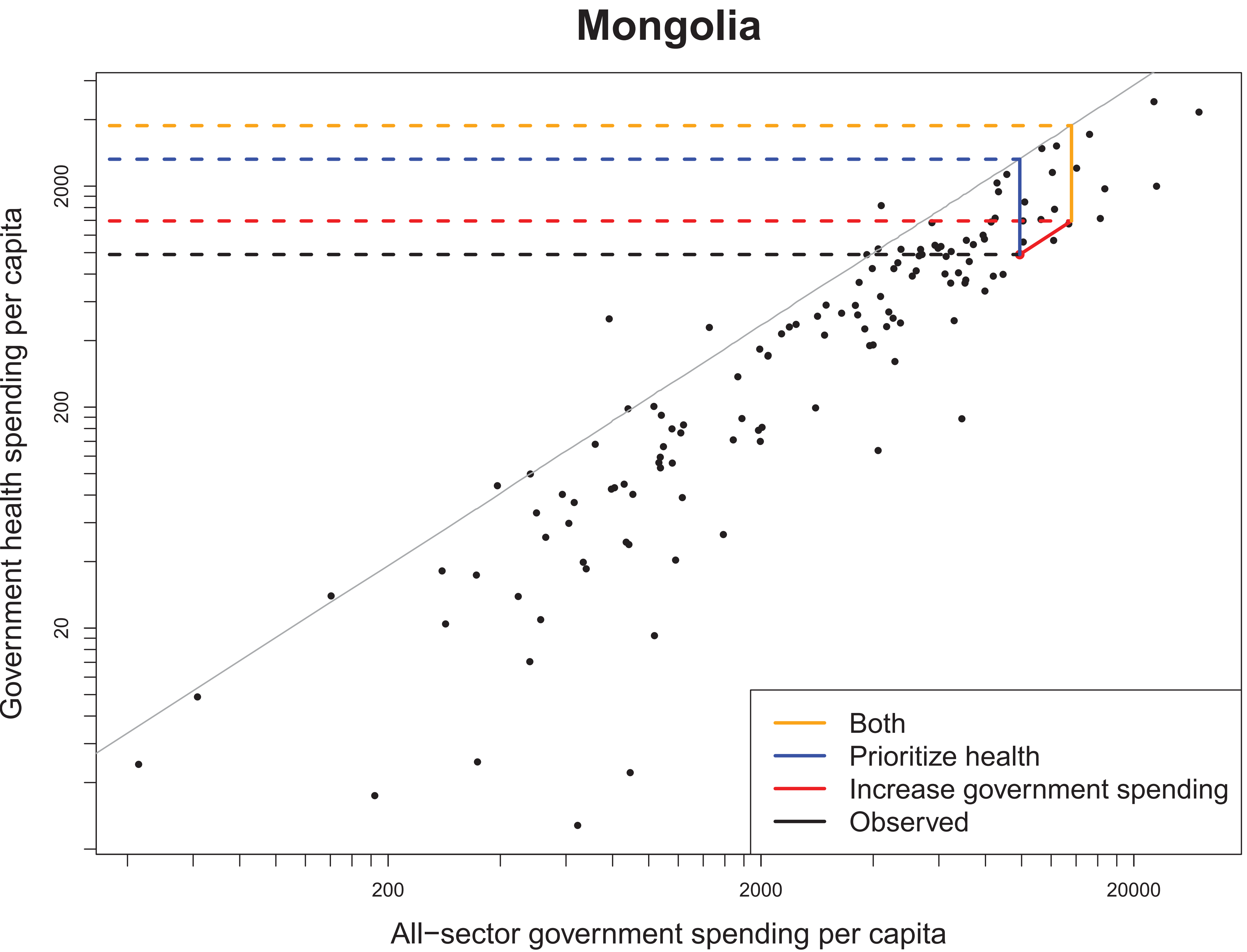

Mongolia
Government health spending per capita
20
200
2000
All−sector government spending per capita
200
2000
20000
Both
Prioritize health
Increase government spending
Observed

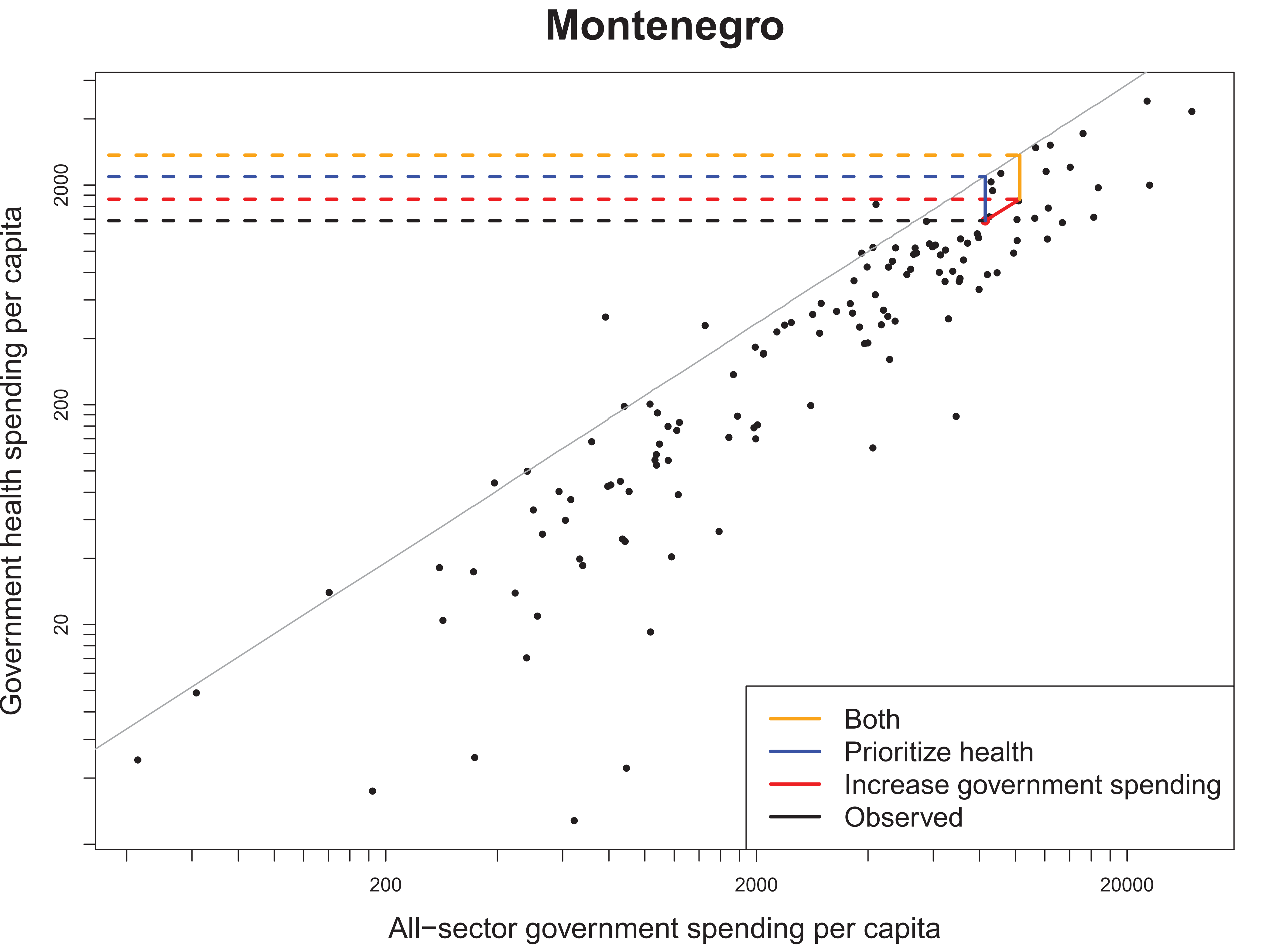
Montenegro
Government health spending per capita
All−sector government spending per capita
20
200
2000
200
2000
20000
Both
Prioritize health
Increase government spending
Observed

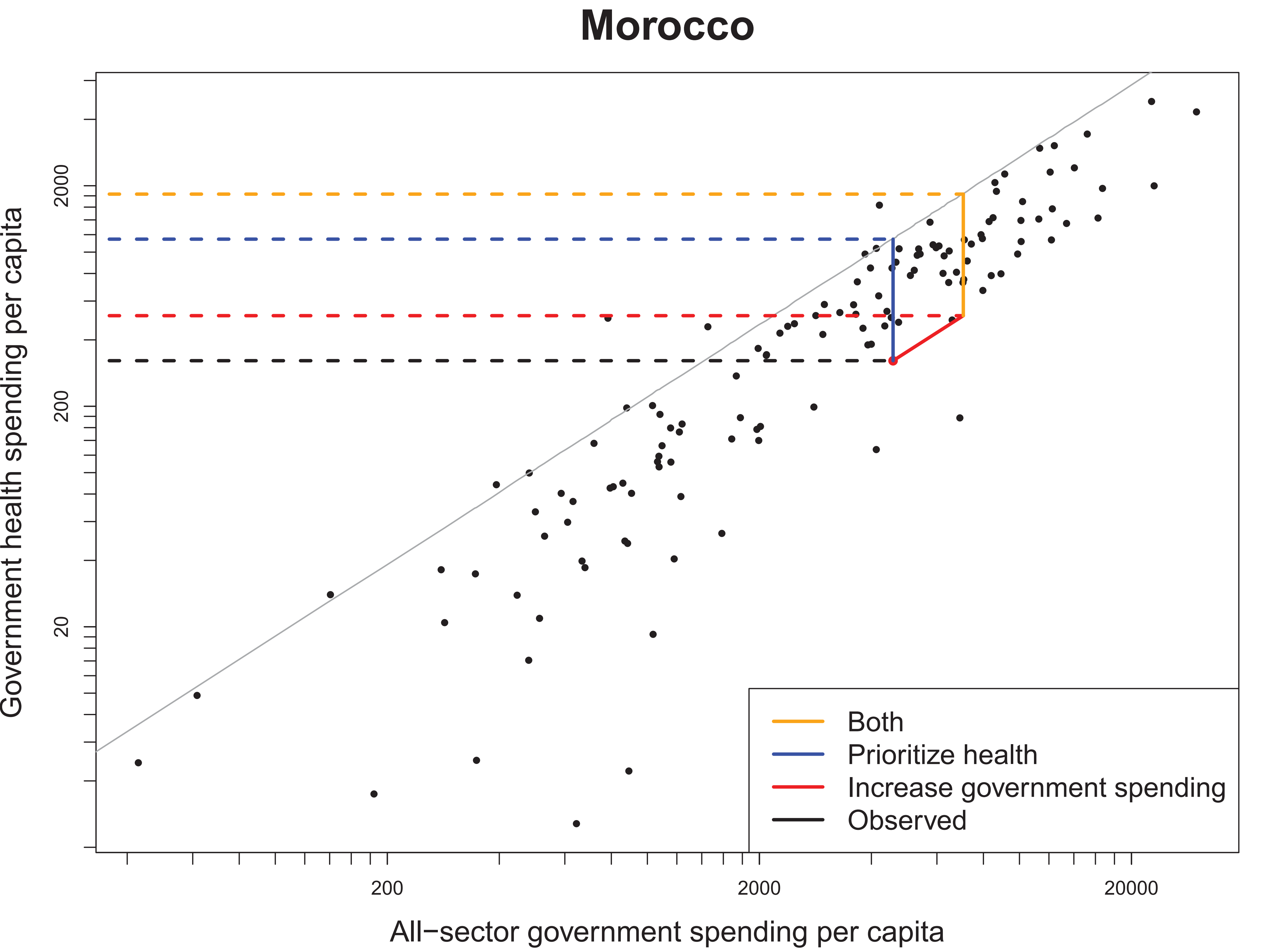

Morocco
Government health spending per capita
All−sector government spending per capita
20
200
2000
20000
Both
Prioritize health
Increase government spending
Observed

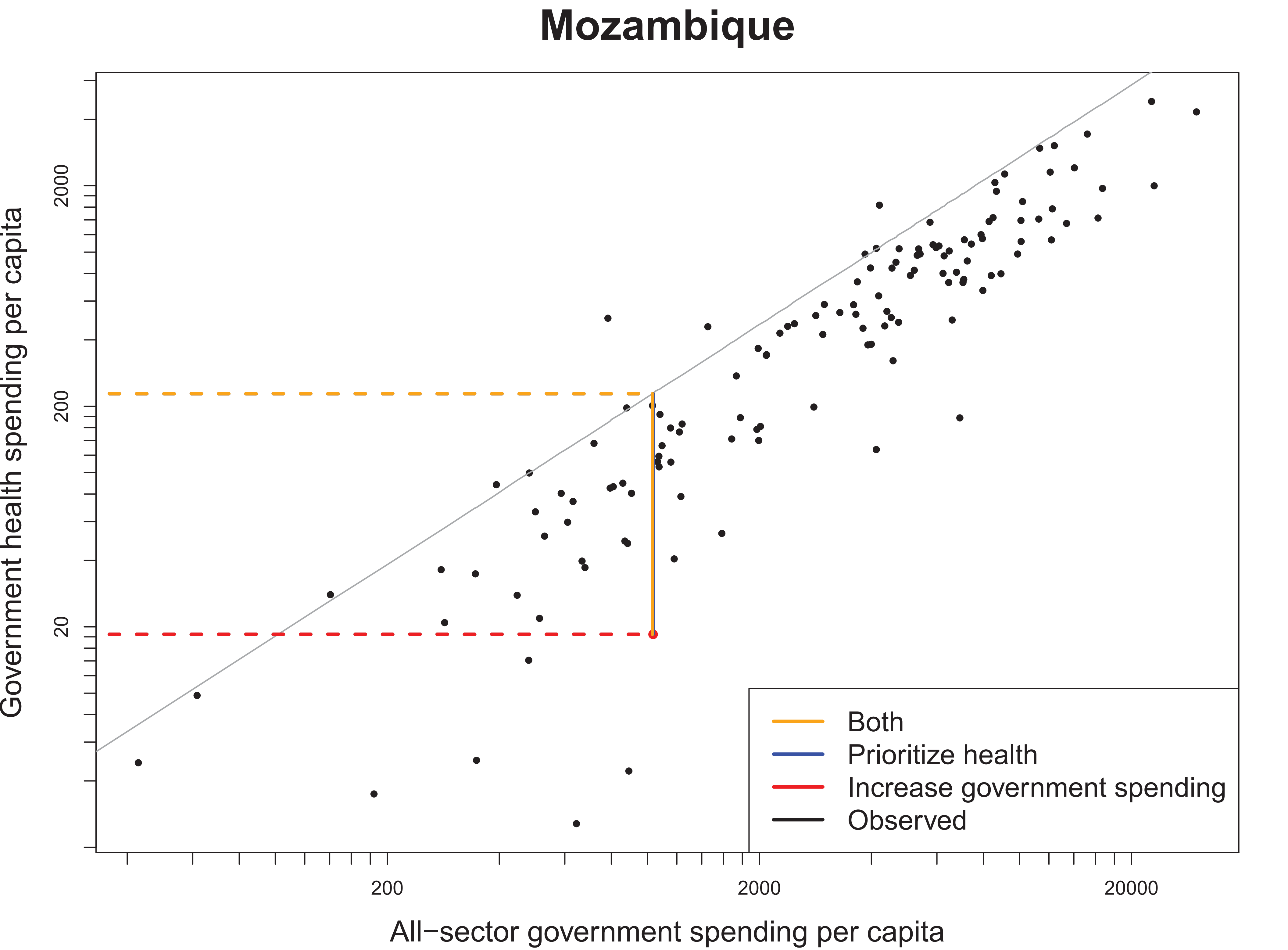

Mozambique
Government health spending per capita
2000
200
20
200
2000
20000
All−sector government spending per capita
Both
Prioritize health
Increase government spending
Observed

# Myanmar

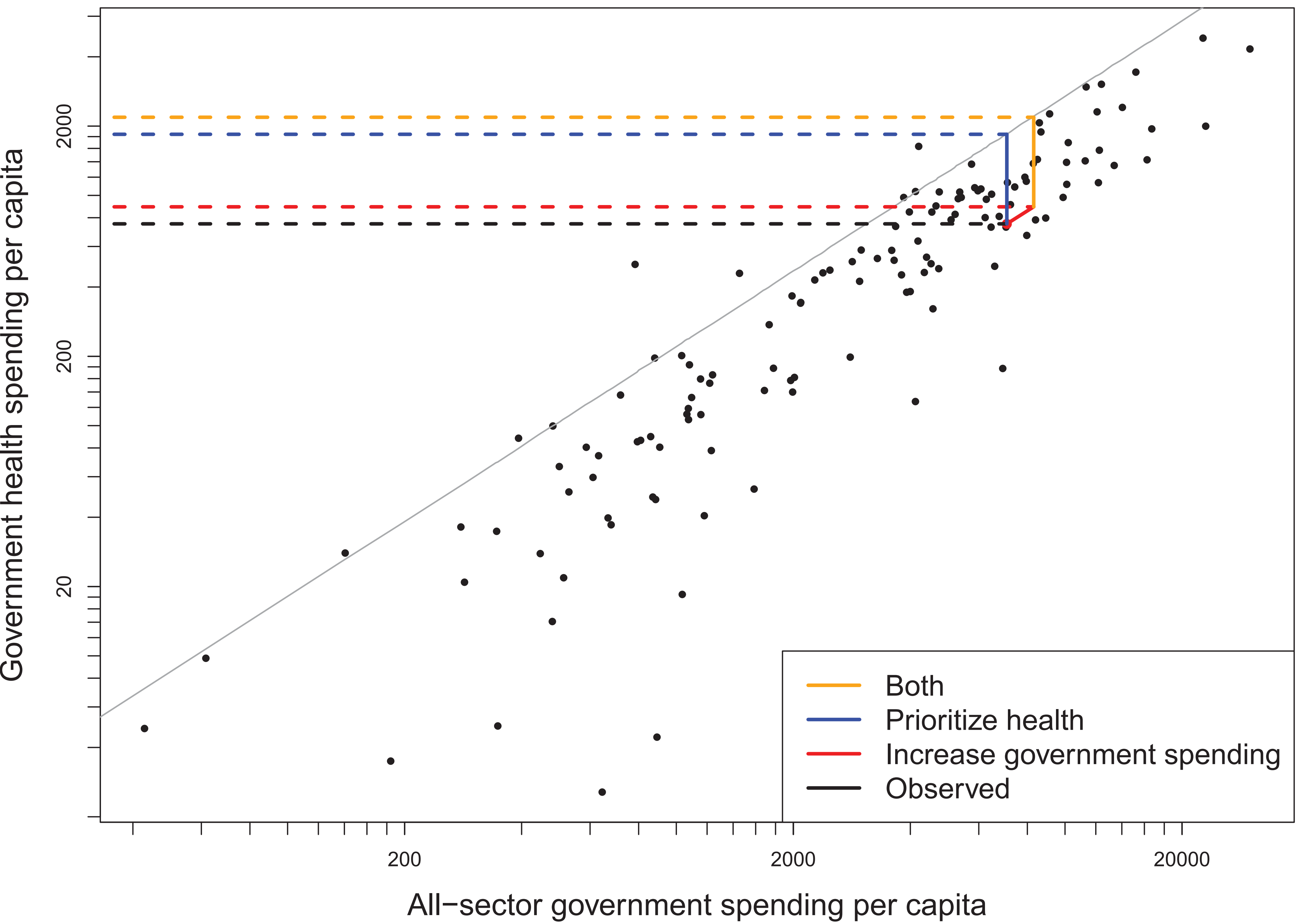
Government health spending per capita
20
200
2000
All−sector government spending per capita
200
2000
20000
Both
Prioritize health
Increase government spending
Observed

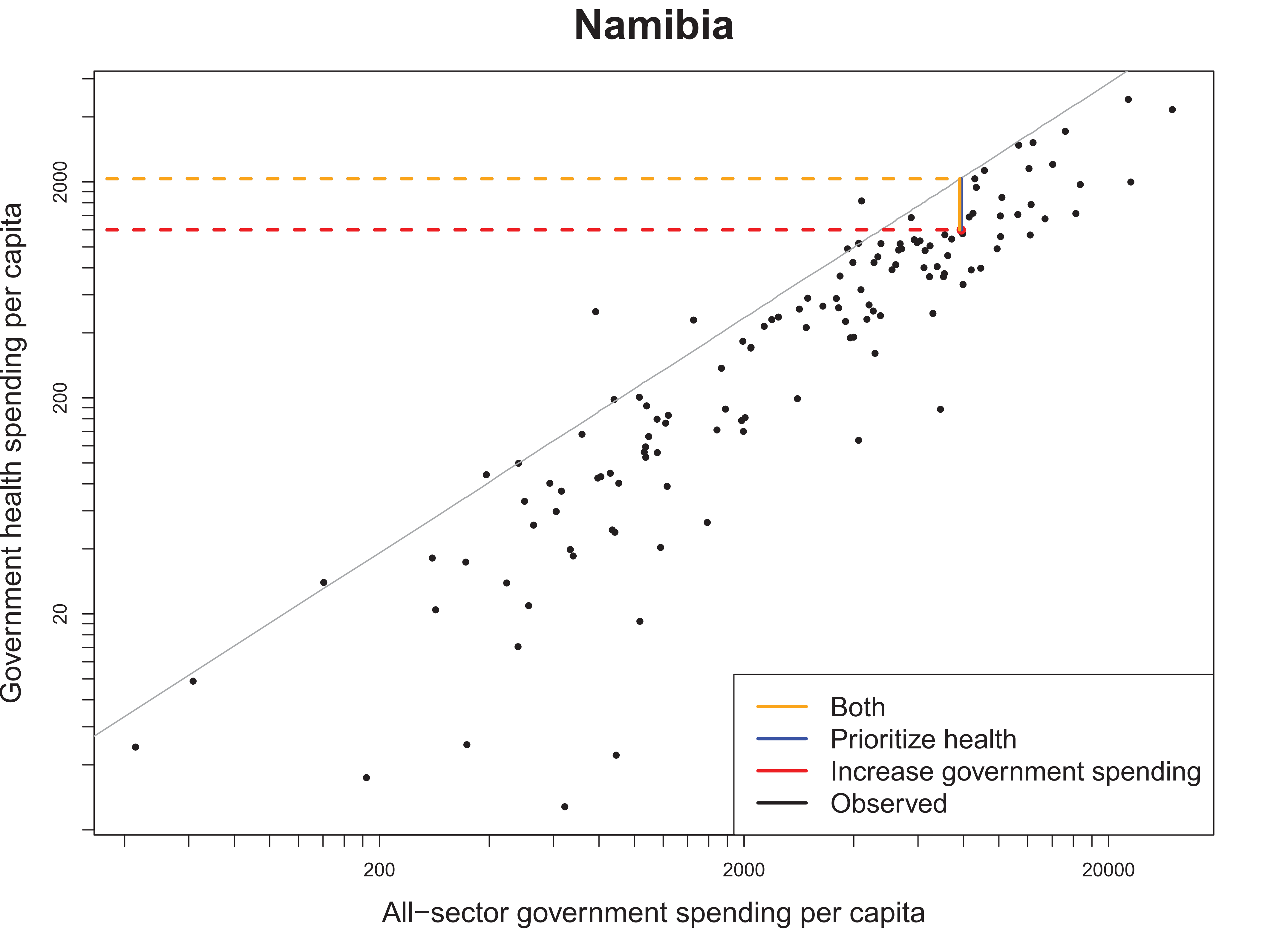

Namibia
Government health spending per capita
All−sector government spending per capita
20
200
2000
20000
Both
Prioritize health
Increase government spending
Observed

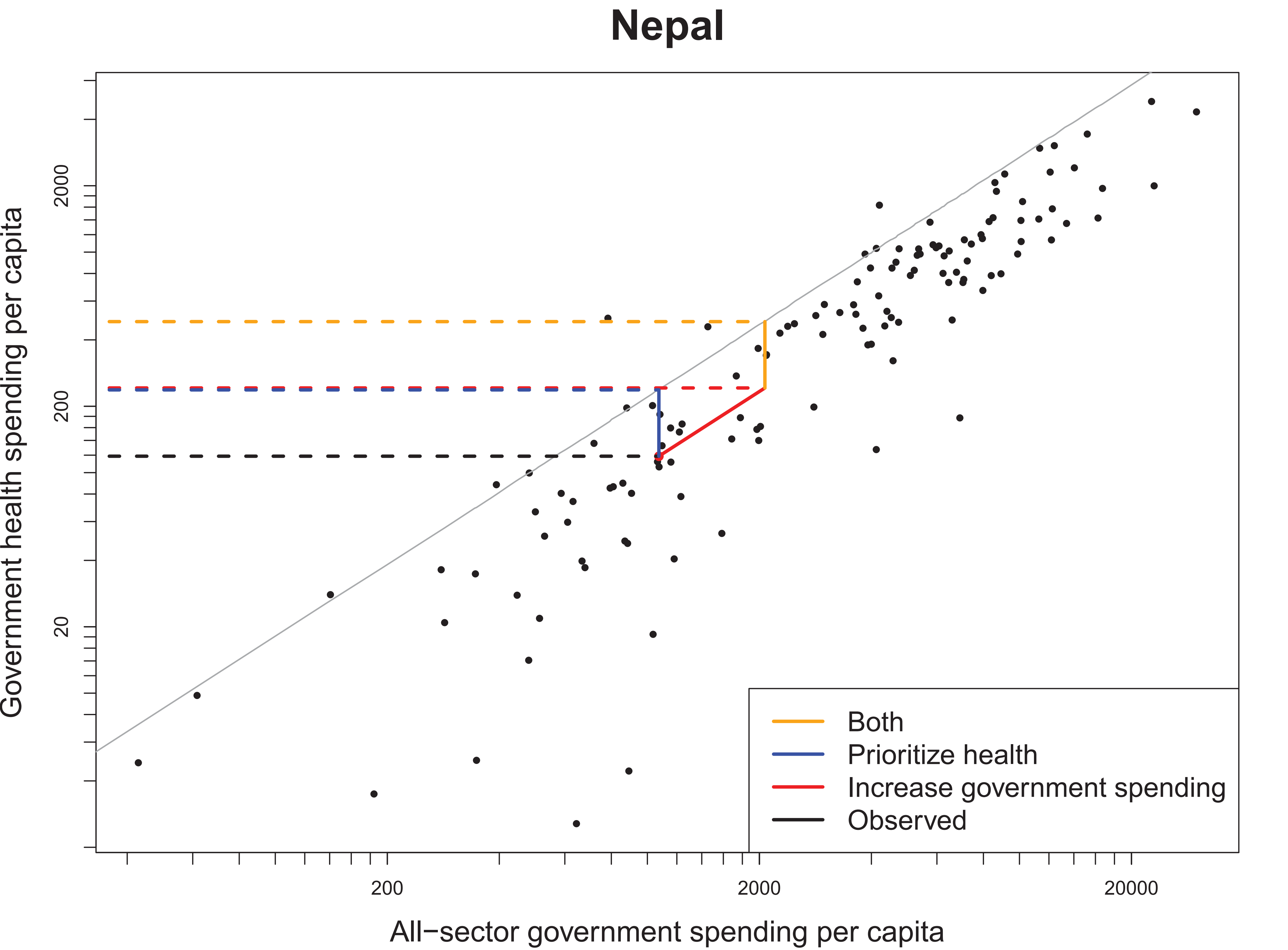
Nepal
Government health spending per capita
2000
200
20
200
2000
20000
All−sector government spending per capita
Both
Prioritize health
Increase government spending
Observed

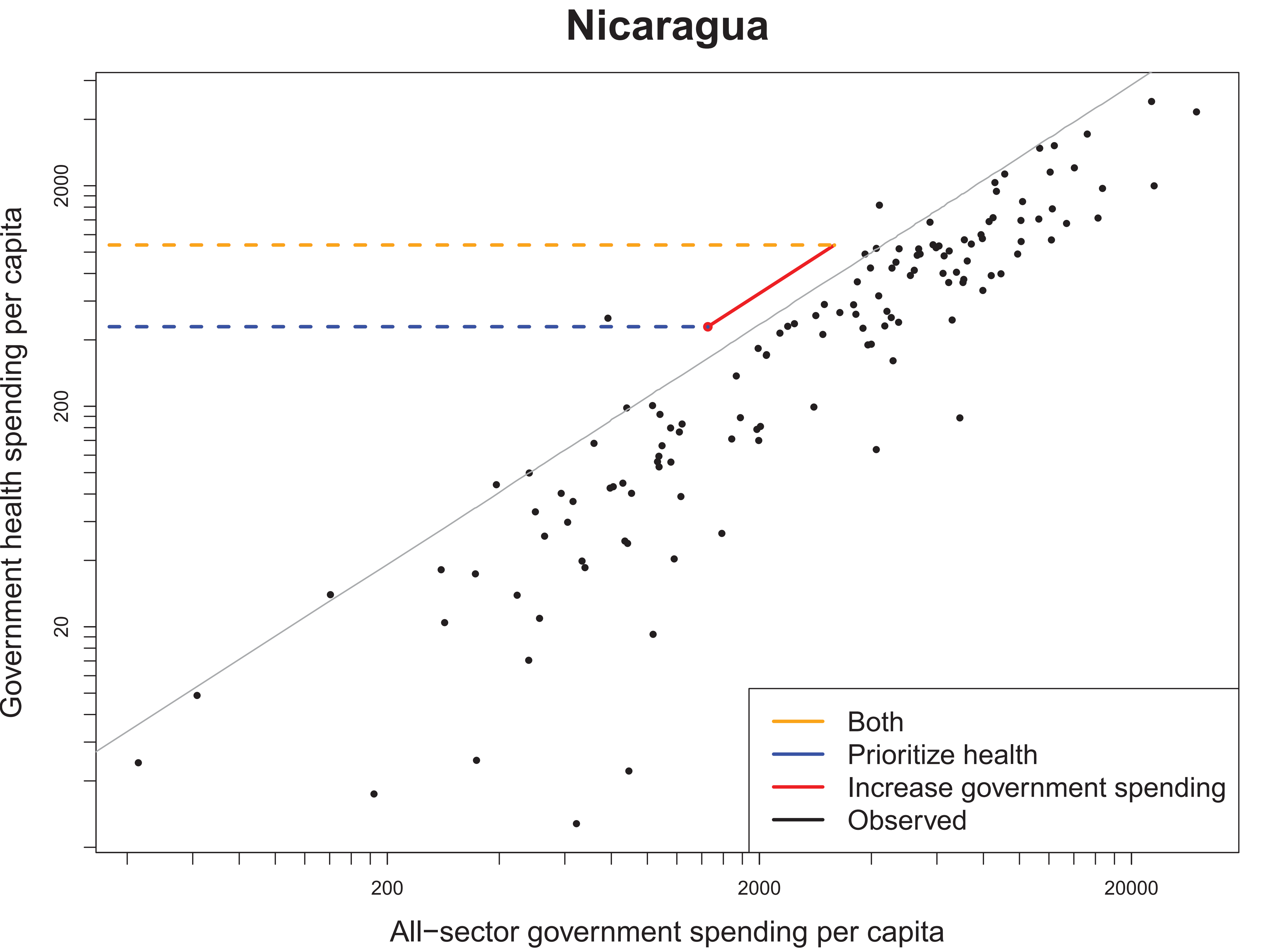

Nicaragua
Both
Prioritize health
Increase government spending
Observed
Government health spending per capita
20
200
2000
All−sector government spending per capita
200
2000
20000

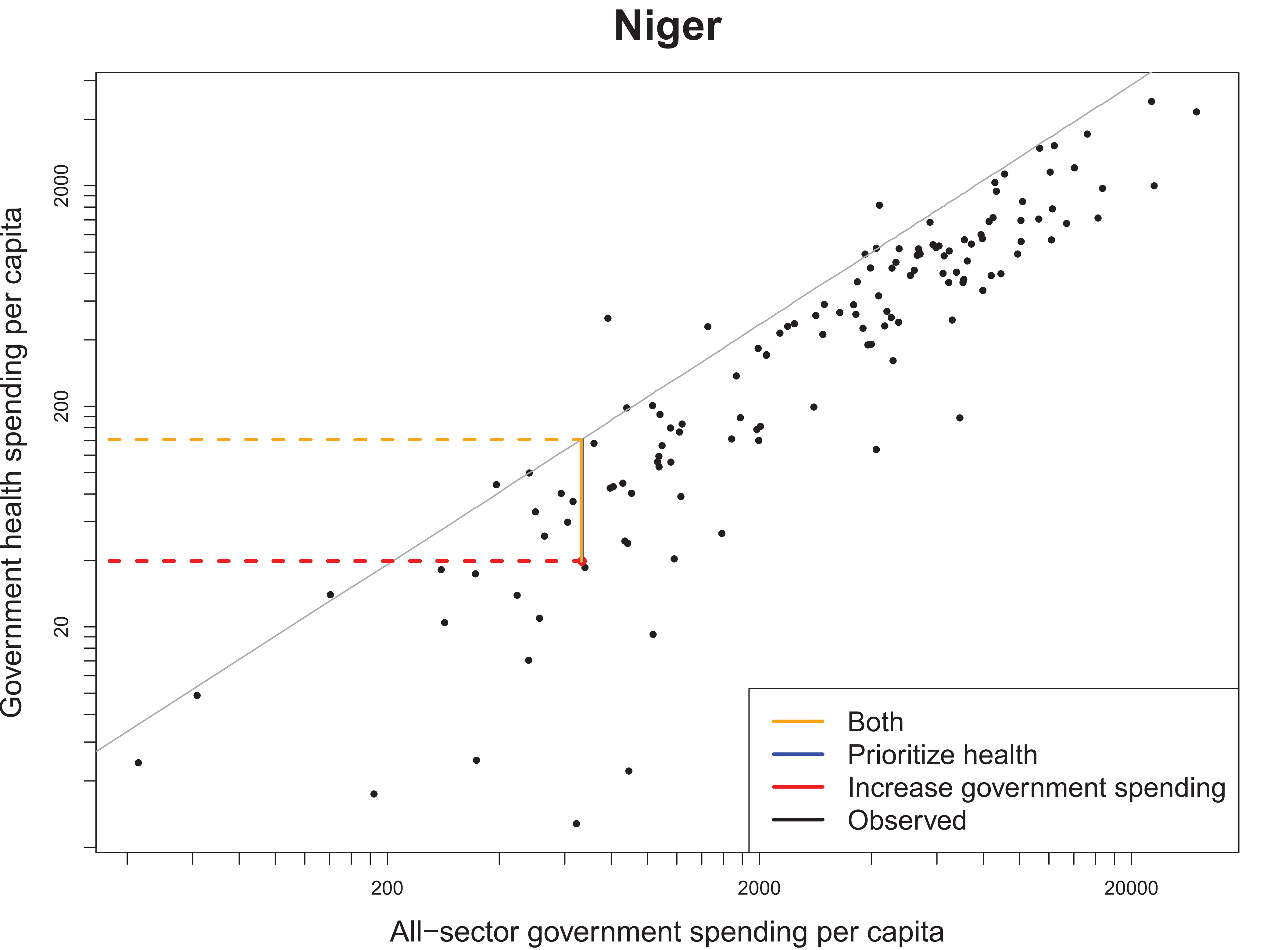

Niger
Government health spending per capita
All−sector government spending per capita
20
200
2000
20000
Both
Prioritize health
Increase government spending
Observed

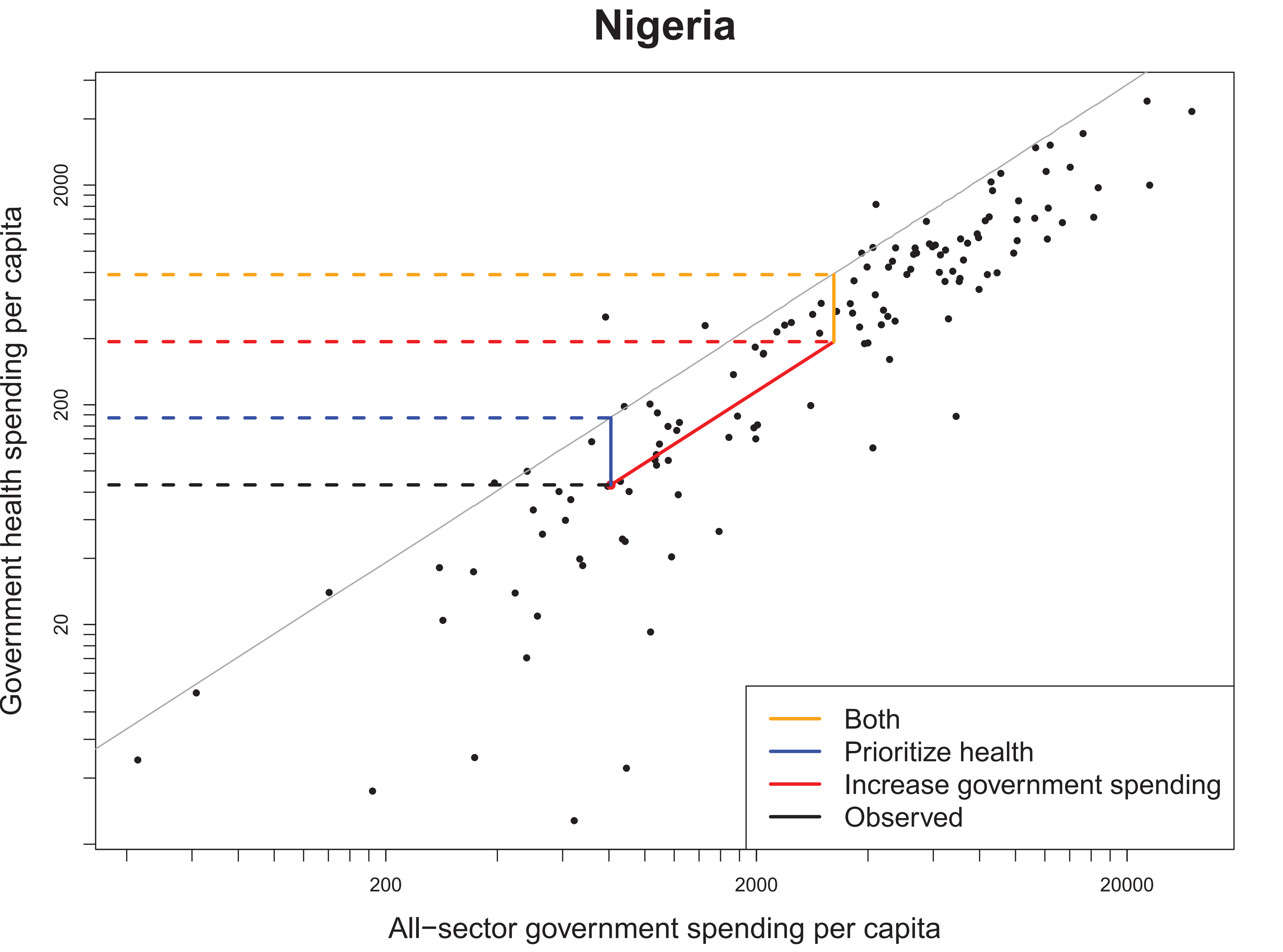

Nigeria
Both
Prioritize health
Increase government spending
Observed
Government health spending per capita
20
200
2000
All−sector government spending per capita
200
2000
20000

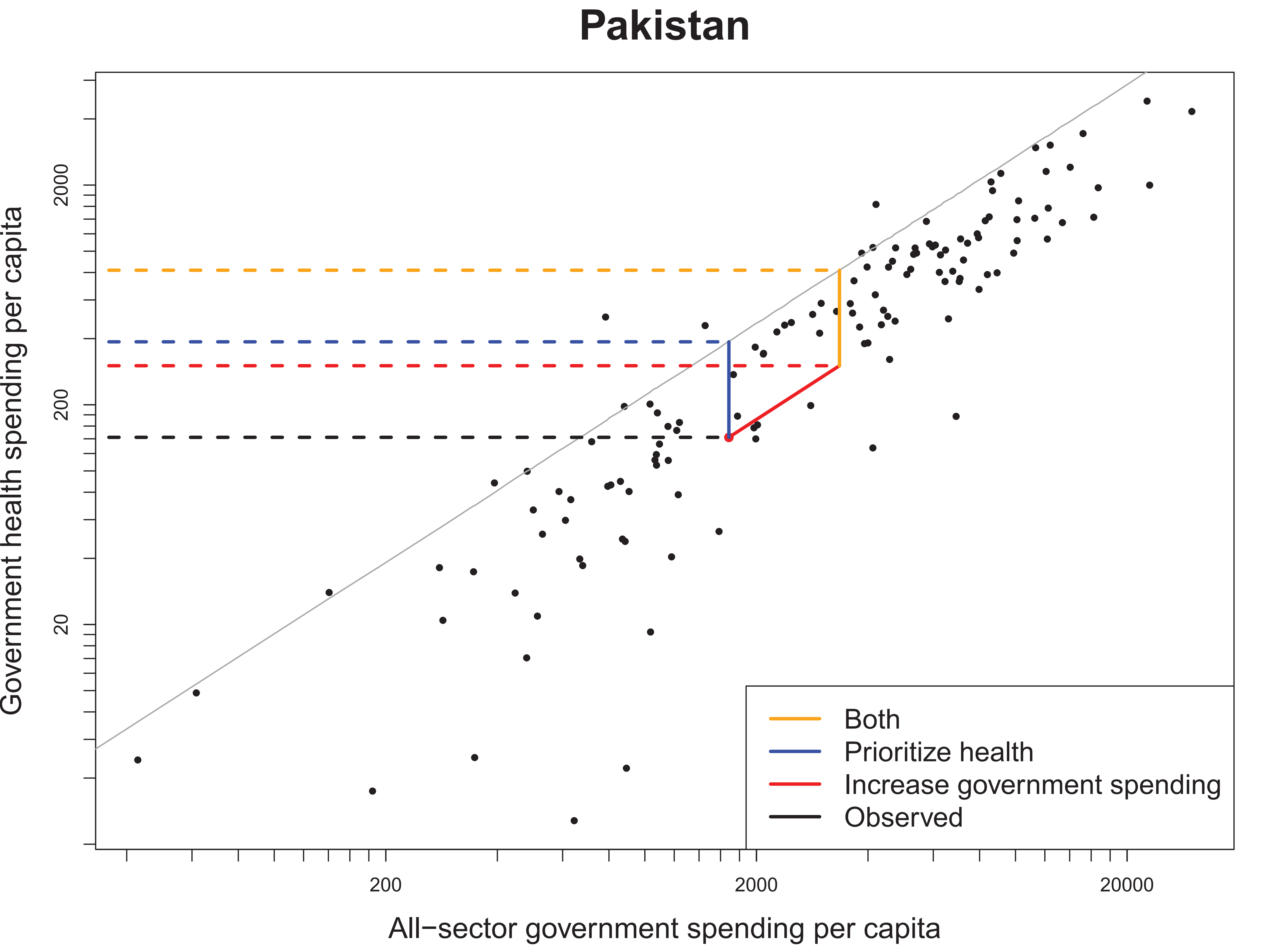

Pakistan
Government health spending per capita
All−sector government spending per capita
20
200
2000
200
2000
20000
Both
Prioritize health
Increase government spending
Observed

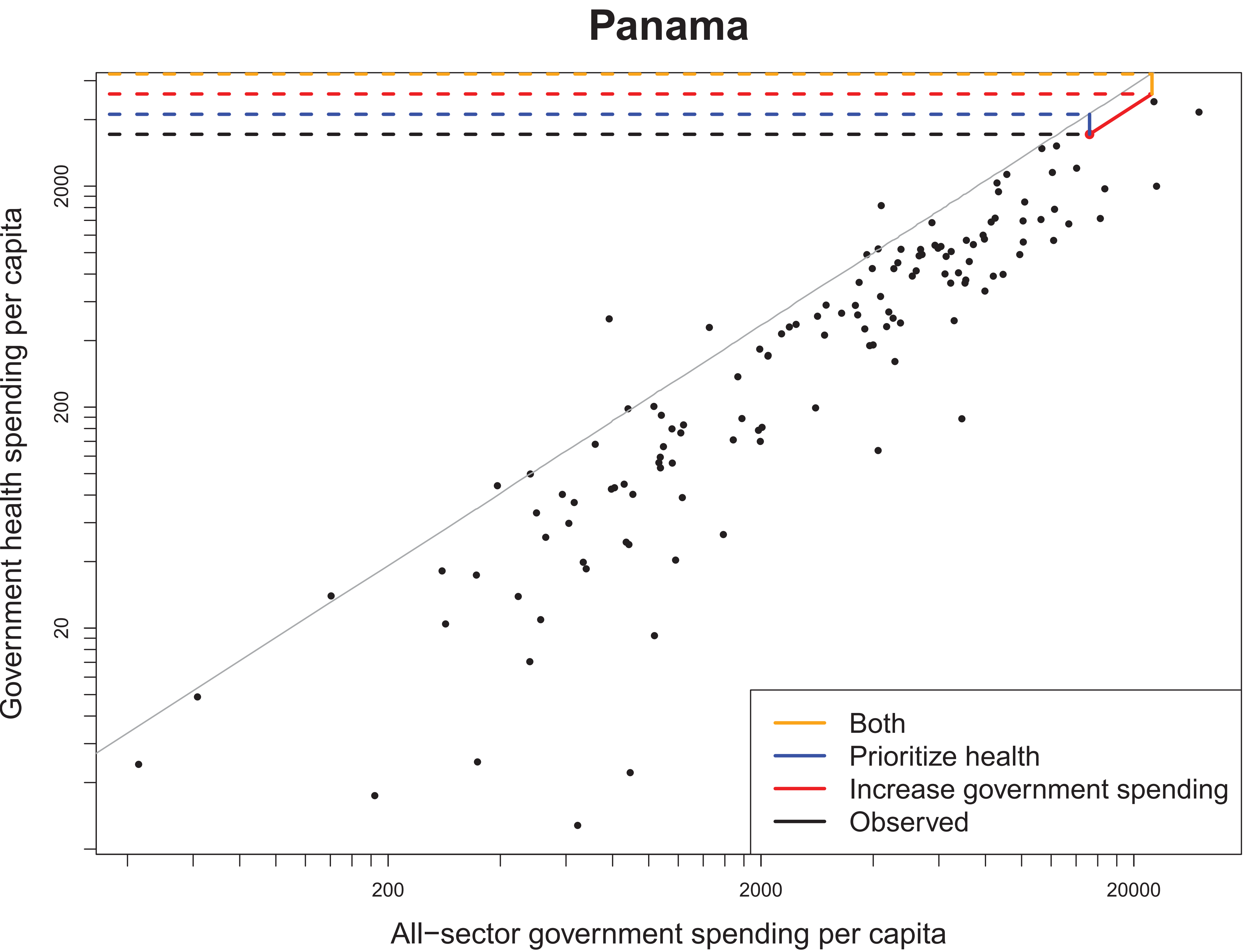

Panama
Government health spending per capita
All−sector government spending per capita
20
200
2000
200
2000
20000
Both
Prioritize health
Increase government spending
Observed

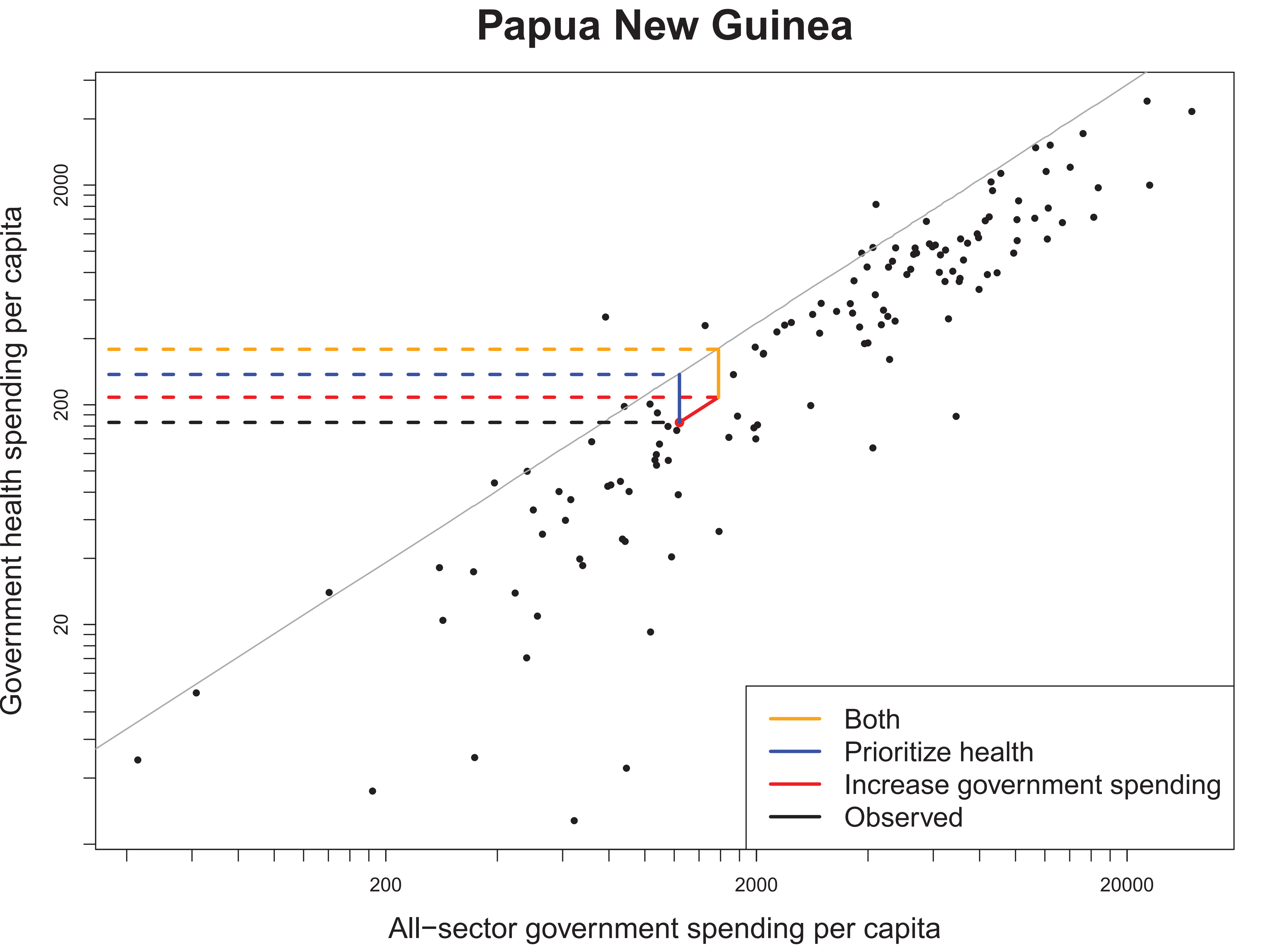
Papua New Guinea
Government health spending per capita
20
200
2000
All−sector government spending per capita
200
2000
20000
Both
Prioritize health
Increase government spending
Observed

# Paraguay

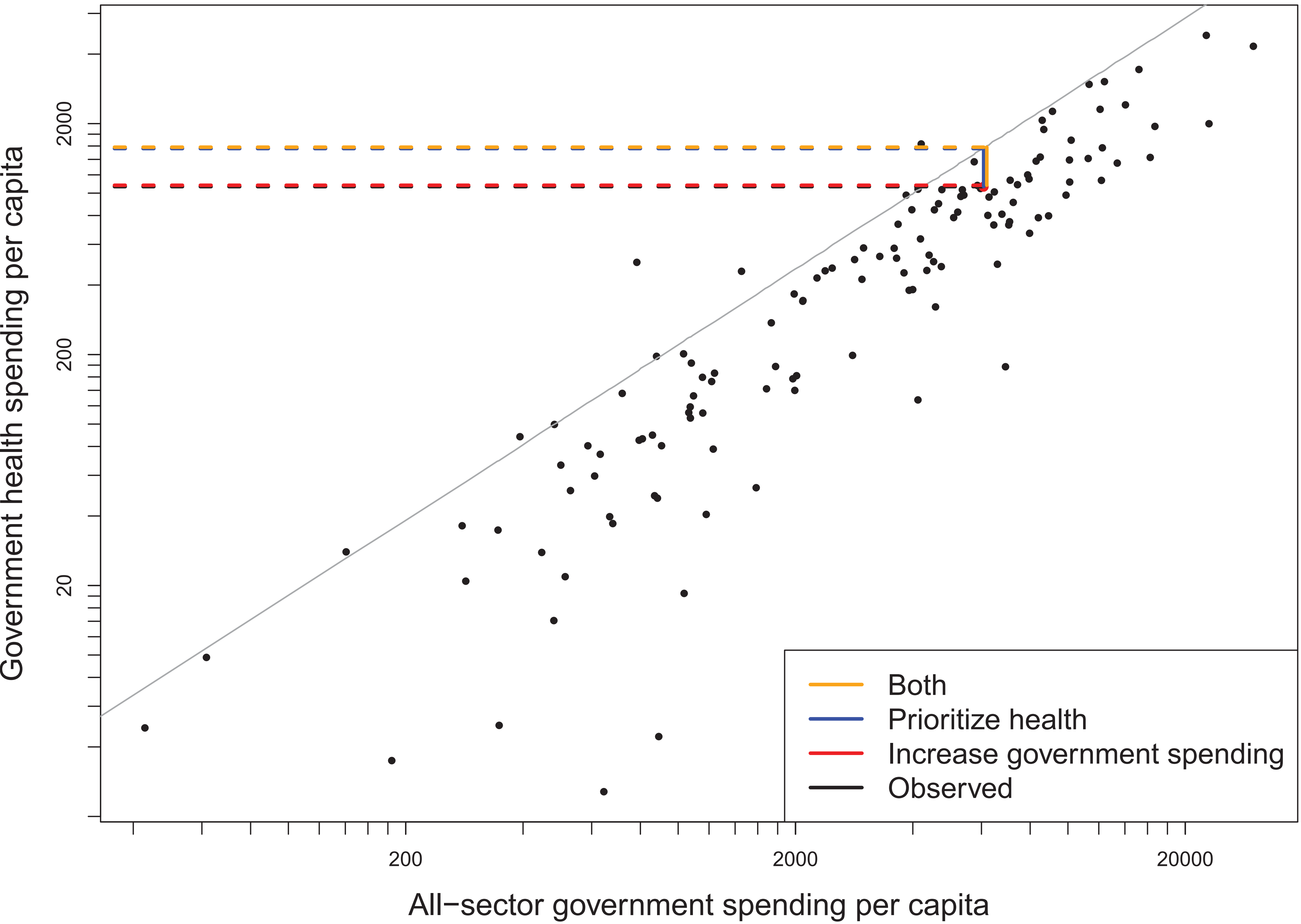

Government health spending per capita
20
200
2000
All−sector government spending per capita
200
2000
20000
Both
Prioritize health
Increase government spending
Observed

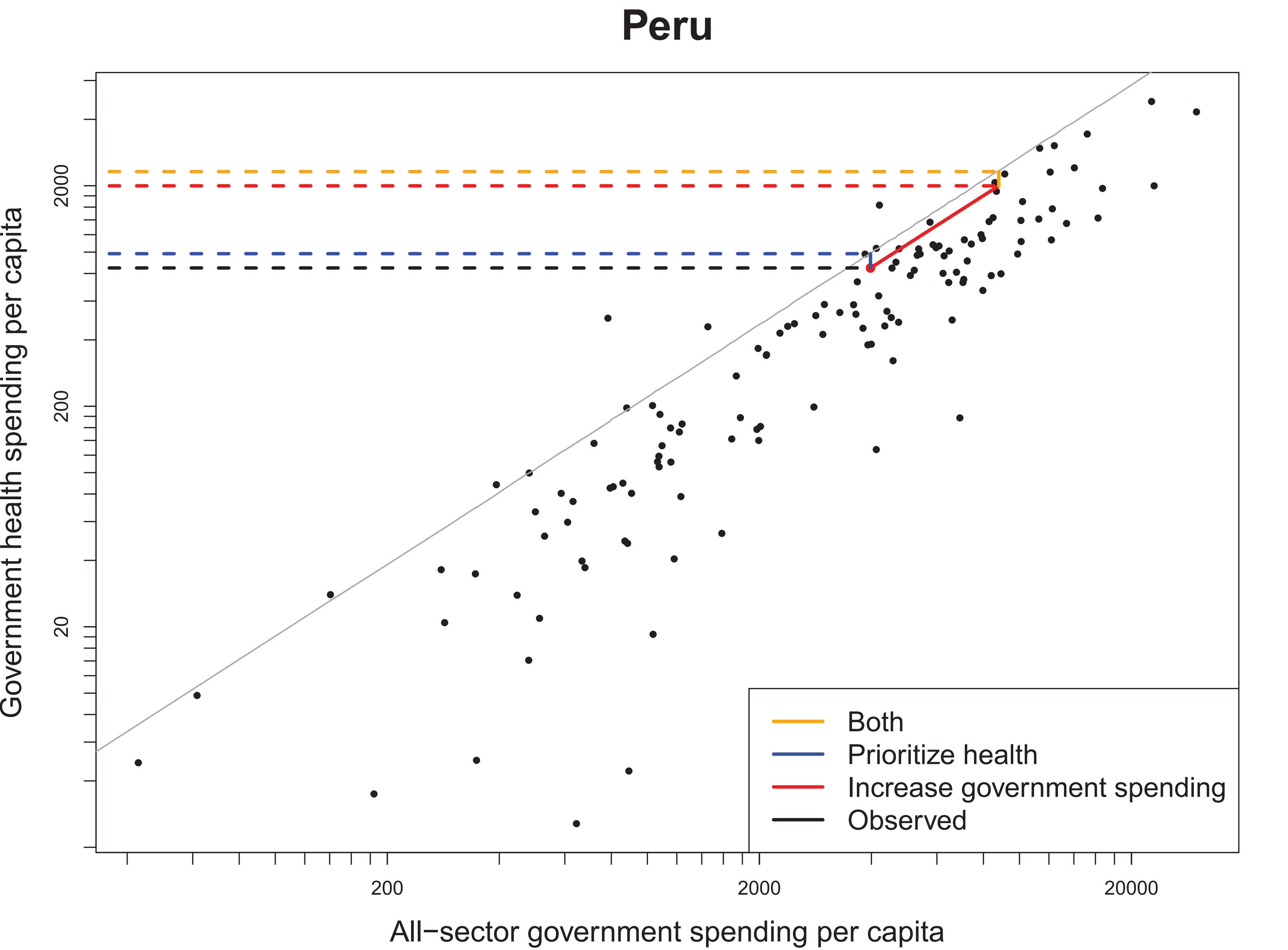

Peru
Government health spending per capita
2000
200
20
All−sector government spending per capita
200
2000
20000
Both
Prioritize health
Increase government spending
Observed

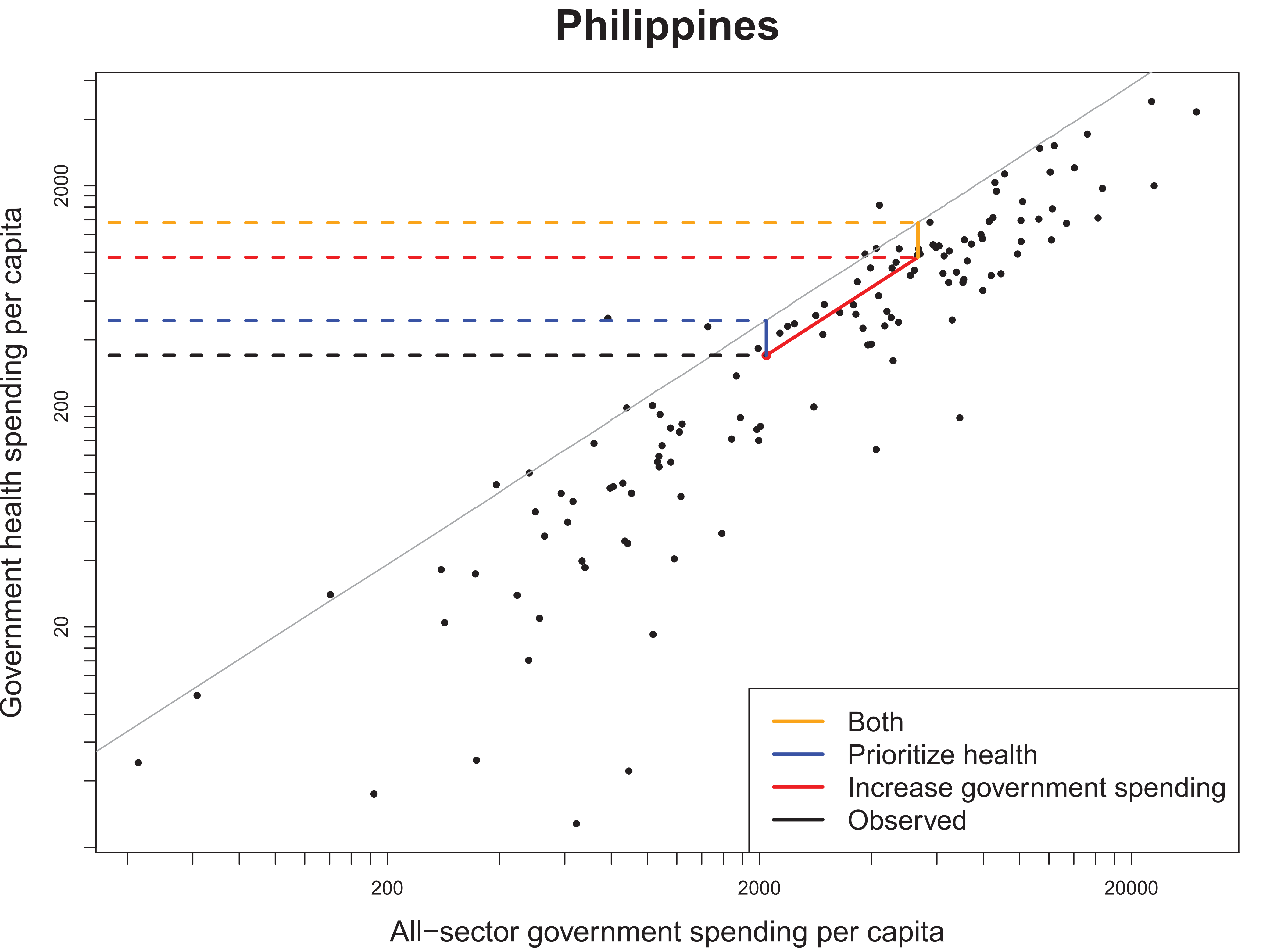

Philippines
Government health spending per capita
All−sector government spending per capita
20
200
2000
200
2000
20000
Both
Prioritize health
Increase government spending
Observed

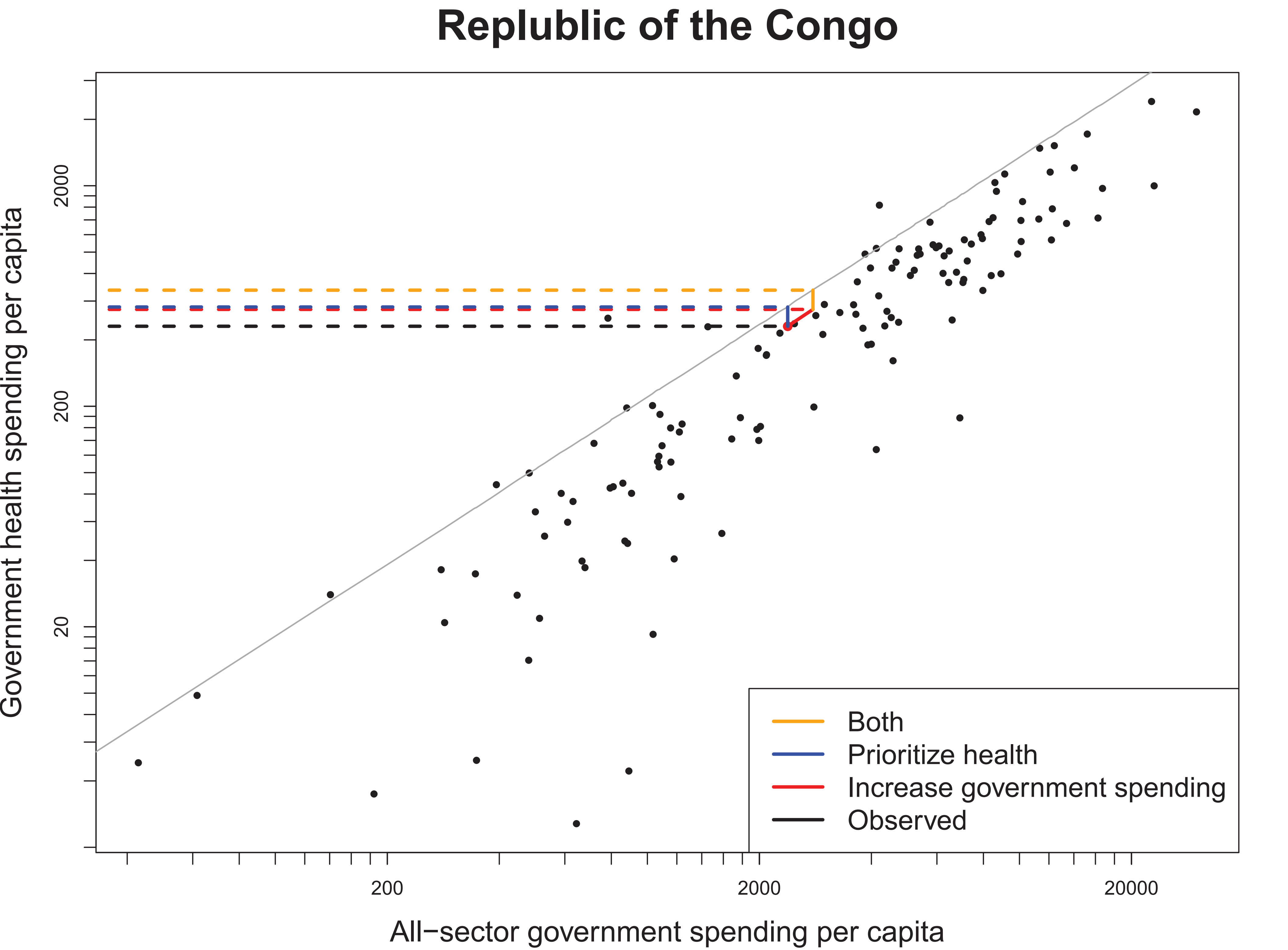

Replublic of the Congo
Government health spending per capita
All−sector government spending per capita
20
200
2000
200
2000
20000
Both
Prioritize health
Increase government spending
Observed

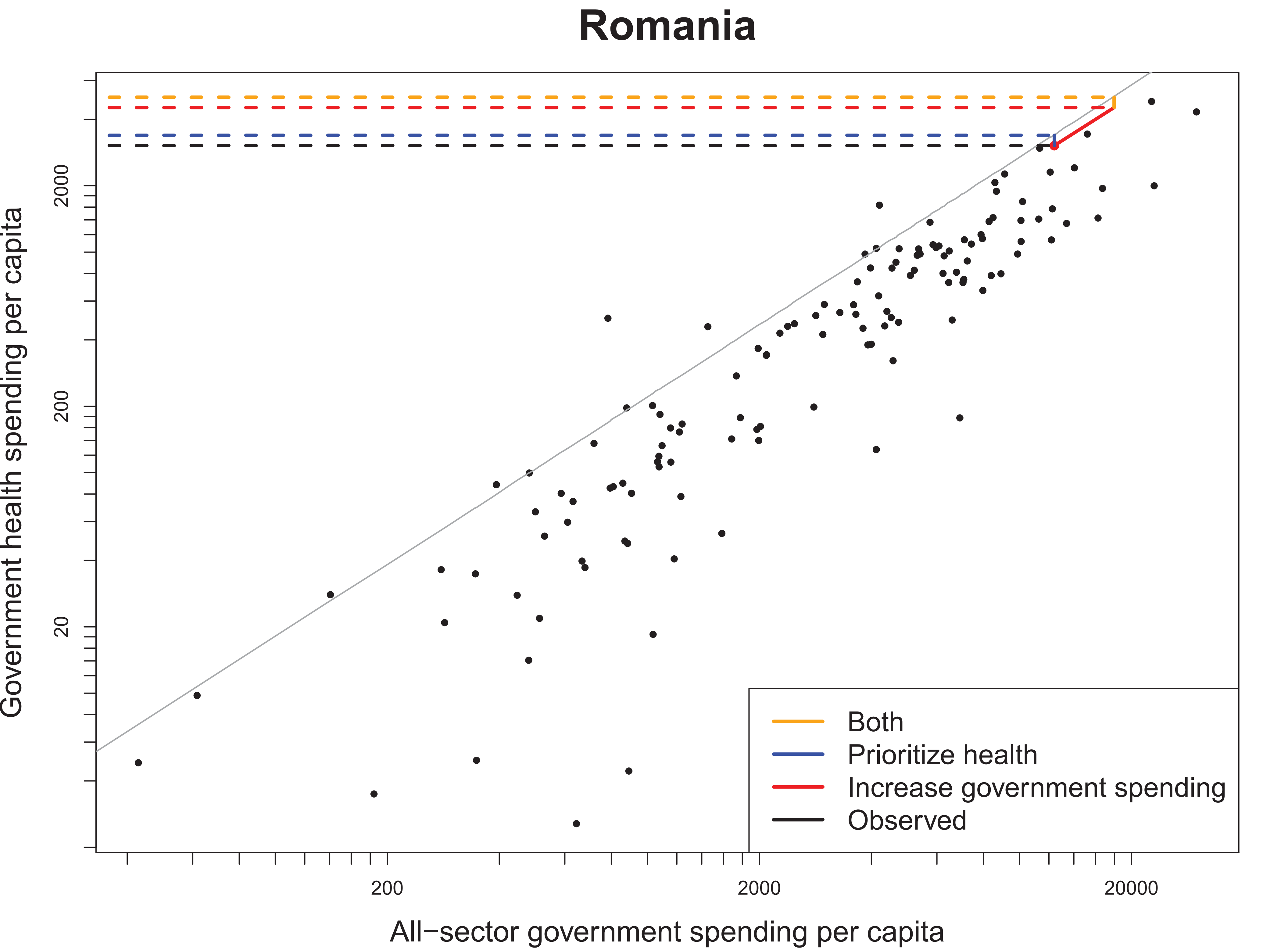
Romania
Government health spending per capita
All−sector government spending per capita
20
200
2000
200
2000
20000
Both
Prioritize health
Increase government spending
Observed

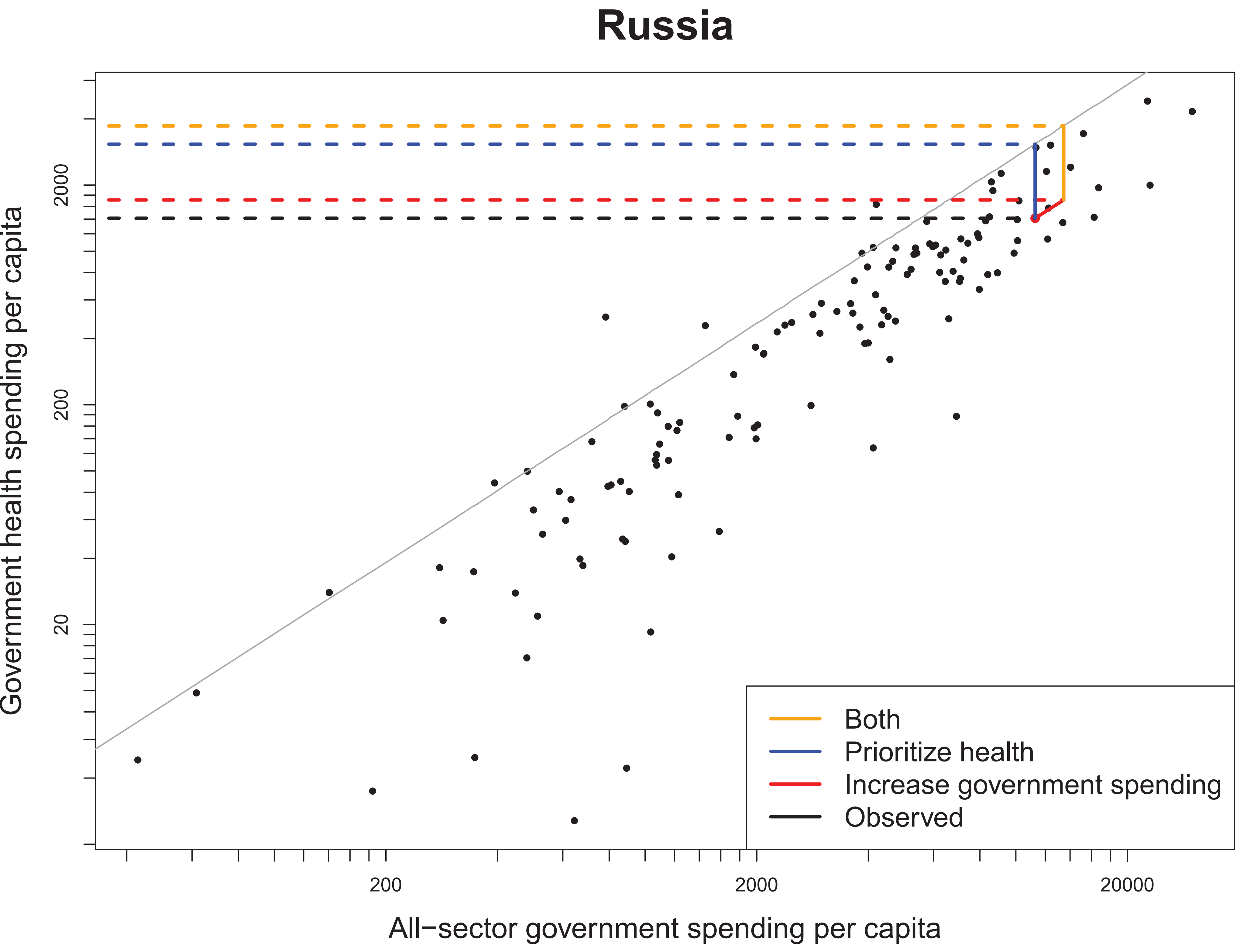

Russia
Government health spending per capita
All−sector government spending per capita
20
200
2000
20000
Both
Prioritize health
Increase government spending
Observed

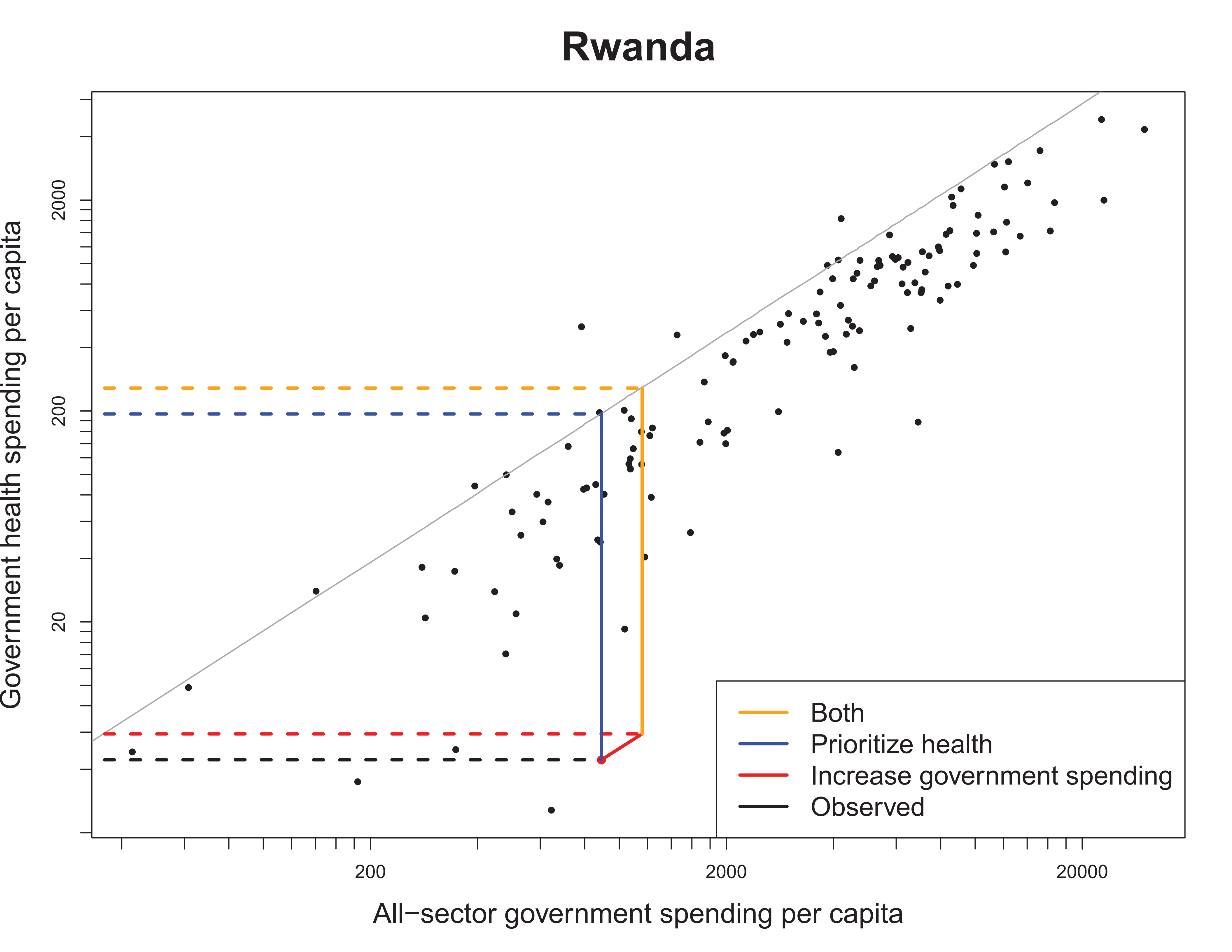
Rwanda
Government health spending per capita
20
200
2000
All−sector government spending per capita
200
2000
20000
Both
Prioritize health
Increase government spending
Observed

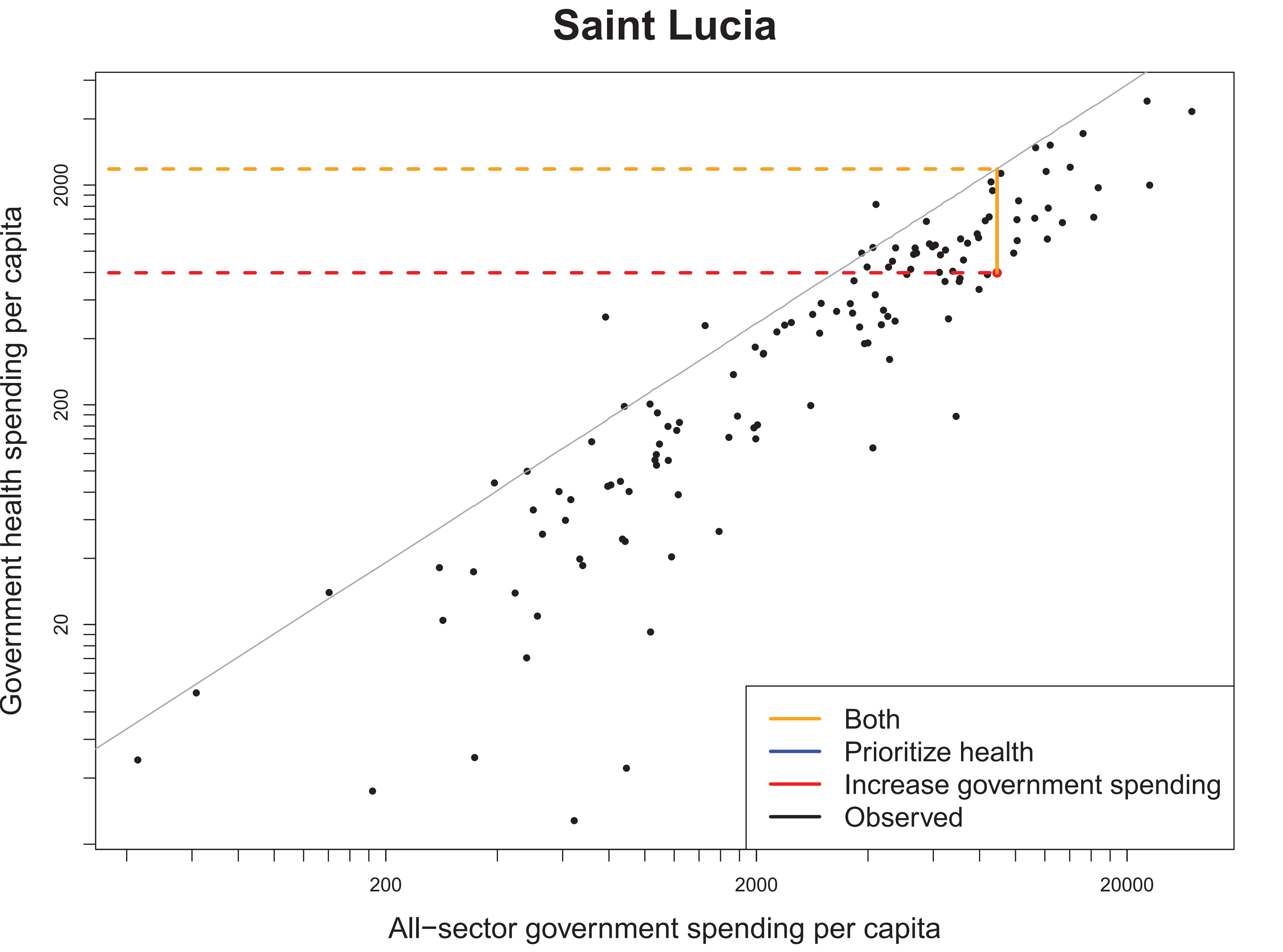
Saint Lucia
Government health spending per capita
All−sector government spending per capita
20
200
2000
200
2000
20000
Both
Prioritize health
Increase government spending
Observed

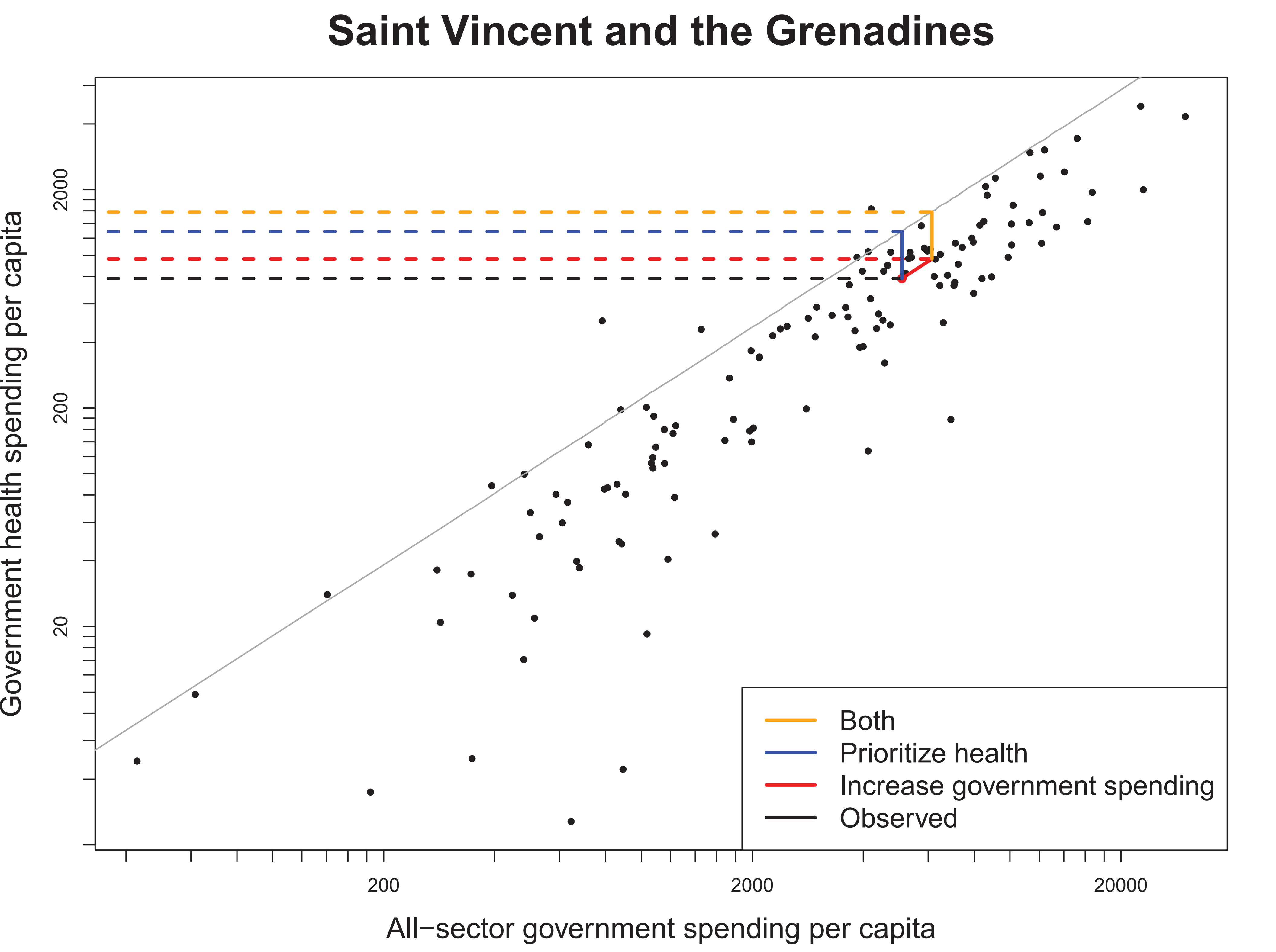

Saint Vincent and the Grenadines
Government health spending per capita
All−sector government spending per capita
20
200
2000
20000
Both
Prioritize health
Increase government spending
Observed

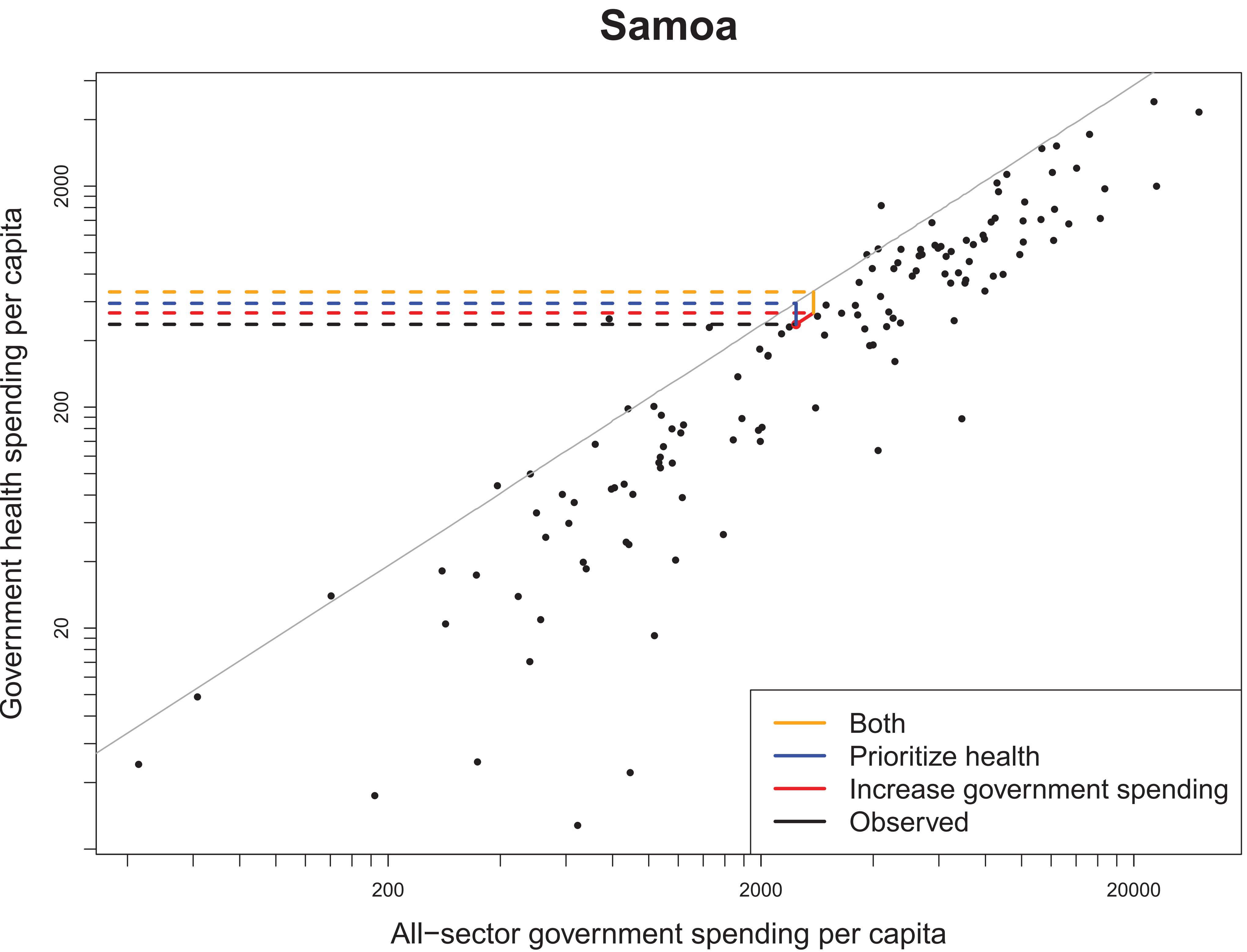

Samoa
Government health spending per capita
All−sector government spending per capita
20
200
2000
20000
Both
Prioritize health
Increase government spending
Observed

# Sao Tome and Principe

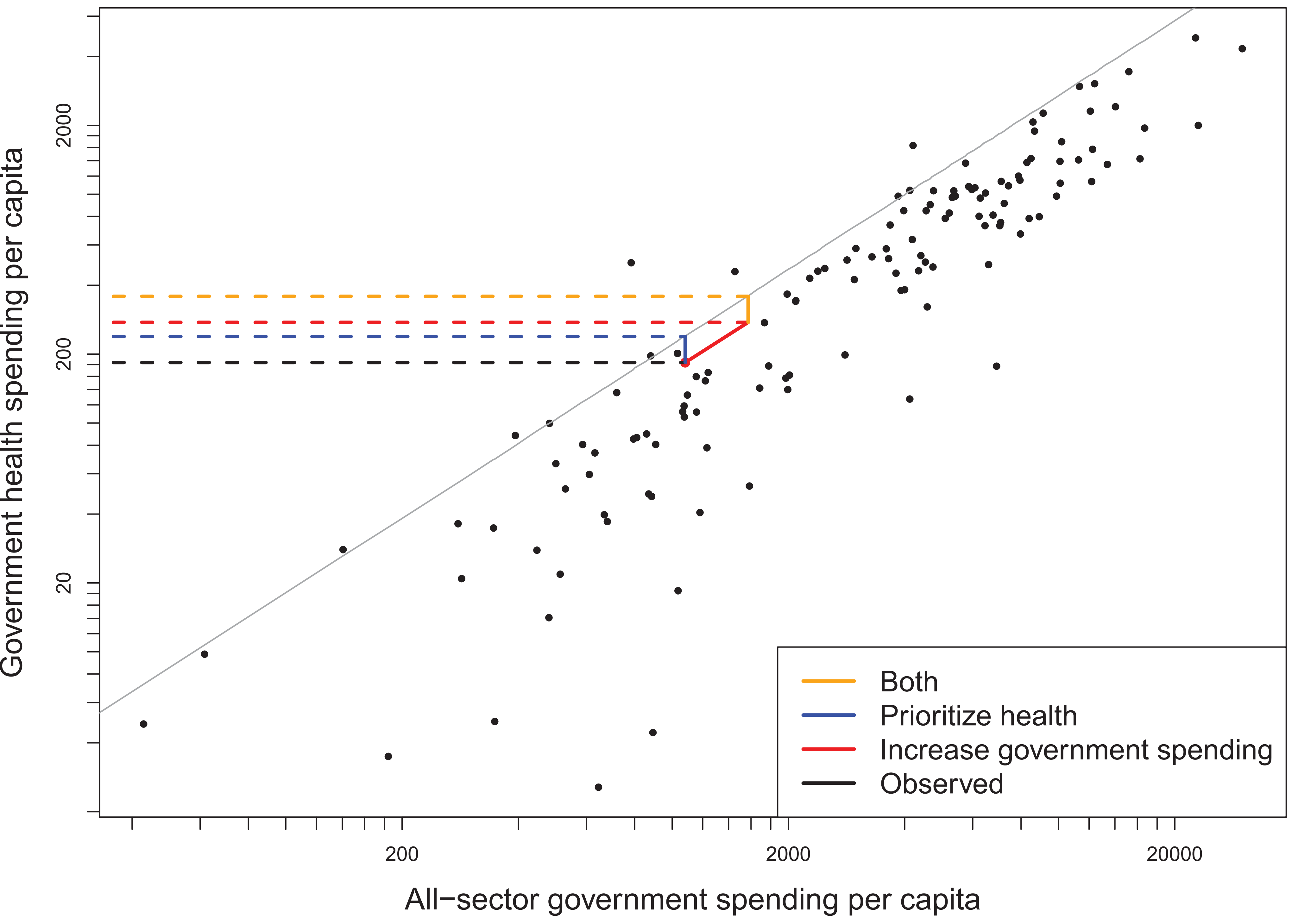

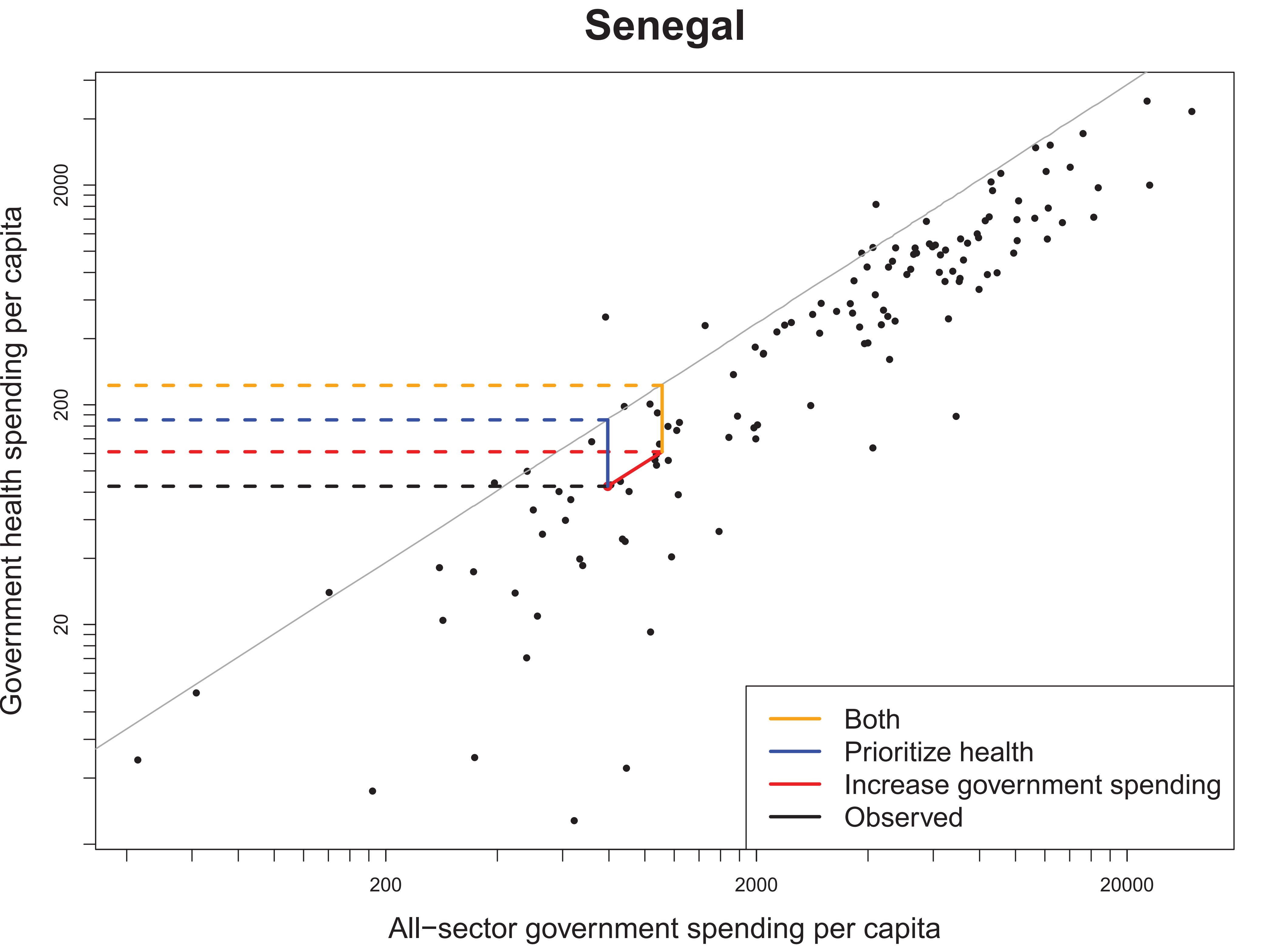

Senegal
Government health spending per capita
All−sector government spending per capita
20
200
2000
20000
Both
Prioritize health
Increase government spending
Observed

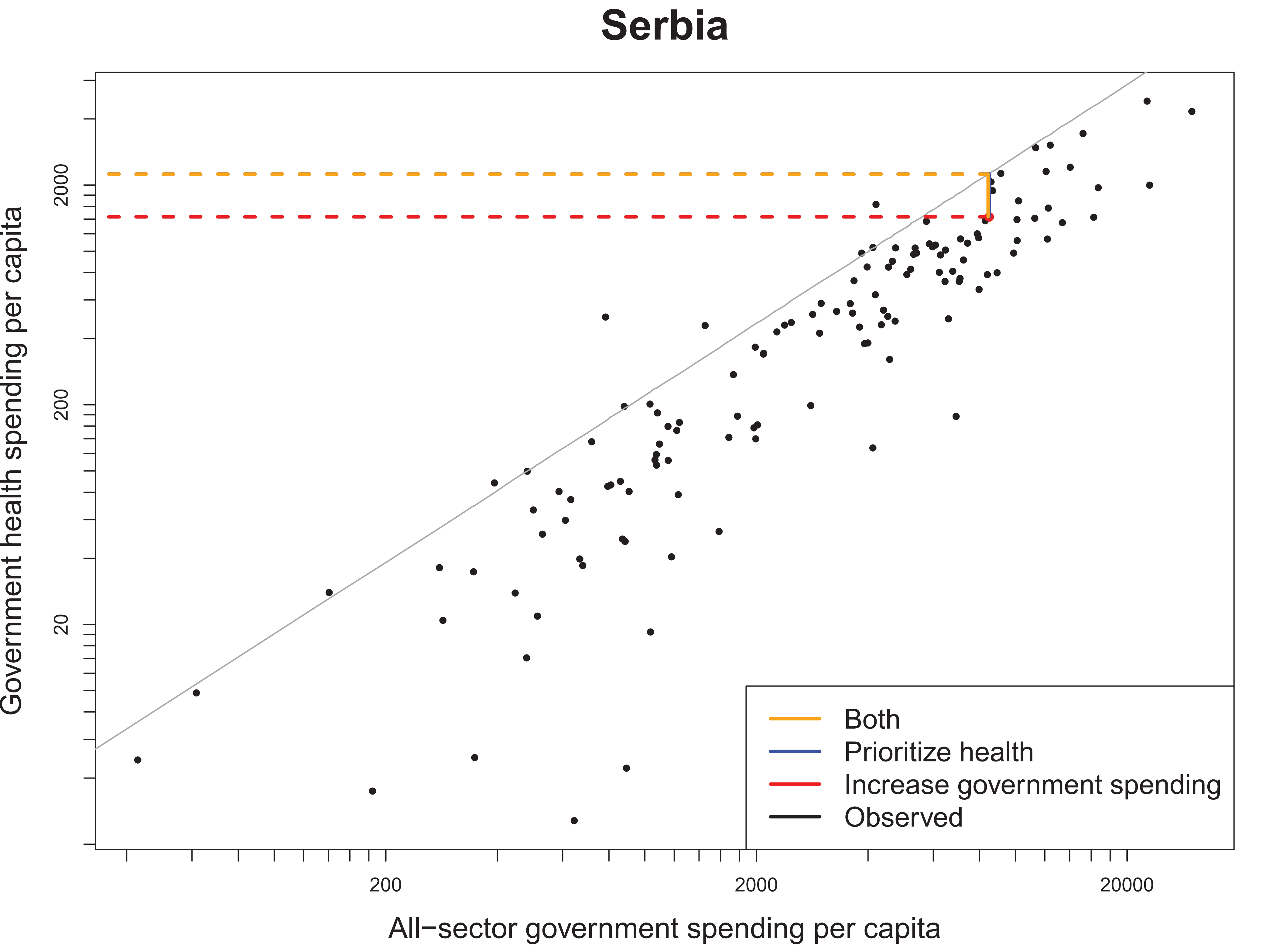

Serbia
Government health spending per capita
All−sector government spending per capita
20
200
2000
20000
Both
Prioritize health
Increase government spending
Observed

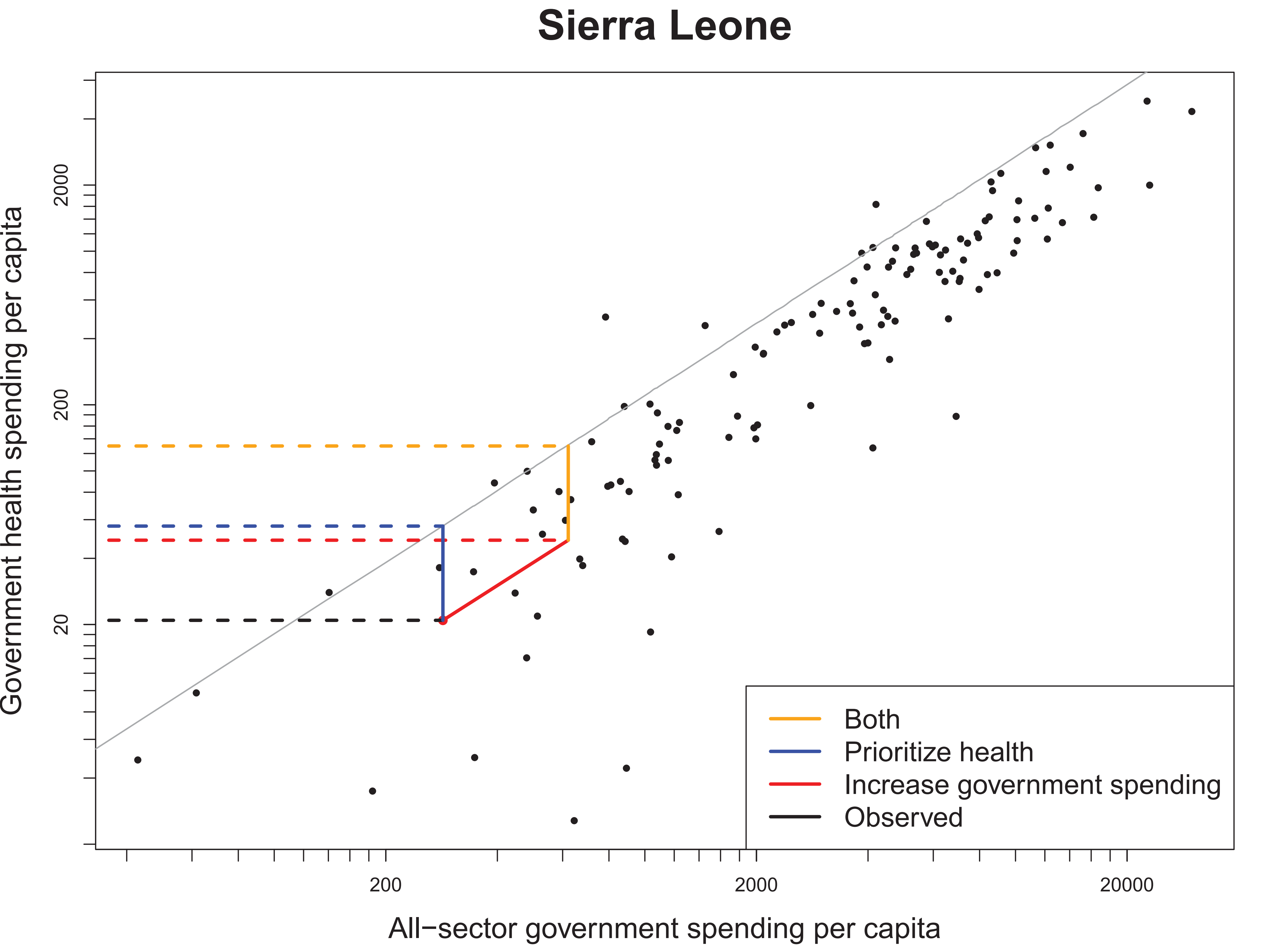

Sierra Leone
Government health spending per capita
20
200
2000
All−sector government spending per capita
200
2000
20000
Both
Prioritize health
Increase government spending
Observed

# Solomon Islands

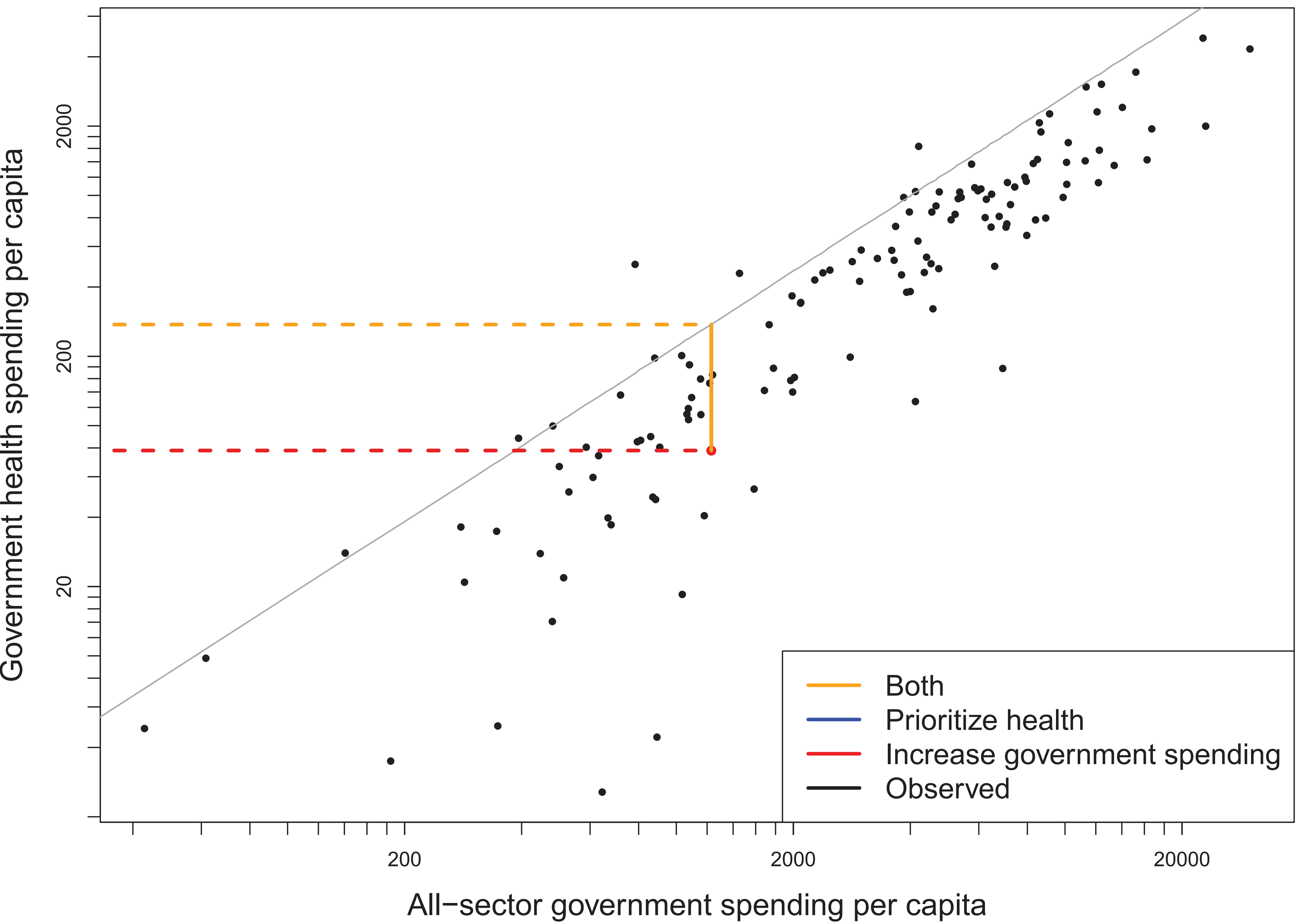

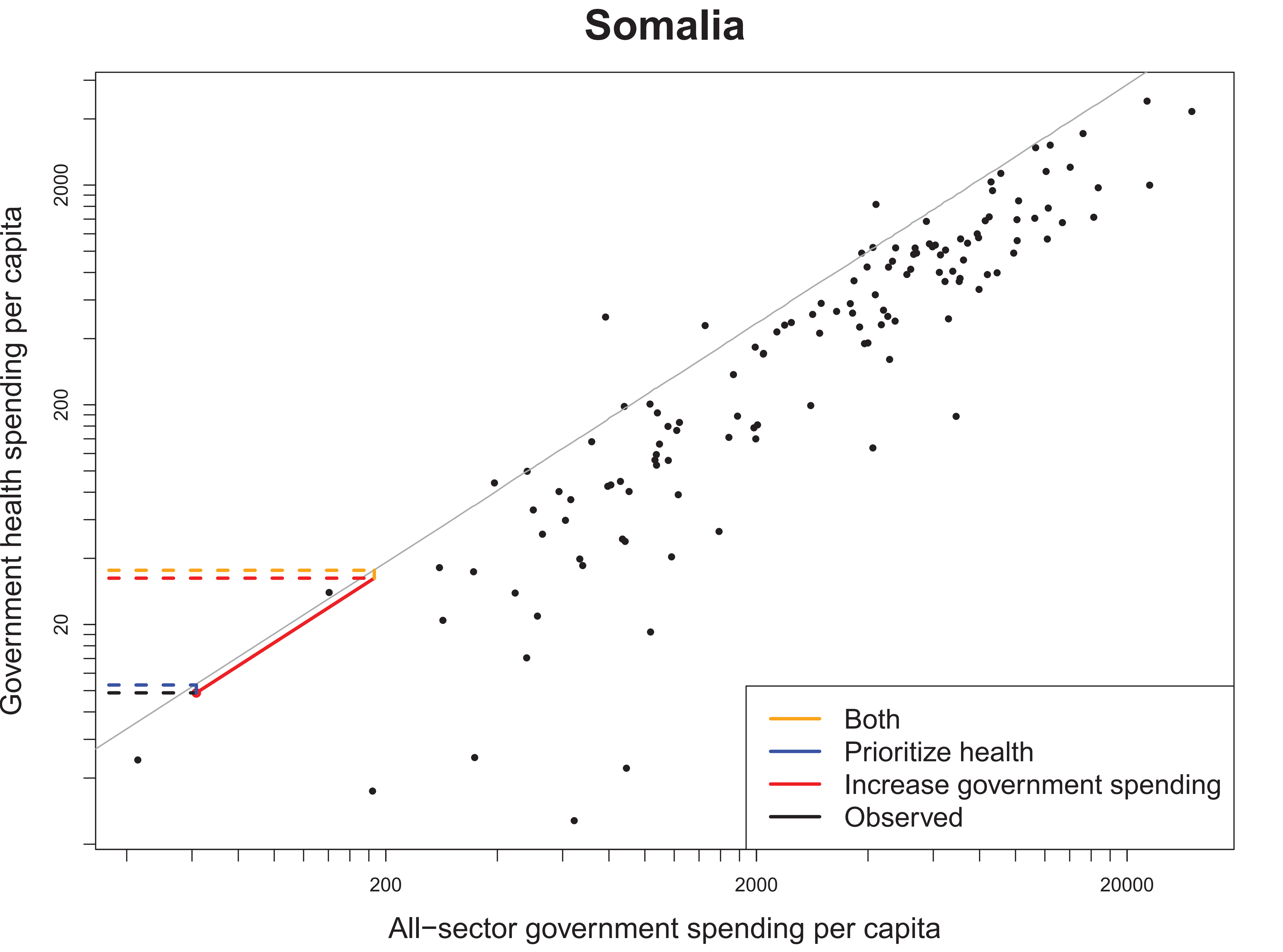

Somalia
Government health spending per capita
All−sector government spending per capita
20
200
2000
20000
Both
Prioritize health
Increase government spending
Observed

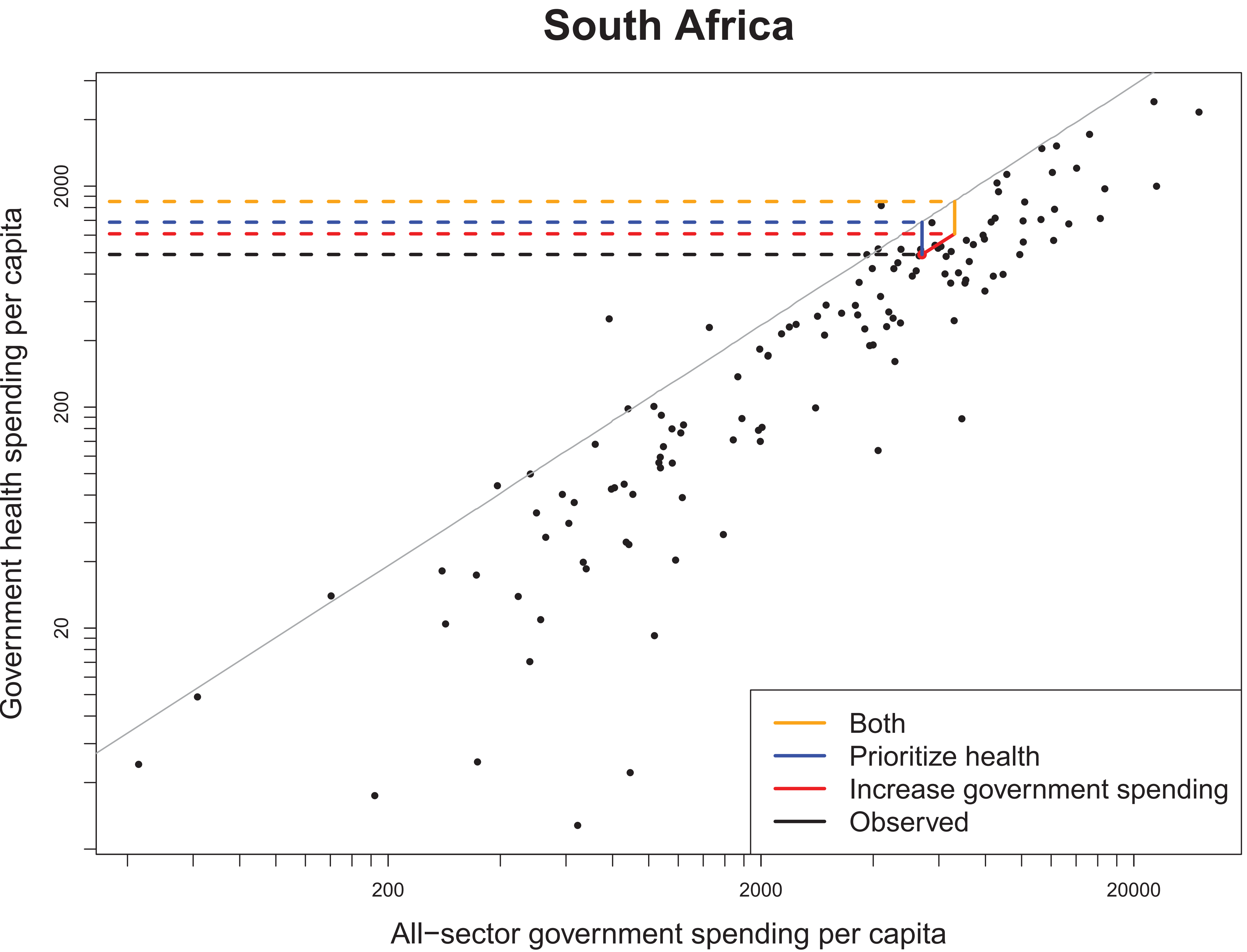
South Africa
Government health spending per capita
All−sector government spending per capita
20
200
2000
20000
Both
Prioritize health
Increase government spending
Observed

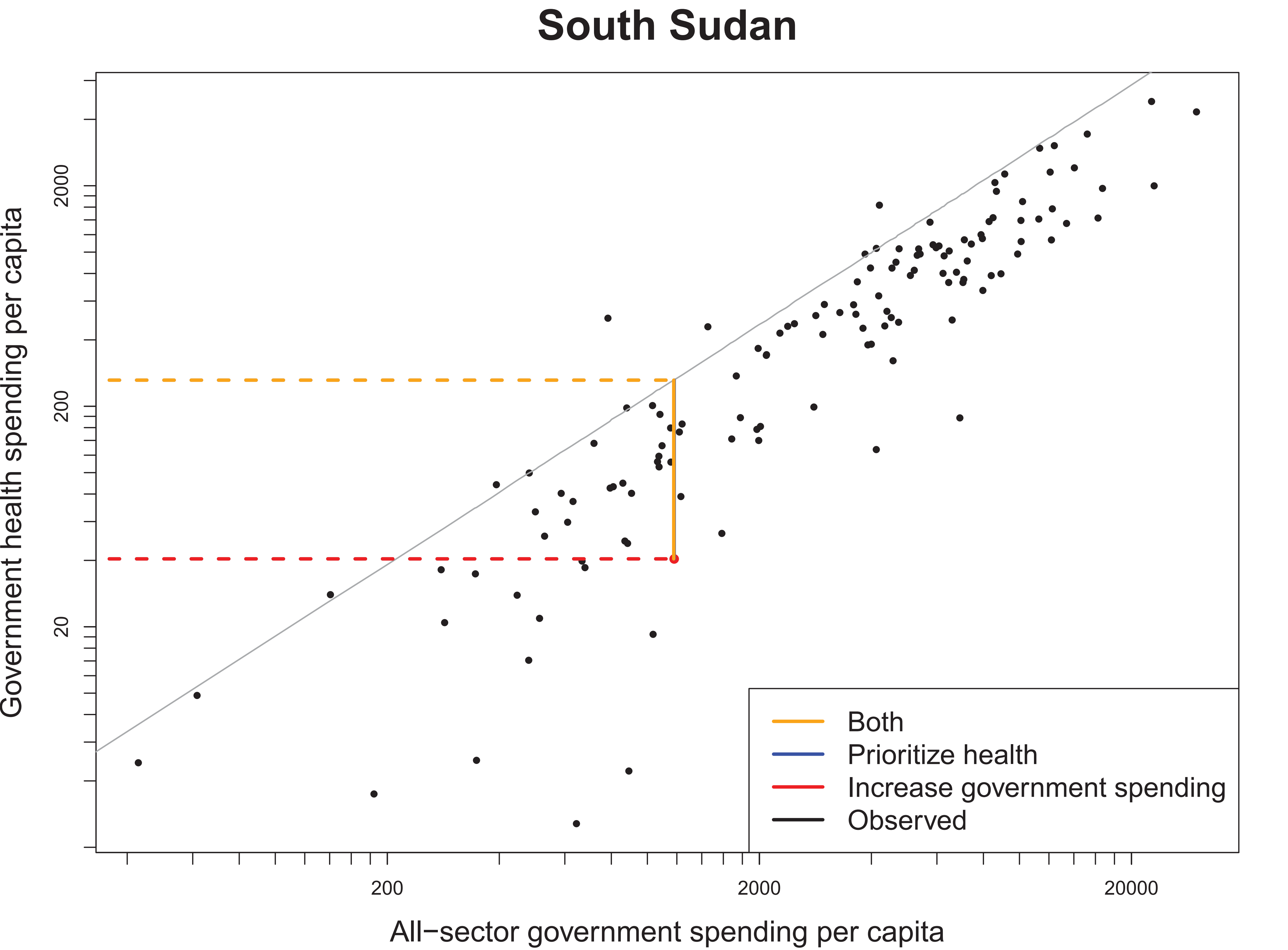

South Sudan
Government health spending per capita
20
200
2000
All−sector government spending per capita
200
2000
20000
Both
Prioritize health
Increase government spending
Observed

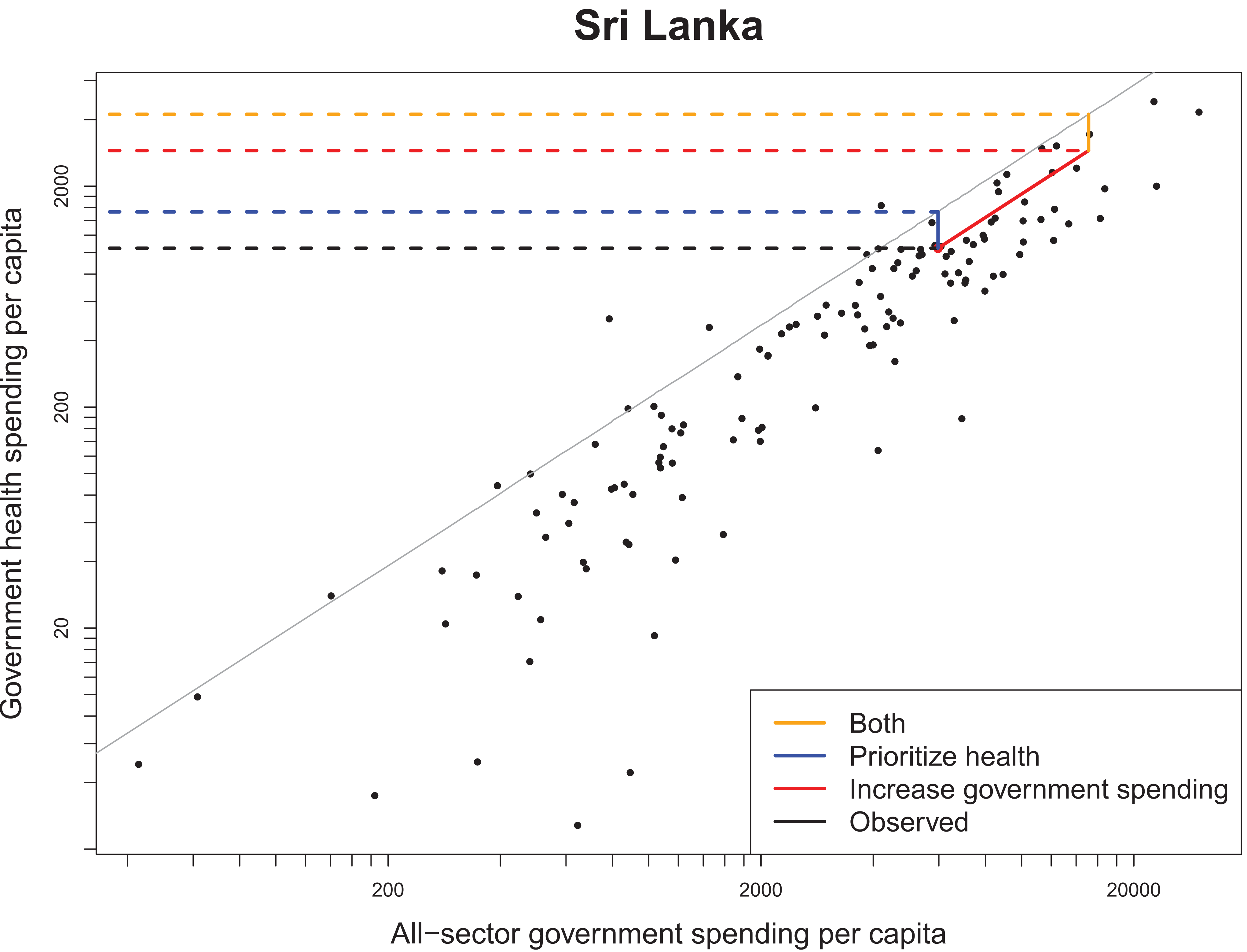
Sri Lanka
Government health spending per capita
All−sector government spending per capita
20
200
2000
20000
Both
Prioritize health
Increase government spending
Observed

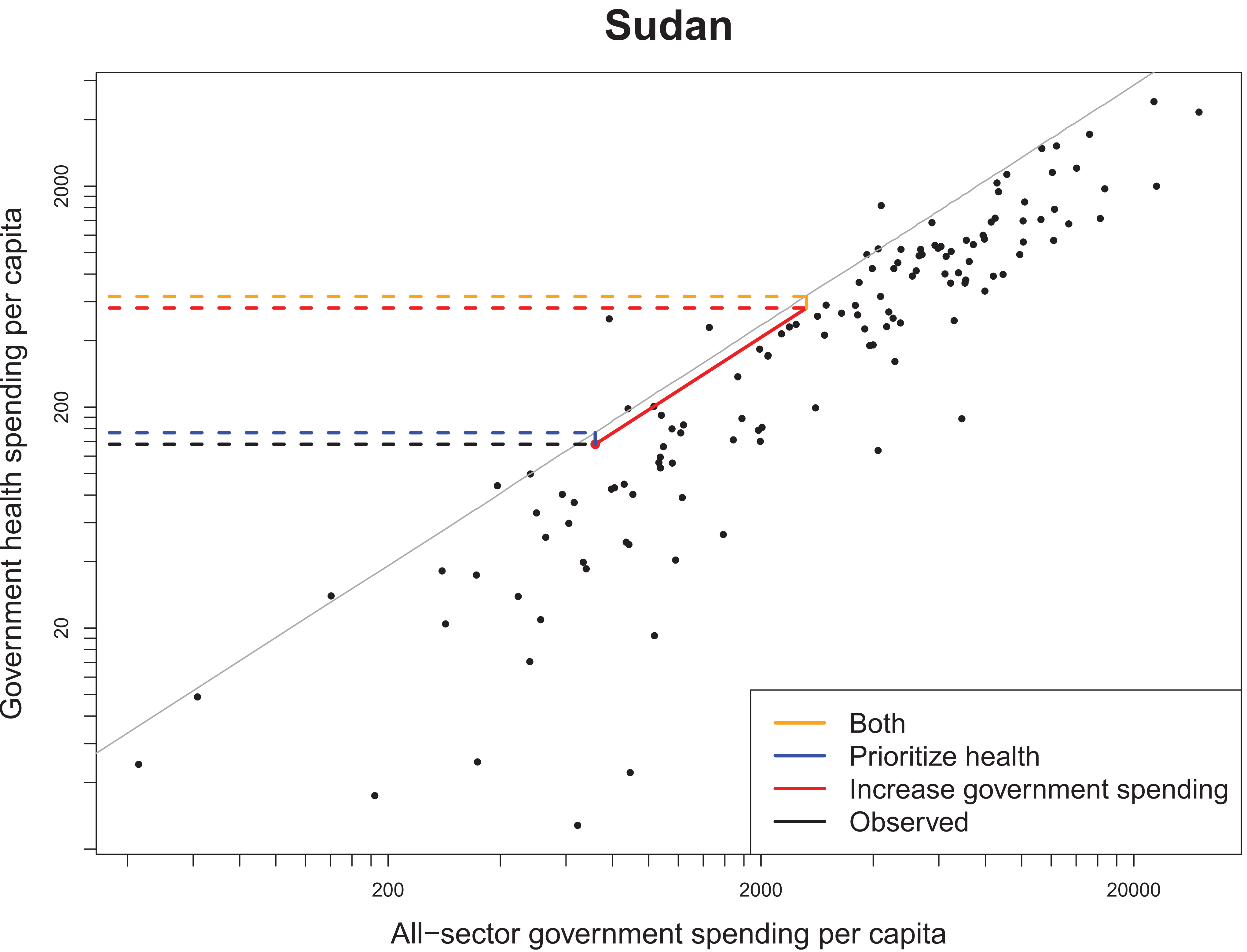

Sudan
Government health spending per capita
All−sector government spending per capita
20
200
2000
20000
Both
Prioritize health
Increase government spending
Observed

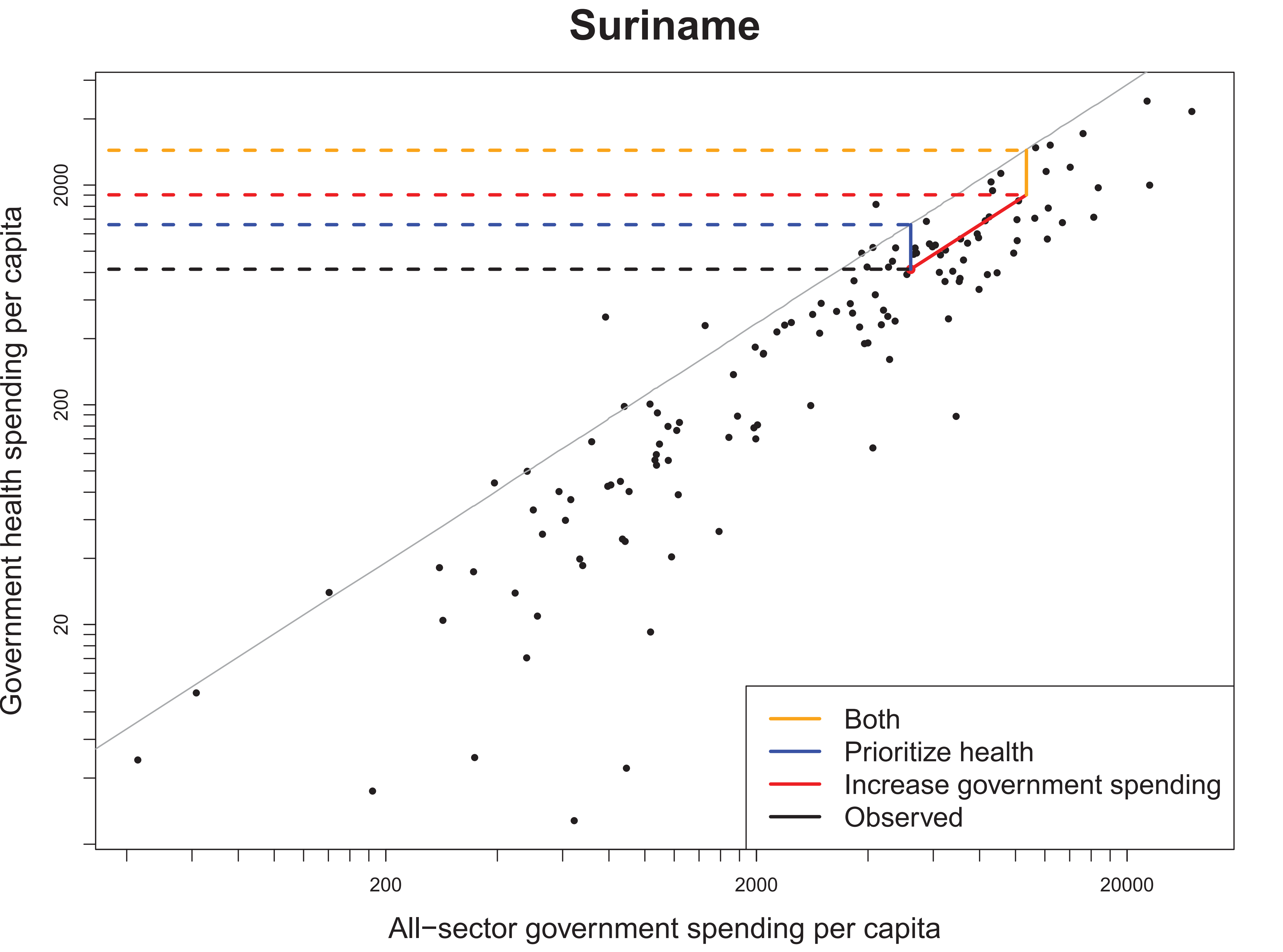

Suriname
Government health spending per capita
All−sector government spending per capita
20
200
2000
20000
Both
Prioritize health
Increase government spending
Observed

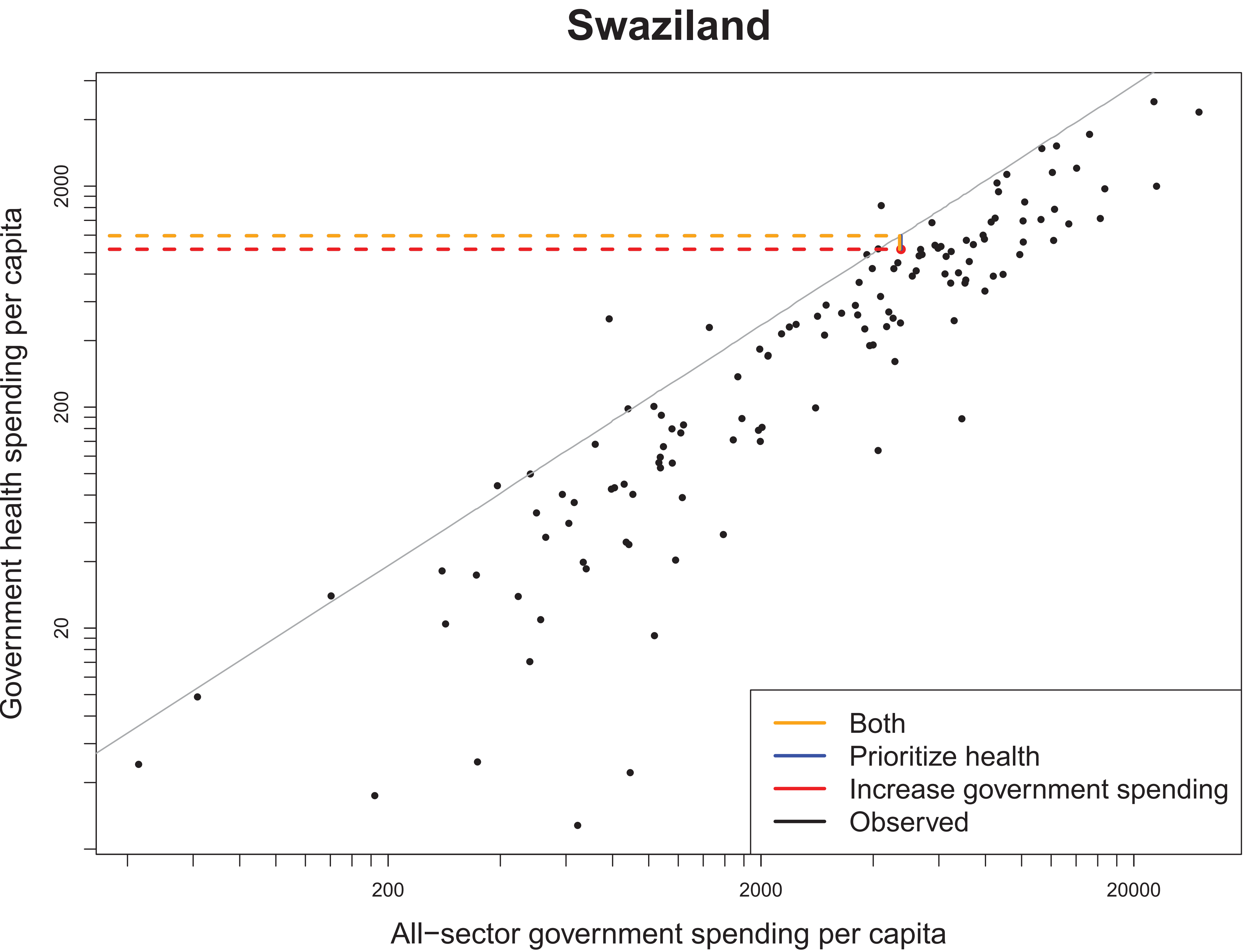

Swaziland
Government health spending per capita
2000
200
20
200
2000
20000
All−sector government spending per capita
Both
Prioritize health
Increase government spending
Observed

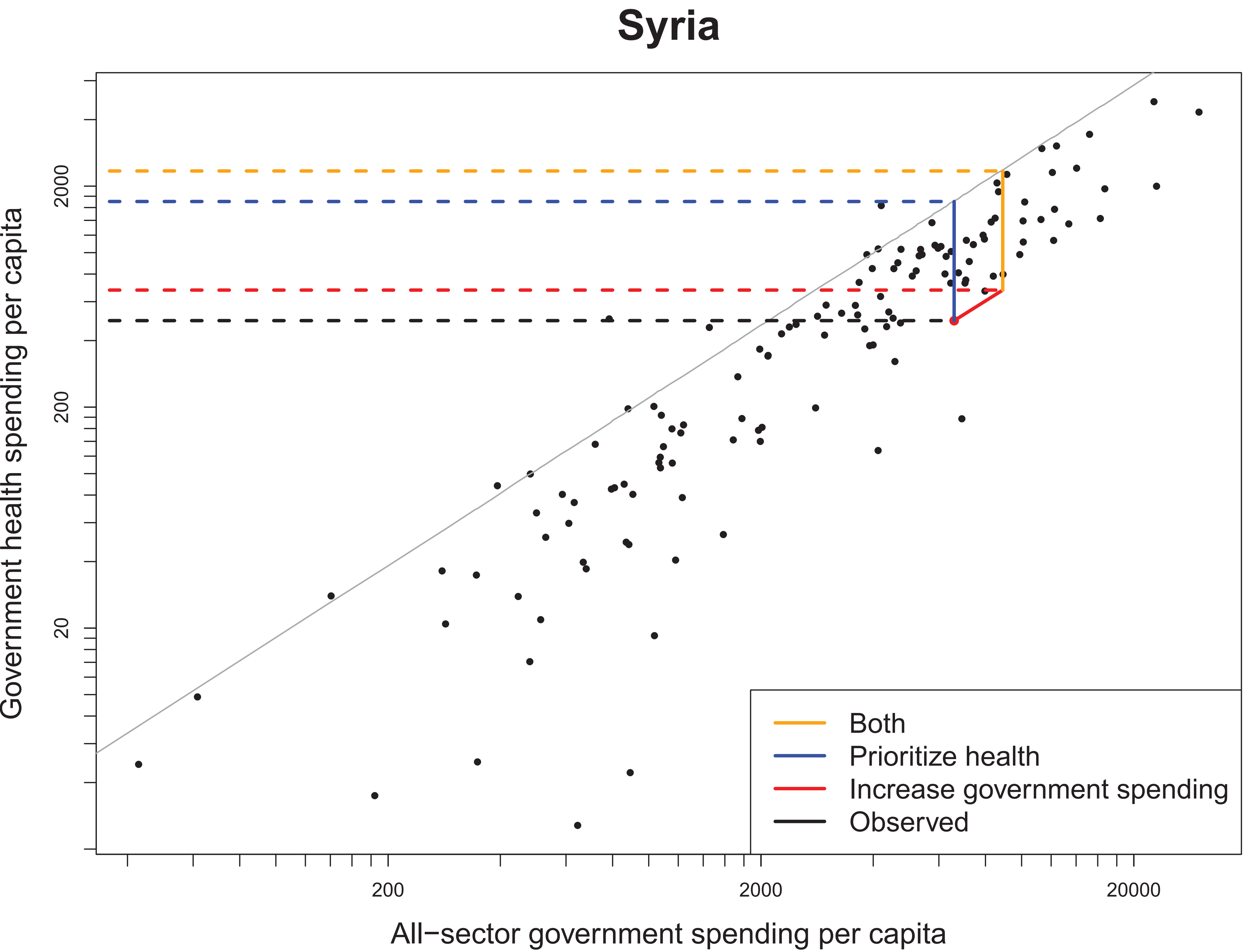

Syria
Government health spending per capita
All−sector government spending per capita
2000
200
20
200
2000
20000
Both
Prioritize health
Increase government spending
Observed

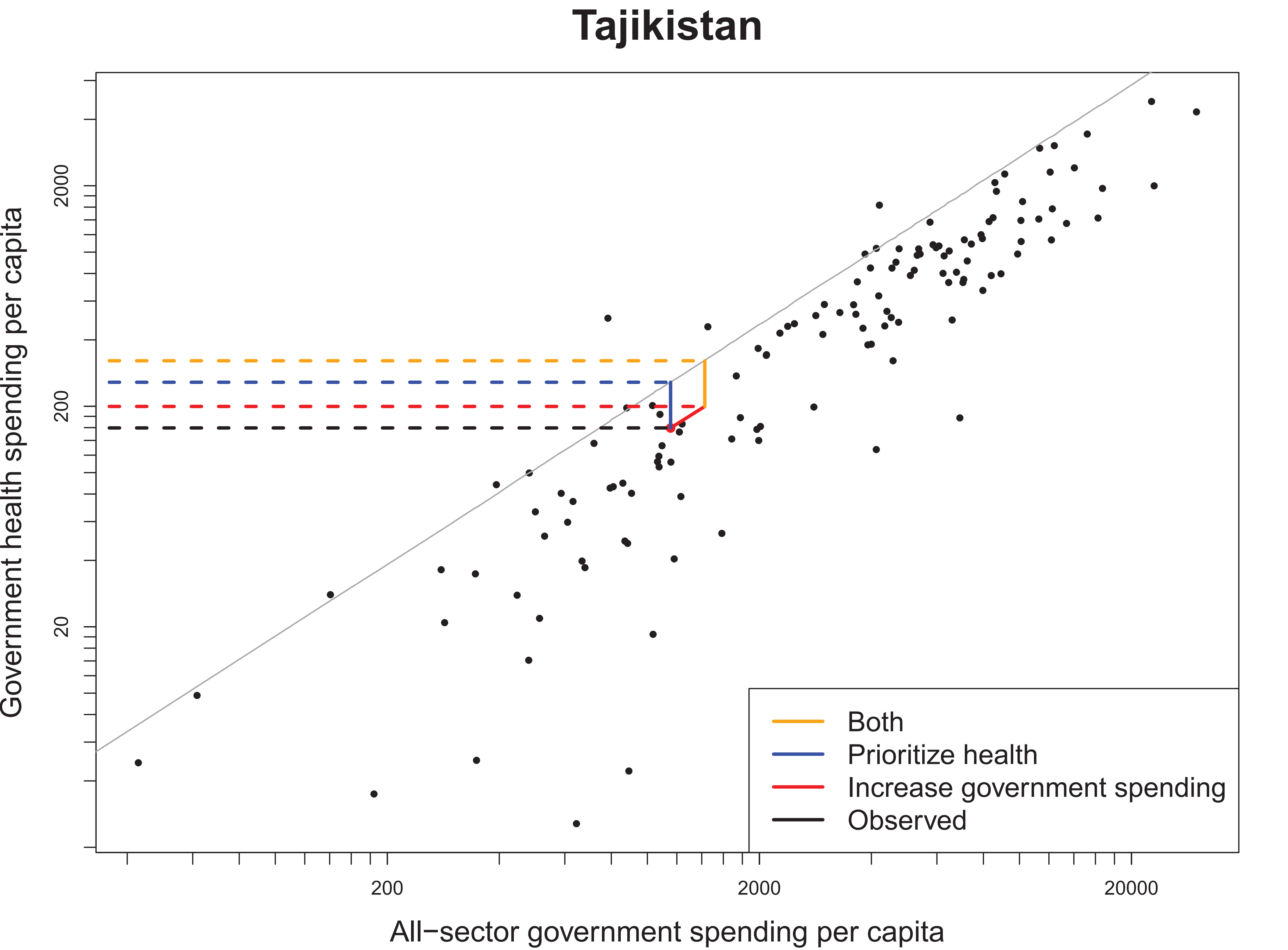

Tajikistan
Government health spending per capita
All−sector government spending per capita
20
200
2000
200
2000
20000
Both
Prioritize health
Increase government spending
Observed

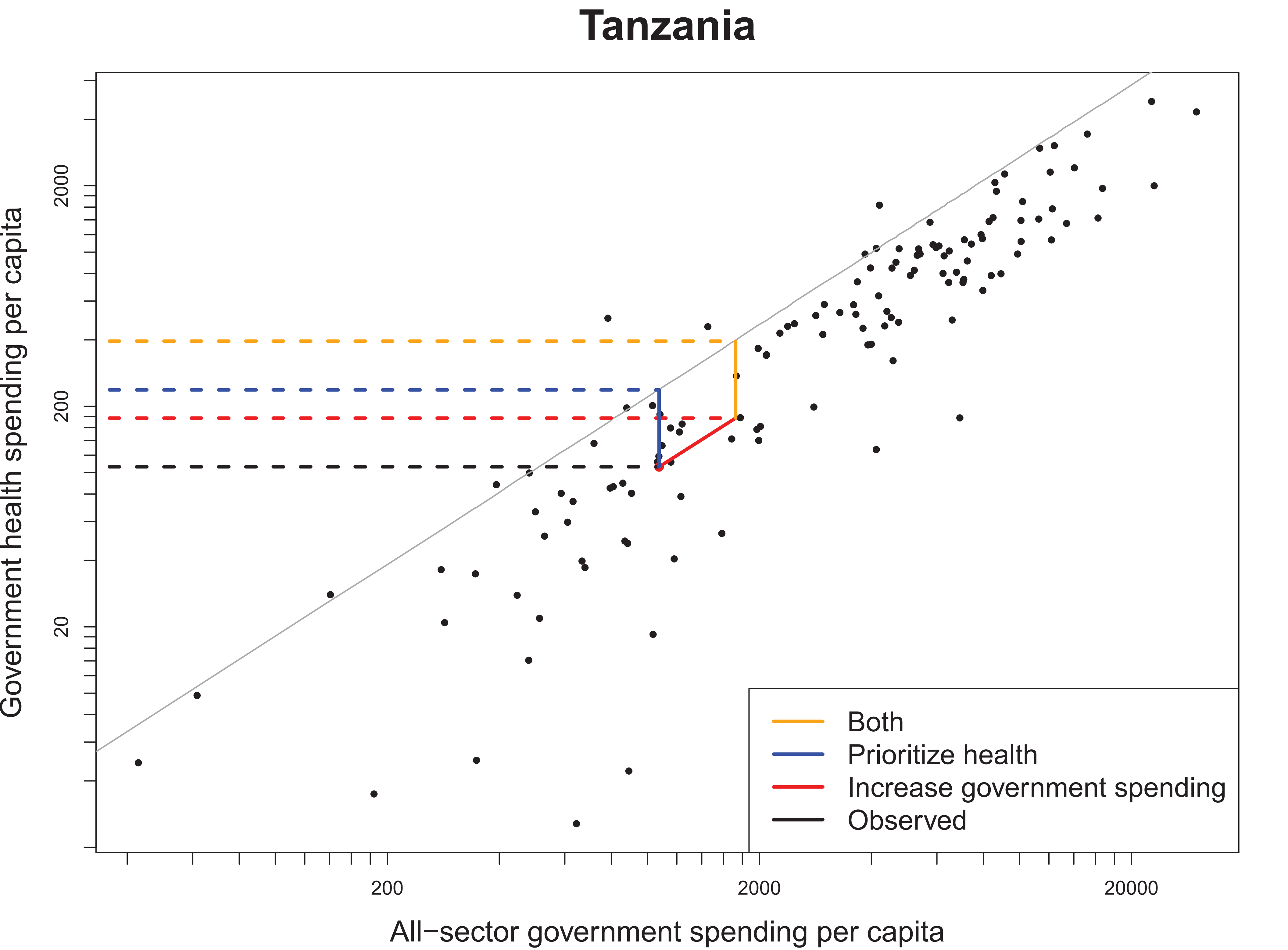

Tanzania
Government health spending per capita
All−sector government spending per capita
20
200
2000
200
2000
20000
Both
Prioritize health
Increase government spending
Observed

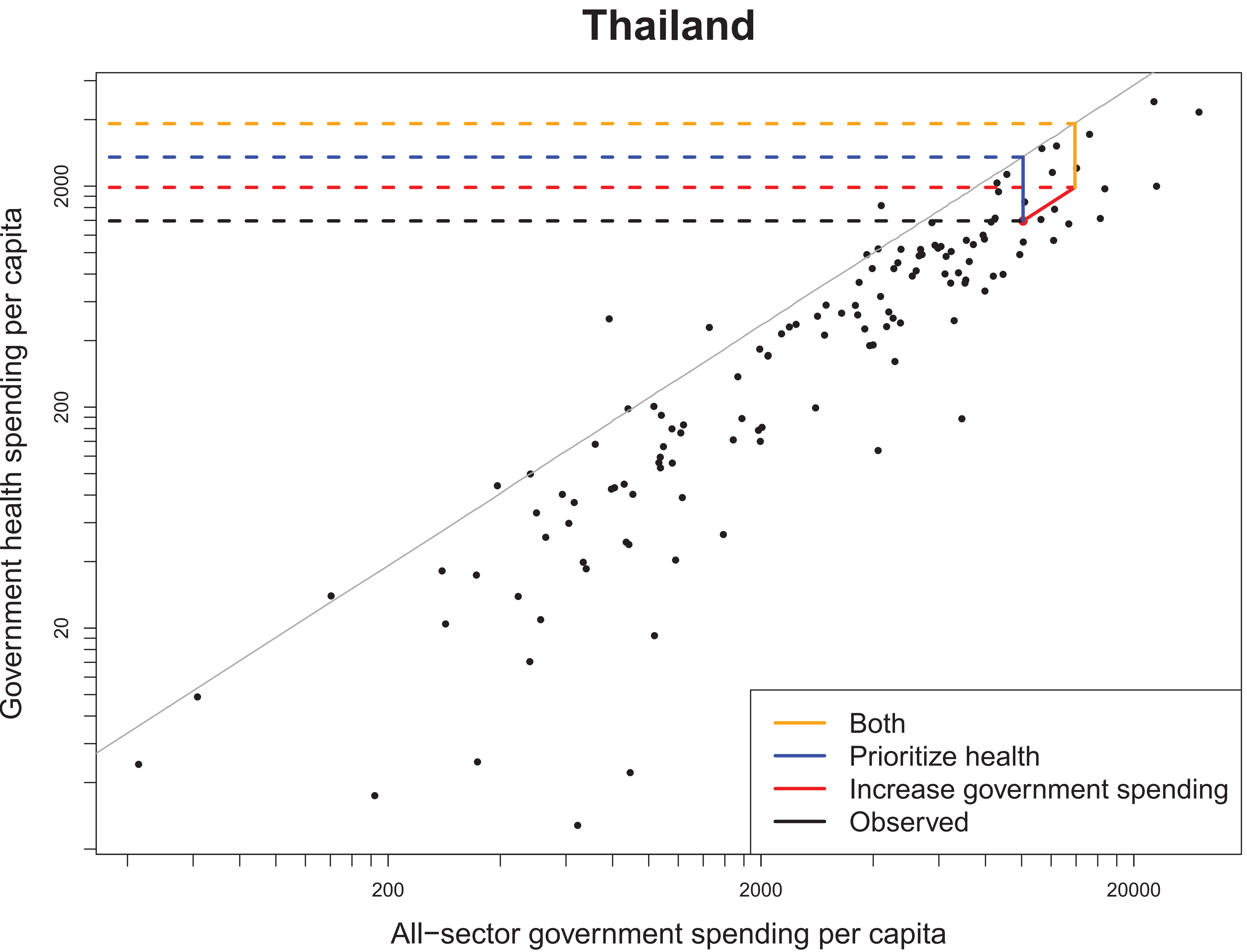
Thailand
Government health spending per capita
All−sector government spending per capita
20
200
2000
20000
Both
Prioritize health
Increase government spending
Observed

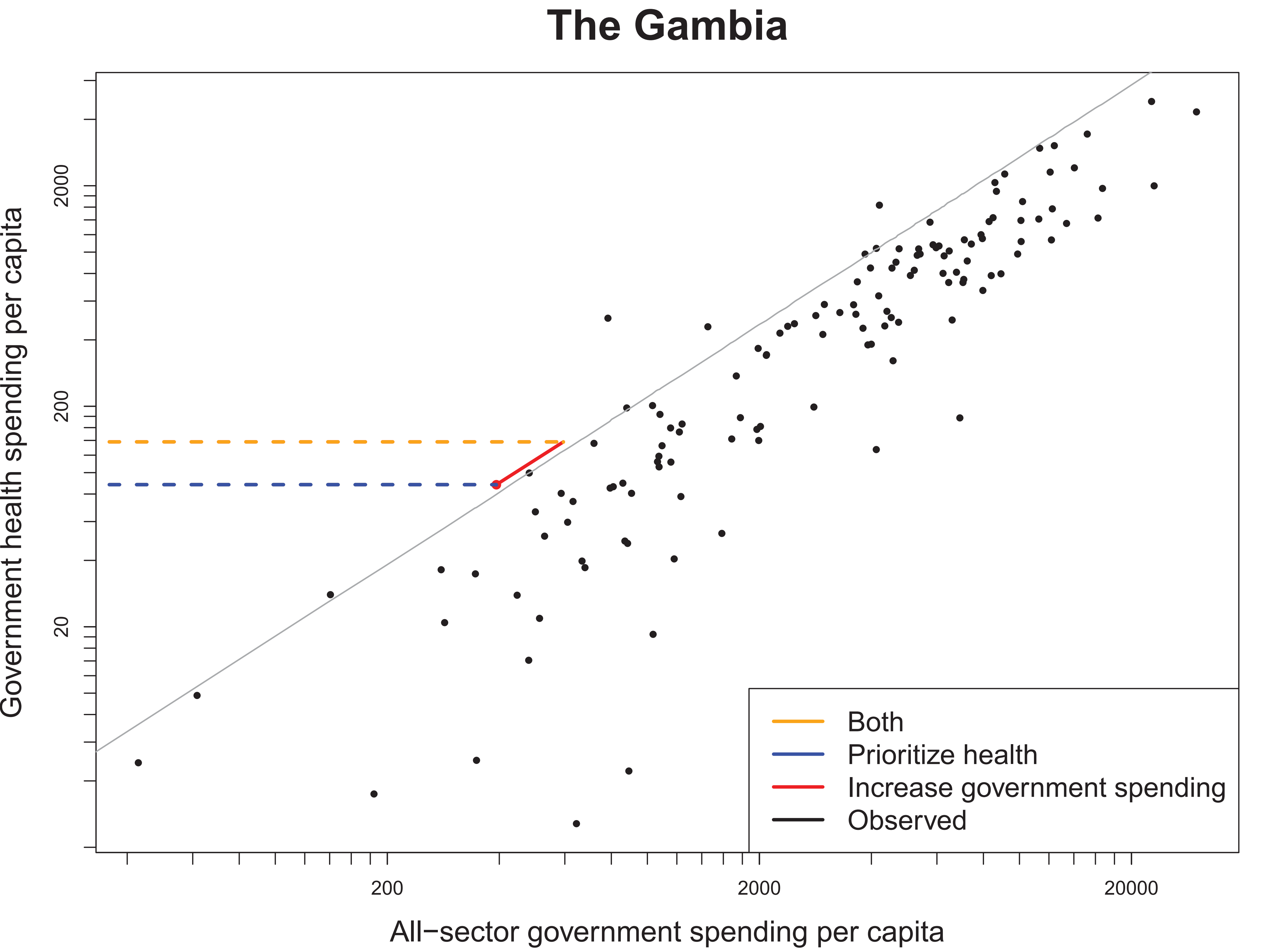
The Gambia
Government health spending per capita
All−sector government spending per capita
20
200
2000
20000
Both
Prioritize health
Increase government spending
Observed

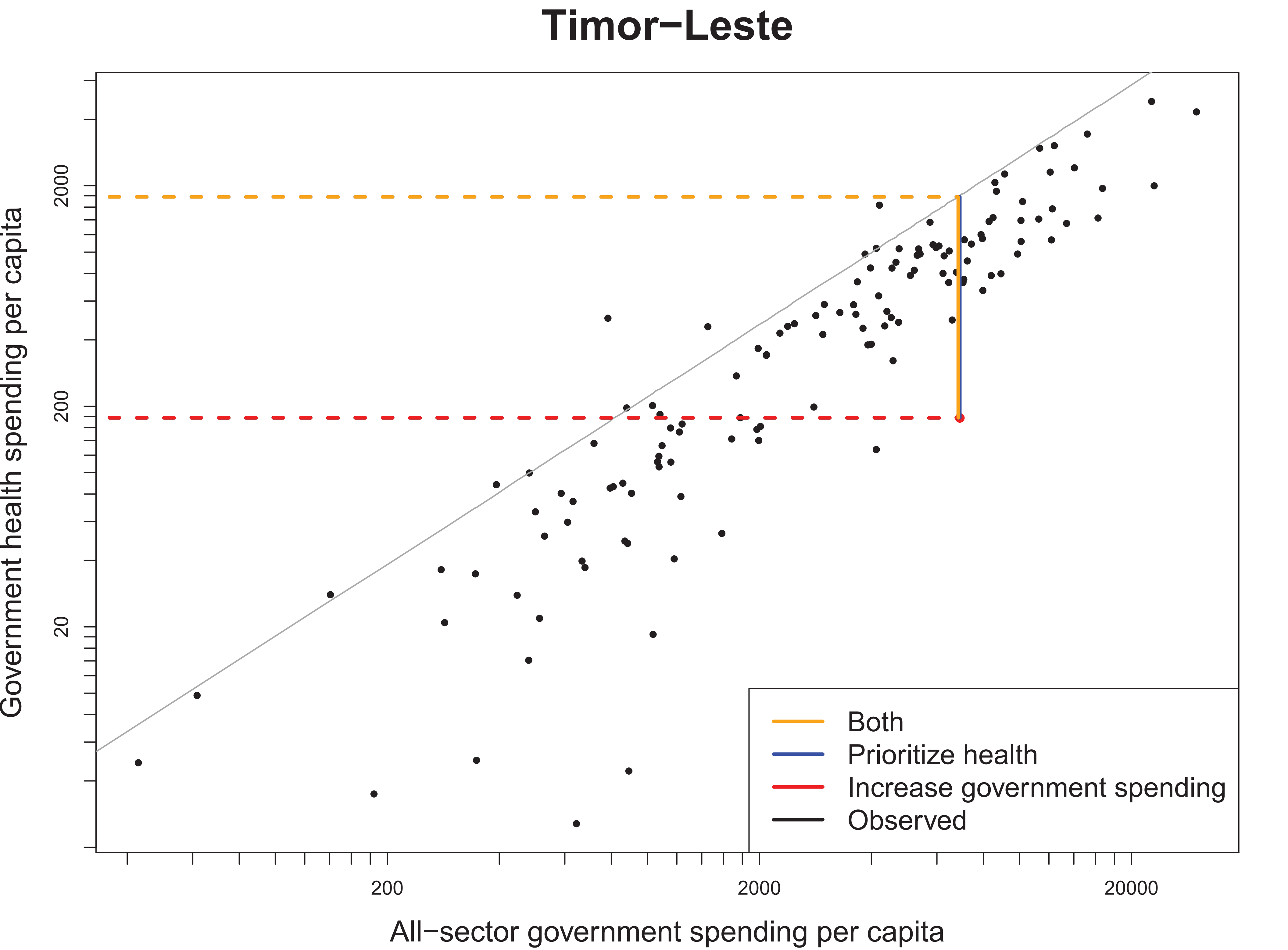
Timor−Leste
Government health spending per capita
All−sector government spending per capita
20
200
2000
20000
Both
Prioritize health
Increase government spending
Observed

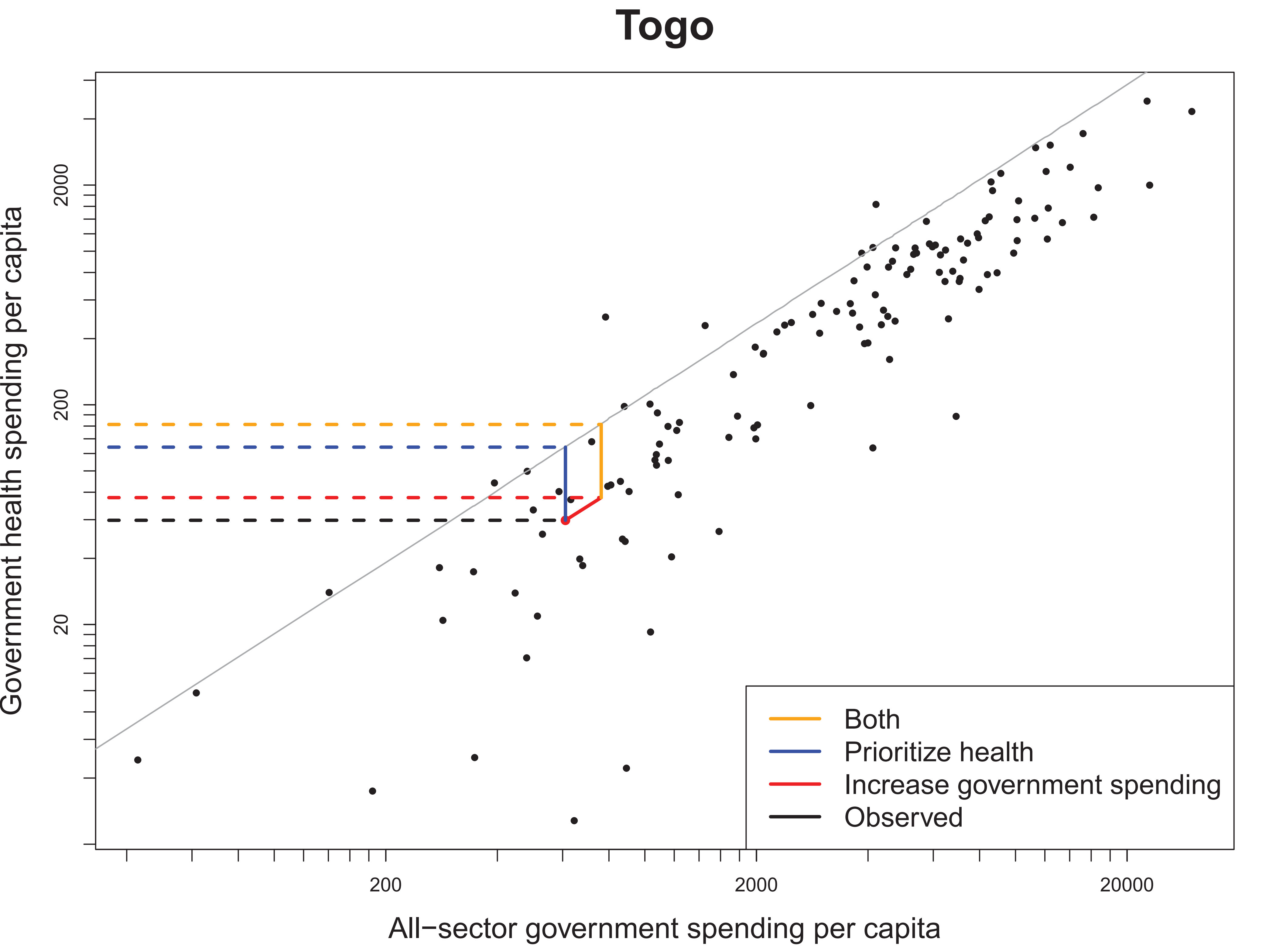

Togo
Government health spending per capita
All−sector government spending per capita
20
200
2000
20000
Both
Prioritize health
Increase government spending
Observed

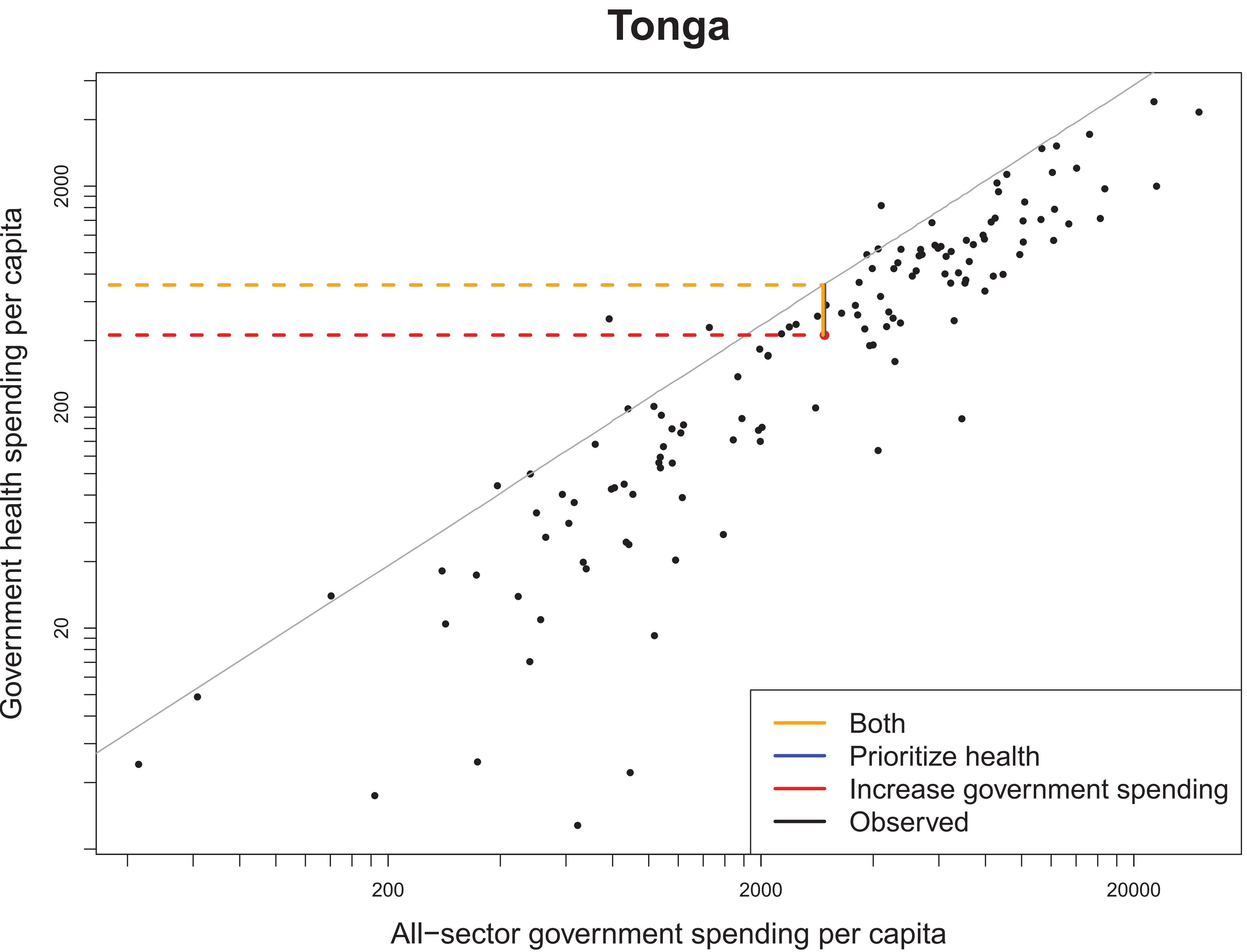

Tonga
Government health spending per capita
All−sector government spending per capita
20
200
2000
200
2000
20000
Both
Prioritize health
Increase government spending
Observed

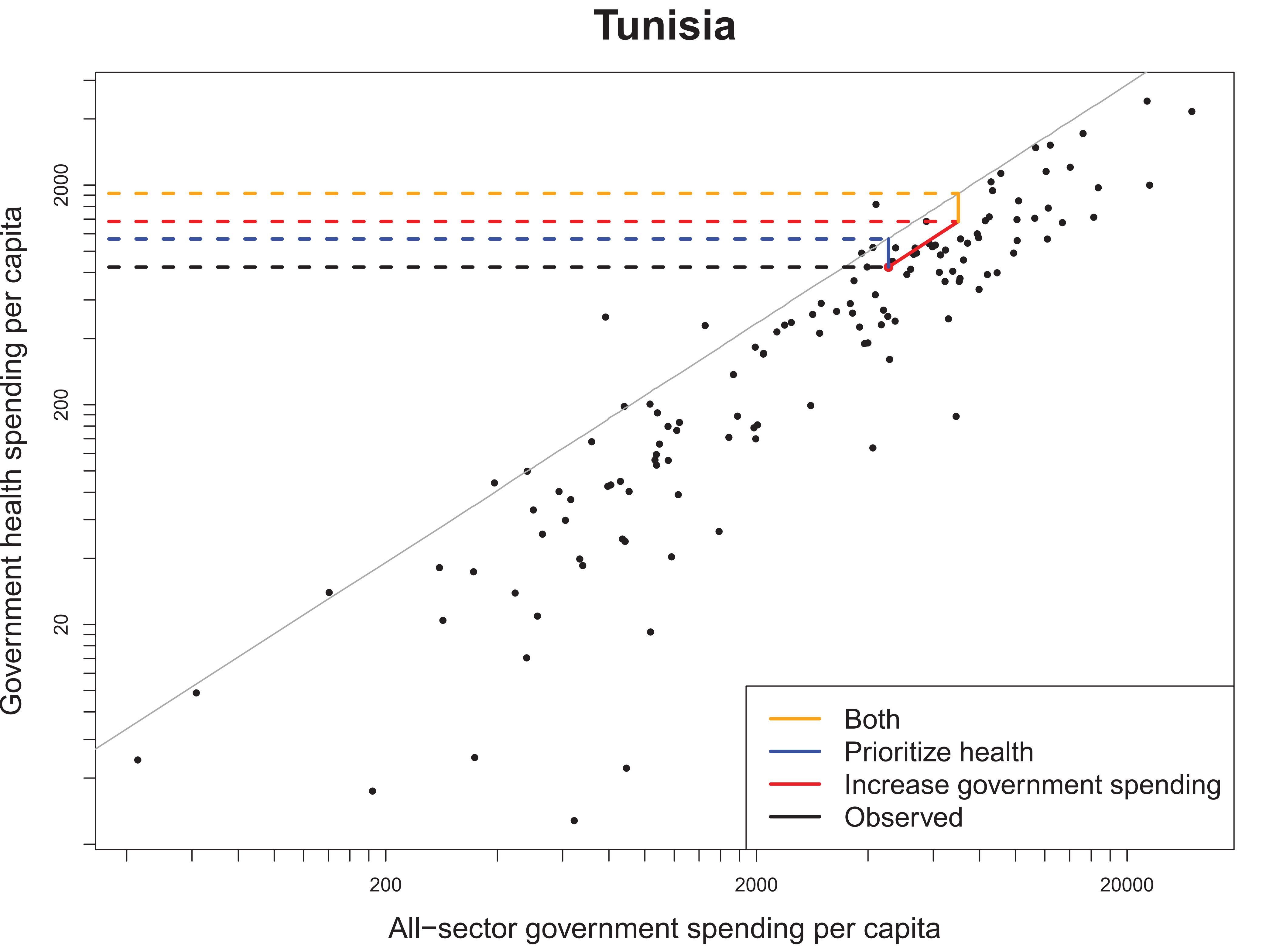
Tunisia
Government health spending per capita
All−sector government spending per capita
20
200
2000
200
2000
20000
Both
Prioritize health
Increase government spending
Observed

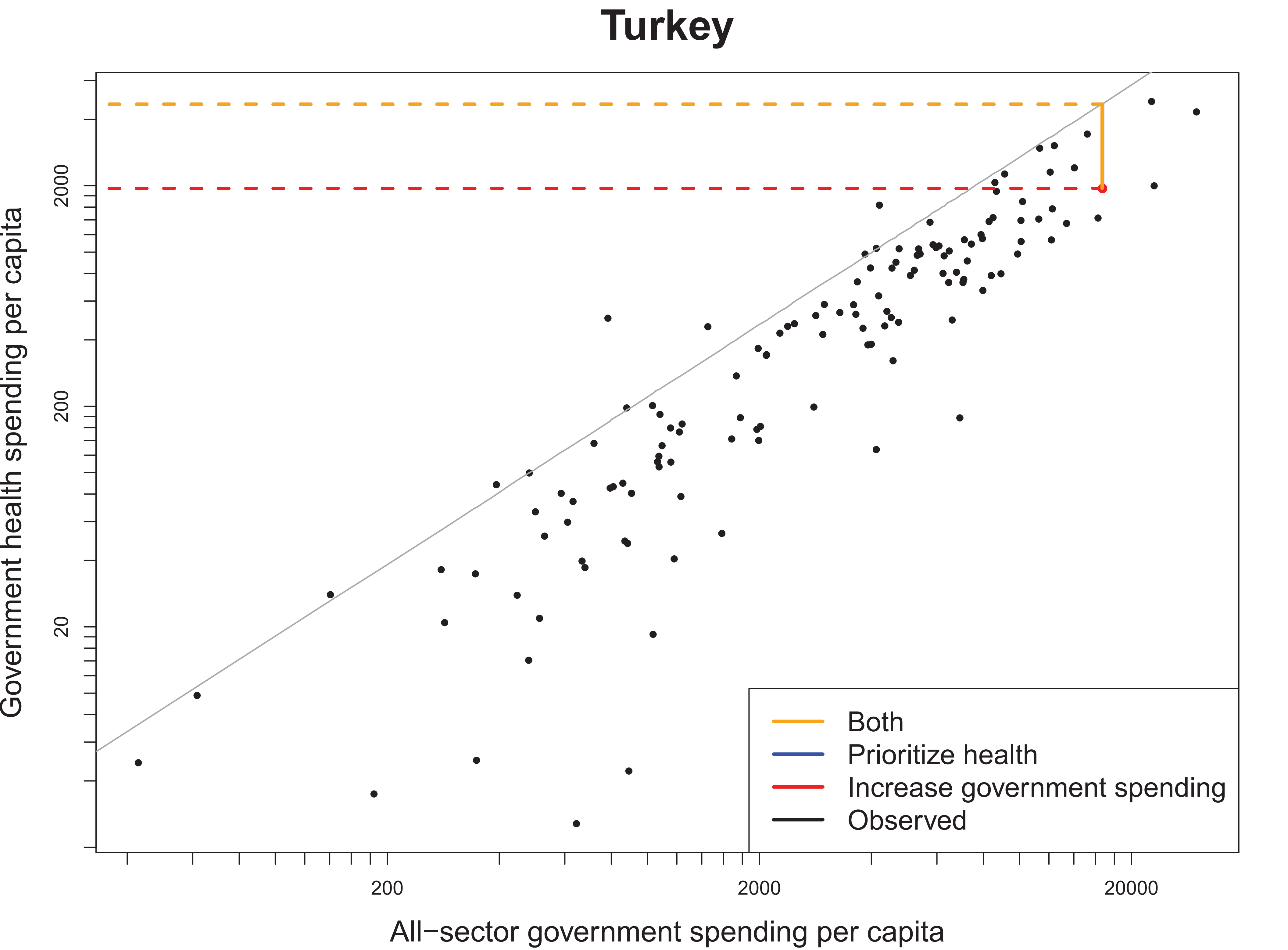

Turkey
Government health spending per capita
20
200
2000
All−sector government spending per capita
200
2000
20000
Both
Prioritize health
Increase government spending
Observed

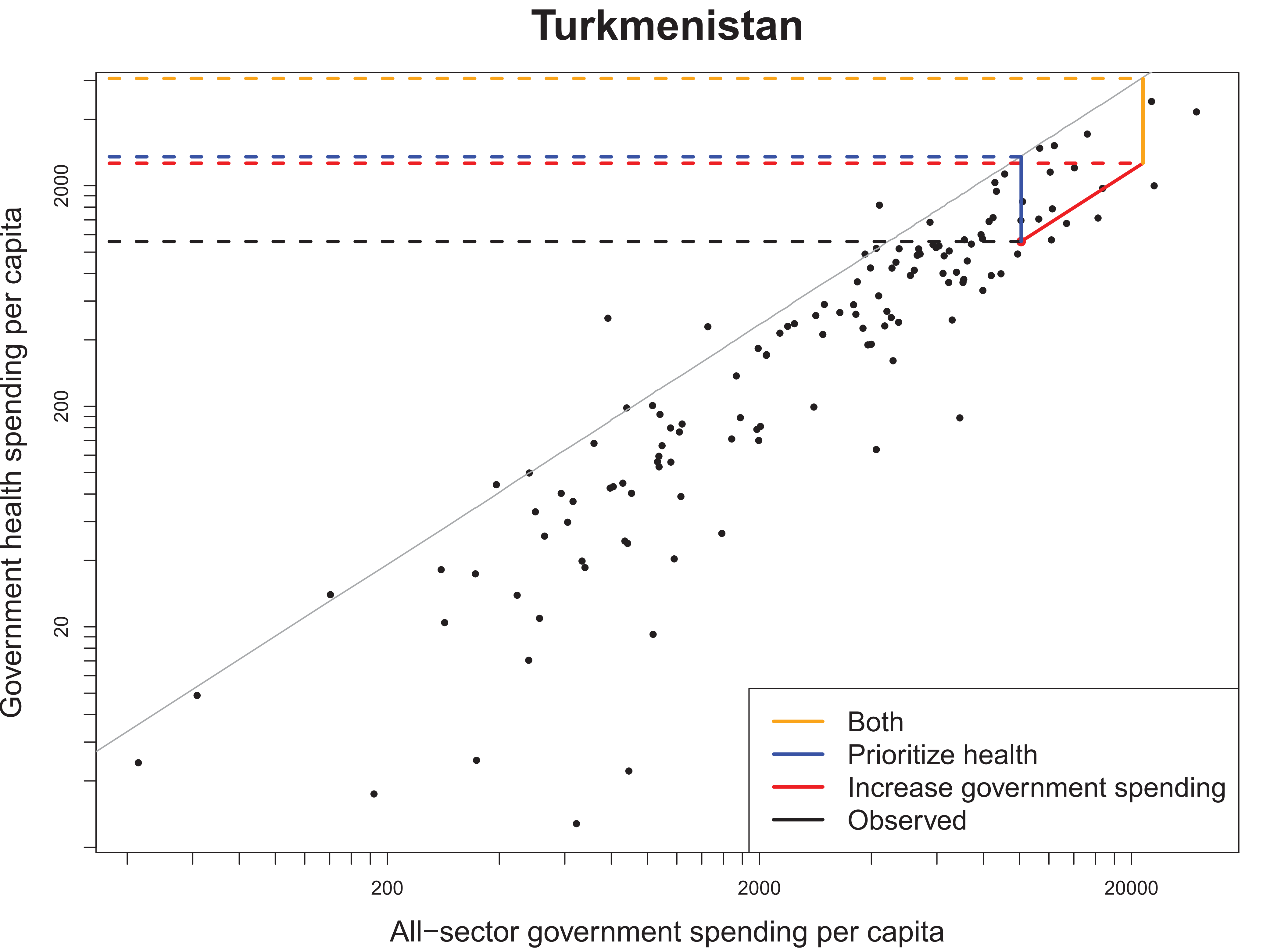

Turkmenistan
Both
Prioritize health
Increase government spending
Observed
2000
200
20
200
2000
20000
Government health spending per capita
All−sector government spending per capita

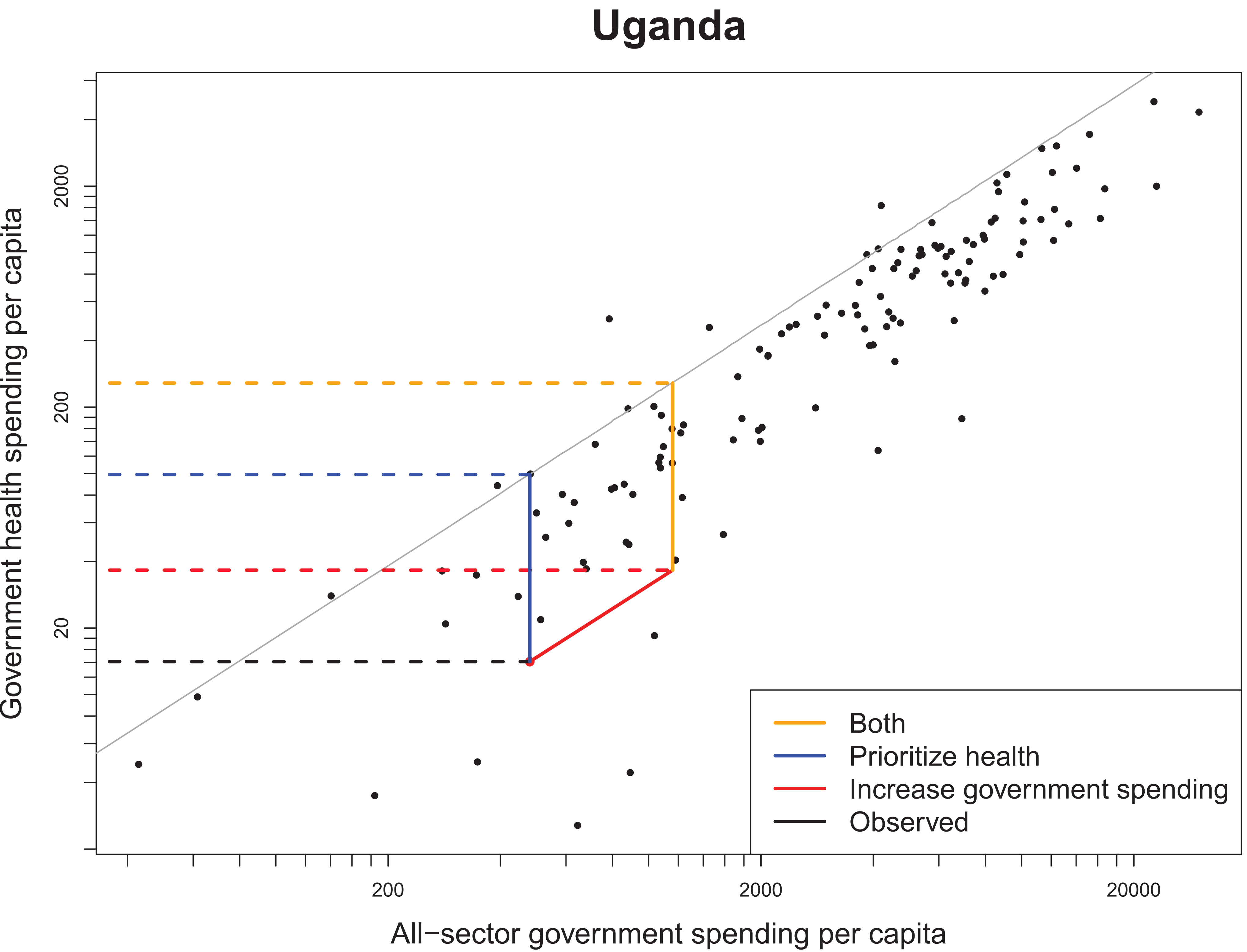

Uganda
Government health spending per capita
20
200
2000
All−sector government spending per capita
200
2000
20000
Both
Prioritize health
Increase government spending
Observed

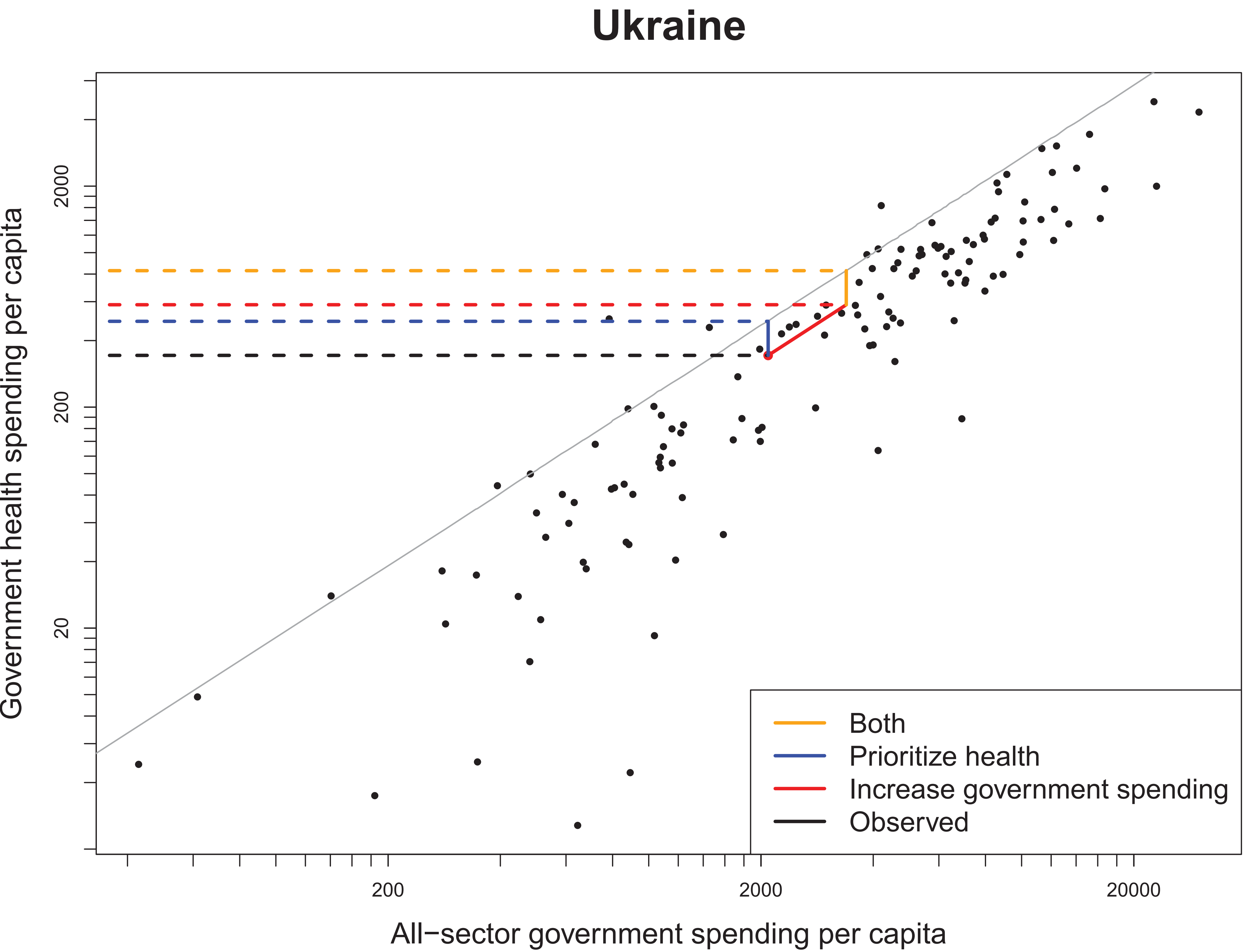

Ukraine
Government health spending per capita
All−sector government spending per capita
20
200
2000
200
2000
20000
Both
Prioritize health
Increase government spending
Observed

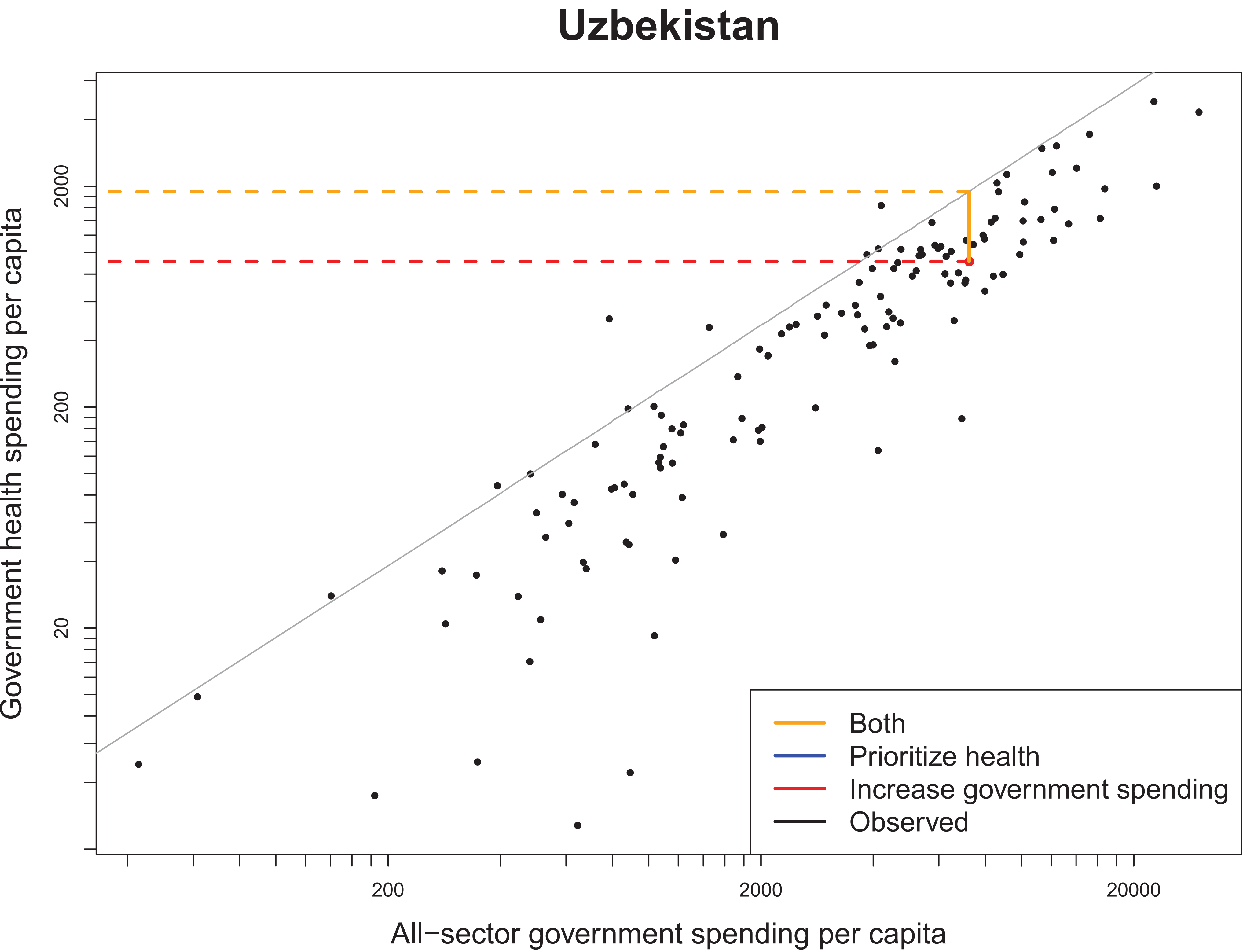
Uzbekistan
Government health spending per capita
All−sector government spending per capita
20
200
2000
200
2000
20000
Both
Prioritize health
Increase government spending
Observed

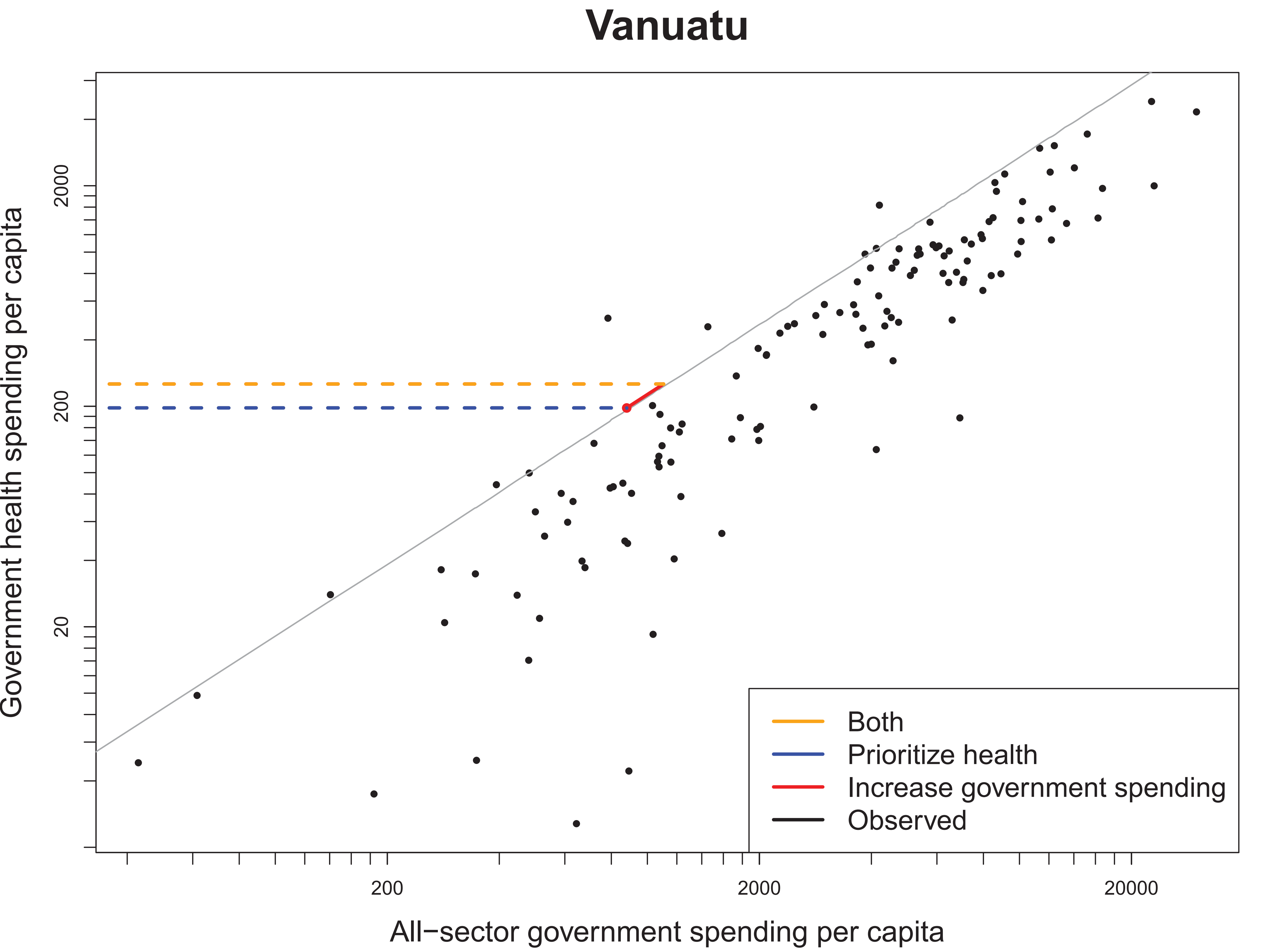

Vanuatu
Government health spending per capita
20
200
2000
200
2000
20000
All−sector government spending per capita
Both
Prioritize health
Increase government spending
Observed

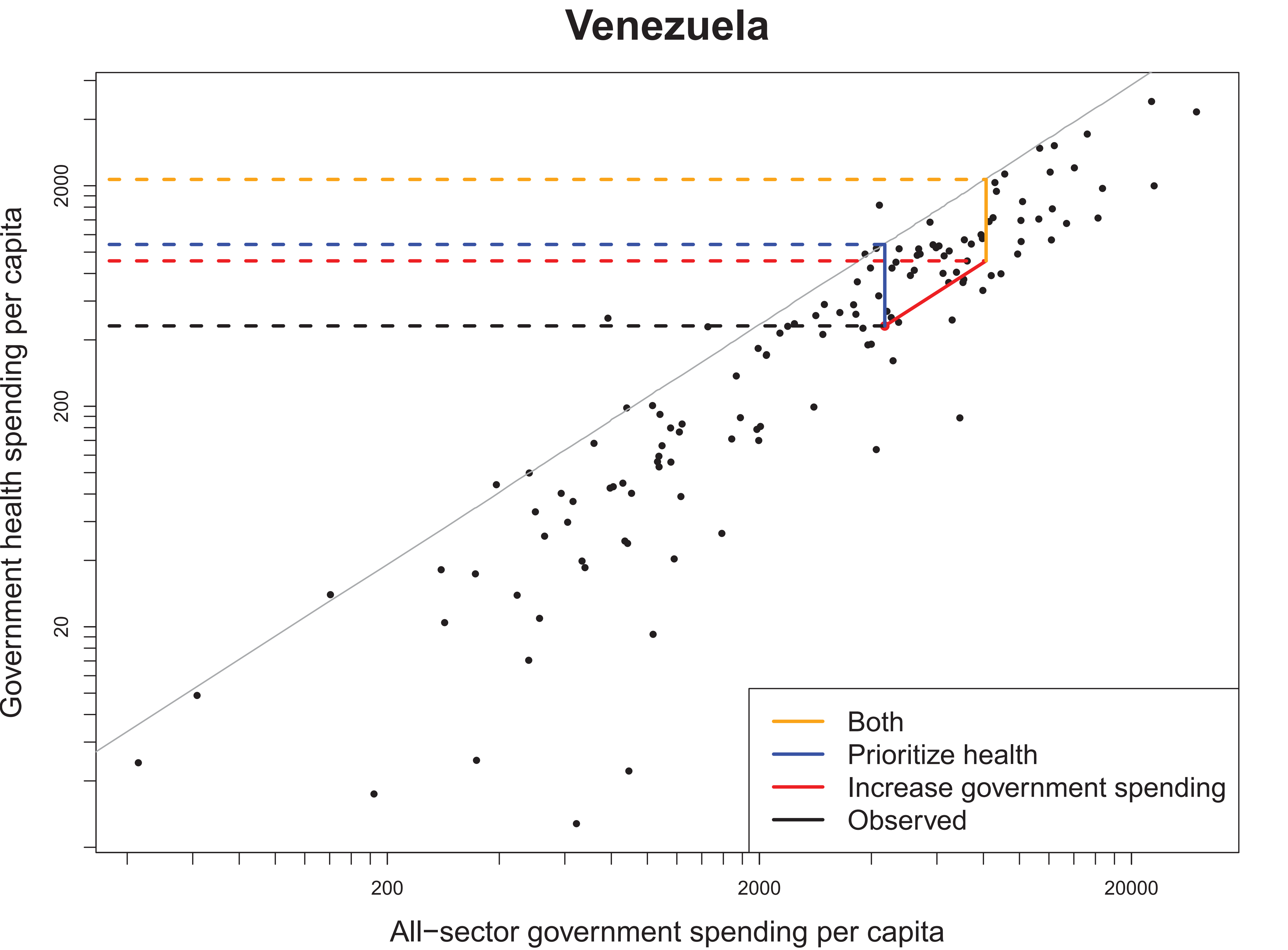

Venezuela
Government health spending per capita
All−sector government spending per capita
20
200
2000
200
2000
20000
Both
Prioritize health
Increase government spending
Observed

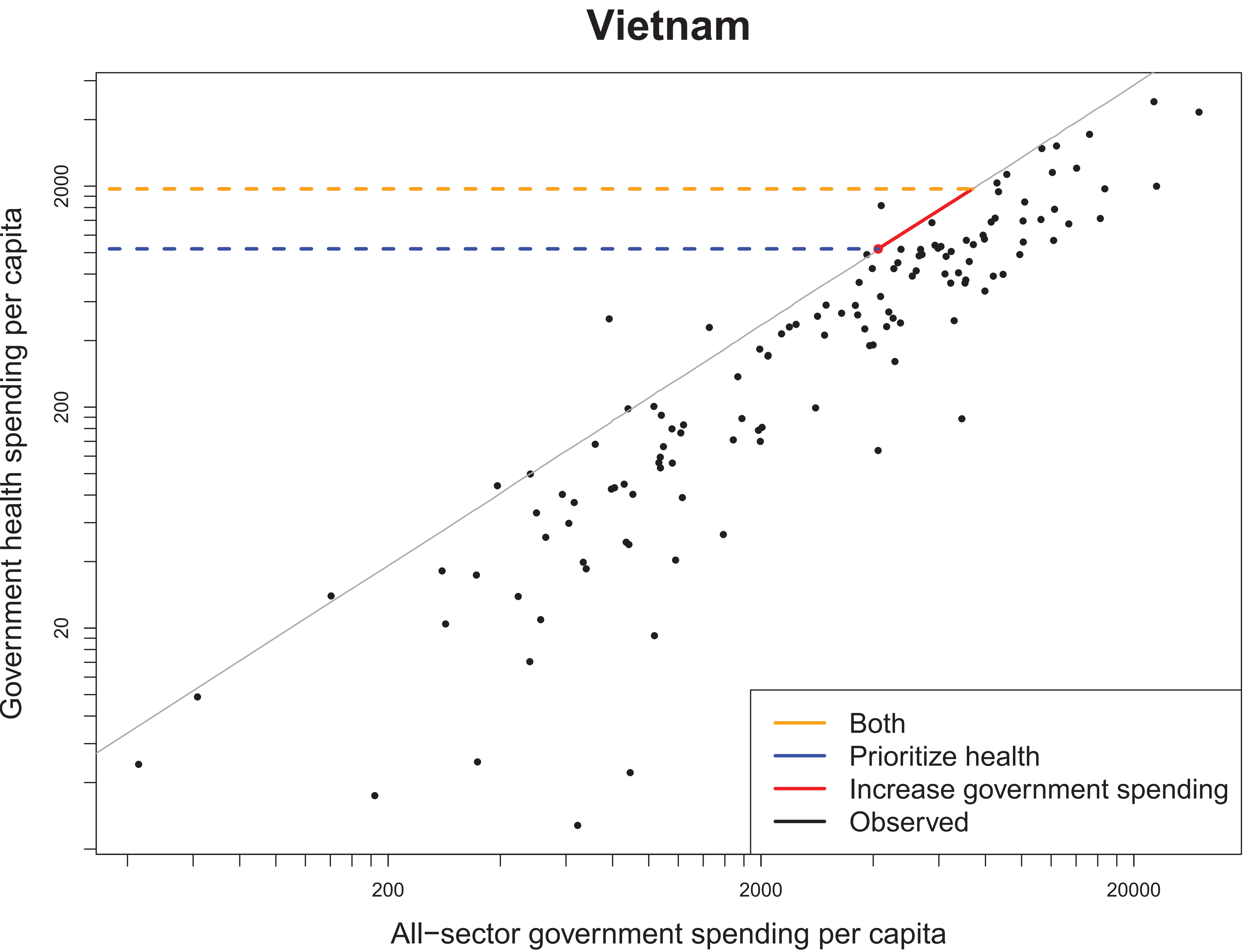

Vietnam
Government health spending per capita
20
200
2000
All−sector government spending per capita
200
2000
20000
Both
Prioritize health
Increase government spending
Observed

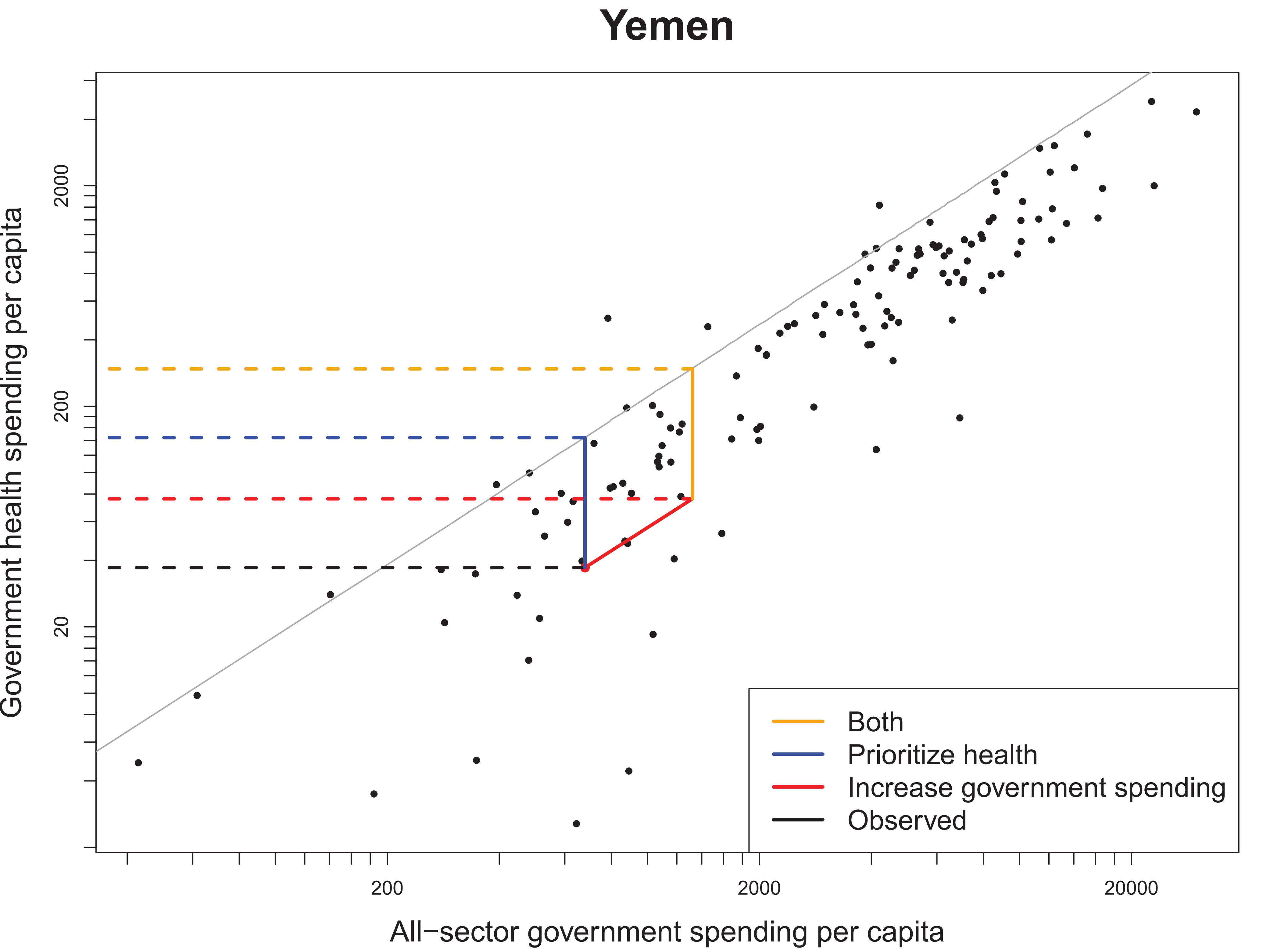

Yemen
Government health spending per capita
20
200
2000
All−sector government spending per capita
200
2000
20000
Both
Prioritize health
Increase government spending
Observed

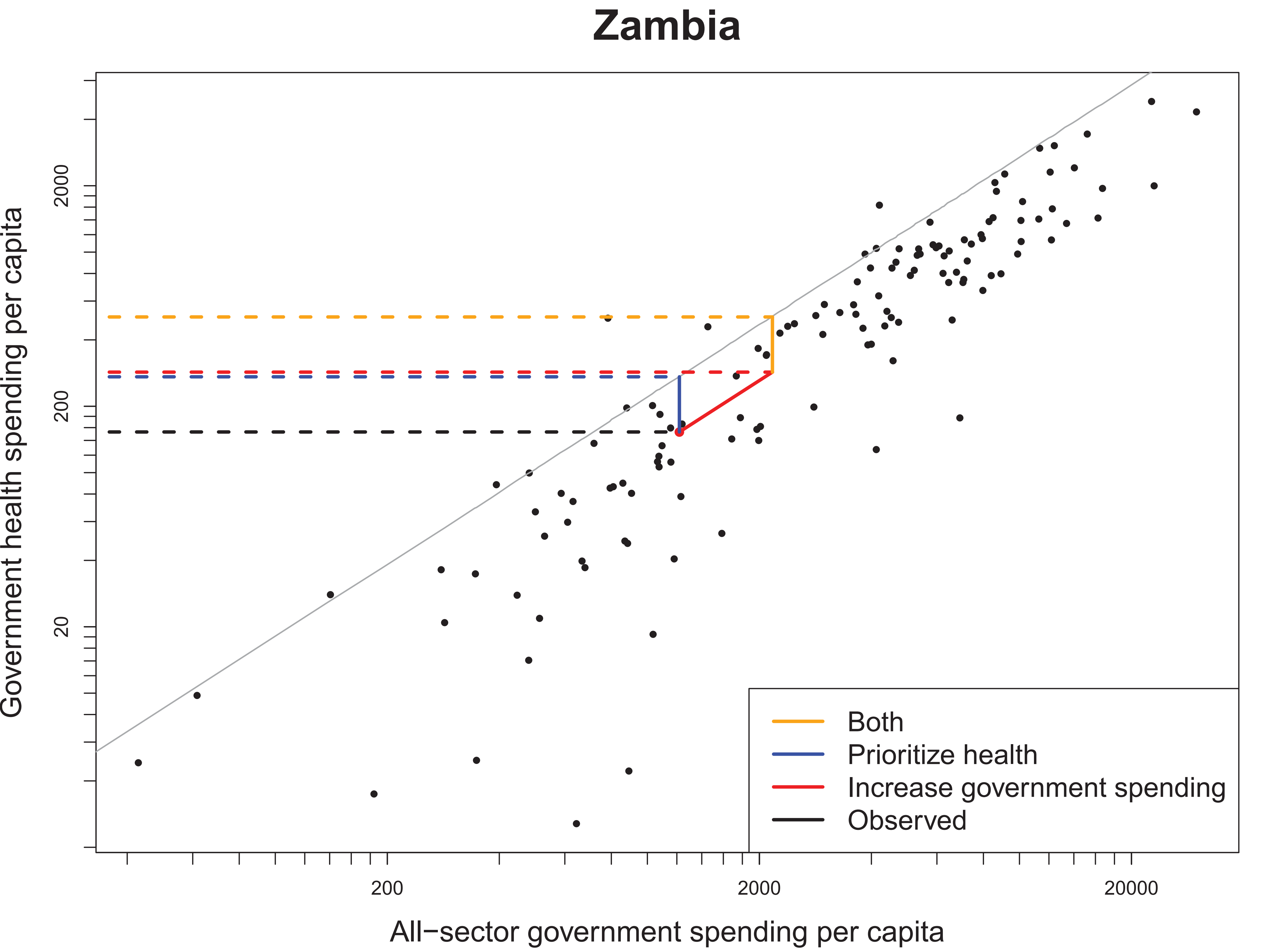

Zambia
Government health spending per capita
All−sector government spending per capita
20
200
2000
20000
Both
Prioritize health
Increase government spending
Observed
